# A heterometallic σ-silane adduct from cooperative reactivity of an iron–aluminium complex

**DOI:** 10.1039/d5cc06456b

**Published:** 2025-12-01

**Authors:** Benedek Stadler, Mark R. Crimmin

**Affiliations:** a Department of Chemistry, Molecular Sciences Research Hub, Imperial College 82 Wood Lane, Shepherds Bush London W12 0BZ UK m.crimmin@imperial.ac.uk

## Abstract

Protonation of a heterometallic silanide complex with an alcohol led to formation of a σ-silane complex. This reaction does not require a strong acid or a weakly coordinating anion. Both metals of the heterometallic complex play an important role, with the aluminium centre sequestering the anion generated in the protonation step.

## Abstract 2

Protonation of a heterometallic silanide complex with an alcohol led to formation of a σ-silane complex. This reaction does not require a strong acid or weakly coordinating anion.

## Graphical abstract

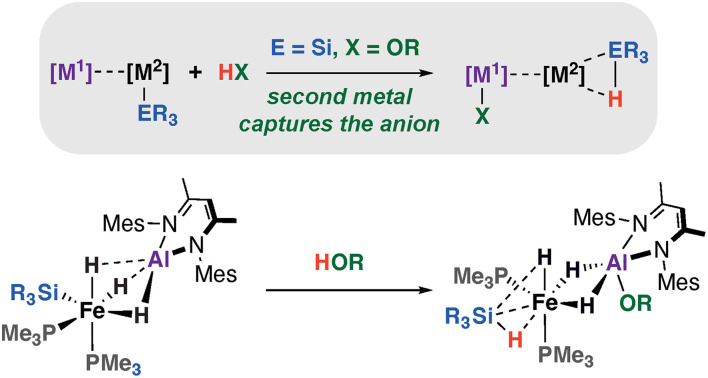

Weakly coordinating anions play a pivotal role in contemporary synthesis.^1–4^ These species are charge diffuse and associate only modestly with their cationic components, allowing the stabilisation of reactive species. For example, weakly coordinating anions have proven essential in the synthesis of σ-complexes of transition metals.^5–12^ Protonation of a metal-based ligand with a strong acid containing a weakly coordinating anion is a widely used strategy to generate σ-complexes that would otherwise be unstable due to competition for the metal coordination site by the anion. This method has been applied to the generation of dihydrogen,^5–7^ σ-alkane,^8,9^ and σ-silane^10–12^ complexes through protonation of hydride, alkyl, and silanide ligands, respectively. While these approaches have led to some remarkable advances, they largely rely on synthetically complex anions used in non-coordinating solvents and have been applied to single-site transition metal complexes (Fig. 1a). As part of our ongoing studies into the properties and reactivity of heterometallic complexes containing both a transition metal and main group metal in close proximity,^13–15^ we recently uncovered an alternative approach to stabilise silane σ-complexes through exploitation of the cooperative reactivity of the two metal sites (Fig. 1b).

**Fig. 1:**
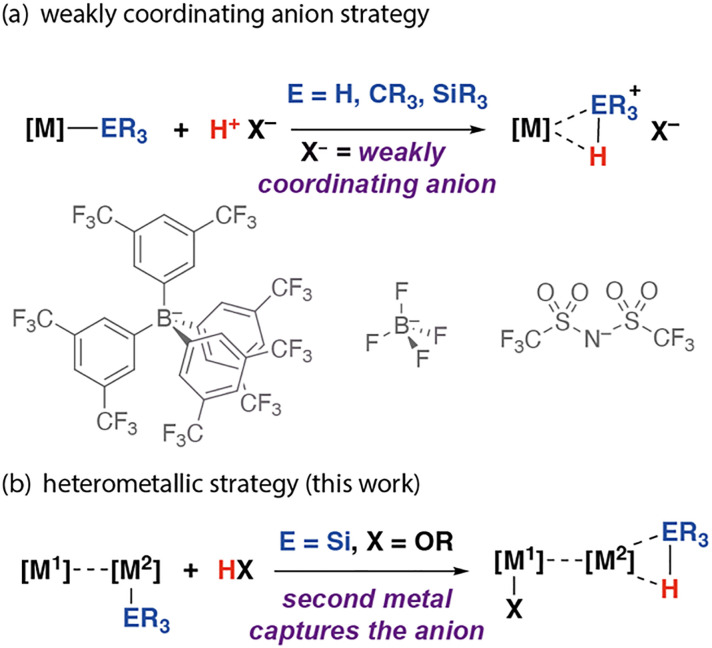
Synthetic approaches to σ-complexes using protonation with (a) monometallic complexes using weakly coordinating anions and (b) heterometallic complexes (this work).

Herein we report that the reaction of the iron–aluminium heterometallic complex 1 with silanes followed by protonation allows facile access to the corresponding σ-silane complexes. This cooperative behaviour imitates reactivity that typically relies on use of weakly coordinating anions and strong acids. Both metals of the heterometallic complex both play an important role, with the aluminium centre sequestering the anion generated in the protonation step. The approach allows the formation of a rare example of a σ-silane complex of iron.^16–20^ Moreover, it suggests that heterometallic complexes may offer new strategies to access previously inaccessible species through intramolecular cooperativity.

The heterometallic complex 1 possesses a polarised Fe^II^–Al^I^ bond. It is highly basic and nucleophilic at Fe and capable of reacting with both pyridines and alkenes through C–H bond activation.^13,14^ Curious as to whether such reactivity could be expanded to Si–H bonds, the reaction of 1 with a small array of aryl silanes (a: PhSiH_3_; b: Ph_2_SiH_2_; c: Ph_3_SiH) was investigated. These reactions proceeded efficiently in benzene-*d*_6_ over 16–72 h at 60 °C to yield the corresponding heterometallic silanide complexes 2a–c along with one equiv. of PMe_3_ as the sole byproduct (Scheme 1). 2a–c could be isolated in modest yields following recrystallisation from mixtures of Et_2_O and hydrocarbon solvents. 2a–b react with isocyanates and carbodiimides to yield 3a–d, products of hydrosilylation.

**Scheme 1:**
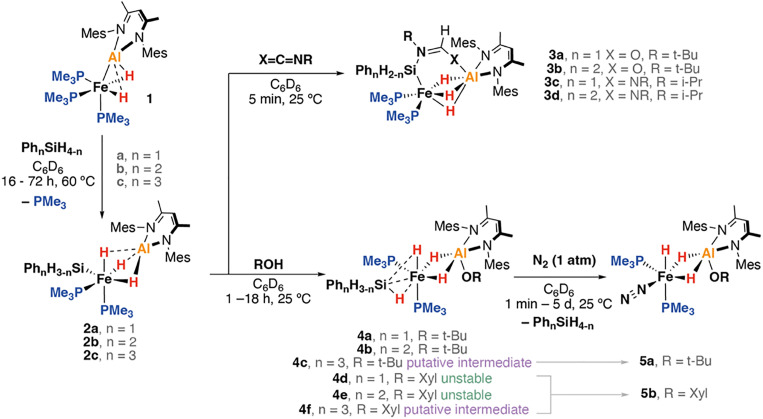
Reaction of 1 to form heterometallic silanide complexes 2a–c along, onwards insertion and protonation reactions. Mes = 2,4,6-trimethylphenyl, Xyl = 2,6-dimethylphenyl.

The ^1^H NMR spectra of 2a featured two distinct metal hydride environments at *δ*_H_ = −12.08 ppm (1H) and *δ*_H_ = −15.94 ppm (2H). The silicon hydride resonance was found at *δ*_H_ = 4.93 ppm (2H, t, ^3^*J*_P–H_ = 6.3 Hz). The ^31^P{^1^H} NMR spectra of 2a showed a sharp singlet resonance at *δ*_P_ = 32.5 ppm. The ^29^Si{^1^H} spectra featured a triplet resonance at *δ*_Si_ = 8.6 ppm (t,^2^*J*_P–Si_ = 43.4 Hz). 2a demonstrates a diagnostic ν(Si–H) stretch at 1944 cm^−1^ in the infrared spectrum. This stretch is found at ν(Si–H) = 1940 cm^−1^ in 2b and was not observed for the triphenylsilanide analogue 2c. X-ray crystallography ultimately confirmed the structure of 2a which features both a short Fe–Al interaction of 2.197(1) Å and an Fe–Si bond of 2.242(1) Å (Fig. 2a). The latter distance is at the shorter end of unsupported Fe–Si bonds and is comparable, for example, to the distance of 2.2689(6) Å found in a monomeric iron silanide complex supported by a pincer ligand.^21^

**Fig. 2:**
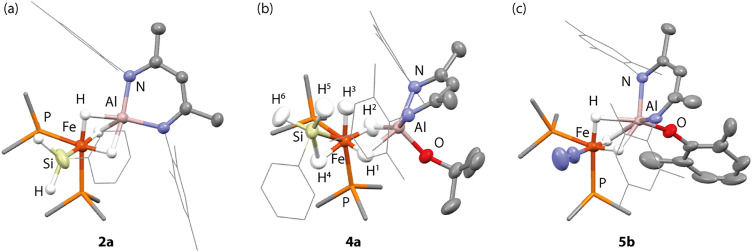
X-ray structures of (a) 2a, (b) HAR structure of 4a, and (c) one of the two molecules within the asymmetric unit of 5b. Hydrogens other than hydrides are hidden for clarity. See Tables S2 and S3 for important geometrical parameters.

Protonation of 2a–b with *t*-BuOH in benzene-*d*_6_ at 25 °C led to formation of the corresponding heterometallic σ-silane complexes 4a–b, while protonation of 2c yielded the dinitrogen complex 5a. Similar reactions of 2a–c with XylOH (Xyl = 2,6-dimethylphenyl) led to the formation of the dinitrogen complex 5b, however, in several cases, the σ-silane complexes could be spectroscopically observed as intermediates. 5b demonstrated a characteristic ν(N_2_) stretch at 2091 cm^−1^ by IR spectroscopy and the structure was confirmed through X-ray crystallography (Fig. 2c). We suggest that the ease of formation of the dinitrogen complexes reflects the steric environment around the metals, with the bulkier triphenylsilane and 2,6-dimethyphenyl ligands promoting dissociation of the σ-silane ligand.

The formation of the σ-silane complexes is enabled by the synergetic action of both metals of the heterometallic complex. Specifically, the Fe site supports the silanide ligand and renders it basic enough to deprotonate the alcohol. The Al site sequesters the resulting alkoxide, enabling formation of a stable σ-complex. Related protonation process are known for σ-alkane complexes and are reversible.^22^4a was characterised by a broad singlet resonance in the ^1^H NMR spectrum at *δ*_H_ = −13.96 ppm (4H) along with a triplet at *δ*_H_ = 4.77 ppm (2H, t, ^3^*J*_P–H_ = 6.6 Hz). These resonances are assigned to the bridging hydride ligands and terminal silicon hydrogen atoms respectively, the latter coupling to the two equivalent trimethylphosphine ligands. The implication is that the Fe–(*μ*-***H̲***)–Al and Fe–(*μ*-***H̲***)–Si environments are equivalent on the NMR timescale and must be in rapid chemical exchange. Variable temperature NMR across the 183–298 K range in toluene-*d*_8_ revealed a broadening of these resonances but no further decoalescence suggesting that the dynamic process responsible for exchange is rapid even at low temperatures. The ^31^P{^1^H} spectra featured a singlet resonance at *δ*_P_ = 27.4 ppm, with the ^29^Si{^1^H} spectrum also showing a singlet resonance at *δ*_Si_ = −27.3 ppm, with no apparent coupling to the phosphine ligands. FT-IR spectra of 4a showed ν(Si–H) stretches at 2104 and 2025 cm^−1^, blue shifted in comparison 4a, as is expected due to the conversion of the silanide to a σ-silane complex. The σ-silane ligand of 4a appears to be strongly bound to the Fe centre as reaction with a further equivalent of PMe_3_ was not facile, occurring only after 18 h at 100 °C. In contrast, the dinitrogen complexes 5a–b react with PMe_3_ below 60 °C.

Single crystals of 4a suitable for X-ray crystallography could be grown from *n*-pentane in 41% isolated yield. The solid-state data support its formulation as a heterometallic σ-silane complex with approximate octahedral and square pyramidal (*τ*_5_ = 0.03) geometries at Fe and Al respectively (Fig. 2b). The σ-silane ligand occupies one site at Fe, coordinating primarily in an η^2^-fashion. 4a features a long Fe–Al distance of 2.4251(4) Å. The Fe–Si distance of 4a is 2.2812(4) Å, elongated in comparison to the genuine Fe–Si σ-bond of 2a but comparable to a previously described phenylsilane η^2^-σ-complex of Fe bearing a pincer ligand, 2.293(1) Å.^23^

The nature the hydride ligands was further probed by Hirshfeld Atom Refinement (HAR) quantum crystallographic studies on the X-ray structure of 4a, performed using the NoSpherA2 method.^24^ As expected, H^1^ and H^2^ bridge across the Fe and Al atoms. H^3^ was essentially terminal but clearly deviated towards the Si rather than the Al atom. H^4^ bridged the Fe–Si interaction, while H^5^ and H^6^ are terminal and located on Si.

Natural Bond Orbital (NBO) analysis suggests that both silanide and σ-silane analogues involve a large component of ionic bonding (Fig. 3, left). The Al atom takes on a high positive NPA charge (1: +1.14; 2a: +1.62; 4a: +1.88), while the Fe atom is evenly negative (1: −1.10; 2a: −1.10; 4a: −1.17) across the series. The Si atom also showed small changes in charge compared to the free silane (PhSiH_3_: +0.84; 2a: +0.74; 4a: +0.93). The Fe–Si Wiberg Bond Index (WBI) of the Fe–Si bond of the silanide complex 2a is 0.50, while that of the Fe–Al interaction is 0.18. The hydride ligands of 2a are all located between Fe and Al and show aspects of unsymmetrical coordination with the dominant interaction being across the Fe–H^1^–Al bond. In the broadest sense, these can all be described by 3-centre, 2-electron bonding. The bonding picture is different for the σ-silane complex 4a. Two of the hydrides, H^1^ and H^2^ bridge Fe and Al sites with similar NPA charges and Fe–H, Al–H and Fe–Al WBIs to those observed in 2a. The remaining hydride, H^3^, sits in an axial coordination site on Fe with a Fe–H^3^ WBI of 0.36. This hydride cants toward the Si atom of the σ-silane ligand, with the Si–H^3^ WBI of 0.26 suggestive of a bonding interaction. This interaction appears to be of a similar magnitude to that of the Fe–H^4^ and Fe–Si bonds based on the comparison to the WBIs values of 0.30 and 0.26 respectively.

Quantum Treatment of Atoms In Molecules (QTAIM) analysis describes a similar bonding scenario (Fig. 3, right). 2a featured a bond critical point (BCP) between Fe and Al with a small *ρ*(*r*) = 0.06 and positive ∇^2^*ρ*(*r*) = 0.14 suggestive of a weak and polar interaction between the metals. H^1^ is bonded to Fe and Al as evidenced BCP between this hydride and both metals. H^2^ and H^3^ only exhibited bond paths to the Fe, but not the Al atom. For comparison, 4a did not show a BCP between Fe and Al, with the key interactions between the metals being through the two bridging hydride ligands H^1^ and H^2^. The interactions associated with the σ-silane ligands are clearly apparent however, with BCPs between Fe, Si and H^4^. No BCP was found between the Si and the H^3^ nuclei, though an accumulation of charge density in the interatomic region was observed.

**Fig. 3:**
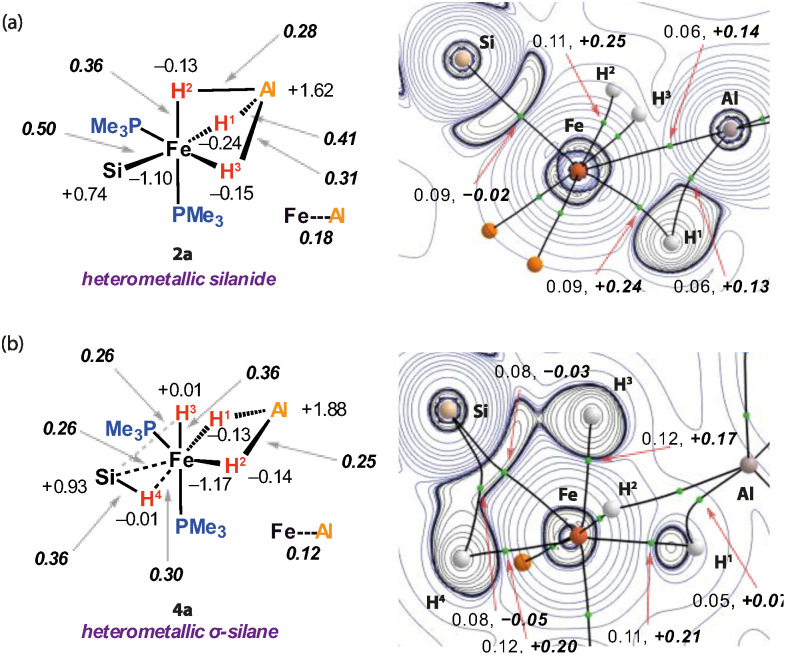
Bonding analysis on (a) silanide (2a) and (b) σ-silane (4a) complexes. Left: NBO analysis of 2a and 4a including NPA charges and ***WBIs*** (in bold italics). Right: QTAIM analysis of 2a and 4a with the contour plot showing the Laplacian of the electron density. Bond paths are represented by solid lines, bond critical points by green dots. The numbers are the electron density (*ρ*(*r*)) and the ***Laplacian of the electron density*** (∇^2^*ρ*(*r*), in bold italics) at the BCPs.

The orbital contributions to 4a were further considered through IBO analysis (Table S7). After localisation of the molecular orbitals, nine occupied valence IBOs with significant contributions from the Fe atom could be identified. Two of these are responsible for the σ-type interactions with the trimethylphosphine ligands and a further two are essentially non-bonding Fe d orbitals. Two more orbitals are account for the Fe–Al bonding through the bridging hydrides H^1^ and H^2^. The last three orbitals of interest are responsible for the Fe–Si bonding; two of these orbitals are once again hydride centred, the third is located primarily of Fe 3d character, with a small contribution from the H and Si centres.

Further experiments were carried out to better understand the protonation event. A competition experiment between 2a and a mixture of excess *t*-BuOH and XylOH yielded exclusive formation 4a in >95% NMR yield (Scheme 2). No formation of 4d was observed even after extended reaction times at room temperature. By DFT calculations^25^4d is predicted to be 2.0 kcal mol^−1^ more stable than 4a, hence the reaction is under kinetic, and not thermodynamic control. Additionally, 2a was reacted with a mixture of KOt-Bu and 18-crown-6 followed by protonation using XylOH. This resulted in the formation of 4a in 85% yield, with no 4d being detected. Attempted protonation of 2a using Brookhart's acid [H(OEt_2_)_2_][BAr_4_] (Ar = 3,5-(CF_3_)_2_C_6_H_3_) followed by addition of a mixture of KOt-Bu and 18-crown-6 yielded an intractable mixture, with no characterised heterometallic complexes detectable. These experiments suggest that the protonation step may be controlled by an initial non-reversible coordination of the alcohol to the Al site of 2a as the less Brønsted acidic, more Lewis-basic protonation reagent reacts preferentially and more selectively.

**Scheme 2:**
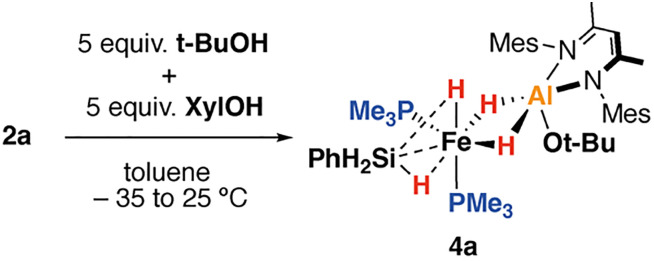
Competition reaction between 2a and *t*-BuOH and XylOH.

In summary, a new strategy is presented to access σ-silane complexes through cooperative action of two metals in an Fe–Al heterometallic complex. Protonation of an iron silanide moiety with *t*-BuOH or XylOH occurs to selectively generate a σ-silane ligand. Such reactivity is only possible due to the close proximity of the aluminium site, which sequesters the alkoxide group, preventing its direct coordination to iron. Our findings suggest that heterometallic complexes are viable precursors to σ-complexes through a protonation approach, and that judicious choice of metals allows generation of target σ-complexes even in the absence of weakly coordinating anions or strong acids.

We thank the European Research Council for funding (101001071). Mr Peter Haycock and Dr Stuart Elliott are thanked for assistance with NMR experiments. Dr Andrew J. P. White is thanked for useful discussions about quantum crystallography. Dr Sara Belazregue is thanked for the donation of Brookhart's acid. The computational results were made possible by the Imperial College Research Computing Service.^26^

## Conflicts of interest

There are no conflicts of interests to declare.

## Supplementary Material

CC-062-D5CC06456B-s001

CC-062-D5CC06456B-s002

CC-062-D5CC06456B-s003

## Data Availability

The data that support the findings of this study are available in the supplementary information (SI): experimental procedures, characterisation data, summary of crystal data, crystal structures for compounds **3b**, **3c** and **S4**, and details of quantum chemical calculations (PDF); computational coordinates (xyz), and crystallographic data (cif). See DOI: https://doi.org/10.1039/d5cc06456b. Raw NMR and computational data are available at the following repository: https://data.hpc.imperial.ac.uk/resolve/?doi=15531. CCDC 2482251–2482257 contain the supplementary crystallographic data for this paper.^27*a*–*g*^
