# Supplementary material for: A heterometallic σ-silane adduct from cooperative reactivity of an iron–aluminium complex
Source: Chem Commun (Camb). 2025 Dec 1;62(2):572–5. doi: 10.1039/d5cc06456b (PMC12679555; doi:10.1039/d5cc06456b)
Supplement: CC-062-D5CC06456B-s002 [file CC-062-D5CC06456B-s002.pdf]

Supplementary Information (SI) for ChemComm.
This journal is © The Royal Society of Chemistry 2025

Electronic Supporting Information for: A Heterometallic σ-Silane Adduct from Cooperative Reactivity of an Iron–Aluminium Complex

Benedek Stadler[a] and Mark R. Crimmin[a*]

* Corresponding author email: m.crimmin@imperial.ac.uk

[a] Department of Chemistry, Imperial College London, Molecular Sciences Research Hub, 82 Wood Lane, Shepherds Bush, London, W12 0BZ, United Kingdom

S1. Experimental section 2
S1.1. General experimental 2
S1.2. Analytical methods 2
S1.3. Reagents 3
S1.4. Synthesis and characterisation of new compounds 4
S1.5. Mechanistic probe experiments 26
S2. X-ray crystallography 30
S2.1. Summary of X-ray data 30
S2.2. Refinement details 33
S2.3. Summary of bond lengths and angles 41
S3. Computational section 42
S3.1. General computational 42
S3.2. Computational benchmarking 44
S3.3. Energies of all stationary points 47
S3.4. Wavefunction analysis 48
S4. NMR and IR spectra 58
S5. References 120

S1. Experimental section

S1.1. General experimental

Unless otherwise specified, all manipulations were carried out using standard Schlenk-line and glovebox techniques under an inert atmosphere of dinitrogen or argon. An MBraun Labmaster glovebox was employed, operating at <0.1 ppm O_2 and <0.1 ppm H_2O. Glassware was dried for >12 h at >120 °C prior to use. Most anhydrous solvents were dried over activated alumina from a solvent purification system (SPS) based upon the Grubbs design under N_2. Reagents were stored at room temperature and not shielded from light unless otherwise specified.

S1.2. Analytical methods

NMR Spectra were recorded on Bruker 400 MHz or 500 MHz at 298 K unless otherwise stated and values recorded in ppm. Data were processed in MestReNova software. Where needed, chemical shifts were assigned with the assistance of 2D NMR (HSQC, HMBC, COSY, NOSEY) spectra. "NMR yield" refers to $^{31}P\{^1H\}$ NMR measurements against a triphenylphosphine in C_6D_6 capillary internal standard or ^1H NMR measurements against a 1,3,5-trimethoxybenzene in C_6D_6 capillary internal standard, unless otherwise specified. Spectra were referenced to solvent residual signals.[1]

FT-IR spectra were recorded on an Agilent Cary630 ATR FT-IR spectrometer located inside a glovebox. IR υ values are reported in cm^{-1}.

X-ray crystallographic data were collected using an Agilent Xcalibur 3 E or an Xcalibur PX Ultra A diffractometer, using Mo-Kα or Cu-Kα radiation, respectively.

Elemental analyses were performed by London Metropolitan University (https://www.londonmet.ac.uk/).

S1.3. Reagents

- **1**: Prepared according to literature procedure in reference [2] as published by our group.
- **S1**: Prepared according to literature procedure in reference [3] as published by our group.
- $[H(OEt_2)_2][BAr_4]$ (Ar = 3,5-$(CF_3)_2C_6H_3$): Prepared according to literature procedure in reference.[4]
- Ph_3SiH, ^{i}PrNCN^{i}Pr, PMe_3, 18-crown-6, KO^{t}Bu: Purchased anhydrous from Sigma Aldrich, stored under N_2, and used without further purification.
- $PhSiH_3$, Ph_2SiH_2: Purchased anhydrous from Sigma Aldrich, stored under N_2 over 3 Å molecular sieves.
- ^{t}BuOH: Purchased from Sigma Aldrich, dried over 3 Å molecular sieves and degassed using 3 freeze-pump-thaw cycles, stored over fresh 3 Å molecular sieves under N_2 at −35 °C.
- 2,6-dimethylphenol (XylOH): Purchased from Sigma Aldrich, twice recrystallised from dry toluene then vacuum dried; stored under N_2.
- ^{t}BuNCO: Purchased from Sigma Aldrich, distilled from CaH_2 under reduced pressure and degassed using 3 freeze-pump-thaw cycles, then filtered through activated neutral alumina; stored over 3 Å molecular sieves under N_2.
- Toluene, *n*-hexane, *n*-pentane, THF, Et_2O: dried and degassed using an SPS system based on the Grubbs design; stored over 3 Å molecular sieves under N_2.
- C_6D_6, toluene-$[D_8]$ C_6H_6, methylcyclohexane, *n*-heptane, HMDSO, TMS: Dried over 3 Å molecular sieves and degassed using 3 freeze-pump-thaw cycles; stored over fresh 3 Å molecular sieves under N_2.

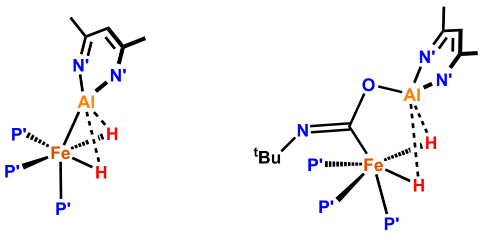

1 **S1**

N' = NMes
P' = PMe_3

Figure S1 Line drawing of compounds **1** and **S1**.

S1.4. Synthesis and characterisation of new compounds

S1.4.1. Synthesis of **2a**

S1.4.1.1.1. Procedure

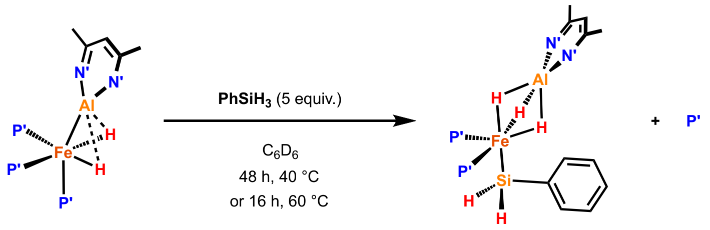

1 2a

N' = NMes
P' = PMe$_3$

To solution of **1** (300 mg, 0.464 mmol, 1 equiv.) in toluene (ca. 4 mL) was added phenylsilane (290 µL, 2.32 mmol, 5 equiv.) and the mixture was heated for 18 hours at 60 °C, or alternatively 2 days at 40 °C. By this time full consumption of **1** and concomitant formation of **2a** in 85 % NMR yield had taken place. The volatiles were removed under vacuum at 80 °C yielding an orange-red residue which was triturated with *n*-pentane (3 x 1 mL). The solids were extracted with Et_2O (ca. 10 mL total), the extracts were filtered and the volatiles removed under vacuum. **2a** could be crystallised in two crops in a mixture of Et_2O:methylcyclohexane (ca. 10 mL total) at -35 °C. The crystals were washed with cold (-35 °C) *n*-pentane (3 x 1 mL) and dried under vacuum. Combined yield of **2a** isolated as orange crystals: 136.8 mg, 0.202 mmol, 43%. Crystals suitable for X-ray diffraction could be grown from a mixture of Et_2O:*n*-hexane at −35 °C over several days.

S1.4.1.2. Spectroscopic data

^{1}H NMR (500 MHz, C_6D_6) δ 8.13 – 8.07 (m, 2H, Ph *o*-C***H***), 7.38 – 7.31 (m, 2H, Ph *m*-C***H***), 7.26 – 7.21 (m, 1H Ph *p*-C***H***), 6.78 (s, 4H, BDI Mes C***H***), 4.93 (t, $^3J_{P\text{-}H}$ = 6.3 Hz, 2H, Si***H***), 4.91 (s, 1H, BDI C***H***), 2.16 (s, 6H, Mes C***H***$_3$), 2.12 (s, 12H, Mes C***H***$_3$), 1.30 (s, 6H, BDI C***H***$_3$), 0.97 – 0.93 (m, 18H, P(C***H***$_3$)$_3$), -12.08 (br m, 1H, Fe–µ-***H***–Al), -15.95 (br d, $^2J_{P\text{-}H}$ = 23.4 Hz, 2H, Fe–µ-***H***–Al).

^{13}C{^{1}H} NMR (126 MHz, C_6D_6) δ 171.0 (2C, BDI N***C***), 153.3 (Ph Si–***C***), 142.0 (2C, Mes-N***C***), 136.3 (2C, Ph *o*-***C***H), 136.2 (4C, Mes ***C***CH$_3$), 133.4 (2C, Mes ***C***CH$_3$), 130.2 (4C, Mes ***C***H), 126.8 (2C, Ph *m*-***C***H) 125.6 (Ph *p*-***C***H), 100.3 (BDI C***H***), 26.0 – 25.6 (m, 6C, P(***C***H$_3$)$_3$), 23.7 (2C, BDI ***C***H$_3$), 20.9 (2C, Mes ***C***H$_3$), 19.1 (4C, Mes ***C***H$_3$).

^{31}P{^{1}H} NMR (202 MHz, C_6D_6) δ 32.51 (s).

^{29}Si{^{1}H} NMR (99 MHz, C_6D_6) δ 8.55 (t, $^2J_{P\text{-}Si}$ = 43.4 Hz).

FT-IR (ATR, thin film) υ 1944 (m, Si–H), 1769 (m, M–H).

Elemental analysis calculated for $C_{35}H_{57}AlFeN_2P_2Si$: C, 61.94, H, 8.47, N 4.13; measured: C, 62.15, H, 8.09, N, 3.83.

S1.4.2. Synthesis of **2b**

S1.4.2.1. Procedure

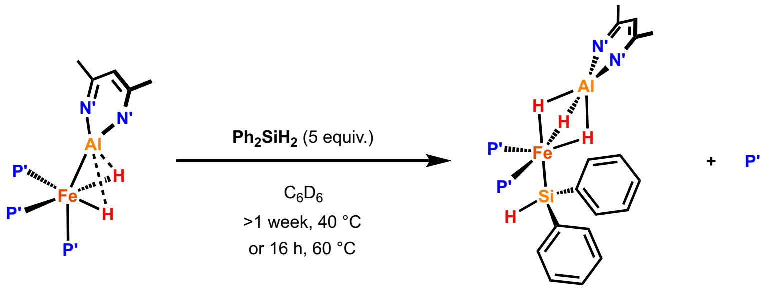

1 **2b**

N' = NMes
P' = PMe_3

To solution of **1** (60 mg, 0.0928 mmol, 1 equiv.) in toluene (ca. 1 mL) was added diphenylsilane (86 µL, 0.464 mmol, 5 equiv.) and the mixture was heated for 18 hours at 60 °C, or alternatively 1 week at 40 °C. By this time full consumption of **1** had taken place as determined by NMR spectroscopy. The volatiles were removed under vacuum at 80 °C yielding an orange-red residue which was triturated with *n*-pentane (3 x 1 mL). The solids were extracted with Et_2O (ca. 10 mL total), the extracts were filtered and the volatiles removed under vacuum. from which **2b** could be crystallised in two crops in Et_2O (ca. 7 mL) or a mixture of Et_2O:methylcyclohexane (ca. 10 mL total) at -35 °C. The crystals were washed with cold (-35 °C) *n*-pentane (3 x 1 mL) and dried under vacuum. Combined yield of **2b** isolated as orange crystals: 27.6 mg, 0.0363 mmol, 39 %.

S1.4.2.2. Spectroscopic data

^{1}H NMR (500 MHz, C_6D_6) δ 8.06 – 8.01 (m, 4H, Ph *o*-C***H***), 7.33 – 7.26 (m, 4H, Ph *m*-C***H***), 7.19 (t, $^3J_{H-H}$ = 7.3 Hz, 2H, Ph *p*-C***H***), 6.80 (s, 4H, Mes C***H***), 5.51 (t, $^3J_{P-H}$ = 7.4 Hz, 1H, Si***H***), 4.92 (s, 1H, BDI C***H***), 2.17 (s, 6H, Mes C***H***$_3$), 2.10 (s, 12H, Mes C***H***$_3$), 1.29 (s, 6H, BDI C***H***$_3$), 0.90 – 0.87 (m, 18H, P(C***H***$_3$)), -12.29 (br m, 1H, Fe–µ-***H***–Al), -15.68 – -15.97 (br m, 2H, Fe–µ-***H***–Al).

^{13}C{^{1}H} NMR (126 MHz, C_6D_6) δ 171.1 (2C, BDI N***C***), 154.5 (2C, Ph Si–***C***), 142.1 (2C, Mes-N***C***), 136.5 (4C, Ph *o*-***C***H), 136.4 (4C, Mes ***C***CH$_3$), 133.5 (2C, Mes ***C***CH$_3$), 130.4 (4C, Mes ***C***H), 126.8 (4C, Ph *m*-***C***H) , 125.6 (2C, Ph *p*-***C***H), 100.45 (BDI ***C***H), 25.9 – 25.6 (m, 6C, P(***C***H$_3$)$_3$), 23.8 (2C, BDI ***C***H$_3$), 20.9 (2C, Mes ***C***H$_3$), 19.1 (4C, Mes ***C***H$_3$).

^{31}P{^{1}H} NMR (202 MHz, C_6D_6) δ 30.53 (s).

^{29}Si{^{1}H} NMR (99 MHz, C_6D_6) δ 50.13 (t, $^2J_{P-Si}$ = 40.4 Hz).

FT-IR (ATR, thin film) υ 1940 (m, Si–H), 1746 (m, M–H).

Elemental analysis calculated for $C_{41}H_{61}AlFeN_2P_2Si$: C, 65.24, H, 8.15, N 3.71; measured: C, 65.34, H, 7.91, N, 3.52.

S1.4.3. Synthesis of **2c**

S1.4.3.1. Procedure

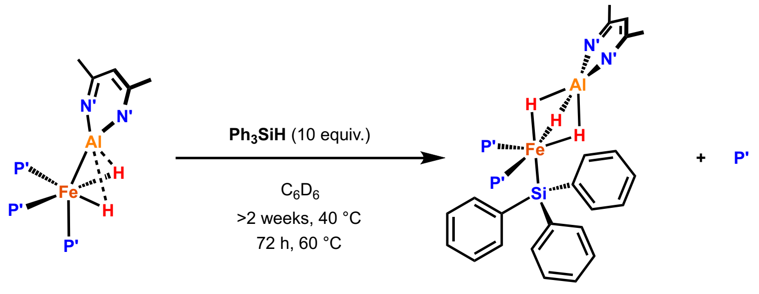

To solution of **1** (30 mg, 0.0464 mmol, 1 equiv.) in toluene (ca. 1 mL) was added triphenylsilane (120 mg, 0.464 mmol, 10 equiv.) and the mixture was heated for 72 hours at 60 °C. By this time full consumption of **1** had taken place as determined by NMR spectroscopy. The volatiles were removed under vacuum yielding an orange-red residue. This was washed with copious amounts of *n*-pentane (ca. 20 x 1 mL). The washings were initially orange, then yellow, then essentially colourless, with an orange residue remaining at the end. **2c** could then be crystallised from this residue in a mixture of toluene:*n*-hexane (approximately 2:1, ca. 2 mL total) at -35 °C over the course of several days. The crystals were washed with *n*-pentane (3 x 1 mL) and dried under vacuum. Yield: 7.0 mg, 0.00842 mmol, 18 % orange block crystals.

S1.4.3.2. Spectroscopic data

^{1}H NMR (500 MHz, C_6D_6) δ 7.95 – 7.91 (m, 6H, Ph *o*-C***H***), 7.30 – 7.26 (m, 6H, Ph *m*-C***H***), 7.23 – 7.18 (m, 3H, Ph *p*-C***H***), 6.83 (s, 4H, Mes C***H***), 4.91 (s, 1H, BDI C***H***), 2.20 (s, 6H, Mes C***H***$_3$), 2.11 (s, 12H, Mes C***H***$_3$), 1.27 (s, 6H, BDI C***H***$_3$), 0.81 – 0.76 (m, 18H, P(C***H***$_3$)), -13.34 (br t, $^{2}J_{P-H}$ = 22.6 Hz, 1H, Fe–μ-***H***–Al), -15.70 – -16.10 (br m, 2H, Fe–μ-***H***–Al).

^{13}C{^{1}H} NMR (126 MHz, C_6D_6) δ 171.1 (2C, BDI N***C***), 153.6 (3C, Ph Si–***C***), 142.1 (2C, Mes-N***C***), 137.9 (6C, Ph *o*-***C***H), 136.4 (4C, Mes ***C***CH$_3$), 133.8 (2C, Mes ***C***CH$_3$), 130.5 (4C, Mes ***C***H), 126.5 (6C, Ph *m*-***C***H), 125.8 (3C, Ph *p*-***C***H), 100.7 (BDI ***C***H), 26.4 – 26.1 (m, 6C, P(***C***H$_3$)$_3$), 23.8 (2C, BDI ***C***H$_3$), 21.0 (2C, Mes ***C***H$_3$), 19.2 (2C, Mes ***C***H$_3$).

^{31}P{^{1}H} NMR (202 MHz, C_6D_6) δ 27.15 (s).

^{29}Si{^{1}H} NMR (99 MHz, C_6D_6) δ 44.56 (t, $^{2}J_{P-Si}$ = 34.2 Hz).

FT-IR (ATR, thin film) υ 1777 (M–H). No Si–H stretch was detected.

S1.4.4. Synthesis of **3a**

S1.4.4.1. Procedure 1

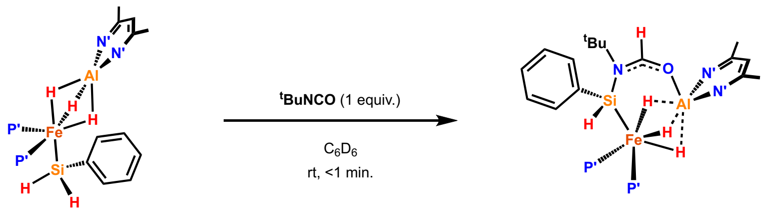

2a **N'** = NMes **P'** = PMe$_3$ **3a**

To a solution of **2a** (15 mg, 0.0221 mmol, 1 equiv.) in C_6D_6 was added ^{t}BuNCO (2.54 µL, 0.0221 mmol, 1 equiv.) and an immediate colour change from red-orange to yellow was observed. ^{31}P{^{1}H} NMR characterisation showed the complete consumption of **2a** and the formation of **3a** in >95% NMR yield. Single crystals suitable for X-ray diffraction could be grown in a low yield from a solution in *n*-pentane at −35 °C over the course of several days.

S1.4.4.2. Procedure 2

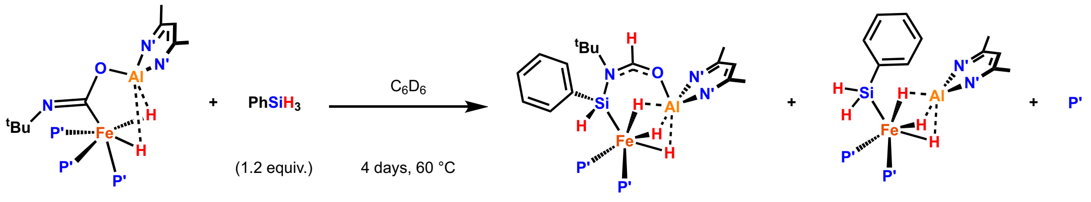

S1 **N'** = NMes **P'** = PMe$_3$ **3a** **2a**

To a solution of **S1** (30 mg, 0.0386 mmol, 1 equiv.) in C_6D_6 was added $PhSiH_3$ (5.48 µL, 0.0463 mmol, 1.2 equiv.). The mixture was heated to 60 °C for 4 days, monitored by NMR spectroscopy. At the end of the 4 days, full consumption of **S1** was observed, along with the formation of **3a** (19% NMR yield) and **2a** (15% NMR yield), free PMe_3 (ca. 20% NMR yield), as well as various other unidentified products. The presence of the other species hindered purification from this mixture.

S1.4.4.3. Spectroscopic data

^{1}H NMR (500 MHz, C_6D_6) δ 8.15 (s, 1H, NC***H***O), 7.77 – 7.71 (m, 2H, Ph *o*-C***H***), 7.31 – 7.27 (m, 2H, Ph *m*-C***H***), 7.14 – 7.09 (m, 1H, Ph *p*-C***H***), 6.94 (s, 1H, Mes C***H***), 6.85 (s, 1H, Mes C***H***), 6.82 (s, 1H, Mes C***H***), 6.78 (s, 1H, Mes C***H***), 6.67 (dt, J = 9.5, 2.4 Hz, 1H, Si***H***), 5.16 (s, 1H, BDI C***H***), 2.66 (s, 3H, Mes C***H***$_3$), 2.52 (s, 3H, Mes C***H***$_3$), 2.50 (s, 3H, Mes C***H***$_3$), 2.22 (s, 3H, Mes C***H***$_3$), 2.20 (s, 3H, Mes C***H***$_3$), 2.13 (s, 3H, Mes C***H***$_3$), 1.58 (s, 3H, BDI C***H***$_3$), 1.52 (s, 3H, BDI C***H***$_3$), 1.19 (s, 9H, NC(C***H***$_3$)$_3$), 1.03 (d, $^{2}J_{P\text{-}H}$ = 6.8 Hz, 9H, P(C***H***$_3$)), 0.54 (d, $^{2}J_{P\text{-}H}$ = 6.9 Hz, 9H, P(C***H***$_3$)$_3$), -10.80 (br, 1H, Fe–µ-***H***–Al), -16.62 (br d, $^{2}J_{P\text{-}H}$ = 33.8 Hz, 1H, Fe–µ-***H***–Al), -18.06 (br d, $^{2}J_{P\text{-}H}$ = 36.7 Hz, 1H, Fe–µ-***H***–Al).

^{13}C{^{1}H} NMR (126 MHz, C_6D_6) δ 170.3 (N***C***HO), 168.0 (BDI N***C***), 167.5 (BDI N***C***), 155.5 (Ph Si–***C***), 145.1 (Mes N***C***), 145.0 (Mes N***C***), 135.1 (Mes ***C***CH$_3$), 134.8 (Mes ***C***CH$_3$), 134.7 (Mes ***C***CH$_3$), 134.5 (Mes ***C***CH$_3$), 133.9 (Mes ***C***CH$_3$), 133.4 (Mes ***C***CH$_3$), 133.0 (2C, Ph *o*-***C***H), 129.96 (Mes ***C***H), 129.78 (Mes ***C***H), 129.71 (Mes ***C***H), 129.70 (Mes ***C***H), 127.4 (2C, Ph *m*-***C***H), 125.9 (Ph *p*-***C***H), 98.5 (BDI ***C***H), 60.1 (N***C***(CH$_3$)$_3$), 32.0 (3C, NC(***C***H$_3$)$_3$), 26.8 (dd, 3C, $^{1}J_{P\text{-}C}$ = 19.2 Hz, $^{3}J_{P\text{-}C}$ = 1.5 Hz, P(***C***H$_3$)$_3$), 26.4 (dd, 3C, $^{1}J_{P\text{-}C}$ = 19.0 Hz, $^{3}J_{P\text{-}C}$ = 2.6 Hz, P(***C***H$_3$)$_3$), 24.0 (BDI ***C***H$_3$), 23.8 (BDI ***C***H$_3$), 20.90 (Mes ***C***H$_3$), 20.86 (Mes ***C***H$_3$), 20.1 (Mes ***C***H$_3$), 20.0 (Mes ***C***H$_3$), 19.6 (Mes ***C***H$_3$), 19.4 (Mes ***C***H$_3$).

$^{31}P\{^1H\}$ NMR (202 MHz, C_6D_6) δ 32.36 (dd, $^2J_{P-P}$ = 59.2, 30.0 Hz), 30.72 (d, $^2J_{P-P}$ = 59.2 Hz).

$^{29}Si\{^1H\}$ NMR (99 MHz, C_6D_6) δ 77.09 (br).

S1.4.5. Synthesis of **3b**

S1.4.5.1. Procedure 1

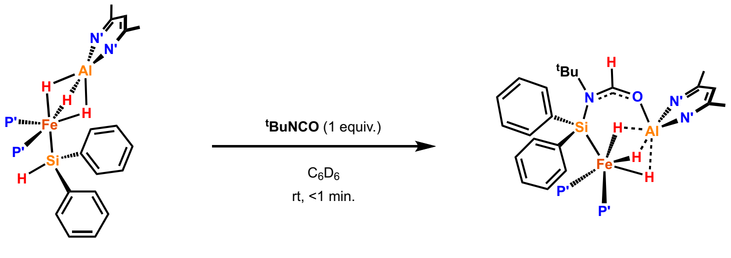

2b
N' = NMes
P' = PMe_3

3b

To a solution of **2b** (15 mg, 0.0199 mmol, 1 equiv.) in C_6D_6 was added ^{t}BuNCO (2.30 μL, 0.0199 mmol, 1 equiv.) and an immediate colour change from red-orange to yellow was observed. ^{31}P{^{1}H} NMR characterisation showed the complete consumption of **2b** and the formation of **3b** in >95% NMR yield.

S1.4.5.2. Procedure 2

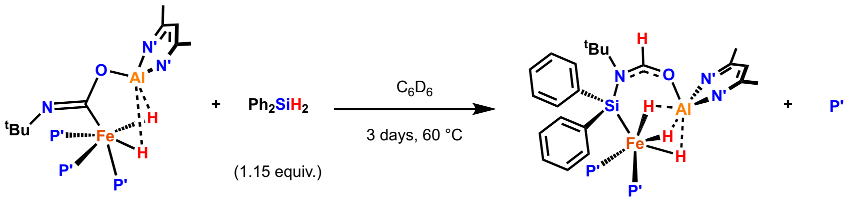

S1
N' = NMes
P' = PMe_3

3b

To a solution of **S1** (30 mg, 0.0386 mmol, 1 equiv.) in C_6D_6 was added Ph_2SiH_2 (8.36 μL, 0.0444 mmol, 1.15 equiv.). The mixture was heated to 60 °C for 3 days, monitored by NMR spectroscopy. At the end of the 3 days, full consumption of **S1** was observed, along with the formation of **3b** and free PMe_3 in >95% NMR yield. The volatiles were removed under vacuum and the red-orange residue was triturated with *n*-pentane (3 x ca. 1 mL). The solids were extracted with *n*-pentane (ca. 3 x 1 mL), the extracts filtered and concentrated to ca. 1.5 mL. **3b** could be purified by crystallisation from this solution (27.0 mg, 0.0316 mmol, 82%, orange blocky needles).

S1.4.5.3. Spectroscopic data

^{1}H NMR (500 MHz, C_6D_6) δ 8.28 (s, 1H, NC***H***O), 8.02 – 7.97 (m, 4H, Ph *o*-C***H***), 7.31 (t, $^3J_{H-H}$ = 7.5 Hz, 4H, Ph *m*-C***H***), 7.13 (dt, $^3J_{H-H}$ = 7.3, 1.3 Hz, 2H, Ph *p*-C***H***), 6.99 (s, 2H, Mes C***H***), 6.79 (s, 2H, Mes C***H***), 5.20 (s, 1H, BDI C***H***), 2.57 (br s, 6H, Mes C***H***$_3$), 2.45 (br s, 6H, Mes C***H***$_3$), 2.17 (s, 6H, Mes C***H***$_3$), 1.56 (s, 6H, BDI C***H***$_3$), 1.03 (s, 9H, NC(C***H***$_3$)$_3$), 0.54 – 0.50 (m, 18H, P(C***H***$_3$)$_3$), -11.59 (br, 1H, Fe–μ-***H***–Al), -17.38 (br m, 2H, Fe–μ-***H***–Al).

^{13}C{^{1}H} NMR (126 MHz, C_6D_6) δ 173.2 (N***C***HO), 167.7 (2C, BDI N***C***), 154.7 (2C, Ph Si–***C***), 145.1 (2C, Mes N***C***), 135.1 (2C, Mes ***C***CH$_3$), 134.8 (br s, 2C, Mes ***C***CH$_3$), 133.9 (br s, 4C, Ph *o*-***C***H), 133.5 (br s, 2C, Mes ***C***CH$_3$), 130.0 (br s, 2C, Mes ***C***H), 129.8 (br s, 2C, Mes ***C***H), 127.1 (4C, Ph *m*-***C***H), 125.7 (2C, Ph *p*-***C***H), 98.7 (BDI ***C***H), 62.3 (N***C***(CH$_3$)$_3$), 33.0 (3C, NC(***C***H$_3$)$_3$), 26.08 – 25.81 (br m, 6C, P(***C***H$_3$)$_3$)), 23.8 (2C, BDI ***C***H$_3$), 20.9 (2C, Mes ***C***H$_3$), 19.9 (br s, 2C, Mes ***C***H$_3$), 19.5 (br s, 2C, Mes ***C***H$_3$).

^{31}P{^{1}H} NMR (202 MHz, C_6D_6) δ 27.74 (s).

^{29}Si{^{1}H} NMR (99 MHz, C_6D_6) δ 93.29 (br m).

FT-IR (ATR, thin film) υ 1785 (m, M–H), 1633 (m, N=C=O).

S1.4.1. Synthesis of **S2** and **S3**

S1.4.1.1. Procedure

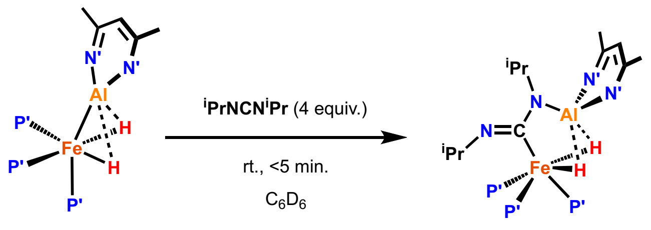

1

N' = NMes
P' = PMe$_3$

S2

To solution of **1** (30 mg, 0.0464 mmol, 1 equiv.) in C_6D_6 was added rapidly ^{i}PrNCN^{i}Pr (29 µL, 0.1086 mmol, 4 equiv.) and a colour change to yellow-orange was observed within minutes. NMR characterisation of the solution revealed the formation of **S2** in ca. 77%. **S2** is also formed when ^{i}PrNCN^{i}Pr is added slowly or in a lower excess, albeit in lower yield. **S2** could not be isolated due to its instability (see below).

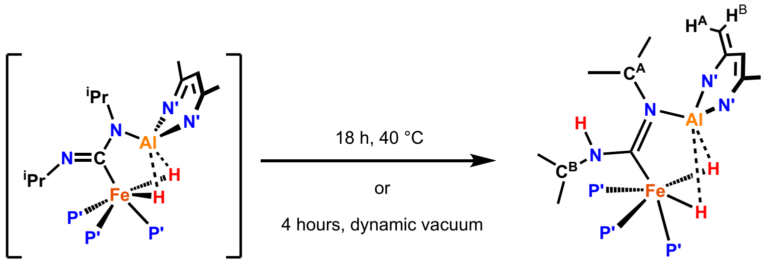

S2
impure solution, prepared *in situ*

S3
tentative assignment

S2 was found to be unstable when heated as well as under vacuum. Heating the reaction mixture described above to 40 °C for 18 hours results in a colour change to yellow along with the formation of **S3** (>95% NMR yield relative to **S2**, ca. 75% overall). The same product is formed in a lower yield when **S2** is held under dynamic vacuum (ca. 1 mbar) for 4 hours. The volatiles were removed under vacuum and the yellow residue triturated with *n*-pentane (ca. 2 x 1 mL). The solids were then extracted with *n*-pentane (ca. 2 x 1 mL), the extracts filtered, and the solution concentrated to ca. half the volume. **S3** could be purified by crystallization from this solution at −35 °C (13.3 mg, 0.0167 mmol, 36 %, pale yellow crystals).

The identity of **S3** could not be determined unambiguously, assignments are presented for the most likely structure based on our understanding.

*S1.4.1.2. Spectroscopic data (in situ) for **S2***

^{1}H NMR (400 MHz, C_6D_6) selected resonances δ 6.85 (s, 2H, Mes C***H***), 6.74 (s, 2H, Mes C***H***), 5.23 (s, 1H, MDI C***H***), 4.82 (hept, 1H, NCH(C***H***$_3$)$_2$), 4.03 (hept, 1H, NCH(C***H***$_3$)$_2$), 2.68 (s, 6H, Mes CH$_3$), 2.18 (s, 6H , Mes CH$_3$), 2.14 (s, 6H, Mes CH$_3$), 1.29 – 1.24 (m, 18H, P(C***H***$_3$)$_3$), 0.71 (d, $^{2}J_{P\text{-}H}$ = 4.9 Hz, 9H, P(C***H***$_3$)$_3$), -15.70 (s, 2H, Fe–*µ*-***H***–Al).

^{31}P{^{1}H} NMR (162 MHz, C_6D_6) δ 21.76 (d, $^{2}J_{P\text{-}P}$ = 24.6 Hz), 19.25 (t, $^{2}J_{P\text{-}P}$ = 25.5 Hz).

*S1.4.1.3. Spectroscopic data for **S3***

^{1}H NMR (400 MHz, C_6D_6) δ 6.99 (s, 1H, Mes C***H***), 6.95 (s, 1H, Mes C***H***), 6.88 (s, 1H, Mes C***H***), 6.84 (s, 1H, Mes C***H***), 5.70 (s, 1H, BDI C***H***), 4.56 (d, J = 9.6 Hz, 1H, N***H***), 4.03 (d, $^2J_{H-H}$ = 1.9 Hz, 1H, BDI C=C$\underline{\boldsymbol{H}}^{A/B}H^{B/A}$), 3.98 (d hept, $^3J_{H-H}$ = 9.8, 6.5 Hz, 1H, NC^B***H***(CH$_3$)$_2$), 3.58 (hept, $^3J_{H-H}$ = 6.4 Hz, 1H, NC^A***H***(CH$_3$)$_2$), 3.38 (d, $^2J_{H-H}$ = 1.9 Hz, 1H, BDI C=CH$^{A/B}\underline{\boldsymbol{H}}^{B/A}$), 2.81 (s, 3H, Mes CH$_3$), 2.70 (s, 3H, Mes CH$_3$), 2.68 (s, 3H, Mes CH$_3$), 2.61 (s, 3H, Mes CH$_3$), 2.34 (s, 3H, Mes CH$_3$), 2.28 (s, 3H, Mes CH$_3$), 1.75 (s, 3H, BDI CH$_3$), 1.61 (d, J = 6.3 Hz, 3H, NC^{B}H(C***H***$_3$)$_2$), 1.40 (d, J = 6.2 Hz, 3H, NC^{B}H(C***H***$_3$)$_2$), 1.00 (d, J = 6.4 Hz, 3H, NC^{A}H(C***H***$_3$)$_2$), 0.96 (d, $^2J_{P-H}$ = 6.0 Hz, 9H, overlapped P(C***H***$_3$)$_3$), 0.93 (s, 3H, overlapped NC^{A}H(C***H***$_3$)$_2$), 0.86 (d, $^2J_{P-H}$ = 5.8 Hz, 9H, P(C***H***$_3$)$_3$), 0.54 (d, $^2J_{P-H}$ = 6.0 Hz, 9H, P(C***H***$_3$)$_3$), -14.55 (br, 1H, Fe–*μ*-***H***–Al), -14.90 (br, 1H, Fe–*μ*-***H***–Al).

^{13}C{^{1}H} NMR (126 MHz, C_6D_6) δ 210.6 (N***C***N), 156.1 (BDI ***C***=CH^{A}H^B), 149.4 (Mes ***C***N), 148.7 (Mes ***C***N), 145.2 (BDI ***C***N), 138.3 (Mes ***C***CH$_3$), 138.1 (Mes ***C***CH$_3$), 137.5 (Mes ***C***CH$_3$), 131.8 (Mes ***C***CH$_3$), 131.5 (Mes ***C***CH$_3$), 129.9 (Mes ***C***H), 129.3 (Mes ***C***H), 129.0 (Mes ***C***H), 128.7 (Mes ***C***H), 105.3 (BDI ***C***H), 78.0 (BDI C=***C***H^{A}H^B), 50.1 (N***C***^{A}H(CH$_3$)$_2$), 48.7 (N***C***^{B}H(CH$_3$)$_2$), 26.2 (NC^{B}H(***C***H$_3$)$_2$), 25.8 (NC^{B}H(***C***H$_3$)$_2$), 25.5 (m, 3C, P(***C***H$_3$)$_3$), 25.2 (NC^{A}H(***C***H$_3$)$_2$), 25.1 – 24.6 (m, 6C, P(***C***H$_3$)$_3$), 24.7 (NC^{A}H(***C***H$_3$)$_2$), 24.3 (BDI ***C***H$_3$), 24.2 (Mes ***C***H$_3$), 22.4 (Mes ***C***H$_3$), 21.2 (Mes ***C***H$_3$), 21.0 (Mes ***C***H$_3$), 20.5 (Mes ***C***H$_3$), 19.9 (Mes ***C***H$_3$). One of the Mes ***C***CH$_3$ resonances was not observed due to possible overlap with a second Mes ***C***CH$_3$ resonance. We were unable to confidently assign all overlapped resonances between 26.2-24.2 ppm.

^{31}P{^{1}H} NMR (202 MHz, C_6D_6) δ 19.88 (m, overlapped), 19.31 (m, overlapped), 19.09 (m, overlapped).

FT-IR (ATR, thin film) υ 3462 (w, N–H), 1752 (m, M–H).

S1.4.2. Synthesis of **3c**

S1.4.2.1. Procedure 1

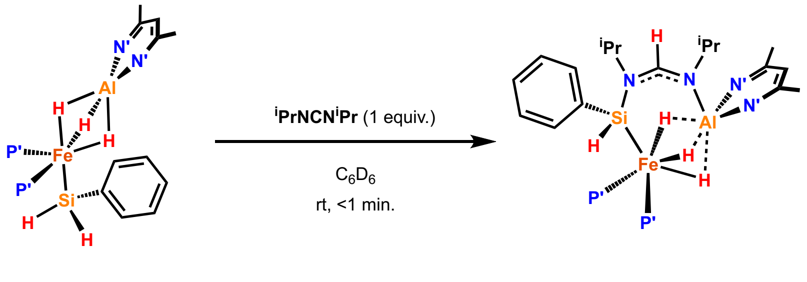

2a **3c**

N' = NMes
P' = PMe_3

To a solution of **2a** (15 mg, 0.0221 mmol, 1 equiv.) in C_6D_6 was added ^{i}PrNCN^{i}Pr (3.44 μL, 0.0221 mmol, 1 equiv.) and an immediate colour change from red-orange to yellow was observed. ^{31}P{^{1}H} NMR characterisation showed the complete consumption of **2a** and the formation of **3c** in >95% NMR yield. The volatiles were removed under vacuum, and the yellow-orange residue was triturated with *n*-pentane (ca. 3 x 1 mL). The solids were extracted with *n*-pentane (ca. 3 x 1 mL), the extracts filtered and concentrated to ca. 1 mL volume. Single crystals suitable for X-ray diffraction could be grown from a solution in *n*-pentane at -35 °C over the course of several days (9.6 mg, 0.0119 mmol, 54%, yellow-orange crystals).

S1.4.2.2. Procedure 2

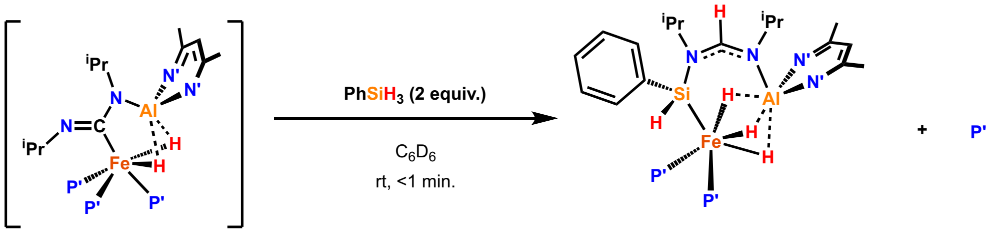

S2 **3c**

impure solution,
prepared *in situ*

To solution of **1** (30 mg, 0.0464 mmol, 1 equiv.) in C_6D_6 was added rapidly ^{i}PrNCN^{i}Pr (29 μL, 0.1086 mmol, 4 equiv.) and a colour change to yellow-orange was observed within minutes. NMR characterisation of the solution revealed the formation of **S2** in ca. 77%. To this mixture was added $PhSiH_3$ (11.6 μL, 0.0928 mmol, 2 equiv.), resulting in an immediate colour change to yellow-orange. NMR characterisation of the mixture revealed the formation of **3c** ca. 75%. The volatiles were removed under vacuum, and the yellow-orange residue was triturated with *n*-pentane (ca. 3 x 1 mL). The solids were extracted with *n*-pentane (ca. 3 x 1 mL), the extracts filtered and concentrated to ca. 1 mL volume. **3c** could be purified by crystallisation from *n*-pentane at −35 °C (5.8 mg, 0.0072, mmol, 16%, yellow-orange crystals).

S1.4.2.3. Spectroscopic data

^{1}H NMR (500 MHz, C_6D_6) δ 7.93 – 7.88 (m, 2H, Ph *o*-C***H***), 7.76 (s, 1H, NC***H***N), 7.36 (t, $^{3}J_{H\text{-}H}$ = 7.5 Hz, 2H, Ph *m*-C***H***), 7.21 – 7.17 (m, 1H, Ph *p*-C***H***), 6.88 (s, 1H, Mes C***H***), 6.83 (m, 2H, 2x overlapping Mes C***H***),6.79 (s, 1H, Mes C***H***), 6.31 (d, $^{3}J_{P\text{-}H}$ = 9.7 Hz, 1H, Si***H***), 5.01 (s, 1H, BDI C***H***), 4.15 (hept, $^{3}J_{H\text{-}H}$ = 6.7 Hz, 1H, C***H***(CH_3)$_2$), 4.04 (hept, $^{3}J_{H\text{-}H}$ = 6.5 Hz, 1H, C***H***(CH_3)$_2$), 2.60 (s, 3H, Mes C***H***$_3$), 2.52 (s, 3H, Mes C***H***$_3$), 2.48 (s, 3H, Mes C***H***$_3$), 2.32 (s, 3H, Mes C***H***$_3$), 2.21 (s, 3H, Mes C***H***$_3$), 2.15 (s, 3H,

Mes C***H***$_3$), 1.46 (s, 3H, BDI C***H***$_3$), 1.46 (s, 3H, BDI C***H***$_3$), 1.18 (overlapped d, 3H, CH(C***H***$_3$)$_2$), 1.16 (overlapped d, 3H, CH(C***H***$_3$)$_2$), 1.15 (overlapped d, 3H, CH(C***H***$_3$)$_2$), 1.06 (d, $^2J_{P\text{-}H}$ = 6.5 Hz, 9H, P(C***H***$_3$)$_3$), 0.86 (d, $^3J_{H\text{-}H}$ = 6.8 Hz, 3H, CH(C***H***$_3$)$_2$), 0.58 (d, $^2J_{P\text{-}H}$ = 6.6 Hz, 9H, P(C***H***$_3$)$_3$), -10.59 (br m, 1H, Fe–μ-***H***–Al), -16.49 (br d, $^2J_{P\text{-}H}$ = 30.7 Hz, 1H, Fe–μ-***H***–Al), -18.05 (br d, $^2J_{P\text{-}H}$ = 31.9 Hz, 1H, Fe–μ-***H***–Al).

^{13}C{^{1}H} NMR (126 MHz, C_6D_6) δ 169.3 (BDI N***C***), 169.1 (BDI N***C***), 163.7 (N***C***HN), 154.3 (Ph Si–***C***), 145.9 (Mes ***C***N), 145.8 (Mes ***C***N), 135.10 (Mes ***C***CH$_3$), 135.0 (Mes ***C***CH$_3$), 134.8 (2C, 2x overlapping Mes ***C***CH$_3$), 134.4 (Mes ***C***CH$_3$), 134.2 (Mes ***C***CH$_3$), 134.1 (2C, Ph *o*-***C***H), 130.3 (Mes ***C***H), 130.1 (2C, 2x overlapping Mes ***C***H), 130.0 (Mes ***C***H), 127.3 (2C, Ph *m*-***C***H), 126.1 (Ph *p*-***C***H), 100.1 (BDI ***C***H), 56.9 (***C***H(CH$_3$)$_2$), 50.4 (***C***H(CH$_3$)$_2$), 27.0 (d, $^1J_{P\text{-}C}$ = 18.3 Hz, 3C, P(***C***H$_3$)$_3$), 26.5 (d, $^1J_{P\text{-}C}$ = 18.6 Hz, 3C, P(***C***H$_3$)$_3$), 26.3 (BDI ***C***H$_3$), 25.1 (CH(***C***H$_3$)$_2$), 24.9 (CH(***C***H$_3$)$_2$), 24.6 (BDI ***C***H$_3$), 24.3 (CH(***C***H$_3$)$_2$), 22.5 (CH(***C***H$_3$)$_2$), 21.4 (Mes ***C***H$_3$), 20.9 (Mes ***C***H$_3$), 20.8 (2C, 2 x overlapping Mes ***C***H$_3$), 20.6 (Mes ***C***H$_3$), 20.5 (Mes ***C***H$_3$).

^{31}P{^{1}H} NMR (202 MHz, C_6D_6) δ 27.15 (s).

^{29}Si{^{1}H} NMR (99 MHz, C_6D_6) δ 86.26 (br t, $^2J_{P\text{-}Si}$ = 30.0 Hz).

FT-IR (ATR, thin film) υ 1950 (m, Si–H), 1790 (m, N=C–N), 1755 (m, M–H), 1678 (m, N=C–N)

S1.4.3. Synthesis of **3d**

S1.4.3.1. Procedure 1

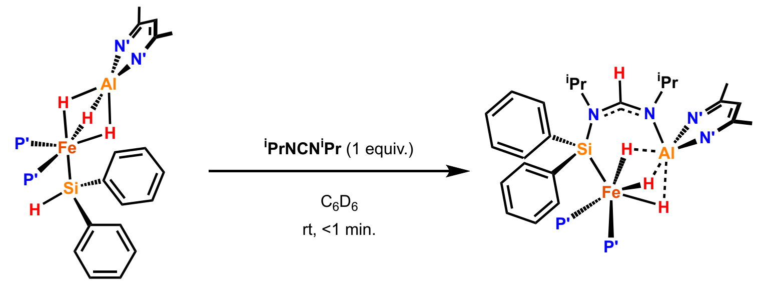

2b **3d**

N' = NMes
P' = PMe_3

To a solution of **2b** (30 mg, 0.0398 mmol, 1 equiv.) in C_6D_6 was added ^{i}PrNCN^{i}Pr (6.2 μL, 0.0398 mmol, 1.0 equiv.) and an immediate colour change from red-orange to yellow was observed. $^{31}P\{^1H\}$ NMR characterisation showed the complete consumption of **2b** and the formation of **3d** in >95% NMR yield. The volatiles were removed under vacuum and the yellow-orange residue was triturated with *n*-pentane (ca. 3 x 1 mL). The solids were extracted with Et_2O (ca. 3 x 1 mL), the extracts filtered and concentrated to ca. 2 mL volume. Analytically pure material could be obtained by crystallisation from Et_2O at −35 °C (18.9 mg, 0.0232 mmol, 58% yellow-orange powder).

S1.4.3.2. Procedure 2

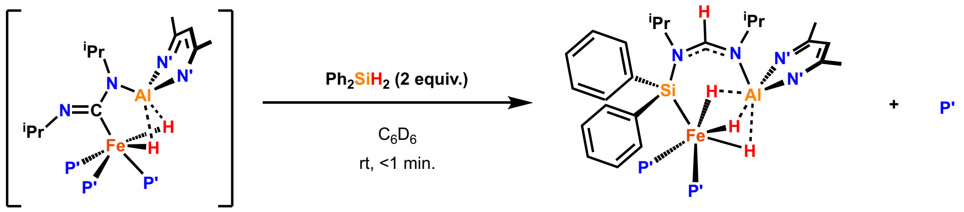

S2 **3d**

impure solution, prepared *in situ*

To solution of **1** (15 mg, 0.0232 mmol, 1 equiv.) in C_6D_6 was added rapidly ^{i}PrNCN^{i}Pr (15 μL, 0.504 mmol, 4 equiv.) and a colour change to yellow-orange was observed within several minutes. NMR characterisation of the solution revealed the formation of **S2** in ca. 77%. To this mixture was added Ph_2SiH_2 (8.6 μL, 0.0464 mmol, 2 equiv.), resulting in an immediate colour change to yellow-orange. NMR characterisation of the mixture revealed the formation of **3c** in 75% yield.

S1.4.3.3. Spectroscopic data

^{1}H NMR (500 MHz, C_6D_6) δ 8.08 (apparent d, $^3J_{H-H}$ = 7.3 Hz, 4H, Ph *o*-C***H***), 7.76 (s, 1H, NC***H***N), 7.38 (apparent t, $^3J_{H-H}$ = 7.3 Hz, 4H , Ph *m*-C***H***), 7.21 (t, $^3J_{H-H}$ = 7.3 Hz, 2H, Ph *p*-C***H***), 6.94 (s, 2H, Mes C***H***), 6.79 (s, 2H, Mes C***H***), 5.05 (s, 1H, BDI C***H***), 4.11 (hept, $^3J_{H-H}$ = 6.6 Hz, 1H, C***H***$(CH_3)_2$), 3.83 (hept, $^3J_{H-H}$ = 6.5 Hz, 1H, C***H***$(CH_3)_2$), 2.57 (s, 6H, Mes C***H***$_3$), 2.46 (s, 6H, Mes C***H***$_3$), 2.17 (s, 6H, Mes C***H***$_3$), 1.47 (s, 6H, BDI C***H***$_3$), 1.16 (d, $^3J_{H-H}$ = 6.6 Hz, 6H, CH(C***H***$_3$)$_2$), 0.71 (d, $^3J_{H-H}$ = 6.6 Hz, 6H, CH(C***H***$_3$)$_2$), 0.63 (br, 18H, P(C***H***$_3$)$_3$), -11.25 (br, 1H, Fe–μ-***H***–Al), -17.28 (br m, 2H, Fe–μ-***H***–Al).

$^{31}P\{^1H\}$ NMR (162 MHz, C_6D_6) δ 28.92 (s).

^{29}Si{^{1}H} NMR (99 MHz, C_6D_6) δ 84.5 (m, $^{2}J_{P\text{-}Si}$ = 26.0 Hz).

^{13}C{^{1}H} NMR (126 MHz, C_6D_6) δ 169.4 (BDI N***C***), 165.3 (N***C***(H)N), 151.8 (2C, Ph Si–***C***), 146.0 (2C, Mes ***C***N), 135.3 (4C, Ph *o*-***C***H), 135.2 (2C, Mes ***C***CH_3), 135.0 (2C, Mes ***C***CH_3), 134.3 (2C, Mes ***C***CH_3), 130.3 (2C, Mes ***C***H), 130.1 (2C, Mes ***C***H), 127.0 (4C, Ph *m*-***C***H), 126.0 (2C, Ph *p*-***C***H), 100.5 (BDI ***C***H), 53.9 (***C***$(CH_3)_2$), 50.9 (***C***$(CH_3)_2$), 26.4 – 26.1 (m, 6C, P(***C***$H_3)_3$), 25.8 (2C, CH(***C***$H_3)_2$), 24.51 (2C, BDI ***C***H_3), 24.47 (2C, CH(***C***$H_3)_2$), 21.4 (2C, Mes ***C***H_3), 20.8 (2C, Mes ***C***H_3), 20.5 (2C, Mes ***C***H_3).

FT-IR (ATR, thin film) υ 1761 (m, M–H), 1659 (m, N=C–N)

S1.4.4. Synthesis of **4a**

S1.4.4.1. Procedure

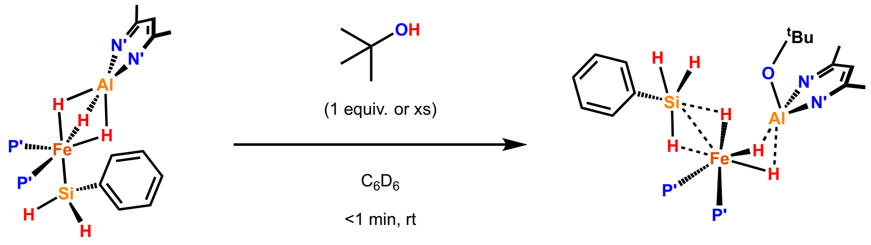

2a
N' = NMes
P' = PMe$_3$

4a

To a solution of **2a** (30 mg, 0.0442 mmol, 1 equiv.) in C_6D_6 was added ^{t}BuOH (4.2 µL, 0.0442 mmol, 1 equiv.) and an immediate colour change from red-orange to very faint yellow-brown was observed. ^{31}P{^{1}H} NMR characterisation showed the complete consumption of **2a** and the formation of **4a** in >95 % yield. The volatiles were removed under vacuum and the pale solids triturated with *n*-pentane (ca. 4 x 1 mL) yielding a tan foamy residue. This was extracted with *n*-pentane (ca. 4 x 0.5 mL), the extracts were filtered, then concentrated to ca. 0.5 mL volume. Colourless single crystals suitable for X-ray diffraction could be grown from this solution at −35 °C over the course of several days (bulk: off-white crystalline solid, 13.5 mg, 0.0179 mmol, 40%).

S1.4.4.2. Spectroscopic data

^{1}H NMR (500 MHz, C_6D_6) δ 7.74 – 7.69 (m, 2H, Ph *o*-C***H***), 7.25 (m, 2H, Ph *m*-C***H***), 7.23 – 7.17 (m, 1H, Ph *p*-C***H***), 6.73 (s, 2H, Mes C***H***), 6.69 (s, 2H, Mes C***H***), 5.23 (s, 1H, BDI C***H***), 4.77 (t, $^{3}J_{P\text{-}H}$ = 6.6 Hz, 2H, Si***H***), 2.70 (s, 6H, Mes C***H***$_3$), 2.30 (s, 6H, Mes C***H***$_3$), 2.08 (s, 6H, Mes C***H***$_3$), 1.53 (s, 6H, BDI C***H***$_3$), 1.51 (s, 9H, OC(C***H***$_3$)$_3$), 1.02 – 1.00 (m, 18H, P(C***H***$_3$)$_3$), -13.96 (br, 4H, exchanging Fe–µ-***H***–Al and Fe–µ-***H***–Si).

^{13}C{^{1}H} NMR (126 MHz, C_6D_6) δ 168.0 (2C, BDI N***C***), 147.2 (Ph Si–***C***), 144.9 (2C, Mes ***C***N), 135.4 (2C, Ph *o*-***C***H), 134.7 (2C, Mes ***C***CH$_3$), 134.6 (2C, Mes ***C***CH$_3$), 134.3 (2C, Mes ***C***CH$_3$), 129.9 (2C, Mes ***C***H), 129.8 (2C, Mes ***C***H), 127.1 (2C, Ph *m*-***C***H), 127.0 (Ph *p*-***C***H), 102.0 (BDI ***C***H), 69.4 (O***C***(CH$_3$)$_3$), 34.2 (3C, OC(***C***H$_3$)$_3$), 24.1 (2C, overlapped BDI ***C***H$_3$), 24.2 – 23.8 (overlapped dd, $^{1}J_{P\text{-}C}$ = 14.2 Hz, $^{3}J_{P\text{-}C}$ = 12.4 Hz, 6C, overlapped P(***C***H$_3$)$_3$), 23.9 (2C, Mes ***C***H$_3$), 22.6 (2C, Mes ***C***H$_3$), 20.8 (2C, Mes ***C***H$_3$), 20.2 (2C, Mes ***C***H$_3$).

^{31}P{^{1}H} NMR (202 MHz, C_6D_6) δ 27.42 (s).

^{29}Si{^{1}H} NMR (99 MHz, C_6D_6) δ 27.28 (s).

FT-IR (ATR, thin film) υ 2104 (w, Si–H), 2025 (m, Si–H), 1808 (m, M–H), 1781 (m, M–H).

Elemental analysis calculated for $C_{39}H_{67}AlFeN_2OP_2Si$: C, 62.22, H, 8.97, N 3.72; measured: C, 63.11, H, 8.49, N, 3.29.

S1.4.5. Synthesis of **4b**

S1.4.5.1. Procedure

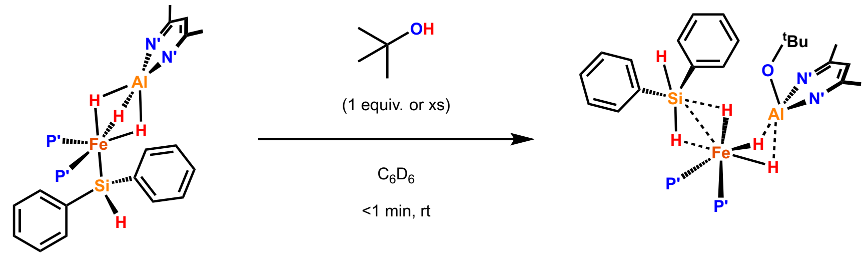

2b

N' = NMes
P' = PMe_3

4b

To a solution of **2b** (70 mg, 0.0926 mmol, 1 equiv.) in C_6D_6 was added ^{t}BuOH (8.86 µL, 0.0926 mmol, 1 equiv.) and an immediate colour change from red-orange to very faint yellow-brown was observed. ^{31}P{^{1}H} NMR characterisation showed the complete consumption of **2b** and the formation of **4b** in >95 % yield. The volatiles were removed under vacuum and the pale solids triturated with *n*-pentane (ca. 6 x 1 mL). During this process first a pale foamy residue was obtained after 3 cycles, then after 5 cycles a pale solid that has poor solubility in *n*-pentane. This was extracted with Et_2O (ca. 5 x 0.5 mL). The extracts were filtered, then concentrated to ca. 0.75 mL volume. Colourless single crystals (bulk: pale yellow solid) could be grown from this solution at −35 °C over the course of several days (45.9 mg, 0.0554 mmol, 60%).

S1.4.5.2. Spectroscopic data

^{1}H NMR (500 MHz, C_6D_6) δ 7.66 – 7.60 (m, 4H, Ph *o*-C***H***), 7.21 (m, 4H, Ph *m*-C***H***), 7.14 – 7.10 (m, 2H, Ph *p*-C***H***), 6.71 (s, 2H, Mes C***H***), 6.61 (s, 2H, Mes C***H***), 5.28 (s, 1H, BDI C***H***), 5.26 (t, $^{3}J_{P\text{-}H}$ = 4.7 Hz, 1H, Si***H***), 2.63 (s, 6H, Mes C***H***$_3$), 2.40 (s, 6H, Mes C***H***$_3$), 2.09 (s, 6H, Mes C***H***$_3$), 1.52 (s, 6H, BDI C***H***$_3$), 1.50 (s, 9H, OC(C***H***$_3$)$_3$), 0.92 – 0.87 (m, 18H, P(C***H***$_3$)$_3$), -14.11 (br, 4H, exchanging Fe–µ-***H***–Al and Fe–µ-***H***–Si).

^{13}C{^{1}H} NMR (126 MHz, C_6D_6) δ 168.3 (2C, BDI N***C***), 148.6 (2C, Ph Si–***C***), 145.3 (2C, Mes ***C***N), 136.0 (2C, Mes ***C***CH$_3$), 134.6 (2C, Mes ***C***CH$_3$), 134.4 (2C, Mes ***C***CH$_3$), 130.0 (2C, Mes ***C***H), 129.8 (2C, Mes ***C***H), 127.2 (4C, Ph *m*-***C***H), 126.9 (2C, Ph *p*-***C***H), 102.5 (BDI ***C***H), 69.6 (O***C***(CH$_3$)$_3$), 34.1 (3C, OC(***C***H$_3$)$_3$), 24.7 (dd, $^{1}J_{P\text{-}C}$ = 14.4 Hz, $^{3}J_{P\text{-}C}$ = 11.4 Hz, 6C, P(***C***H$_3$)$_3$)), 24.3 (2C, BDI ***C***H$_3$), 22.5 (2C, Mes ***C***H$_3$), 20.9 (2C, Mes ***C***H$_3$), 20.3 (2C, Mes ***C***H$_3$).

^{31}P{^{1}H} NMR (202 MHz, C_6D_6) δ 25.90 (s).

^{29}Si{^{1}H} NMR (99 MHz, C_6D_6) δ -4.21 (t, $^{2}J_{P\text{-}Si}$ = 5.4 Hz).

FT-IR (ATR, thin film) υ 2098 (w, Si–H), 1798 (m, M–H).

Elemental analysis calculated for $C_{45}H_{71}AlFeN_2OP_2Si$: C, 65.20, H, 8.63, N 3.38; measured: C, 65.37, H, 8.04, N, 3.22.

S1.4.6. Synthesis of **4c** and **5a**

S1.4.6.1. Procedure

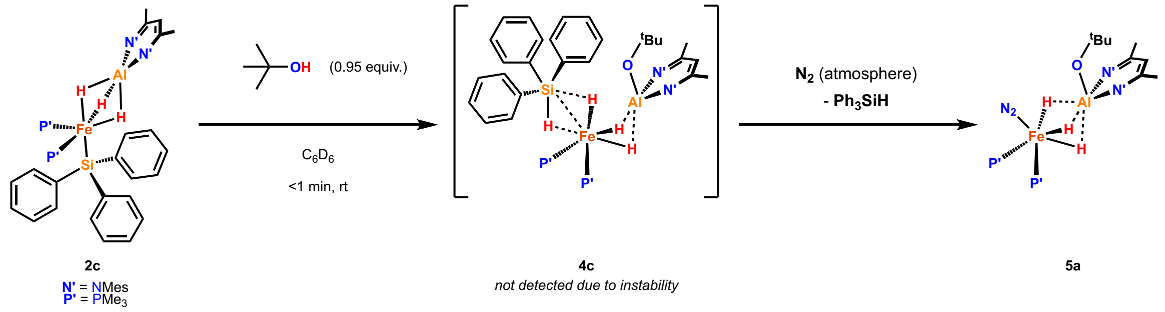

To a solution of **2c** (4 mg, 0.0048 mmol, 1 equiv.) in C_6D_6 was added ᵗBuOH (0.54 µL, 0.0056 mmol, 0.95 equiv., *via* stock solution in C_6D_6) and an immediate colour change to pale yellow was observed. NMR characterisation revealed complete consumption of the starting materials and the formation of **5a** and Ph_3SiH in 91% NMR yield. The putative intermediate **4c** could not be detected. Due to the small scale of the reaction **5a** could not be isolated and purified.

NMR characterisation if **5a** revealed that ca. 60% of the ^1H hydride resonances were exchanged to ^2H presumably due to exchange with the deuterated solvent C_6D_6. The ratio of isotopic incorporation did not change over 1 week at room temperature in C_6D_6 solution.

*S1.4.6.2. Spectroscopic data (in situ) for **5a***

^{1}H NMR (500 MHz, C_6D_6) δ 6.90 (s, 2H, Mes C***H***), 6.84 (s, 2H, Mes C***H***), 5.24 (s, 1H, BDI C***H***), 2.78 (s, 6H, Mes C***H***₃), 2.34 (s, 6H, Mes C***H***₃), 2.20 (s, 6H, Mes C***H***₃), 1.65 (s, 6H, BDI C***H***₃), 1.57 (s, 9H, C(C***H***₃)₃), 0.96 – 0.91 (m, 18H, P(C***H***₃)₃), -13.32 (s, 2H, Fe–µ-***H***–Al, 62% ^{2}H incorporation), -19.55 (s, 1H, Fe–µ-***H***–Al, 57% ^{2}H incorporation).

^{2}H NMR (77 MHz, C_6D_6) δ -13.42 (br, 2H), -19.66 (unresolved m, 1H).

^{31}P{^{1}H} NMR (202 MHz, C_6D_6) δ 26.17.

S1.4.7. Synthesis of **4d**

S1.4.7.1. Procedure

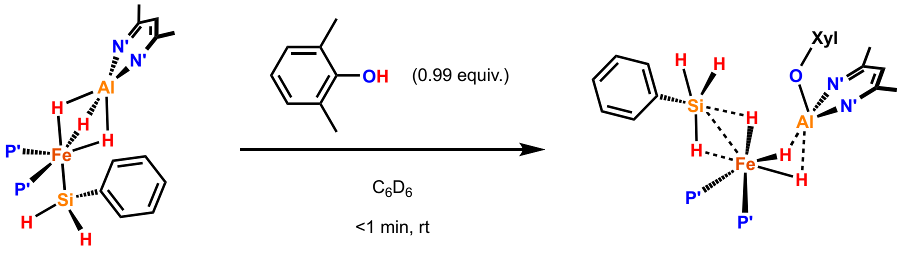

2a
N' = NMes
P' = PMe_3

4d

To a solution of **2a** (30 mg, 0.0442 mmol, 1 equiv.) in C_6D_6 was added XylOH (5.3 mg, 0.0538 mmol, 0.99 equiv., *via* stock solution in C_6D_6) and an immediate colour change from red-orange to very faint yellow-brown was observed. $^{31}P\{^1H\}$ NMR characterisation showed the complete consumption of **2a** and the formation of **4d** in 70% yield. The volatiles were removed under vacuum, the residue was triturated with *n*-pentane (2 x 1 mL), then extracted into *n*-pentane (ca. 3 x 1 mL). The extracts were filtered and concentrated to ca. 1 mL volume. **4a** could be purified by crystallisation from *this solution* at −35 °C (16.0 mg, 0.0200 mmol, 45%, off-white crystals).

4d is unstable at room temperature, forming ca. 40% **5b** as the major product overall. The use of excess XylOH was found to result in more rapid decomposition but forming a similar mixture over 24 h at room temperature. The presence of the other species prevented purification of **5b** from the mixture.

S1.4.7.2. Spectroscopic data

^{1}H NMR (500 MHz, C_6D_6) δ 7.81 – 7.77 (m, 2H, Ph *o*-C***H***), 7.23 (m 5H, overlapped Ph *m*-C***H***, and Xyl *m*-C***H***, Xyl p-**C**H), 6.88 (t, *J* = 7.3 Hz, 1H, Ph *p*-C***H***), 6.74 (s, 2H, Mes C***H***), 6.70 (s, 2H, Mes C***H***), 5.41 (t, $^3J_{P\text{-}H}$ = 7.0 Hz, 2H, Si***H***), 5.27 (s, 1H, BDI C***H***), 2.68 (s, 6H, Xyl C***H***$_3$), 2.38 (s, 6H, Mes C***H***$_3$), 2.30 (s, 6H, Mes C***H***$_3$), 2.12 (s, 6H, Mes C***H***$_3$), 1.49 (s, 6H, BDI C***H***$_3$), 0.78 – 0.72 (m, 18H, P(C***H***$_3$)$_3$), -14.05 (br m, 4H, exchanging Fe–μ-***H***–Al and Fe–μ-***H***–Si).

^{13}C{^{1}H} NMR (126 MHz, C_6D_6) δ 168.6 (2C, BDI N***C***), 157.9 (Xyl O***C***), 145.7 (Ph Si–***C***), 144.8 (2C, Mes **C**N), 135.9 (2C, Mes/Xyl **C**CH$_3$), 135.5 (2C, Mes/Xyl **C**CH$_3$), 134.9 (2C, Mes/Xyl **C**CH$_3$), 133.8 (Mes/Xyl, Ar **C**CH$_3$), 130.2 (Mes/Xyl **C**H), 129.8 (2C, Mes **C**H), 129.3 (2C, Mes **C**H), 128.6 (2C, Mes/Xyl **C**H), 117.4 (Xyl *p*-**C**H), 101.7 (BDI **C**H), 24.2 (2C, BDI **C**H$_3$), 24.1 – 23.9 (m, 6C, P(**C**H$_3$)$_3$), 20.8 (4C, overlapped Xyl **C**H$_3$, Mes **C**H$_3$), 20.20 (2C, Mes **C**H$_3$), 20.16 (2C, Mes **C**H$_3$).

^{31}P{^{1}H} NMR (202 MHz, C_6D_6) δ 23.30 (s).

^{29}Si{^{1}H} NMR (99 MHz, C_6D_6) δ -24.09 (s).

S1.4.8. Synthesis of **4e** and **5b**

S1.4.8.1. Procedure 1

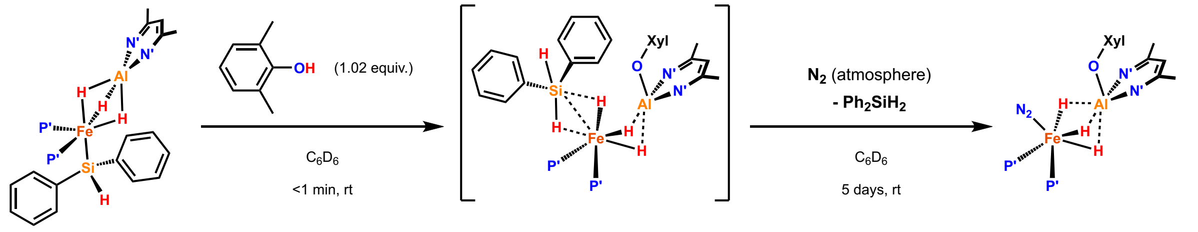

2b N' = NMes P' = PMe3 — **4e** *limited stability* — **5b**

To a solution of **2b** (30 mg, 0.0396 mmol, 1 equiv.) in C_6D_6 was added XylOH (4.7 mg, 0.040 mmol, 1.02 equiv., *via* stock solution in C_6D_6) and an immediate colour change to pale yellow-brown was observed. NMR characterisation indicated that only traces of **2b** remained, with ca. 90% conversion to **4e** and **5b**. After 4 days at room temperature the ratio of products stabilised as determined by $^{31}P\{^1H\}$ spectroscopy, with the major species in solution being **5b** (80% NMR yield) and Ph_2SiH_2 (80% NMR yield), with ca. 10% remaining **4e** remaining. The volatiles were removed under vacuum, the residue was trituated with *n*-pentane (ca. 2 x 1 mL), then the pale residue was extracted in *n*-pentane (ca. 3 x 1 mL). The extracts were filtered through glass fibre, and the volatiles once again removed under vacuum. **5b** could be purified by crystallisation from a solution in *n*-pentane (ca. 1 mL) at −35 °C (10.4 mg, 0.0143 mmol, 36%, pale yellow crystals).

4e is highly unstable at room temperature and could not be isolated. **5b** is slightly unstable at room temperature, losing ca. 10% purity over 24 h.

S1.4.8.2. Procedure 2

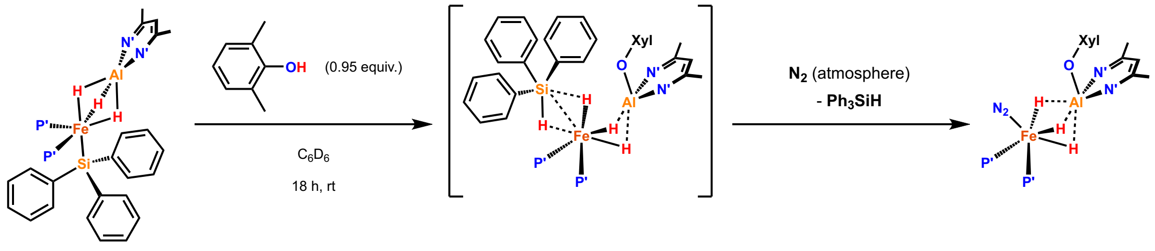

2c N' = NMes P' = PMe3 — **4f** *not detected due to instability* — **5b**

To a solution of **2c** (4 mg, 0.0048 mmol, 1 equiv.) in C_6D_6 was added XylOH (0.68 mg, 0.0056 mmol, 0.95 equiv., *via* stock solution in C_6D_6). After 18 h at room temperature the solution has turned pale yellow. NMR characterisation of the reaction mixture revealed the complete consumption of the starting materials and the formation of **5b** and Ph_3SiH in 84% yield. The putative intermediate **4f** could not be detected.

*S1.4.8.3. Spectroscopic data (in situ) of **4e***

^{1}H NMR (400 MHz, C_6D_6) δ 6.66 (s, 2H, Mes C***H***), 6.63 (s, 2H, Mes C***H***), 6.16 (t, $^3J_{P\text{-}H}$ = 8.6 Hz, 1H, Si***H***), 5.28 (s, 1H, BDI C***H***), 2.65 (s, 6H, Ar CH_3), 2.35 (s, 6H, Ar CH_3), 2.34 (s, 6H, Ar CH_3), 2.05 (s, 6H, Ar CH_3), 1.46 (s, 6H, BDI CH_3), 0.76 – 0.72 (m, 18H, P(C***H***$_3$)$_3$), -14.07 (br s, 4H, exchanging Fe–μ-***H***–Al and Fe–μ-***H***–Si).

^{31}P{^{1}H} NMR (162 MHz, C_6D_6) δ 21.21 (s).

S1.4.8.4. *Spectroscopic data for* ***5b***

^{1}H NMR (500 MHz, C_6D_6) δ 7.22 (d, *J* = 7.4 Hz, 2H, Xyl *m*-C***H***), 6.87 (t, *J* = 7.3 Hz, 1H, Xyl *p*-C***H***), 6.79 (s, 2H, Mes C***H***), 6.78 (s, 2H, Mes C***H***), 5.23 (s, 1H, BDI C***H***), 2.62 (s, 6H, Xyl C$\boldsymbol{H}_3$), 2.35 (s, 6H, Mes C$\boldsymbol{H}_3$), 2.34 (s, 6H, Mes C$\boldsymbol{H}_3$), 2.16 (s, 6H, Mes C$\boldsymbol{H}_3$), 1.56 (s, 6H, BDI C$\boldsymbol{H}_3$), 0.83 – 0.79 (m, 18H, P(C$\boldsymbol{H}_3$)$_3$), -13.58 (s, 2H, Fe–μ-***H***–Al), -18.33 (s, 1H, Fe–μ-***H***–Al).

^{13}C{^{1}H} NMR (126 MHz, C_6D_6) δ 167.8 (BDI N***C***), 157.8 (Xyl O***C***), 145.2 (2C, Mes N***C***), 135.6 (2C, Mes ***C***CH_3), 134.5 (2C, Mes ***C***CH_3), 133.6 (2C, Mes ***C***CH_3), 129.7 (4C, overlapped Mes ***C***H), 129.1 (2C, Xyl *m*-C***H***), 117.1 (Xyl *p*-C***H***), 100.7 (BDI ***C***H), 24.0 (2C, BDI ***C***H_3), 21.9 – 21.5 (m, 6C, P(***C***H_3)$_3$), 20.8 (4C, overlapped Mes ***C***H_3), 20.1 (2C, Mes ***C***H_3), 20.0 (2C, Xyl ***C***H_3). The Xyl CCH_3 resonance is possibly overlapped with the C_6D_6 residual peak.

^{31}P{^{1}H} NMR (202 MHz, C_6D_6) δ 25.32.

^{1}H-^{29}Si HSQC NMR (500 MHz, C_6D_6) no resonances were observed.

FT-IR (ATR, thin film) υ 2091 (s, N=N), 1791 (m, M–H)

S1.4.9. Synthesis of **5c**

S1.4.9.1.1. Procedure

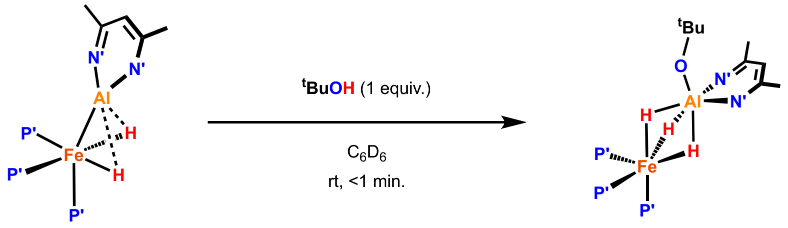

1
N' = NMes
P' = PMe_3

5c

To a solution of **1** (15 mg, 0.0464 mmol, 1 equiv.) in C_6D_6 was added ^tBuOH (4.44 µL, 0.0464 mmol, 1 equiv.) and an immediate colour change from dark red-orange to yellow was observed. NMR characterisation showed the complete consumption of the starting materials and the formation of **5c** in >95 % yield. The volatiles were removed under vacuum and the yellow residue was triturated with *n*-pentane (ca. 4 x 1 mL), yielding a foamy yellow solid. This was extracted with *n*-pentane (ca. 3 x 1 mL), the extracts were filtered, and the resulting yellow solution was concentrated to ca. 0.5 mL volume. Analytically pure samples of **5c** could be obtained by storing this solution −35 °C (yellow crystals, 9.4 mg, 0.0130 mmol, 28%).

S1.4.9.2. Spectroscopic data

^{1}H NMR (400 MHz, C_6D_6) δ 6.87 (s, 2H, Mes C***H***), 6.78 (s, 2H, Mes C***H***), 5.24 (s, 1H, BDI C***H***), 2.80 (s, 6H, Mes C***H***$_3$), 2.32 (s, 6H, Mes C***H***$_3$), 2.21 (s, 6H, Mes C***H***$_3$), 1.61 (s, 9H, OC(C***H***$_3$)$_3$), 1.52 (s, 6H, BDI C***H***$_3$), 0.97 (s, 27H, P(C***H***$_3$)$_3$), -15.85 (br s, 3H, Fe–µ-***H***–Al).

^{13}C{^{1}H} NMR (101 MHz, C_6D_6) δ 166.6 (2C, BDI N***C***), 146.8 (2C, Mes ***C***N), 136.1 (2C, Mes ***C***CH$_3$), 134.1 (2C, Mes ***C***CH$_3$), 134.0 (2C, Mes ***C***CH$_3$), 130.0 (2C, Mes ***C***H), 129.4 (2C, Mes ***C***H), 101.3 (BDI ***C***H), 68.3 (O***C***(CH$_3$)$_3$), 34.8 (3C, OC(***C***H$_3$)$_3$), 26.6 (m, 9C, P(***C***H$_3$)$_3$), 24.2 (2C, BDI ***C***H$_3$), 22.7 (2C, Mes ***C***H$_3$), 20.9 (2C, Mes ***C***H$_3$), 20.5 (2C, Mes ***C***H$_3$).

^{31}P{^{1}H} NMR (162 MHz, C_6D_6) δ 29.11 (s).

FT-IR (ATR, solid) υ 1760 (m, M–H).

S1.4.10. Synthesis of **5d**

S1.4.10.1. Procedure

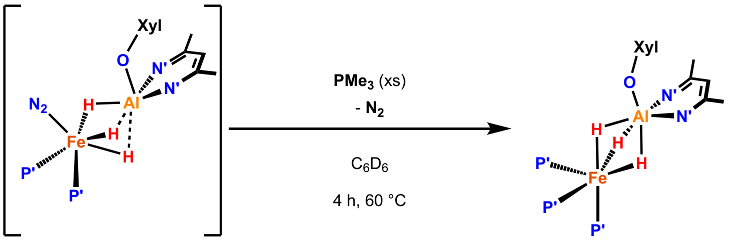

5b
prepared *in situ*
N' = NMes
P' = PMe_3

5d

5d was only ever prepared on a very small scale as part of mechanistic probe experiments and never isolated.

To solution of **5b** in a J Young NMR tube prepared *in situ* (from 4 mg of **2c** and 0.95 equiv. of XylOH) was added PMe_3 (approximately 2 equiv.). After 2 h at room temperature NMR characterisation against a PPh_3/1,3,5-trimethoxybenzene capillary showed ca. 20% conversion to **5d**. The mixture was heated to 60 °C for 4 h, after which NMR characterisation >95% conversion to **5d**.

S1.4.10.2. Spectroscopic data (in situ)

^{1}H NMR (400 MHz, C_6D_6) δ 7.36 (m, 2H, Xyl *m*-C***H***), 6.89 (t, $^{3}J_{H-H}$ = 7.3 Hz, 1H, Xyl *p*-C***H***), 6.79 (s, 2H, Mes C***H***), 6.73 (s, 2H, Mes C***H***), 5.23 (s, 1H, BDI C***H***), 2.81 (s, 3H, Xyl C***H***$_3$), 2.62 (s, 3H, Xyl C***H***$_3$), 2.46 (s, 6H, Mes C***H***$_3$), 2.35 (s, 6H, Mes C***H***$_3$), 2.18 (s, 6H, Mes C***H***$_3$), 1.47 (s, 6H, BDI C***H***$_3$), 0.89 (br m, 27H, P(C***H***$_3$)$_3$), -15.79 (br, 3H , Fe–μ-***H***–Al).

^{31}P NMR (162 MHz, C_6D_6) δ 27.27.

S1.4.11. Synthesis of **S4**

S1.4.11.1. Procedure

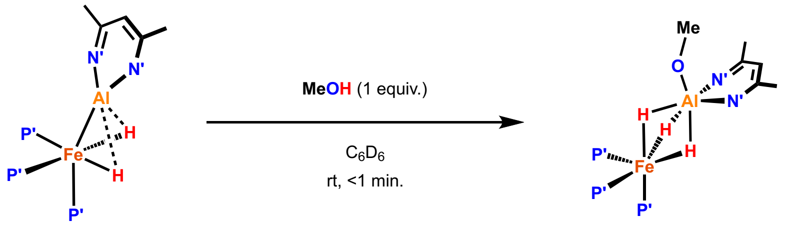

1 **S4**

N' = NMes
P' = PMe_3

To a solution of **1** (30 mg, 0.0464 mmol, 1 equiv.) in C_6D_6 was added MeOH (1.88 μL, 0.0464mmol, 1 equiv.) and an immediate colour change from dark red-orange to pale red-orange was observed. NMR characterisation showed the complete consumption of the starting materials and the formation of **S4** in >95 % yield. The volatiles were removed under vacuum and the residue was triturated with *n*-pentane (5 x 1 mL). Over the course of this process, the residue became pale and foamy, then a yellow solid with poor solubility in *n*-pentane. The solids were extracted with toluene (ca. 3 x 0.5 mL), the extracts were filtered, the yellow solution was concentrated to ca. 0.5 mL volume then layered with *n*-hexane. **S4** crystallised from this mixture as yellow crystals after several days at −35 °C (12.1 mg, 0.0178 mmol, 38%).

S1.4.11.2. Spectroscopic data

^{1}H NMR (400 MHz, C_6D_6) δ 6.88 (s, 2H, Mes C***H***), 6.78 (s, 2H, Mes C***H***), 5.10 (s, 1H, BDI C***H***), 4.02 (s, 3H, OC***H***$_3$), 2.77 (s, 6H, Mes C***H***$_3$), 2.34 (s, 6H, Mes C***H***$_3$), 2.22 (s, 6H, Mes C***H***$_3$), 1.55 (s, 6H, BDI C***H***$_3$), 0.96 (s, 27H, P(C***H***$_3$)$_3$), -15.90 (br s, 3H, Fe–μ-***H***–Al).

^{13}C{^{1}H} NMR (101 MHz, C_6D_6) δ 167.0 (2C, BDI N***C***), 146.3 (2C, Mes ***C***N), 136.0 (2C, Mes ***C***CH$_3$), 134.2 (2C, Mes ***C***CH$_3$), 133.5 (2C, Mes ***C***CH$_3$), 130.2 (2C, Mes ***C***H), 129.4 (2C, Mes ***C***H), 99.4 (BDI ***C***H), 54.0 (O***C***H$_3$), 26.4 (m, 9C, P(***C***H$_3$)$_3$), 23.8 (2C, BDI ***C***H$_3$), 21.0 (2C, Mes ***C***H$_3$), 12.0 (2C, Mes ***C***H$_3$), 18.9 (2C, Mes ***C***H$_3$).

^{31}P{^{1}H} NMR (162 MHz, C_6D_6) δ 29.69 (s).

FT-IR (ATR, solid) υ 1782 (m, M–H), 1735 (m, M–H).

S1.5. Mechanistic probe experiments

S1.5.1. The reactivity of weakly bound complexes with PMe_3

*S1.5.1.1. The reaction of **4a** with PMe_3*

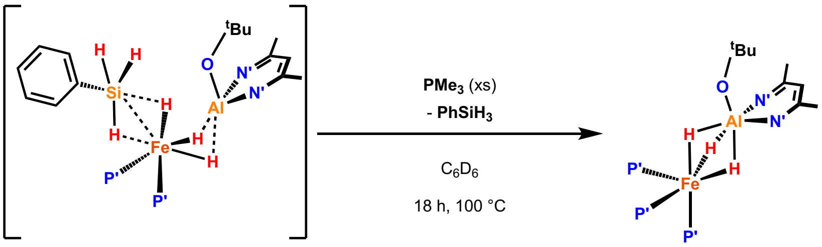

4a
prepared *in situ*
N' = NMes
P' = PMe_3

5c

To solution of **4a** in a J Young NMR tube prepared *in situ* (from **2a** (0.0221 mmol, 15 mg, 1 equiv.) and ^{t}BuOH (100 µL of a 0.221 M stock solution in C_6D_6, 0.0221 mmol, 1.0 equiv.) was added PMe_3 (ca. 20 µL, 0.2 mmol, 10 equiv.). No reaction was observed at room temperature. The mixture was heated to 80 °C for 18 h, after which NMR characterisation against a PPh_3/1,3,5-trimethoxybenzene capillary showed ca. 10 % decomposition but no product formation. The mixture was subsequently heated to 100 °C for 18 h. A colour change to pale yellow was observed and NMR characterisation showed 83% conversion to **5c**.

*S1.5.1.2. The reaction of **5a** with PMe_3*

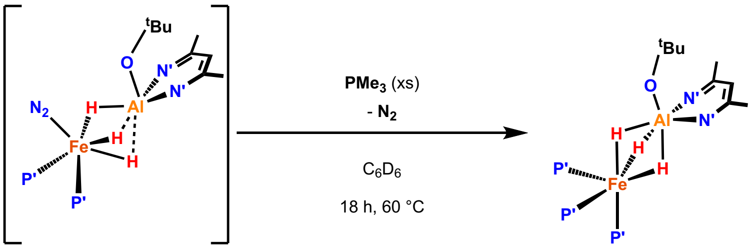

5a
prepared *in situ*
N' = NMes
P' = PMe_3

5c

To solution of **5a** in a J Young NMR tube prepared *in situ* (from **2c** (4 mg, 0.00483 mmol, 1 equiv.) and ^{t}BuOH (20.7 µL of a 0.221 M stock solution in C_6D_6, 0.00459 mmol, 0.95 equiv.)) was added PMe_3 (ca. 5 µL, 0.0486 mmol, 10 equiv.). No reaction was observed at room temperature. The mixture was heated to 60 °C for 18 h, after which NMR characterisation against a PPh_3/1,3,5-trimethoxybenzene capillary showed ca. >95% conversion to **5c**.

*S1.5.1.3. The reaction of **5b** with PMe_3*

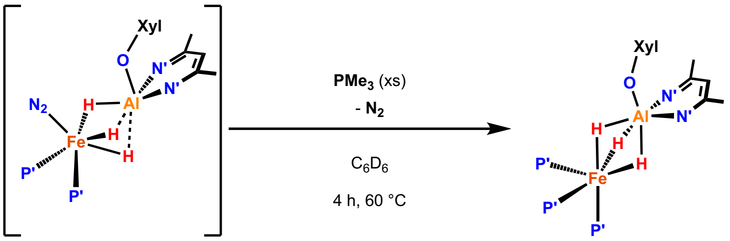

5b
prepared *in situ*
N' = NMes
P' = PMe_3

5d

To solution of **5b** in a J Young NMR tube prepared *in situ* (from **2c** (4 mg, 0.00483 mmol, 1 equiv.) and XylOH (20.7 µL of a 0.221 M stock solution in C_6D_6, 0.00459 mmol, 0.95 equiv.)) was added PMe_3 (ca. 5 µL, 0.0486 mmol, 10 equiv.). After 2 h at room temperature NMR characterisation against a PPh_3/1,3,5-trimethoxybenzene capillary showed ca. 20% conversion to **5d**. The mixture was heated to 60 °C for 4 h, after which NMR characterisation >95% conversion to **5d**.

S1.5.2. Mechanistic probes for the formation of the σ-silane complexes

S1.5.2.1. Competition reaction between ᵗBuOH and XylOH

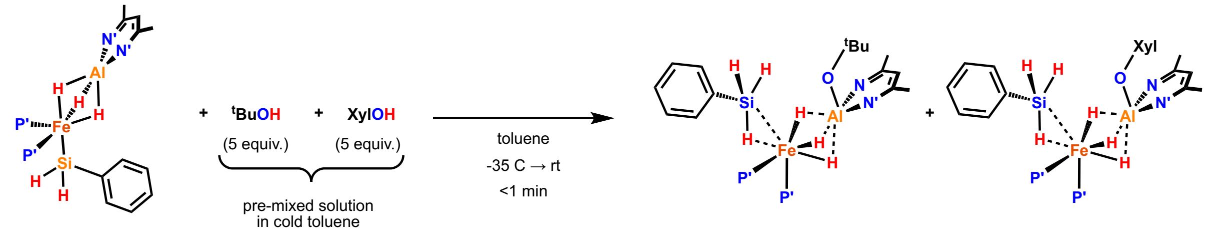

2a
solution in cold toluene
N' = NMes
P' = PMe_3

4a
>95% conv.

4d
not detected

To cooled (−35 °C) solution of **2a** (15 mg, 0.0221 mmol., 1 equiv.) in toluene in a J Young NMR tube was added *via* a cooled (−35 °C) a mixture of ᵗBuOH and XylOH was added (200 µL of a 0.555 M stock solution of both ᵗBuOH and XylOH in toluene, 0.110 mmol, 5 equiv. each). A rapid colour change to very pale yellow-brown was observed. NMR characterisation of the mixture showed >95% conversion to **4a**, **4d** could not be detected.

The volatiles were removed under vacuum and the mixture redissolved in C_6D_6, leaving a mixture of **4a** and XylOH (5 equiv.). This mixture was observed for a further 2 weeks at room temperature to ensure no further exchange occurred to form **4d** at these temperatures.

*S1.5.2.2. Crossover experiment between **4d** and ᵗBuOH*

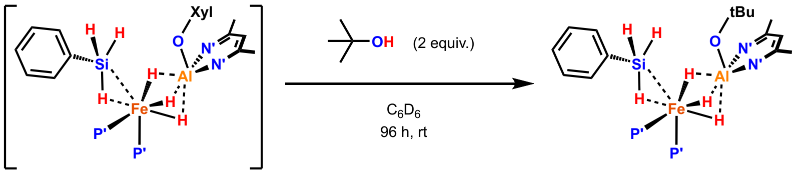

4d
prepared *in situ*
N' = NMes
P' = PMe_3

4a
not detected

To solution of **4d** in a J Young NMR tube prepared *in situ* (from **2a** (0.0221 mmol, 15 mg, 1 equiv.) and XylOH (99 µL of a 0.221 M stock solution in C_6D_6, 0.0219 mmol, 0.99 equiv.) was added ᵗBuOH

(200 µL of a 0.221 M stock solution in C_6D_6, 0.0442 mmol, 2.0 equiv.). NMR characterisation over the course of 96 h at room temperature against a PPh_3/1,3,5-trimethoxybenzene capillary showed slow decomposition with small amounts of **5b** observed, **4a** could not be detected.

S1.5.2.3. Coordination of KO^{t}Bu to the Al centre prior to protonation

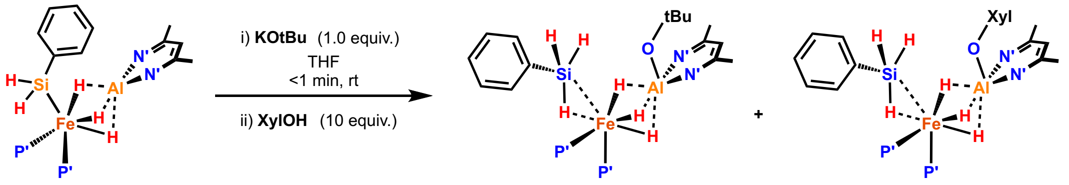

2a — N' = NMes, P' = PMe_3; 4a — 15% isol.; 4d — *not detected*

To a solution of **2a** (15 mg, 0.0221 mmol, 1 equiv.) in THF (ca. 0.3 mL)was added KO^{t}Bu (200 µL of a 0.110 M stock solution in THF). A rapid colour change to yellow was observed. To this mixture was added XylOH (27.0 mg, 0.221 mmol, 10 equiv.) in THF (ca. 0.2 mL). A minor colour change to pale yellow was observed. The volatiles were removed under vacuum, the pale residue was triturated with *n*-pentane (ca. 4 x 1 mL) and then extracted into *n*-pentane (ca. 3 x 1 mL). The filtered extracts were once again evaporated to yield a pale residue. NMR characterisation of the solid revealed 15% conversion to **4a**. **4d** could not be detected.

S1.5.2.4. Coordination of [18-crown-6-K][O^{t}Bu] to the Al centre prior to protonation

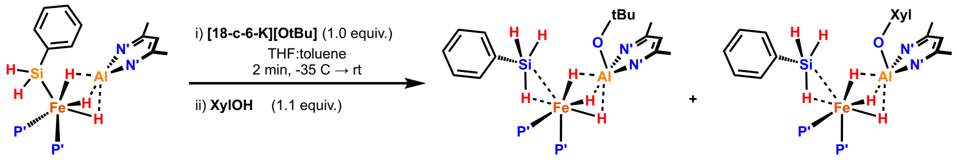

2a — N' = NMes, P' = PMe_3; 4a — 85% isol.; 4d — *not detected*

To a solution of **2a** (15 mg, 0.0221 mmol, 1 equiv.) in THF cooled to −35 °C was added a stock solution of [18-crown-6-K][O^{t}Bu] (200 µL of a 0.110 M stock in 1:1 THF:toluene, 0.0221 mmol, 1 equiv.). A rapid colour change to yellow was observed. To this mixture was added XylOH (2.96 mg, 0.0243 mg, 1.1 equiv.) in THF. A minor colour change to pale yellow-brown was observed. The volatiles were removed under vacuum, the pale residue was triturated with *n*-pentane (ca. 3 x 1 mL) and then extracted into *n*-pentane (ca. 3 x 1 mL). The filtered extracts were once again evaporated to yield a pale residue. NMR characterisation of the solid revealed 85% conversion to **4a**. **4d** could not be detected.

S1.5.2.5. Protonation with a non-coordinating acid, $[H(OEt_2)_2][BAr_4]$

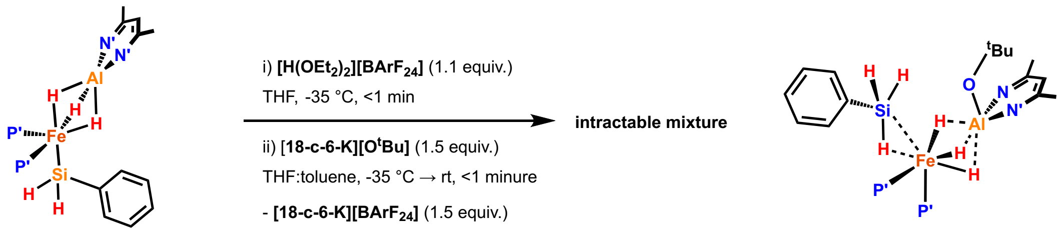

2a — 4a *desired* *not detected*

To a solution of **2a** (15 mg, 0.0221 mmol, 1 equiv.) in THF (ca. 0.3 mL) cooled to −35 °C was added a cooled (−35 °C) solution of $[H(OEt_2)_2][BAr_4]$ (24.6 mg, 0.0243 mmol, 1.1 equiv.) in THF (ca. 0.5 mL). An immediate colour change to yellow-orange was observed. After approximately 1 minute, to this mixture was added a pre-mixed stock solution of [18-crown-6-K][O^tBu] (300 µL of a 0.110 M stock in 1:1 THF:toluene, 0.0332 mmol, 1.5 equiv.) and over the next minute a colour change to green was observed, along with the formation of a light coloured solid, presumably [18-crown-6-K][BAr^F_{24}]. The volatiles were removed under vacuum, the green residue was triturated with *n*-pentane (ca. 3 x 1 mL) and then extracted into *n*-pentane (ca. 3 x 1 mL). The filtered extracts were once again evaporated to yield a pale green residue. NMR characterisation of the residue showed a complex mixture, with no characterised compounds being present.

The reaction was also performed using $[H(OEt_2)_2][BAr_4]$ (0.95 equiv.) and KO^tBu (1.1 equiv.) which also yielded an intractable mixture.

*S1.5.2.6. The protonation of **3a***

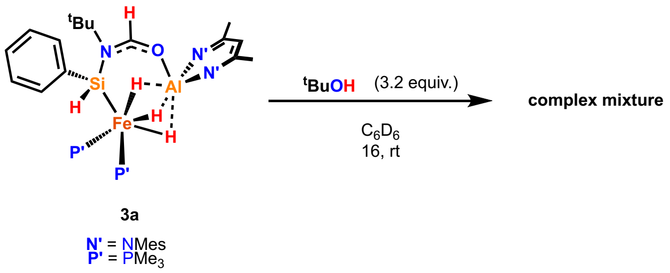

To a solution of **3a** (5 mg, 0.00643 mmol, 1 equiv.) in C_6D_6 was added ^{t}BuOH (2.0 µL, 0.0206 mmol, 3.2 equiv.). After 16 h at rt NMR characterisation revealed the formation of a complex mixture.

S1.5.3. Unsuccessful substrates

Complex **1** showed no reaction towards excess Et_3SiH, HBpin, 9-BBN and ^{t}BuNCN^{t}Bu at 60 °C. An unselective reaction was observed with excess $(Me_2SiH)_2O$ at 60 °C.

Complex **2a** did not react with excess (^{Mes}BDI)AlH_2, phenylacetylene, diphenylacetylene, ^{t}BuNCN^{t}Bu, HBpin, H_2 (ca. 4 bar), CO (ca. 4 bar), $PhSiH_3$, Ph_2SiH_2, HMDS, PMe_3, 2,6-di-tert-butyl-4-methylphenol (BHT) at >100 °C. An immediate but unselective reaction was observed between **2a** and CO_2 (1 bar) at room temperature.

Complex **2b** did not react with excess $PhSiH_3$ at 100 °C.

S2. X-ray crystallography

S2.1. Summary of X-ray data

data	2a	3b	3c
**CCDC number**	2482251	2482252	2482253
**formula**	$C_{35}H_{57}AlFeN_2P_2Si$	$C_{46}H_{70}AlFeN_3OP_2Si$, 1[$C_5H_{12}$]	$C_{42}H_{71}AlFeN_4P_2Si$, 0.25(C_5H_{12})
**formula weight (g·mol^{-1})**	678.68	926.05	822.92
**colour, habit**	orange block	orange blocky needle	orange block
**crystal size (mm)**	0.265 x 0.246 x 0.163	0.216 x 0.168 x 0.092	0.245 x 0.179 x 0.104
**crystal system**	triclinic	monoclinic	triclinic
**space group**	P-1 (no. 2)	P2$_1$/c (no. 14)	P-1 (no. 2)
***a* (Å)**	10.8871(7)	14.59351(6)	11.9256(5)
***b* (Å)**	11.7522(7)	10.02490(6)	12.2370(4)
***c* (Å)**	16.8494(9)	35.96271(15)	18.8626(8)
***α* (°)**	75.091(5)	90	85.197(3)
***β* (°)**	80.271(5)	94.4545(4)	81.930(4)
***γ* (°)**	71.666(6)	90	61.139(4)
**V (Å^3)**	1968.4(2)	5245.40(4)	2386.51(18)
**Z**	2	4	2
**T (K)**	172.95(10)	173.05(10)	173(3)
**D_c (g·cm^{-3})**	1.145	1.173	1.145
**radiation used, μ (mm^{-1})**	Cu Kα, 4.519	Cu Kα, 3.539	Cu Kα, 3.820
**F(000)**	728	2000	889
**absorption correction**	analytical	analytical	analytical
**min-max transmission**	0.416 - 0.578	0.606 - 0.791	0.583 - 0.751
**Θ range for data collection (°)**	4.061 - 73.789	3.761 - 73.638	4.126 - 73.368
**no. of unique reflns measured, obs [F > 4σ(F)]**	7517, 6001	10511, 9805	9274, 7246
**R_{int}, R_{sigma}**	0.0472, 0.0625	0.0356, 0.0201	0.0393, 0.0726
**completeness to Θ(full) (°)**	0.987 to 67.684	0.999 to 67.684	0.995 to 67.684
**no. of parameters, restraints**	431, 95	525, 0	540, 68
**R_1, wR_2 [F > 4σ(F)] [a]**	0.0536, 0.1442	0.0298, 0.0803	0.0428, 0.0965
**R_1, wR_2 [all data] [a]**	0.0669, 0.1623	0.0327, 0.0825	0.0582, 0.1087
**GooF**	1.064	1.052	1.017
**largest diff. Fourier peak, hole (eÅ^{-3})**	0.682, -0.716	0.242, -0.290	0.395, -0.449

Table S1 Summary of the crystallographic data. Data were collected using Agilent Xcalibur 3 E Agilent and Xcalibur PX Ultra A diffractometers, and the structures were solved and refined using the OLEX2[56], SHELXTL and SHELX-2018[5,7] program systems. [a] $R_1 = \Sigma||F_o| - |F_c||/\Sigma|F_o|$; $wR_2 = \{\Sigma[w(F_o^2 - F_c^2)^2] / \Sigma[w(F_o^2)^2]\}^{1/2}$; $w^{-1} = \sigma_2(F_o^2) + (aP)^2 + bP$. [b] The asymmetric unit contains two crystallographically independent molecules. [c] HAR using olex2.refine/NoSpherA2.[8]

data	4a	4a-HAR [c]	5b
**CCDC number**	2482254	2482255	2482256
**formula**	$C_{39}H_{67}AlFeN_2OP_2Si$, 0.5($C_5H_{12}$)	$C_{39}H_{67}AlFeN_2OP_2Si$, 0.5($C_5H_{12}$)	$C_{37}H_{59}AlFeN_4OP_2$
**formula weight (g·mol^{-1})**	788.87	788.915	720.65
**colour, habit**	colourless blocky needle	colourless blocky needle	pale yellow block
**crystal size (mm)**	0.19 x 0.155 x 0.117	0.19 x 0.155 x 0.117	0.468 x 0.425 x 0.24
**crystal system**	monoclinic	monoclinic	monoclinic
**space group**	$P2_1/c$ (no. 14)	$P2_1/c$ (no. 14)	$P2_1$ (no. 4)
***a* (Å)**	17.6664(2)	17.6664(2)	12.7184(3)
***b* (Å)**	11.47260(10)	11.4726(1)	17.9691(3)
***c* (Å)**	22.7577(2)	22.7577(2)	17.6796(4)
***α* (°)**	90	90	90.0
***β* (°)**	95.9620(10)	95.962(1)	90.455(2)
***γ* (°)**	90	90	90.0
**V (Å^3)**	4587.57(8)	4587.57(8)	4040.33(15)
**Z**	4	4	4[b]
**T (K)**	173(3)	173(3)	172.95(10)
**D_c (g·cm^{-3})**	1.142	1.142	1.185
**radiation used, μ (mm^{-1})**	Cu Kα, 3.957	Cu Kα, 3.956	Mo Kα, 0.518
**F(000)**	1708	1712.009	1544
**absorption correction**	analytical	analytical	analytical
**min-max transmission**	0.565 - 0.706	0.565 - 0.706	0.840 - 0.916
**Θ range for data collection (°)**	3.906 - 73.667	3.91 - 73.67	2.543 - 28.333
**no. of unique reflns measured, obs [F > 4σ(F)]**	9215, 8014	9215, 8014	17439, 15440
**R_{int}, R_{sigma}**	0.0466, 0.0269	0.0467, 0.0269	0.0325, 0.0354
**completeness to Θ(full) (°)**	1.000 to 67.684	0.9996 to 67.684	0.999 to 25.242
**no. of parameters, restraints**	553, 314	1196, 2619	886, 1
**R_1, wR_2 [F > 4σ(F)] [a]**	0.0363, 0.0954	0.0282, 0.0652	0.0388, 0.0896
**R_1, wR_2 [all data] [a]**	0.0460, 0.1009	0.0378, 0.0694	0.0494, 0.0968
**GooF**	1.056	1.0958	1.041
**largest diff. Fourier peak, hole (eÅ^{-3})**	0.257, -0.315	0.3199, -0.4290	0.361, -0.354

Table S1 continued.

data	S4
**CCDC number**	2482257
**formula**	$C_{33}H_{62}AlFeN_2OP_3$
**formula weight (g·mol^{-1})**	678.58
**colour, habit**	pale yellow tablet
**crystal size (mm)**	0.359 x 0.336 x 0.074
**crystal system**	monoclinic
**space group**	$P2_1/n$ (no. 14)
a **(Å)**	15.26490(10)
b **(Å)**	14.44850(10)
c **(Å)**	17.29140(10)
α **(°)**	90
β **(°)**	91.2590(10)
γ **(°)**	90
**V (Å^3)**	3812.78(4)
**Z**	4
**T (K)**	173.00(14)
**D_c (g·cm^{-3})**	1.182
**radiation used, μ (mm^{-1})**	Cu Kα, 4.775
**F(000)**	1464
**absorption correction**	analytical
**min-max transmission**	0.310 - 0.724
**Θ range for data collection (°)**	3.821 - 73.812
**no. of unique reflns measured, obs [F > 4σ(F)]**	7594, 6799
**R_{int}, R_{sigma}**	0.0435, 0.0263
**completeness to Θ(full) (°)**	0.998 to 67.684
**no. of parameters, restraints**	401, 0
**R_1, wR_2 [F > 4σ(F)] [a]**	0.0316, 0.0807
**R_1, wR_2 [all data] [a]**	0.0373, 0.0854
**GooF**	1.039
**largest diff. Fourier peak, hole (eÅ^{-3})**	0.258, -0.371

Table S1 continued.

S2.2. Refinement details

S2.2.1. **2a**

The P1-based PMe_3 ligand was found to be disordered. It was modelled as two orientations at a ratio of ca. 0.86:0.14. The two orientations were restrained to be similar and only the non-hydrogen atoms of the major orientation were modelled anisotropically, the rest were modelled isotropically. All three Fe–*μ*-***H***–Al hydrides were found in the Δ*F* map and freely refined. The two Si–***H*** hydrides were found in the Δ*F* map. Free refinement gave highly asymmetrical Si–H distances, so they were refined with weak similarity restrains.

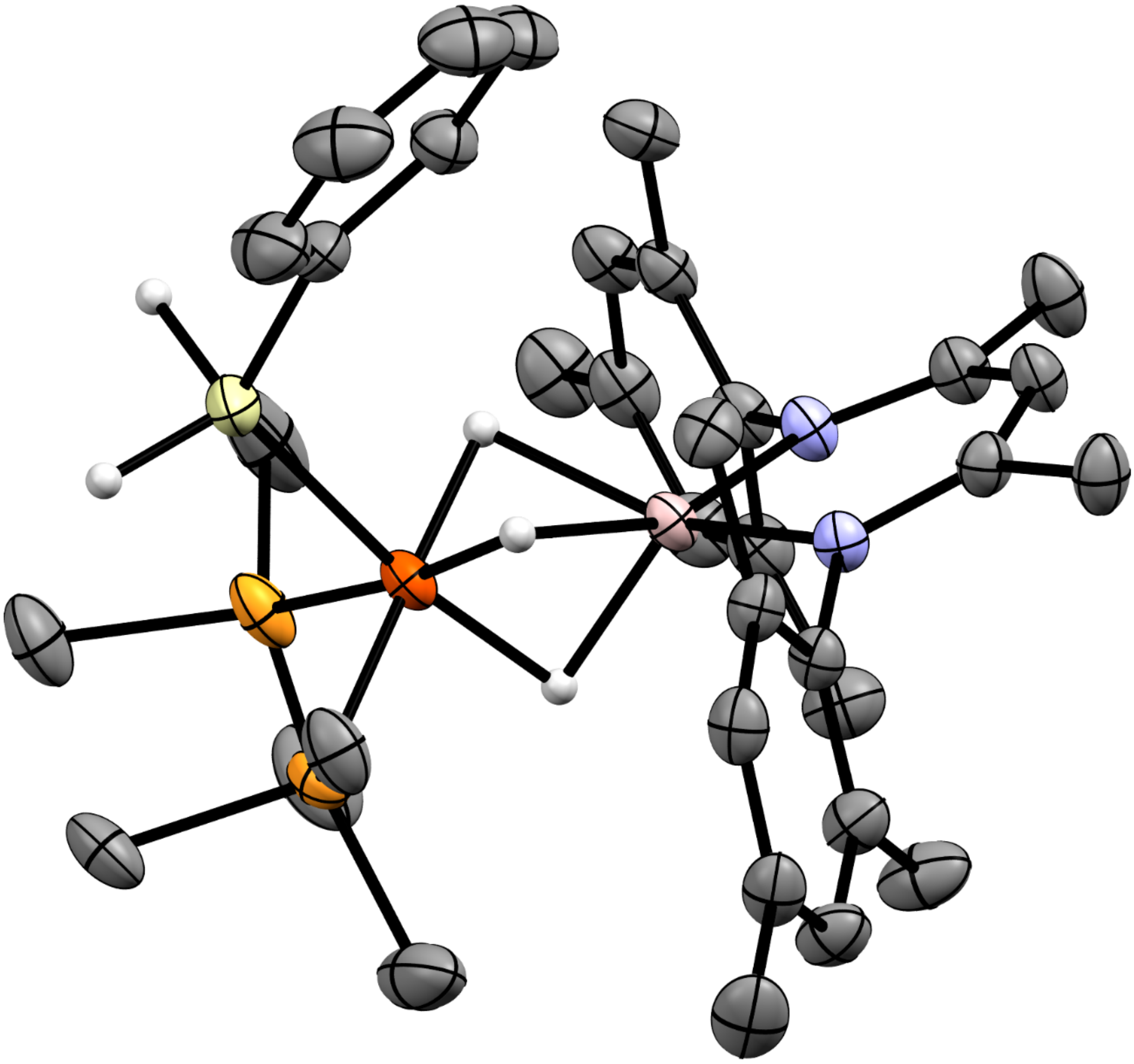

Figure S2 X-ray structure of **2a**. Most hydrogen atoms and atoms of the minor occupancy orientation are hidden for clarity.

S2.2.2. **3b**

All three Fe–*μ*-***H***–Al hydrides were found in the Δ*F* map and freely refined. The NC(***H***)O atom was found in the Δ*F* map. It was ultimately treated using the conventional riding model which put it in a very similar position to the free refinement.

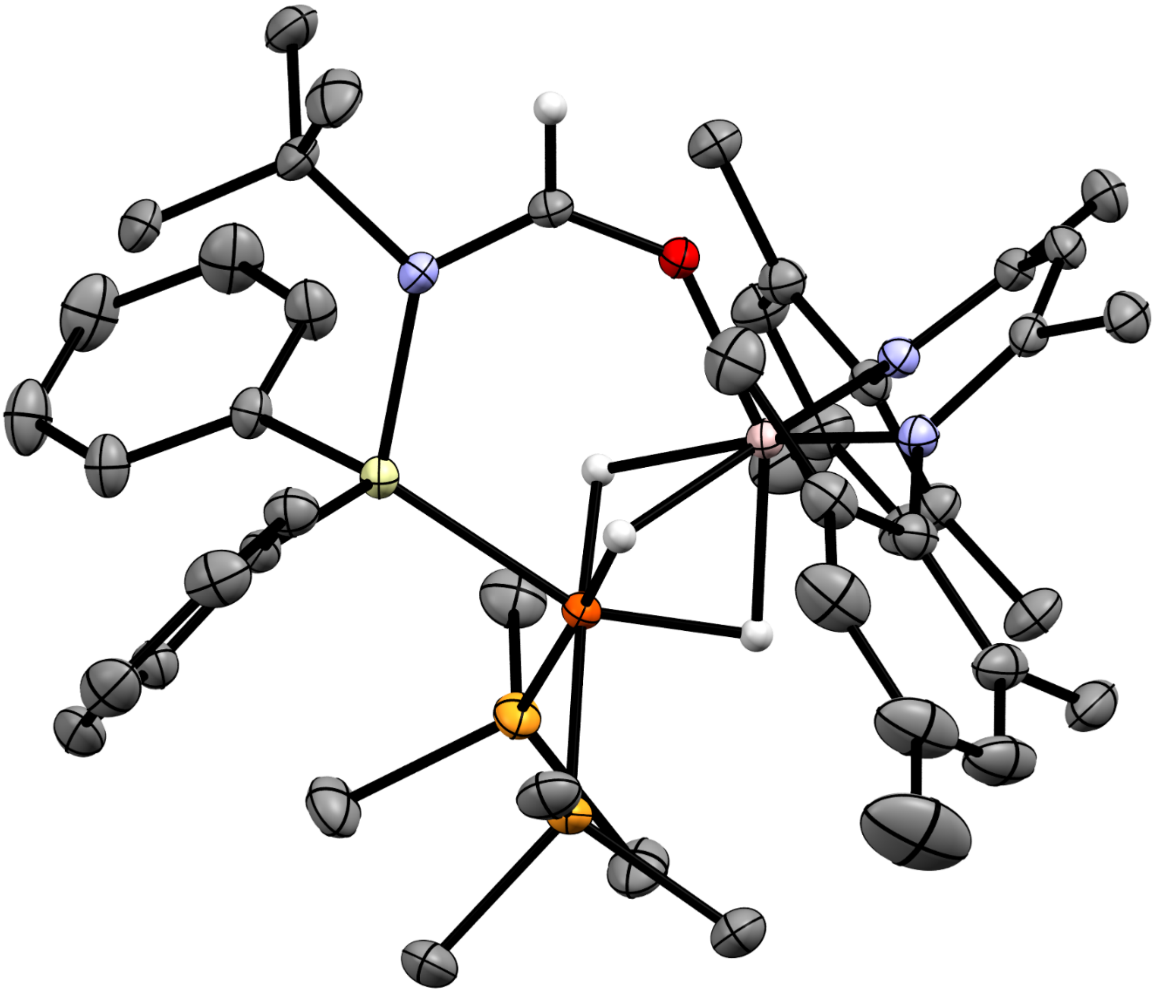

Figure S3 X-ray structure of **3b**. Most hydrogen atoms are hidden for clarity.

S2.2.3. **3c**

The asymmetric unit was found to contain 0.25 molecule of *n*-pentane solvent, disordered across a centre of inversion located ca. 0.27 Å from the central carbon. This was modelled in a single orientation (and its symmetry-related orientation), with only the non-hydrogen atoms being anisotropic. The solvent atoms were restrained to be similar. All three Fe–*μ*-***H***–Al hydrides and the Si–***H*** hydride were found in the Δ*F* map and freely refined. The NC(***H***)N atom was found in the Δ*F* map. It was ultimately treated using the conventional riding model which put it in a very similar position to the free refinement.

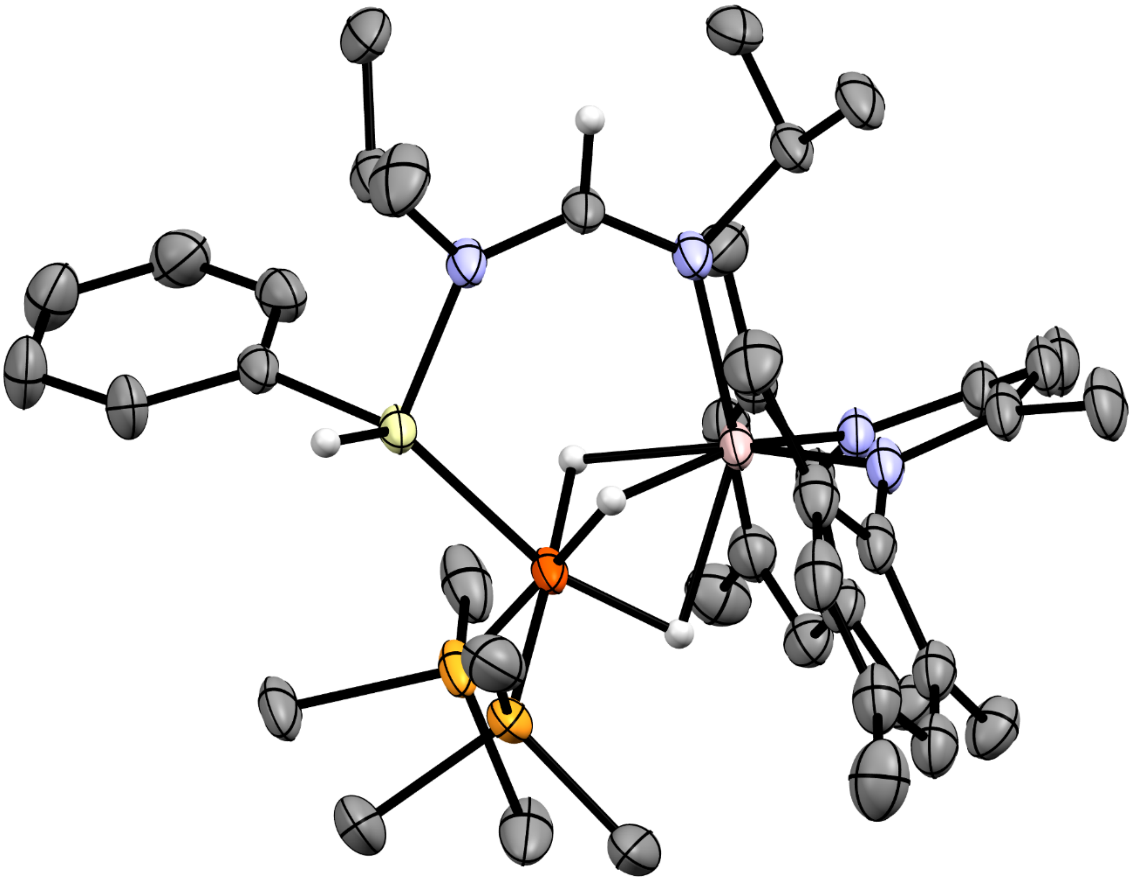

Figure S4 X-ray structure of **3c**. Most hydrogen atoms and the included solvent are hidden for clarity.

S2.2.4. **4a**

The asymmetric unit was found to contain 0.5 molecule of *n*-pentane disordered across a centre of inversion located ca. 0.22 Å from the central carbon. This was modelled in a single orientation (and its symmetry-related orientation), with only the non-hydrogen atoms being anisotropic. The ^{t}Bu group was found to be disordered. It was modelled in two orientations at a ratio of ca. 0.89:0.11, with only the non-hydrogen atoms of the major orientation being anisotropic, the rest isotropic. The phenyl group was found to be disordered. It was modelled in two orientations at a ratio of ca. 0.80:0.20, with only the non-hydrogen atoms of the major orientation being anisotropic, the rest isotropic. For all the above disorders the disordered orientations were restrained to be similar. All six hydrides were found in the ΔF map and freely refined.

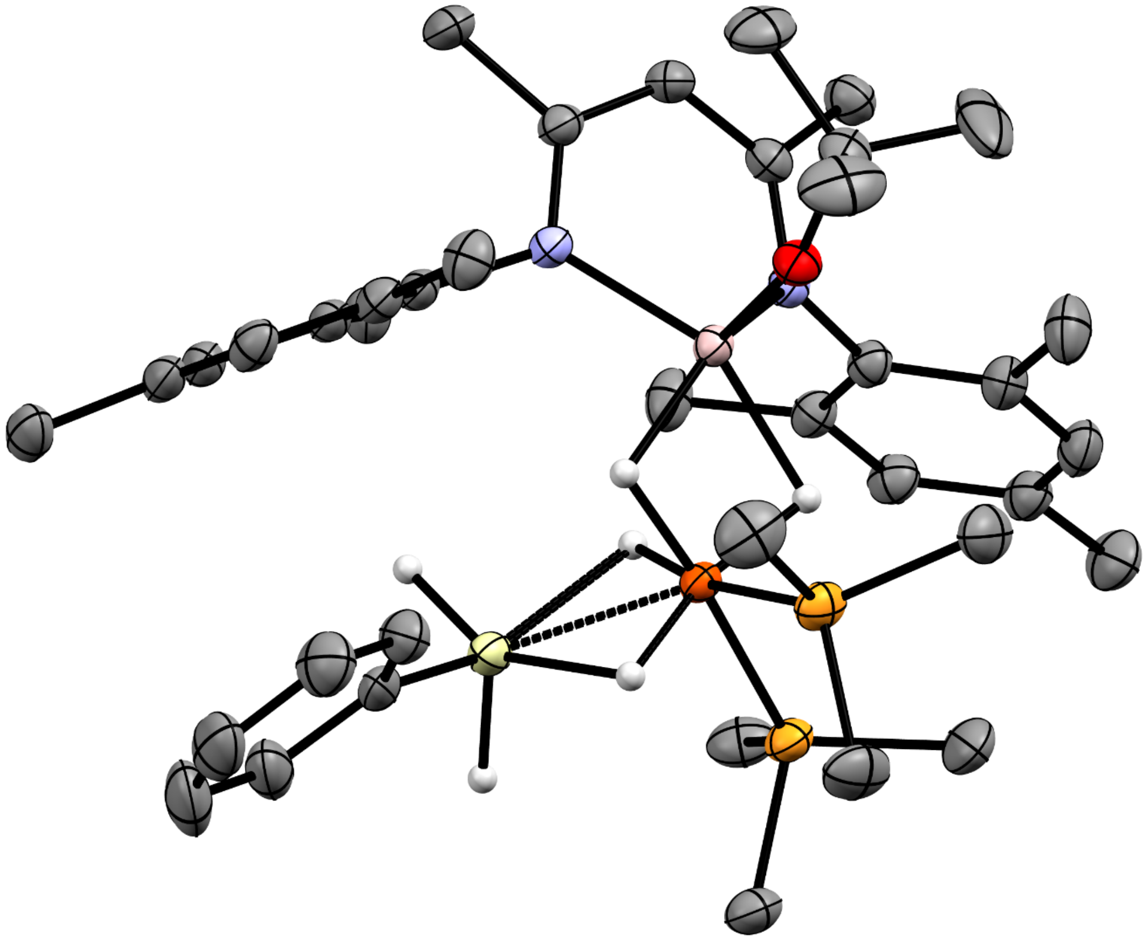

Figure S5 IAM X-ray structure of **4a**. Most hydrogen atoms, atoms of the minor occupancy orientation and included solvent are hidden for clarity.

S2.2.5. **4a-HAR**

This is a Hirshfeld Atom Refinement (HAR) of the dataset of **4a** using olex2.refine/NoSpherA2,[8] as implemented in Olex2.

The following settings were used for NoSpherA2: program: ORCA 6.0.1 (Windows version), method: B3LYP, basis set: def2-TZVP, charge: 0, multiplicity: 1, integration accuracy: normal, SCF threshold: NormalSCF, SCF strategy: NormalConv, solvation: hexane, dispersion: on. Initially, the def2-SVP basis set was used until the model was close to converged. Then, refinement was continued using an iterative strategy with the def2-TZVP basis set until full convergence was achieved.

All disorders were treated similarly to the IAM refinement, resulting in a ca. 0.12:0.88 occupancy ratio for the two orientations of the ^{t}Bu, and a ca. 0.20:0.80 occupancy ratio for the two orientations of the Ph group. All atoms including hydrogens of the non-disordered region and the major orientations of the disordered regions were modelled anisotropically, the minor orientations were modelled isotropically. Hydrogens of the major orientations and the non-disordered region were modelled freely. The hydrogens of the *n*-pentane solvent were modelled using the riding model but with free thermal parameters. Hydrogens of the minor occupancy orientations were modelled isotropically using the riding model for both cartesian coordinates and thermal parameters. Weak restraints were applied to the whole molecule to stop hydrogens becoming too flat.

We note that the poor data/parameter ratio of 7.70 is a result of the high parameter usage of the full free anisotropic refinement of hydrogens. The alert can be eliminated by masking the solvent using PLATON SQUEEZE, but it was determined that modelling it gave additional value, due to the exact match to the IAM model.

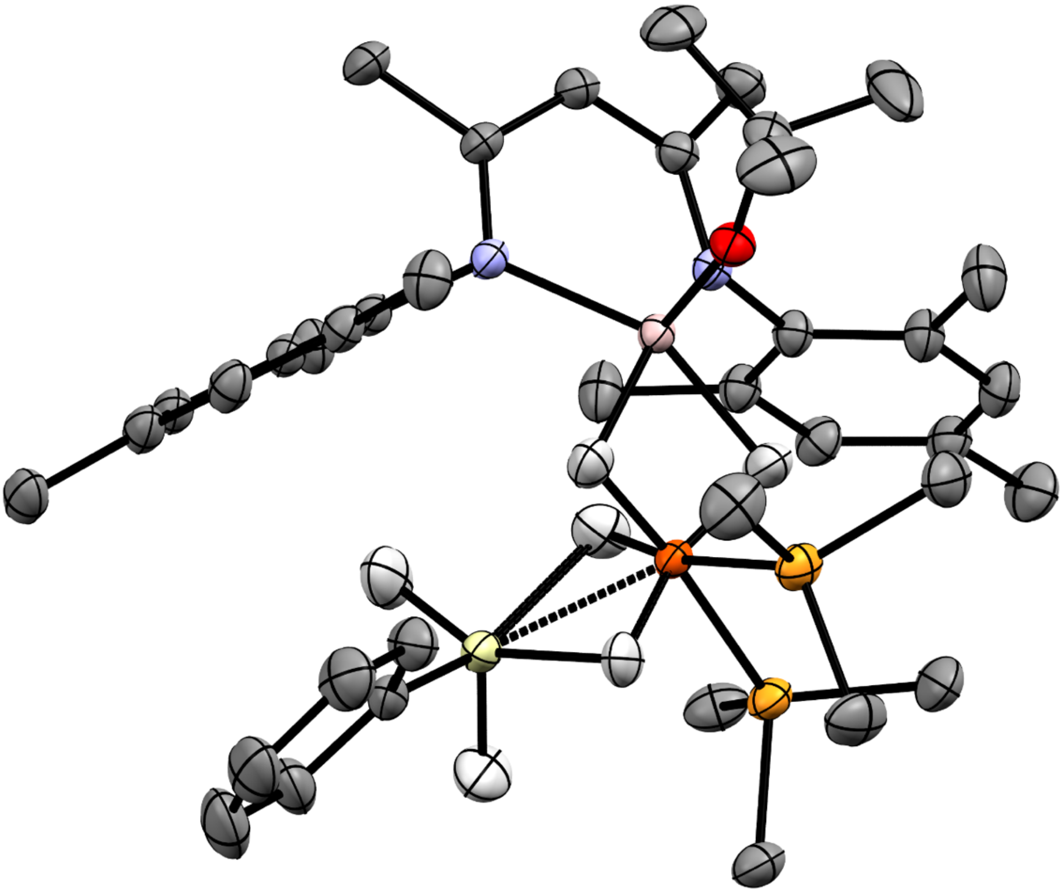

Figure S6 HAR X-ray structure of **4a**. Most hydrogen atoms, atoms of the minor occupancy orientation and included solvent are hidden for clarity.

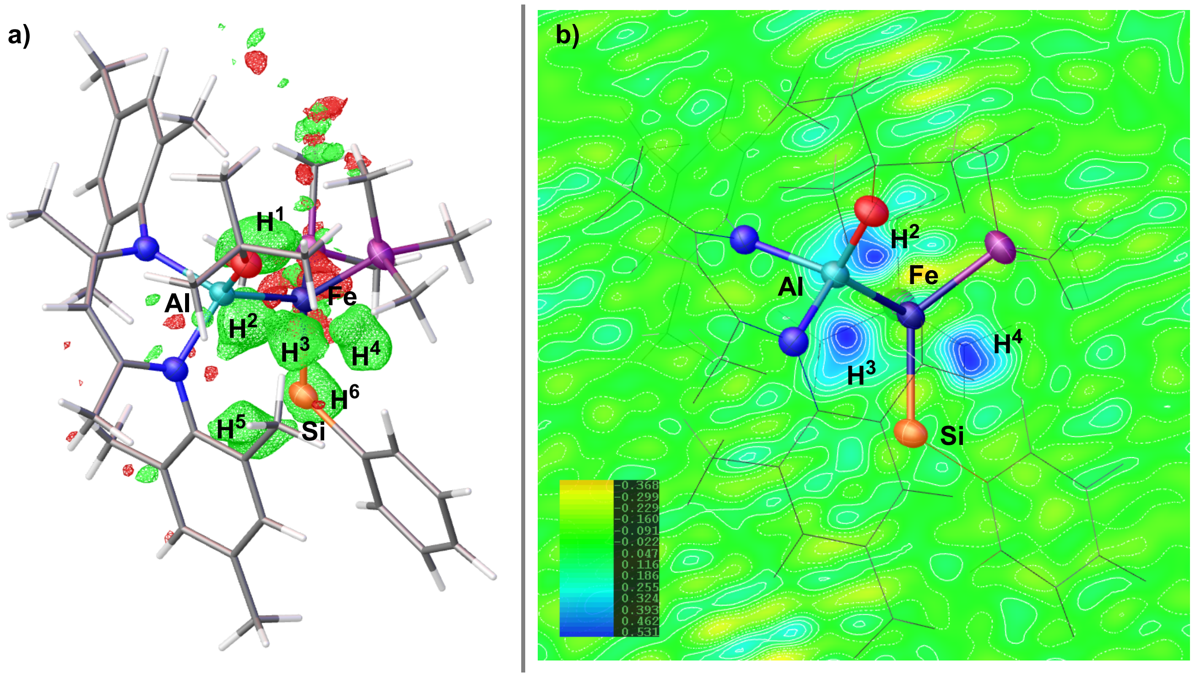

Figure S7 Difference electron density plots of the HAR structure of **4a**, with the 6 hydrides removed from the converged geometry. a) isosurface plot of Δ*F*, isovalue = 0.0251 eÅ^{-3}; b) coloured contour plot of Δ*F* in the H^2-H^3-H^4 plane. In both cases, the regions of positive electron density correspond to the hydrides.

S2.2.6. **5b**

The crystal was found to be a two-component (approximate occupancy ratio: 0.72:0.28) pseudo-merohedral twin disordered along a pseudo-C_2 axis described by the twin law [1 0 0 0 -1 0 0 0 -1] within the refinement program. Modelling the twinning as a non-merohedral twin was also attempted but this gave worse results. All three Fe–μ-***H***–Al hydrides in both independent molecules (6 total per ASU) were found in the ΔF map and freely refined.

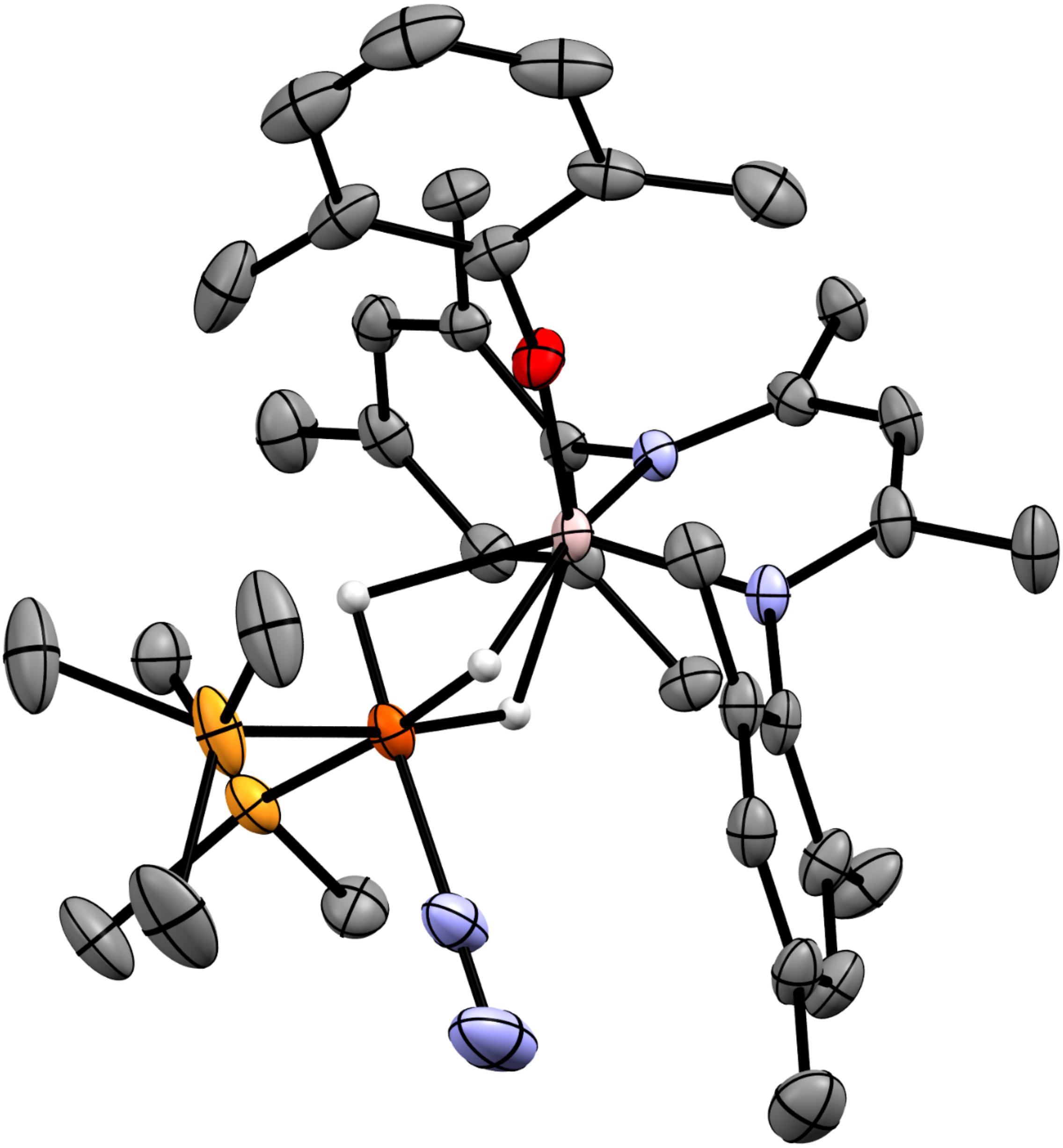

Figure S8 X-ray structure of **5b**. Most hydrogen atoms and the second independent molecule are hidden for clarity.

S2.2.7. **S4**

All three Fe–*μ*-***H***–Al hydrides were found in the Δ*F* map and freely refined.

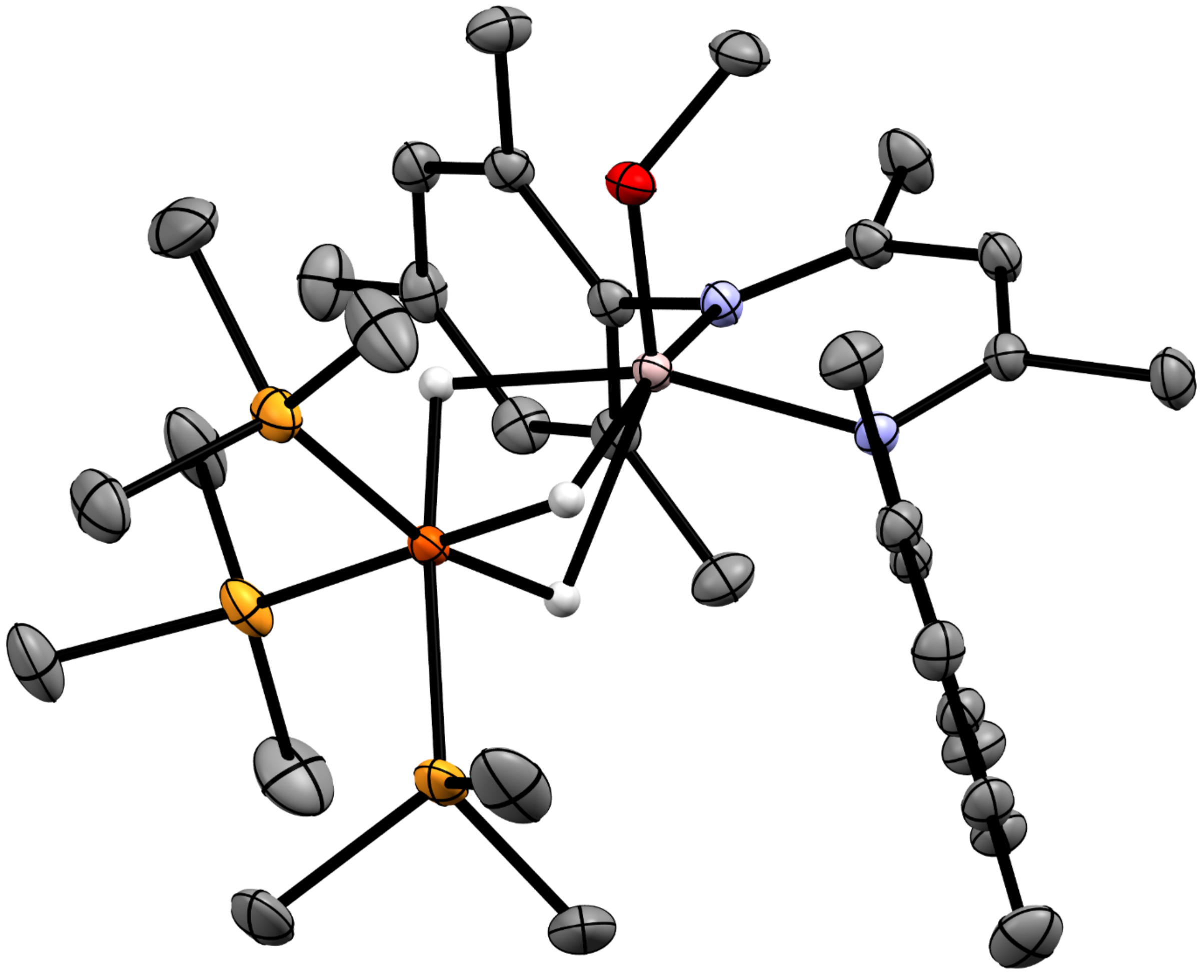

Figure S9 X-ray structure of **S4**. Most hydrogen are hidden for clarity.

S2.3. Summary of bond lengths and angles

	**2a**	**3b**	**3c**	**5b**	**S4**
d(Fe–Al) (Å)	2.197(1)	2.2580(6)	2.275(1)	2.3718(16) – 2.3848(14)	2.3605(5)
d(Fe–Si) (Å)	2.242(1)	2.2146(7)	2.1822(6)		
d(N–C) (Å)		1.316(2)	1.322(3)		
d(C–X) (X = N/O, Å)		1.268(2) (X = O)	1.321(3) (X = N)		
d(N=N) (Å)				1.107(8) – 1.114(8)	

Table S2 Important bond lengths for **2a**, **3b**, **3c**, **5b** and **S4**.

	**4a**	**4a-HAR**
d(Fe---Al) (Å)	2.4251(6)	2.4251(4)
d(Fe---Si) (Å)	2.2814(6)	2.2812(4)
d(Fe---H^1, Al---H^1) (Å)	1.51(2), 1.88(2)	1.561(16), 1.906(16)
d(Fe---H^2, Al---H^2) (Å)	1.48(2), 1.83(2)	1.550(16), 1.859(15)
d(Fe---H^3, Si---H^3, Al---H^3) (Å)	1.48(2), 2.04(2), 2.37(2)	1.532(16), 2.094(17), 2.382(17)
d(Fe---H^4, Si---H^4) (Å)	1.42(2), 1.82(2)	1.463(16), 1.842(15)
d(Si---H^5, Si---H^6) (Å)	1.44(3), 1.47(3)	1.498(16), 1.493(16)
a(Fe-H^1-Al, Fe-H^2-Al, Fe-H^3-Al) (°)	90.7(10), 93.8(11), 73.9(10)	88.2(7), 90.3(7), 73.0(7)
a(Fe-H^3-Si, Fe-H^4-Si) (°)	78.8(10), 88.5(12)	76.2(7), 86.5(8)

Table S3 Important bond lengths and angles for **4a** (IAM) and **4a-HAR**.

S3. Computational section

S3.1. General computational

S3.1.1. Optimisations

All geometry optimisations were performed using ORCA 6.0.0.[9–11]

Geometry optimisation calculations were performed without symmetry constraints. We employed the r^2SCAN-3c composite method developed by the Grimme group.[12] This uses the regularised and restored SCAN *meta*-GGA functional.[13–17] Similarly to other Grimme group "3c" methods, it uses well-balanced approximations to give optimal speed for reasonable accuracy: the polarised triple-ζ basis set termed mTZVPP (a modified version of def2-TZVP[18]), the atomic-charge dependent D4 dispersion correction[19] and the geometrical counterpoise (gCP)[20] correction to reduce BSSE. Solvation was modelled using the Conductor-like Polarizable Continuum Model (CPCM) using benzene solvent. All calculations were performed with the resolution of identity approximation for the Coulomb integrals, and chain of spheres approximation for the exchange integrals (RIJCOSX)[21–23] with the def2/j auxiliary basis set,[24] as is the default in ORCA v5+. For pure functionals, this defaults to the Split-RI-J algorithm.

Analytical frequency analyses for all stationary points were performed using the enhanced criteria to confirm the nature of the structures as either minima (no imaginary frequency) or transition states (only one imaginary frequency). The thermostatistical corrections used the q-RRHO approximation as is the default in ORCA 6.0.0 using unscaled frequencies.

N_2 was treated as an unsolvated gas.

S3.1.2. Single-point corrections

The electronic energies were recomputed using the def2-QZVPP basis set[18] for all atoms, with no effective core potentials. The mPW1LYP-D4[25] exchange-correlation functional was used. Solvent corrections were applied using the universal solvent model (SMD) using parametrisation for benzene solvent.[26] Dispersion corrections were applied using Grimme's atomic-charge dependent D4 dispersion correction.[19] A correction for the change of state of 1.89 kcal/mol was added to each species.

S3.1.3. Conformer searching

For all species, a full conformational search was conducted using the GOAT procedure[27] as included in ORCA 6.0.0, at the GNF2-xTB[28] / ALPB(benzene)[29] level of theory, as part of the xTB 6.7.1 package bundled with ORCA 6.0.0. For some species where GFN2-xTB failed to converge the wavefunction GFN1-xTB was used instead. GFN1-xTB appears to perform similarly for this system in terms of qualitatively similar geometries, but tight-binding geometries and energies were not investigated in detail.

As per before, we note that the use of GFN-FF[30] gave significant speed-ups, but was found to give a qualitatively incorrect model for the bridging hydrides of the system.

The ca. 50 lowest energy conformers were reoptimized with DFT for each species.

S3.1.4. Wavefunction analysis

NBO analysis was performed using NBO 7.0[31] as interfaced with ORCA 5.0.4. The LC-BLYP-D4/def2-TZVPP/SMD(benzene) level of theory was used on the previously optimised geometries.

QTAIM analyses were performed using the AIMAll package (version 17.01.25). The LC-BLYP-D4/def2-TZVPP/SMD(benzene) level of theory was used on the previously optimised geometries.

Localised orbital calculations were performed using ORCA 6.0.0. The LC-BLYP-D4/def2-TZVPP/SMD(benzene) level of theory was used on the previously optimised geometries, using the IBO[32] localisation criteria. Atomic contributions to the localised orbitals were taken from the localisation procedures or from subsequent a Löwdin population analysis.

ETS-NOCV[33] calculations were performed in the ORCA 5.0.4 [11] with optimised geometries obtained at the regular optimisation level of theory. ORCA 6.0.0 could not be used as NOCV calculations are disabled. The LC-BLYP-D4/def2-QZVPP/SMD(benzene) level of theory was used on the previously optimised geometries.

FOD analysis[34] was performed in ORCA 6.0.0, as defined by the FOD keyword. This means, the TPSS functional, the def2-TZVP basis set and an electronic temperature of T_{el} = 5000 K were used. FOD analysis was performed using the same implicit solvent model as single point calculations (SMD, benzene).

S3.2. Computational benchmarking

S3.2.1. Benchmarking methodology

Changing from Gaussian to ORCA in comparison from our previous publications on the system, we performed benchmarking with respect to experimental data. Namely, the Eyring analysis for the intramolecular C–H activation of **1** (**TS**-S1, $\Delta G^{\ddagger}_{exp}$ = 27.1 kcal/mol, Figure S10 left),[2] and the van't Hoff analysis for the reaction between **1** and styrene (**S6**, ΔG_{exp} = −0.4 kcal/mol, Figure S10 right).[35]

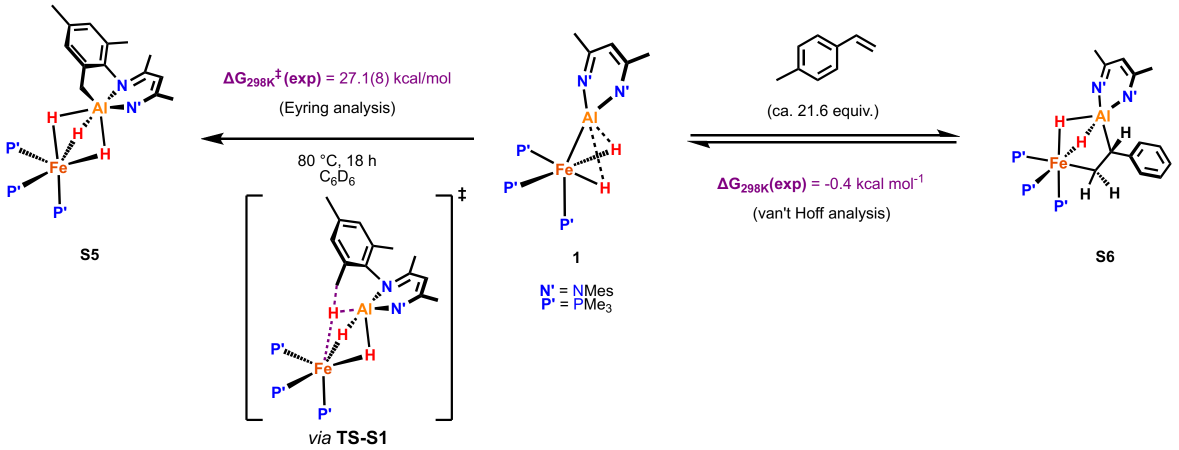

Figure S10 Left: intramolecular C–H activation of **1** (Eyring analysis)[2] and right: equilibrium between **1** and styrene (van't Hoff analysis)[35] used for computational benchmarking.

Gibbs free energies are given below, including the 1.89 kcal/mol correction for the change of state from gas to liquid.

For composite methods, their associated basis sets were used. For other optimisations, the following hybrid basis set was used: def2-TZVPP(Fe,Al)/def2-TZVP(-f)(O,N,P)/def2-SVP(H,C). For single point corrections, unless otherwise specified, the def2-QZVPP basis set was used for all atoms. All optimisations used the CPCM(toluene) and single point corrections the SMD(toluene) implicit solvation models. For all calculations, the D4 dispersion correction was applied, unless the method already includes dispersion in its definition, either explicitly (coupled cluster methods), or the definition already includes a dispersion correction (composite methods).

S3.2.2. Benchmarking results

Optimisation	Single Point	S6	TS-S1
Experiment		**−0.4**	**+27.1**
ωB97X-3c		−11.2	+29.3
	DLPNO-CCSD(T)/def2-TZVPP	+0.0	+34.6
	ωB97X-D4rev	−7.4	+28.8
	ωB97M-D4rev	−6.7	+29.1
	wB97M(2)	+13.3	+32.8
	CAM-B3LYP-D4	−4.8	+28.4
ωB97X-D4rev		−13.9	+29.1
	ωB97X-D4rev	−12.2	+29.0
r^2SCAN-3c		−4.5	+21.1
	DLPNO-CCSD(T)/def2-TZVPP	−1.9	+33.7
	ωPr^2SCAN50-D4	+6.2	+29.5
	ωB97X-D4rev	−8.4	+28.2
	B3LYP-D4	−1.6	+22.8
	CAM-B3LYP-D4	−5.0	+27.9
	**LC-BLYP-D4**	**−4.4**	**+28.4**
	PWPB95-D4	+3.8	+27.2
	PW1PW-D4	−5.4	+23.6
	mPW1PW-D4	−7.2	+22.6
	**mPW1LYP-D4**	**−1.0**	**+25.0**
	PW6B95-D4	−6.0	+23.8
	r^2SCAN-D4	−1.6	+21.4
	r^2SCANh-D4	−3.7	+22.9
	r^2SCAN0-D4	−7.5	+24.8
	TPSS-D4	−2.2	+19.5
	TPSSh-D4	−4.4	+21.4
	TPSS0-D4	−8.5	+23.6

Table S4 Computational thermochemistry benchmarking results.

Optimisation	Single Point	styrene van't Hoff	Ligand C-H activation TS
B3LYP-D4		−11.0	+22.7
	DLPNO-CCSD(T)/def2-TZVPP	−1.3	+34.7
	B3LYP-D4	−1.7	+23.5
	CAM-B3LYP-D4	−5.4	+27.8
	B2PLYP-D4	+14.5	+29.9
	B2GP-PLYP-D4	+11.6	+32.0
	mPW2PLYP-D4	+12.2	+30.9
	SCS-B2GP-PLYP21-D4	+11.6	+32.0
	DSD-BLYP-D3BJ	+10.7	+32.1
	PWPB95-D4	+3.8	+27.4
CAM-B3LYP-D4		−14.3	+26.7
	CAM-B3LYP-D4	−4.8	+27.8

Table S4 continued.

S3.2.3. Summary of computational benchmarking results

We found that the r^2SCAN-3c/CPCM(toluene/benzene) level of theory gave geometries that were close to crystal structures and accurate energies using the right single point correction method, for a reasonable computational cost. Marginally better geometries were obtained using the ωB97X-3c/CPCM(toluene/benzene),[36] but for a significant additional computational cost (up to 2-3x SCF timings, up to 2-3x geometry optimisation cycles).

Using the SMD instead of the CPCM implicit solvation model for optimisations yielded only marginal improvements, but was found to be computationally more expensive, as well as often giving low negative frequencies on stationary points that should be minima.

Based on the above results the r^2SCAN-3c/CPCM(benzene) level of theory was chosen for geometry optimisations.

No method gave perfect agreement with both thermodynamic and kinetic data. Namely, the DLPNO-CCSD(T)/def2-TZVPP/SMD(toluene)/ωB97X-3c/CPCM(toluene) *ab initio* method gave the best agreement with van't Hoff analysis, however, this was found to severely overestimate transition state energies, and for a very high computational cost. The generally very reliable B97-based range separated hybrids overestimated transition state energies and overstabilised intermediates. These results are possibly explained by the fact that the system exhibits some amount of multireference character, as demonstrated by FOD analysis (see section S3.4.3).

For DFT methods, B3LYP-D4 gave good thermodynamics, but vastly underestimated barrier heights. Ultimately, the mPW1LYP-D4[25] global hybrid functional was chosen as it gave balanced errors for both kinetics and thermodynamics on r^2SCAN-3c geometries. The LC-BLYP-D4[37] range separated hybrid functional appeared to be a close second with slightly different errors. This was used for wavefunction analysis.

S3.3. Energies of all stationary points

Absolute energies include dispersion and solvation corrections and are reported in eH.

Single point corrected ΔG values are reported in kcal/mol with respect to **1** + substrates according to reaction stoichiometry.

Species	G(r^2SCAN-3c)	E(r^2SCAN-3c)	E(mPW1LYP-D4)	ΔG
**1**	-3893.06429534	-3893.81012037799	-3894.29283958896	
$PhSiH_3$	-522.7705525	-522.85346598893	-522.951795458664	
Ph_2SiH_2	-753.70355415	-753.862622346676	-754.010686799101	
Ph_3SiH	-984.63715514	-984.873707975355	-985.071239345199	
^{t}BuOH	-233.51401498	-233.619767915719	-233.668253478147	
XylOH	-385.89418455	-386.020539704199	-386.103213085409	
PMe_3	-460.94832311	-461.031059560014	-461.104997638118	
N_2	-109.52058698	-109.507075358441	-109.539595776828	
**2a**	-3954.91177055	-3955.66066410228	-3956.17101707953	−17.9
**2b**	-4185.84450874	-4186.67104647109	-4187.23001136896	−17.0
**2c**	-4416.77023477	-4417.67591439182	-4418.28262845443	−11.0
**4a**	-4188.45150231	-4189.32982732344	-4189.88263009214	−32.1
**4b**	-4419.38302503	-4420.33980924128	-4420.94196153660	−31.0
**4c**	-4650.30401694	-4651.34215580789	-4651.99010226358	−20.7
**4d**	-4340.83427548	-4341.73507541383	-4342.32257805758	−34.1
**4e**	-4571.76432970	-4572.74384005053	-4573.38030051916	−31.7
**4f**	-4802.68261349	-4803.74335845126	-4804.42411255313	−18.9
**5a**	-3775.20383886	-3775.97825311559	-3776.46583893391	−32.0
**5b**	-3927.59256958	-3928.38925702324	-3928.91178938527	−37.9
**5c**	-4126.63949699	-4127.51659969639	-4128.05050435845	−42.0
**5d**	-4279.02427415	-4279.92380197609	-4280.49365333406	−46.0

Table S5 Computed energies of stationary points.

S3.4. Wavefunction analysis

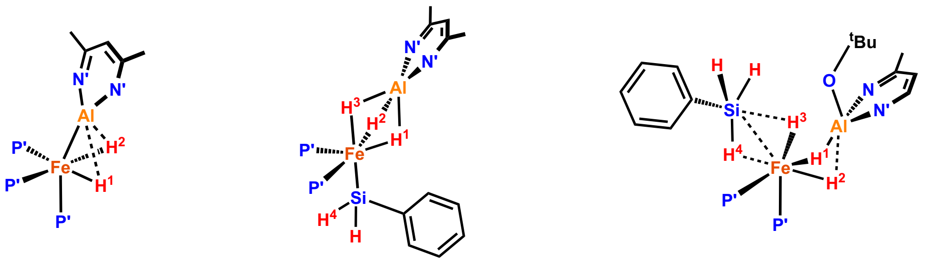

1 **2a** **4a**

Figure S11 Hydride atom numbering for wavefunction analyses.

S3.4.1. NBO analysis

	**1**	$PhSiH_3$	**2a**	**4a**
**NPA charge**				
Fe	-1.16		-1.1	-1.17
Al	+1.14		+1.62	+1.88
H^1	-0.19		-0.24	-0.13
H^2	-0.16		-0.15	-0.14
H^3			-0.13	+0.01
Si		+0.84	+0.74	+0.93
H^4		-0.15	-0.20	-0.01
**WBI**				
Fe–Al	0.41		0.18	0.12
Fe–H^1	0.38		0.24	0.38
Fe–H^2	0.35		0.36	0.38
Fe–H^3			0.34	0.36
Fe–H^4				0.30
Al–H^1	0.38		0.41	0.25
Al–H^2	0.33		0.28	0.25
Al–H^3			0.31	0.08
Fe–Si			0.50	0.26
Si–H^3				0.36
Si–H^4		0.95	0.90	0.34

Table S6 Numerical data from NBO analyses.

S3.4.2. QTAIM analysis

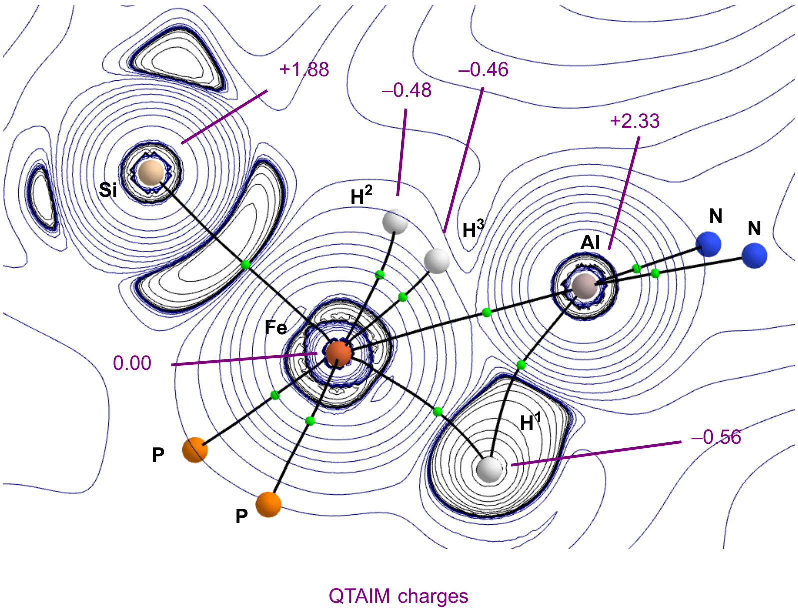

BCP	ρ(r)	$\nabla^2\rho(r)$
**Fe–Al**	0.063	0.142
**Fe–H^1**	0.086	0.238
**Fe–H^2**	0.106	0.245
**Fe–H^3**	0.100	0.237
**Fe–Si**	0.086	-0.016
**Al–H^1**	0.064	0.133

Figure S12 QTAIM analysis of **2a**. The background shows the Laplacian of the electron density in the Si-Fe-Al plane.

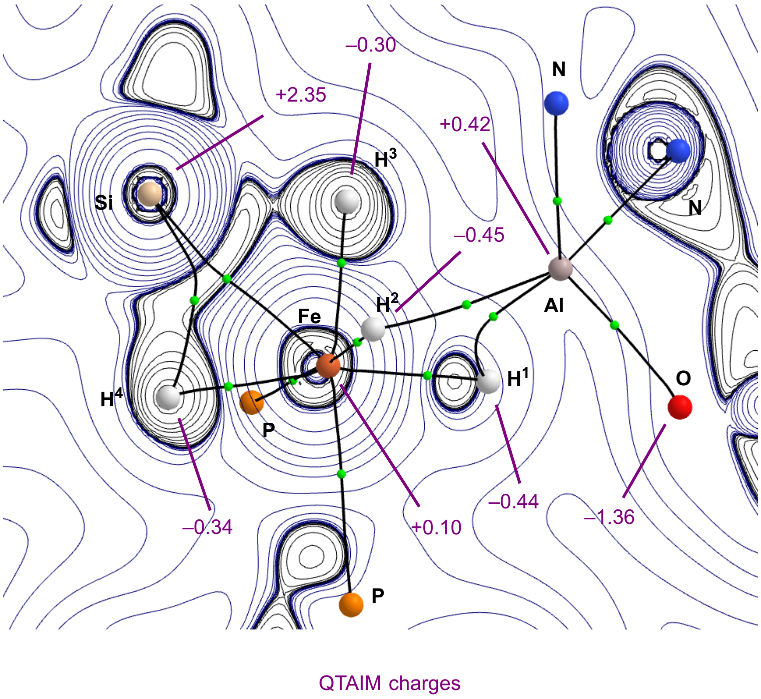

BCP	ρ(r)	$\nabla^2\rho(r)$
**Fe–H^1**	0.110	0.213
**Fe–H^2**	0.114	0.243
**Fe–H^3**	0.121	0.173
**Fe–H^4**	0.118	0.201
**Al–H^1**	0.053	0.070
**Al–H^2**	0.053	0.073
**Al–O**	0.090	0.593
**Fe–Si**	0.082	-0.031
**Si–H^4**	0.081	-0.053

Figure S13 QTAIM analysis of **4a**. The background shows the Laplacian of the electron density in the Si-H^3-H^4 plane.

S3.4.3. FOD analysis

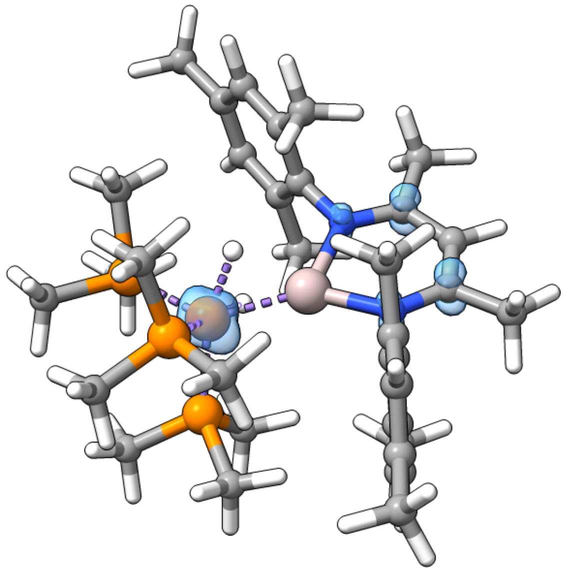

Figure S14 FOD analysis of **1**: Isosurfaces with left: isovalue = 0.005, right: isovalue = 0.002, N^{FOD} = 0.758.

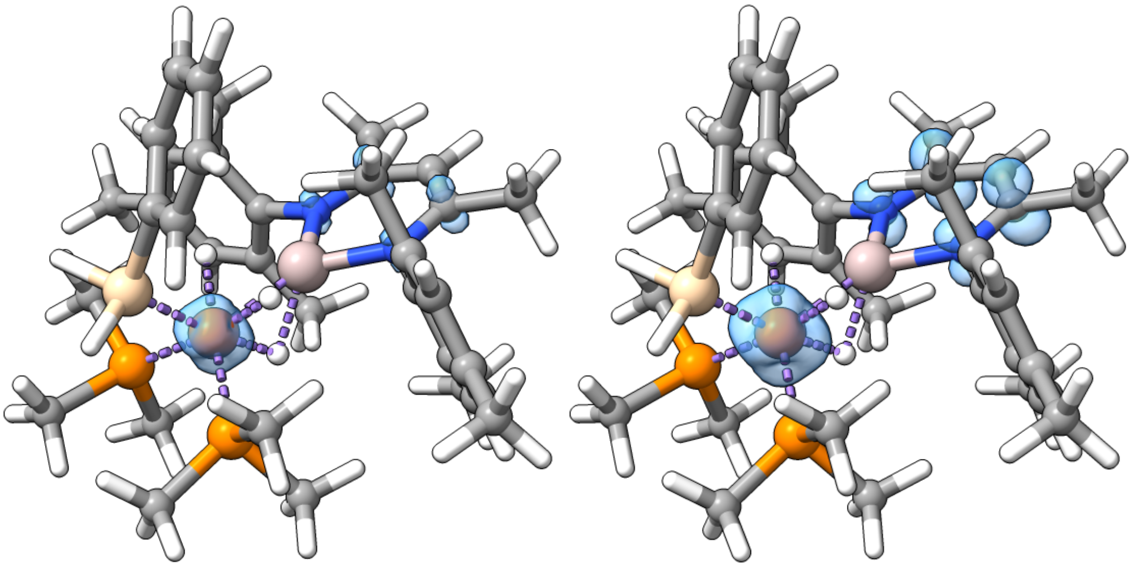

Figure S15 FOD analysis of **2a**: Isosurfaces with left: isovalue = 0.005, right: isovalue = 0.002, N^{FOD} = 0.572.

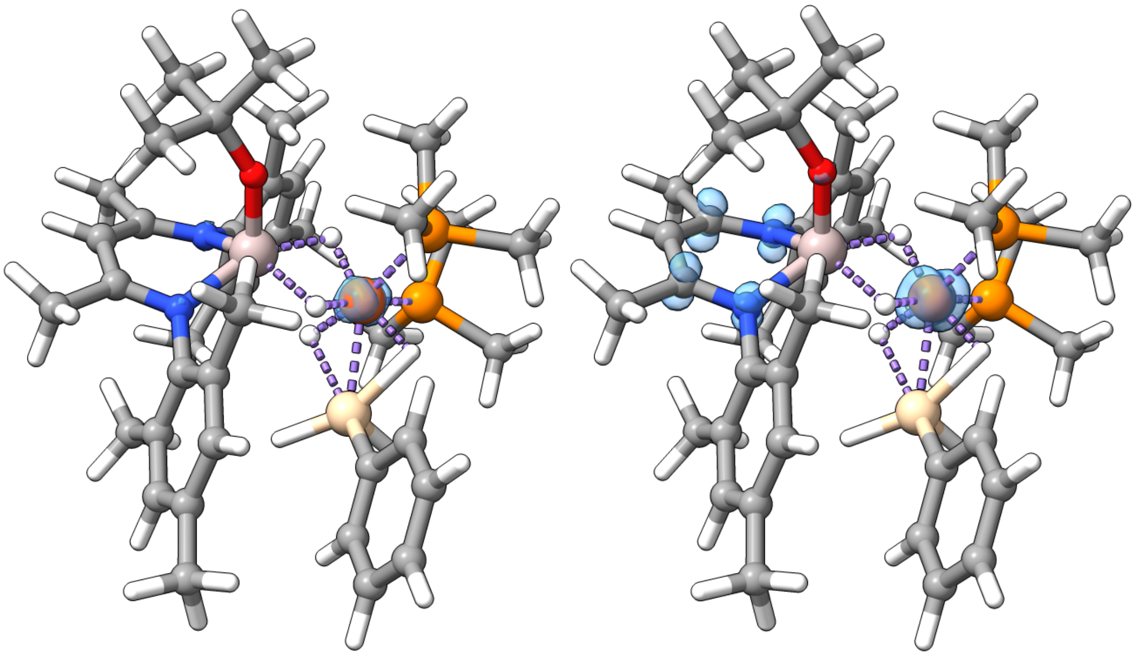

Figure S16 FOD analysis of **4a**: Isosurfaces with left: isovalue = 0.005, right: isovalue = 0.002, N^{FOD} = 0.336.

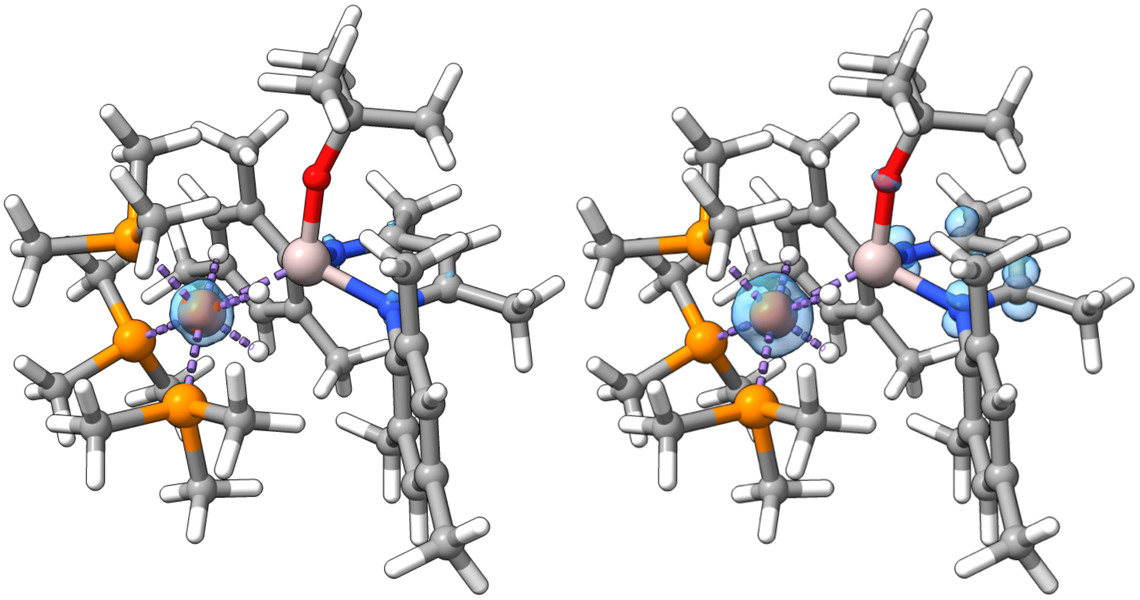

Figure S17 FOD analysis of **5c**: Isosurfaces with left: isovalue = 0.005, right: isovalue = 0.002, N^{FOD} = 0.413.

S3.4.4. IBO analysis of **4a**

**Orbital/composition**	**Isosurface**
**Fe–H–Si**<br><br>Fe 32.5% (2.7% s, 11.7% p, 18.1% d)<br>H 36.1% (35.6% s)<br>Si 21.7% (3.8% s, 12.1% p)	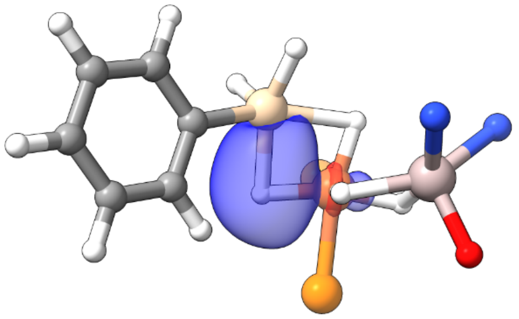
**Fe–H–Si**<br><br>Fe 34.9% (3.1% s, 9.9% p, 21.9% d)<br>H 36.9% (36.7% s)<br>Si 16.6% (2.0% s, 8.9% p, 5.1% d)	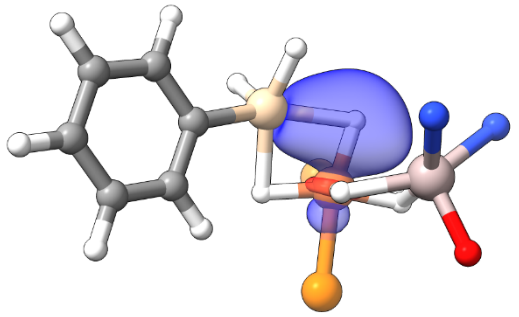
**Fe–Si**<br><br>Fe 79.2% (79.1% d)<br>Si 6.8% (4.5% p)	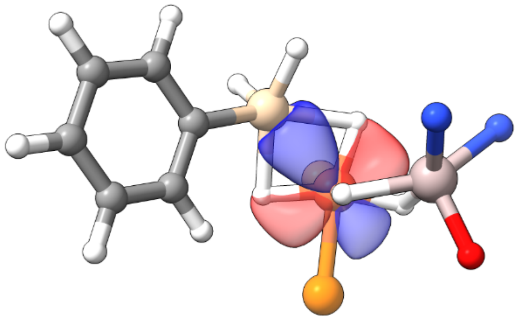
**Fe–H–Al**<br><br>Fe 33.5% (3.1% s, 10.0% p, 20.4% d)<br>H 37.2% (37.0% s)<br>Al 18.1% (3.5% s, 7.6% p)	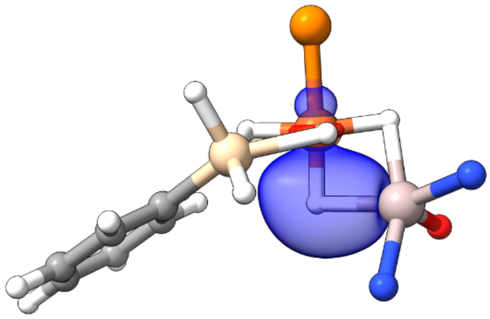
**Fe–H–Al**<br><br>Fe 34.1% (2.5% s,11.9% p, 19.7% d)<br>H 37.7% (37.4% s)<br>Al 18.6% (3.6% s, 7.8% p)	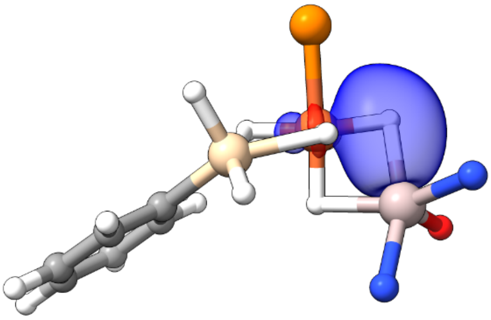

Table S7 IBO analysis of **4a**. Isovalue = 0.05.

S3.4.5. ETS-NOCV analysis of **4a**

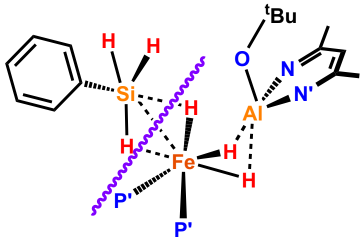

4a N' = NMes
P' = PMe_3

ΔE_{orb} = −110.6 kcal/mol

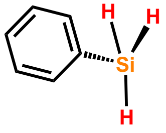

frag1
charge = 0
multiplicity = 1

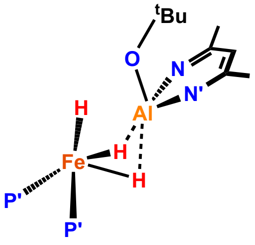

frag2
charge = 0
multiplicity = 1

Deformation densities are shown with an isovalue of 0.01 with charge from red to blue.

The individual donor and acceptor NOCVs are shown with an isovalue of 0.1.

Energy, composition	Deformation density	Donor NOCV	Acceptor NOCV
**$\Delta\rho_1$** = −58.2 kcal/mol (52.3 %)<br><br>**Donor**<br>54.1 % Fe (18.1 % d_{xy}, 12.4 % d_{z2}, 11.9 % d_{yz})<br>11.6 % H^3 (10.1 % s)<br>0.9 % H^4<br>15.7 % Si (5.5 % s, 4.5 % p_z)<br><br>**Acceptor**<br>27.5 % Fe (9.4 % d_{z2})<br>4.4 % H^3<br>18.8 % H^4 (16.3 % s)<br>32.0 % Si (5.3 % p_x, 5.2 % p_z, 4.7 % d_{z2})	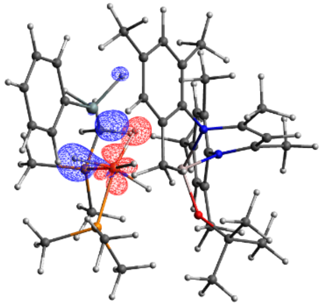	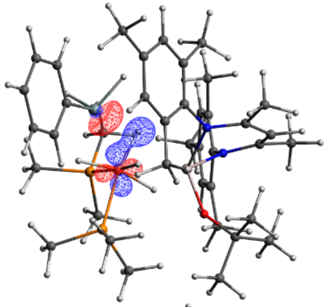	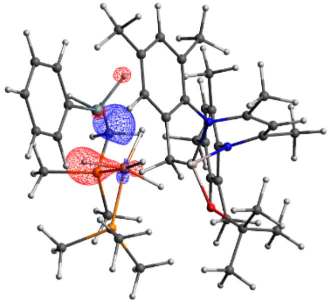
	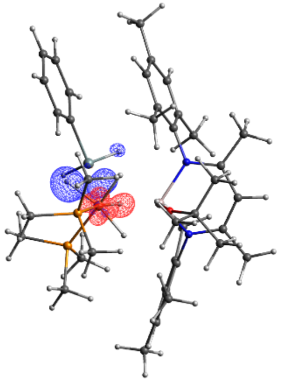	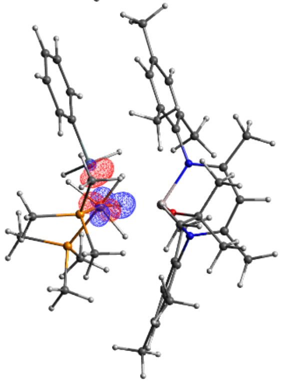	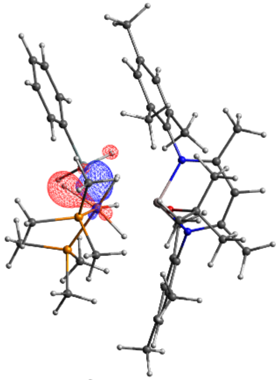
	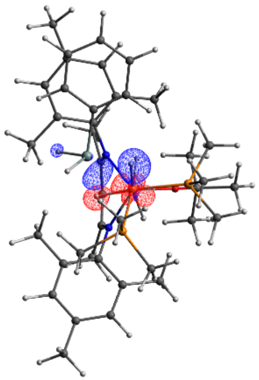	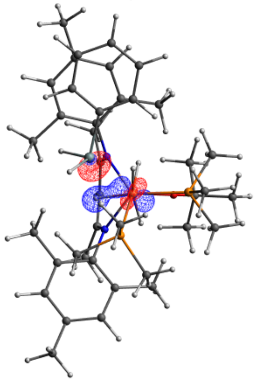	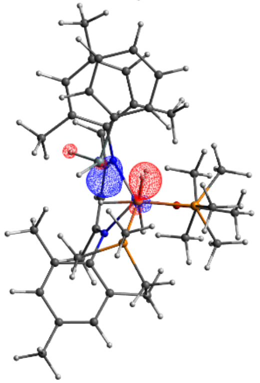

Table S8 ETS-NOCV analysis of **4a**. ΔE_{orb} = −110.6 kcal/mol.

**$\Delta\rho_2$** = −31.6 kcal/mol (28.8 %)<br><br>**Donor**<br>31.1 % Fe (14.1 % d_{z2}, 9.8 % d_{xz})<br>1.2 % H^3<br>25.3 % H^4<br>18.7 % Si (5.4 % p_y, 4.5 % p_x)<br><br>**Acceptor**<br>58.9 % Fe (27.5 % d_{xz}, 5.9 % d_{yz}, 4.4 % d_{xy})<br>3.3 % H^3<br>6.9 % H^4<br>12.3 % Si	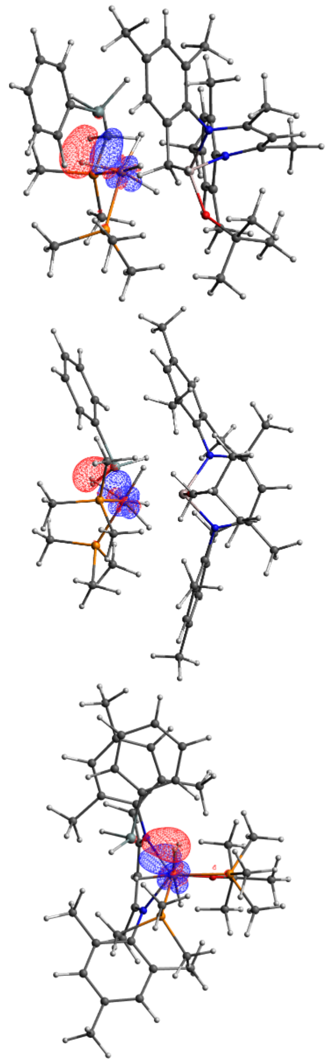	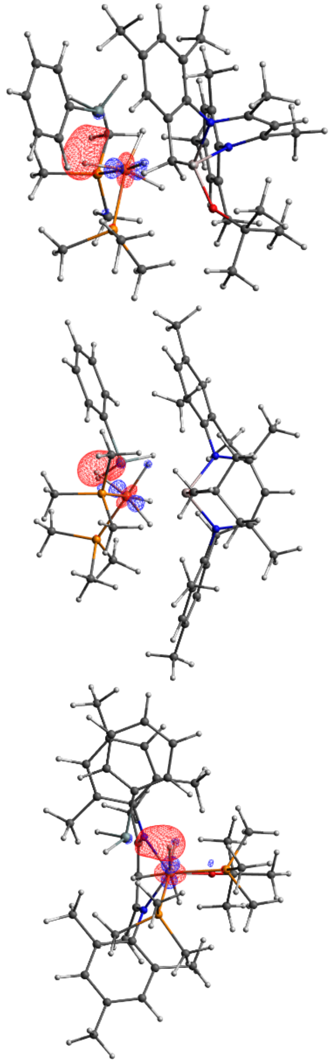	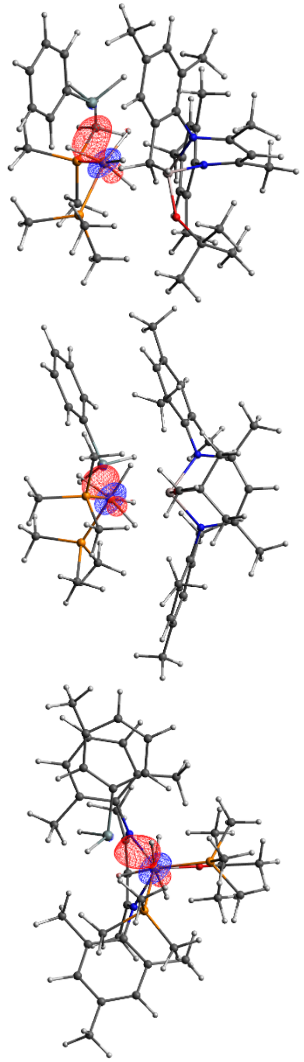

Table S8 continued. ETS-NOCV analysis of **4a**. ΔE_{orb} = −110.6 kcal/mol.

**Δρ₃** = −8.5 kcal/mol (7.5 %)<br><br>**Donor**<br>45.8 % Fe (15.7 % d_{x2-y2}, 15.2 % d_{yz})<br>2.2 % H^3<br>0.8 % H^4<br>21.7 % Si (9.7 % p_x)<br><br>**Acceptor**<br>30.9 % Fe (10.9 % d_{yz}, 8.1 % d_{x2-y2})<br>8.6 % H^3 (7.6 % s)<br>15.6 % H^4<br>24.7 % Si (6.8% d_{z2}, 5.5 % d_{x2-y2})	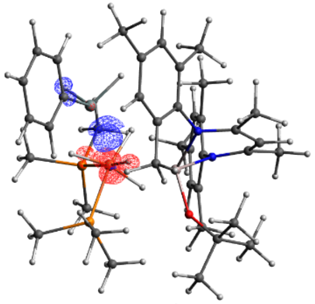<br>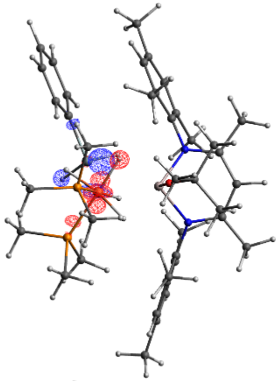<br>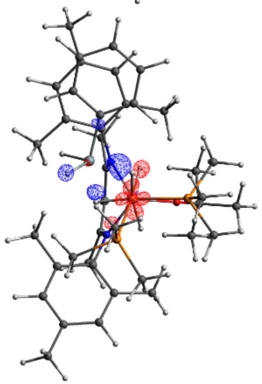	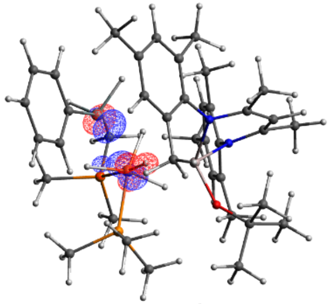<br>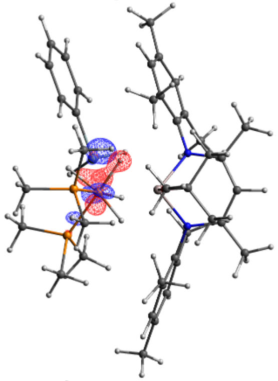<br>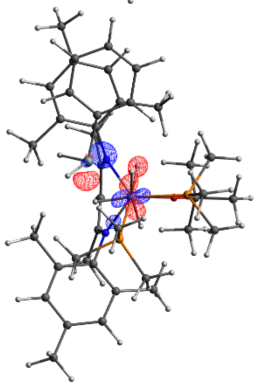	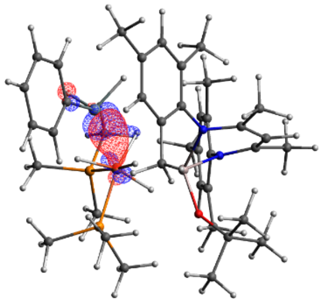<br>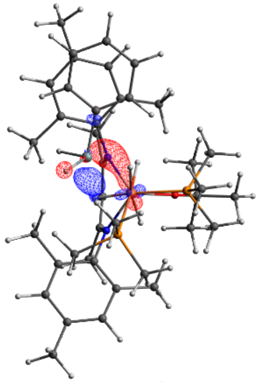<br>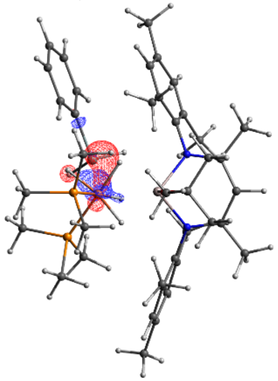

Table S8 continued. ETS-NOCV analysis of **4a**. ΔE_{orb} = −110.6 kcal/mol.

Δρ₄ = −4.5 kcal/mol (4.0 %)

Donor

34.0 % Fe (10.4 % d_{yz}, 8.5 % d_{x2-y2})

3.3 % H^3

5.4 % H^4

22.1 % Si (10.0 % p_y)

Acceptor

58.9 % Fe (27.5 % d_{xz}, 5.9 % d_{yz}, 4.4 % d_{xy})

3.3 % H^3

6.9 % H^4

12.3 % Si

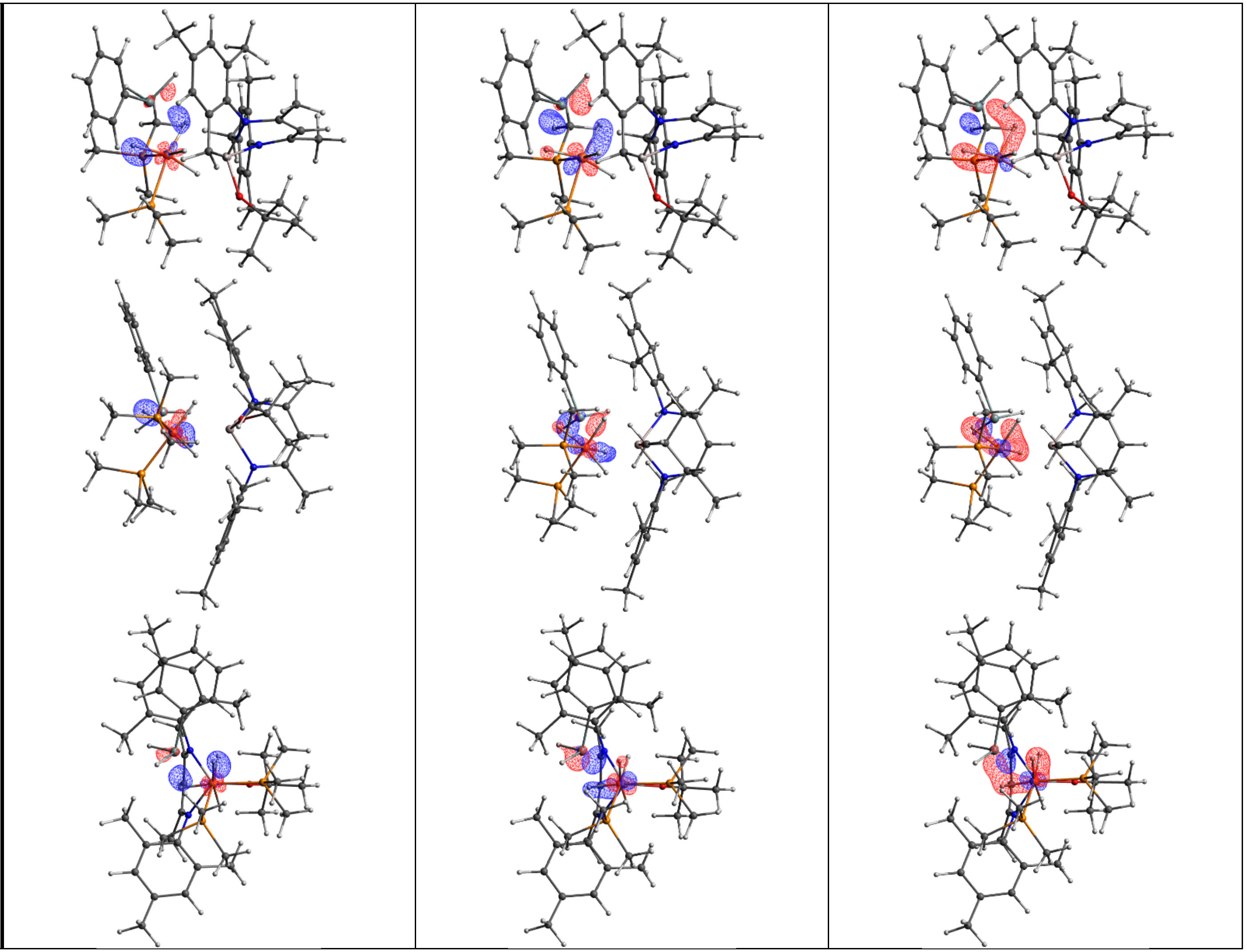

Table S8 continued. ETS-NOCV analysis of **4a**. ΔE_{orb} = −110.6 kcal/mol.

S4. NMR and IR spectra

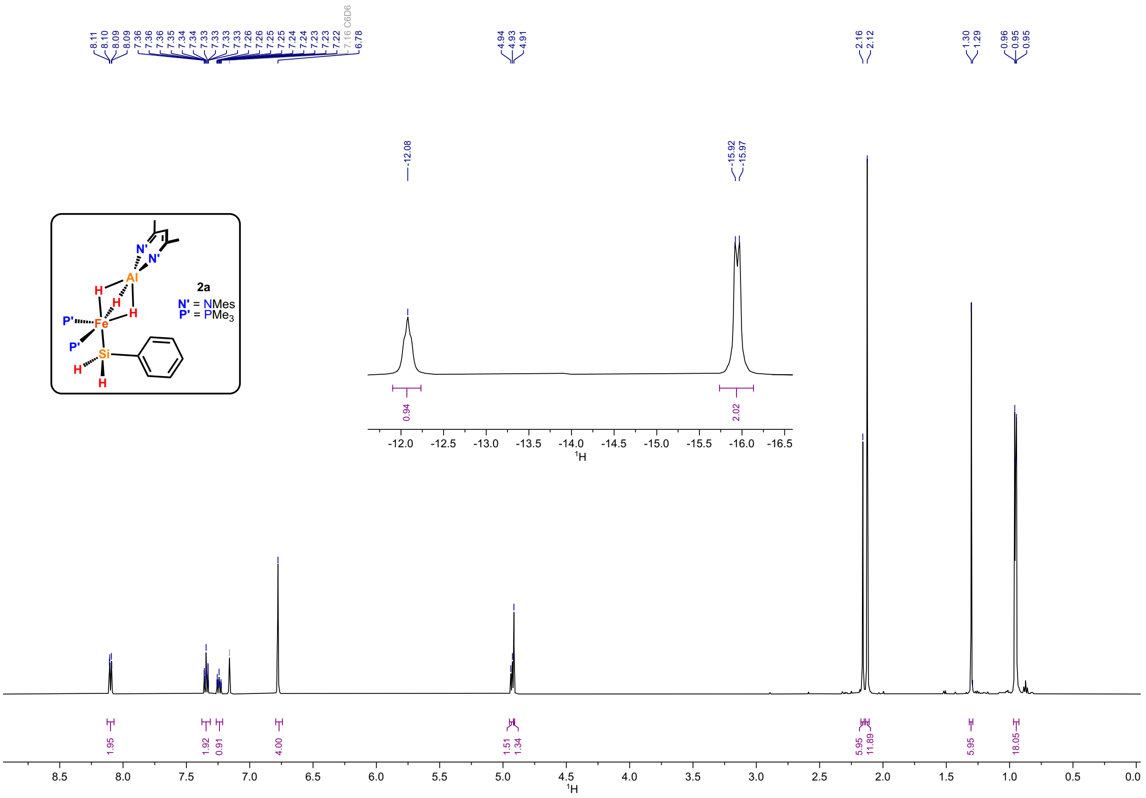

Figure S18 ^{1}H NMR spectrum of **2a** (C_6D_6, 298 K, 500 MHz).

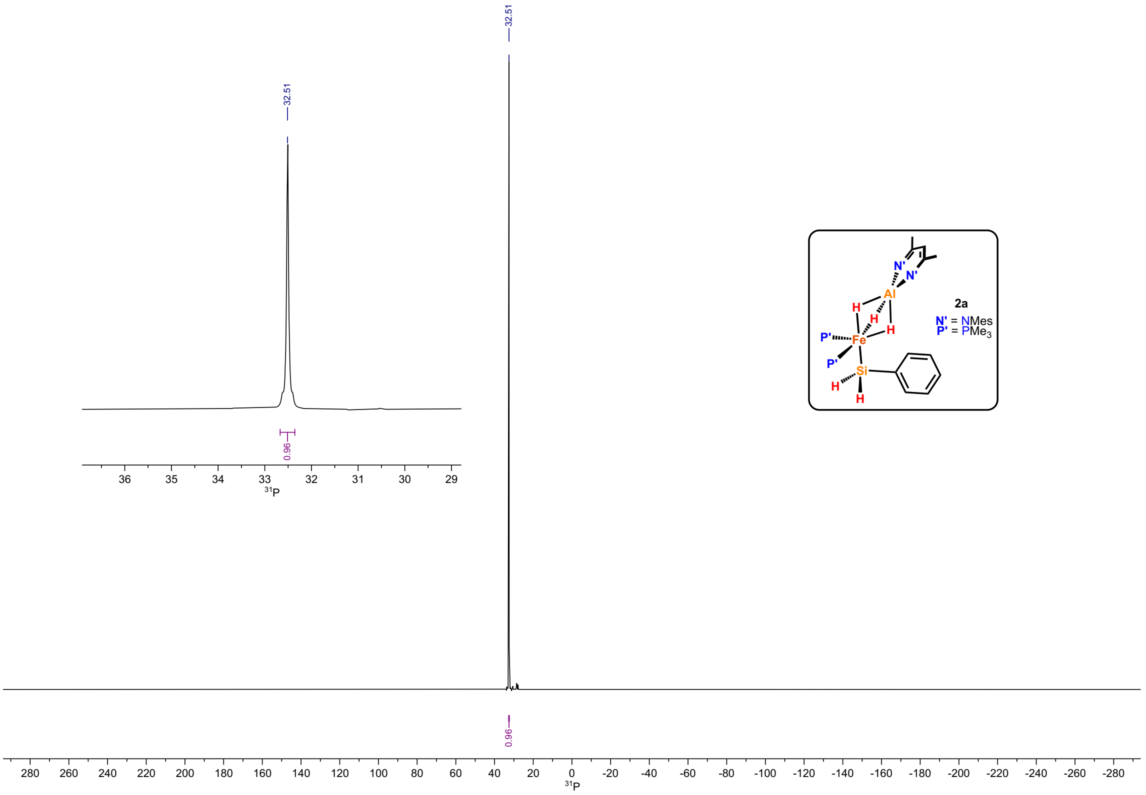

Figure S19 $^{31}P\{^{1}H\}$ NMR spectrum of **2a** (C_6D_6, 298 K, 202 MHz).

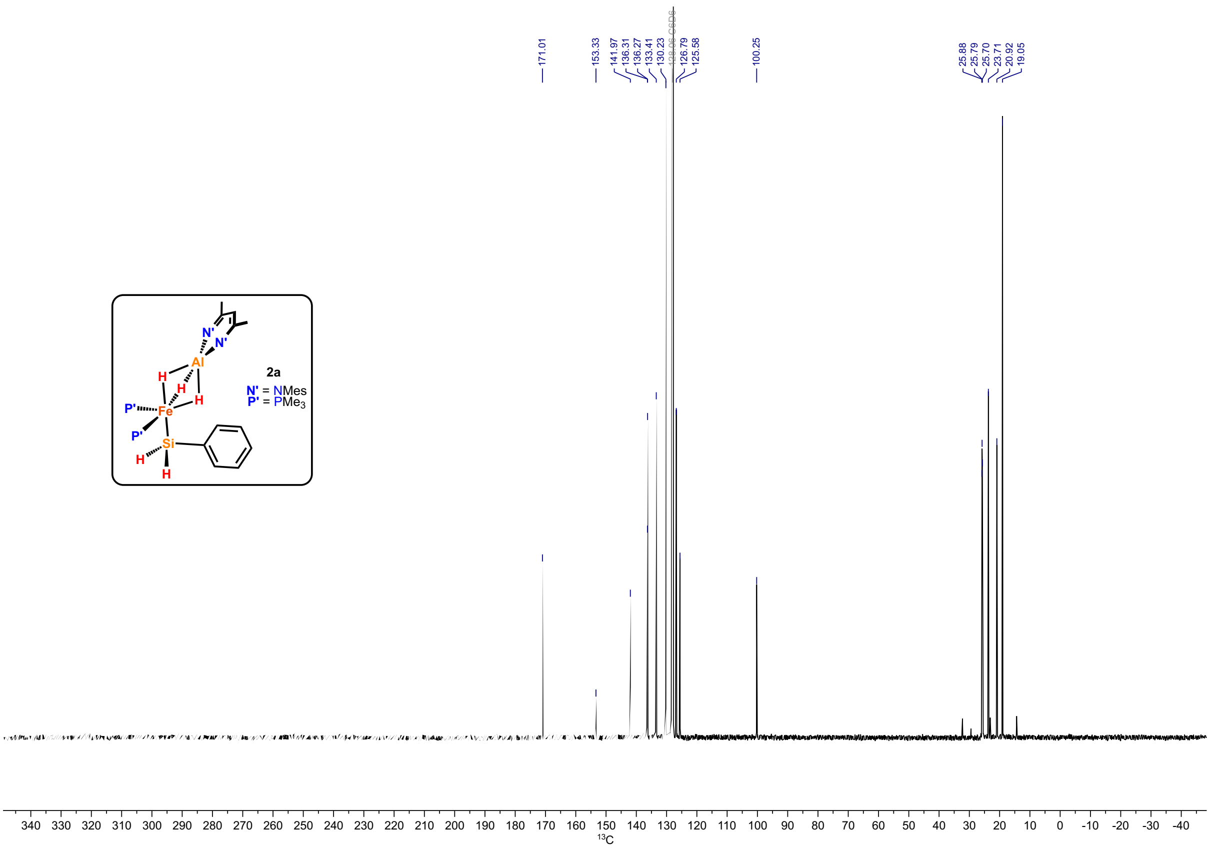

Figure S20 $^{13}C\{^{1}H\}$ NMR spectrum of **2a** (C_6D_6, 298 K, 126 MHz).

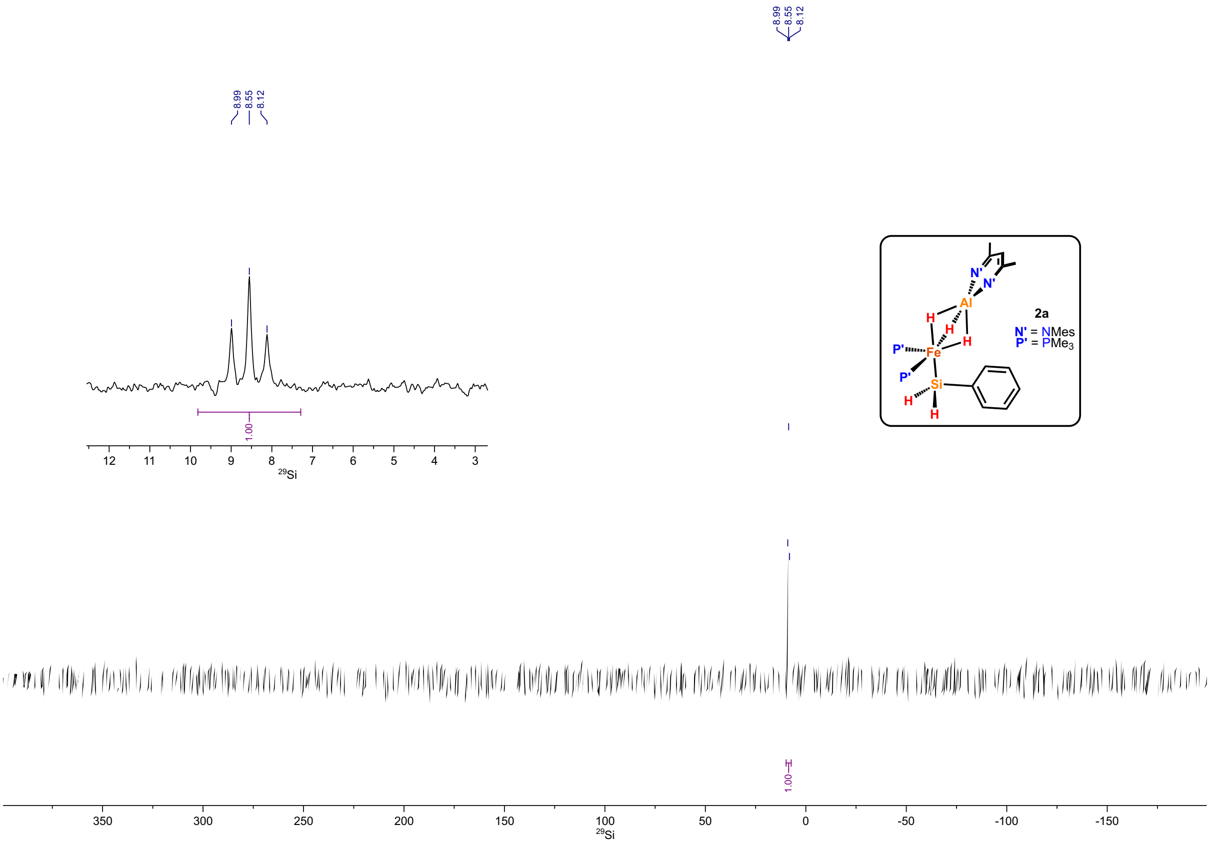

Figure S21 $^{29}Si\{^{1}H\}$ NMR spectrum of **2a** (C_6D_6, 298 K, 99 MHz, inverse gated).

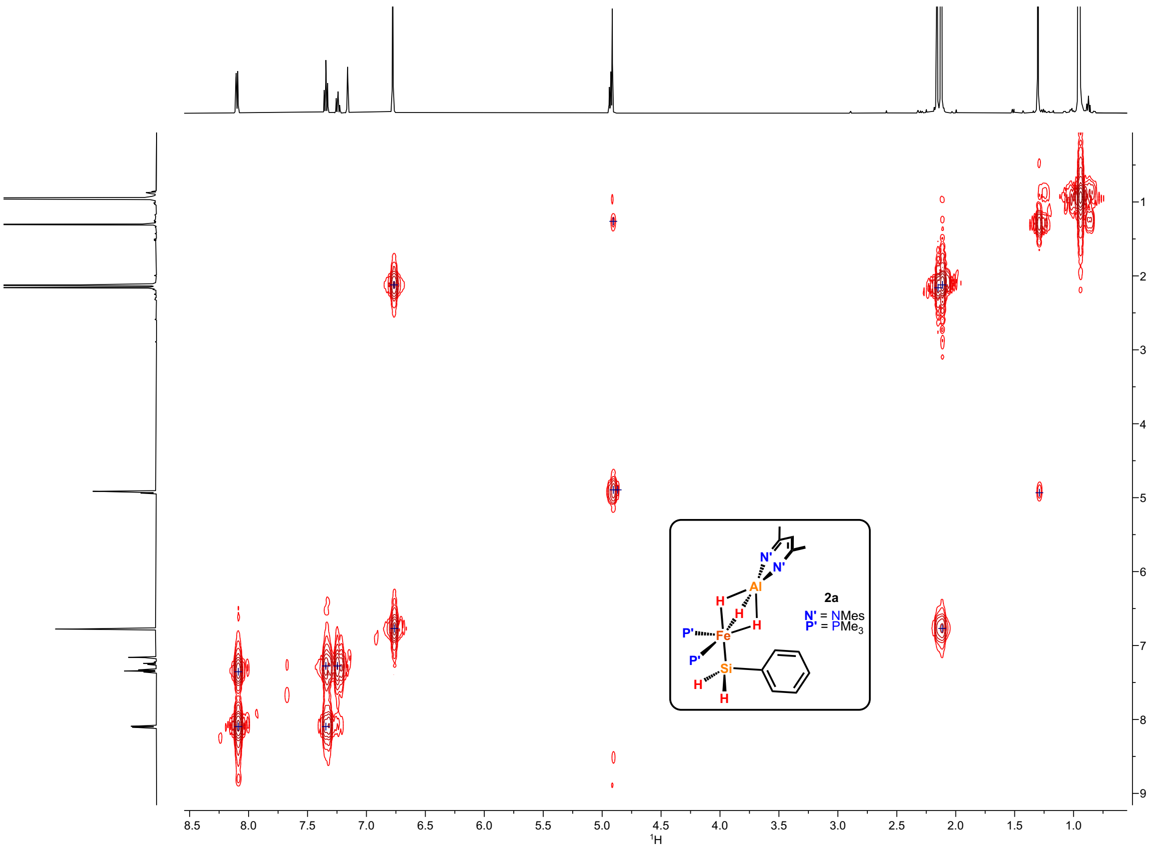

Figure S22 ^{1}H-^{1}H COSY NMR spectrum of **2a** (C_6D_6, 298 K, 500 MHz).

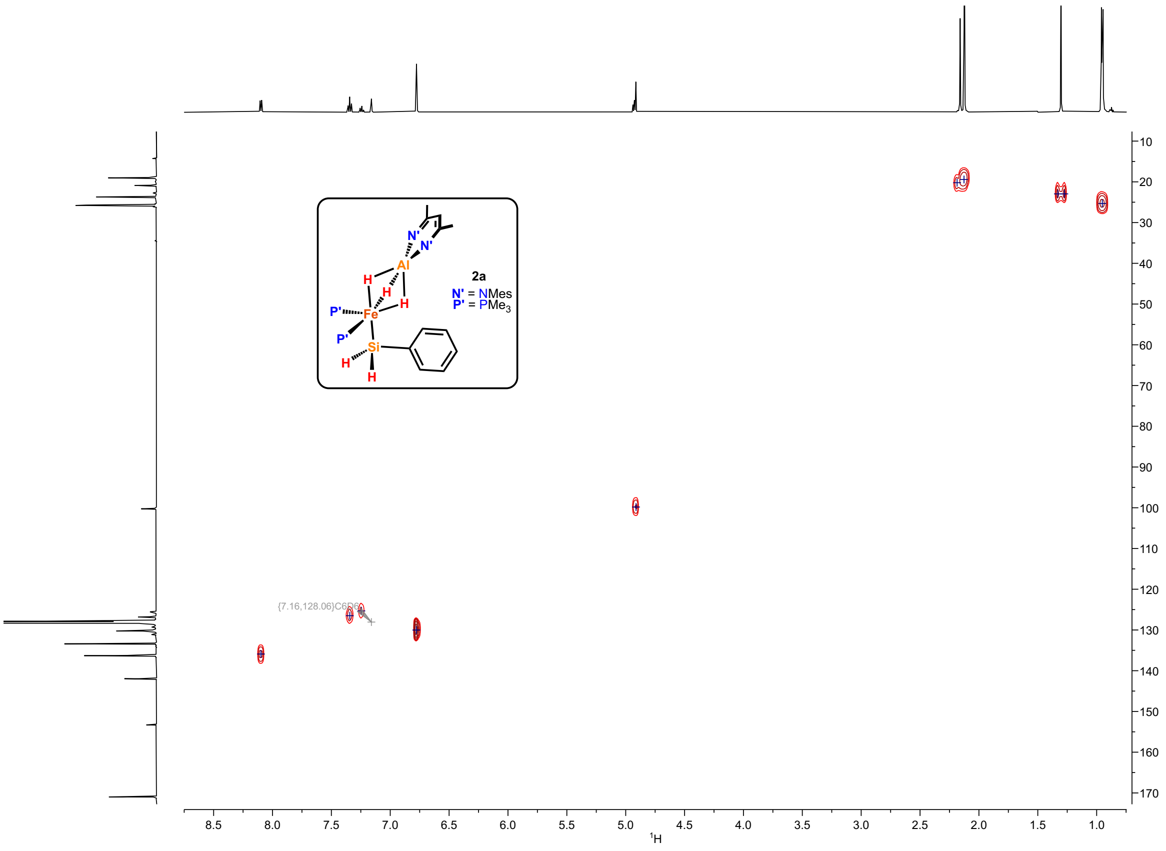

Figure S23 ^{1}H-^{13}C HSQC NMR spectrum of **2a** (C_6D_6, 298 K, 500 MHz).

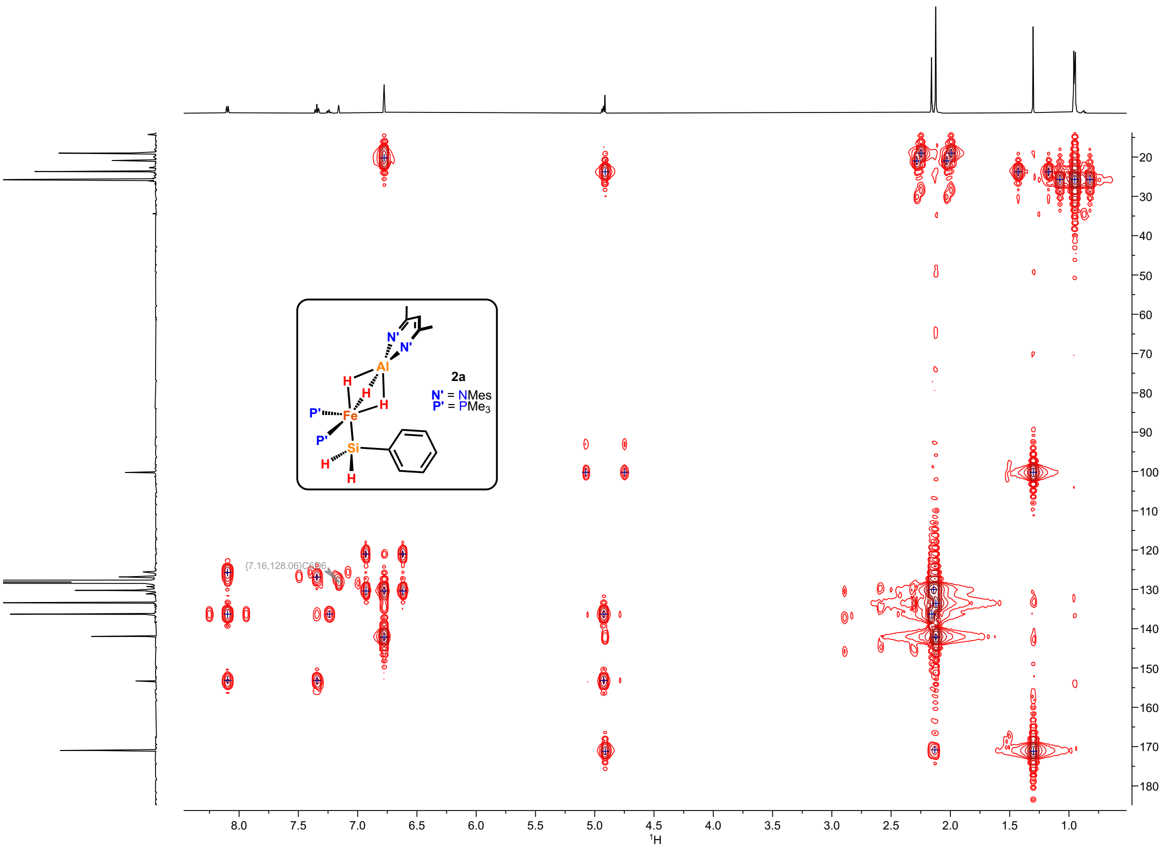

Figure S24 ^{1}H-^{13}C HMBC NMR spectrum of **2a** (C_6D_6, 298 K, 500 MHz).

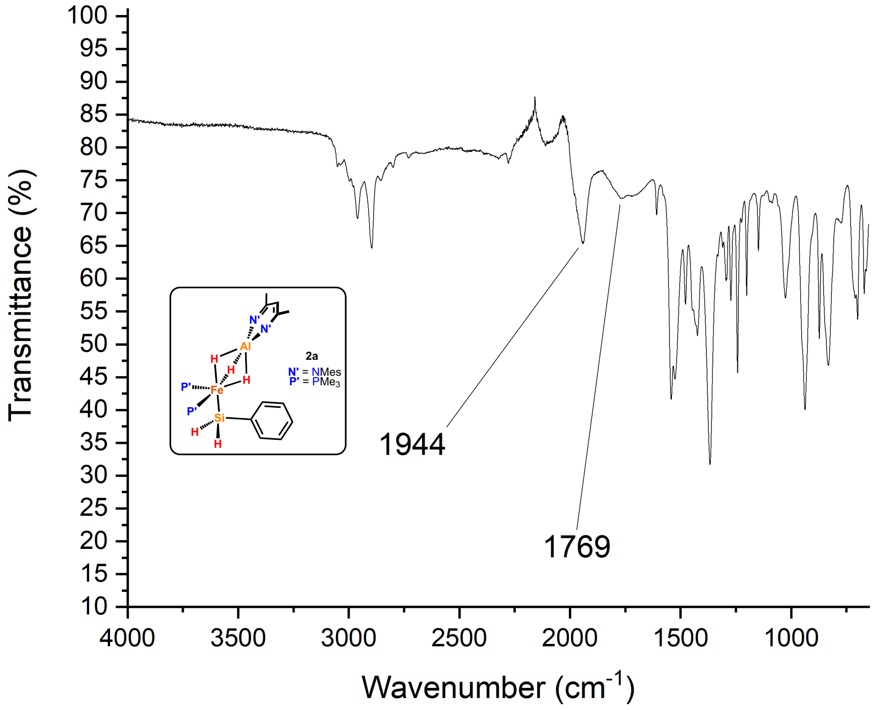

Figure S25 FT-IR spectrum of **2a** (thin film, 298 K, ATR).

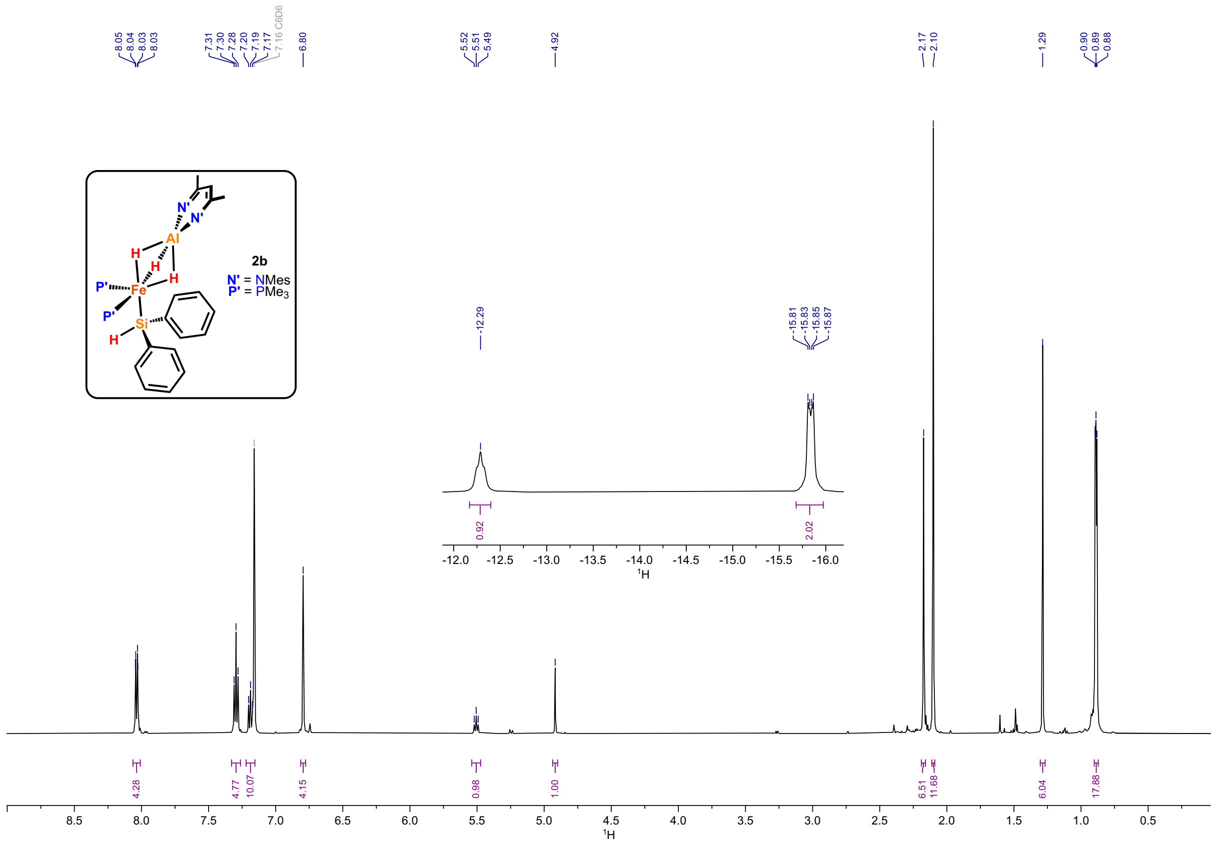

Figure S26 ^{1}H NMR spectrum of **2b** (C_6D_6, 298 K, 500 MHz).

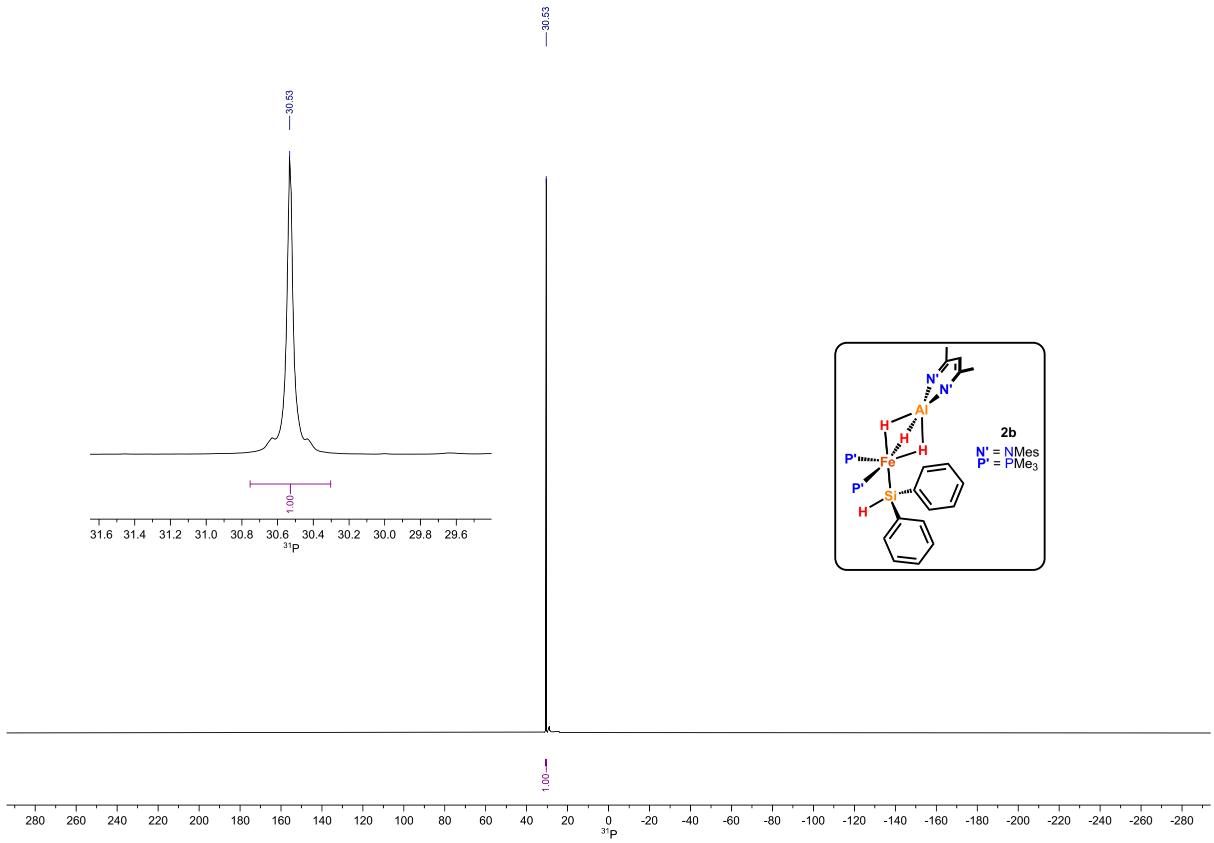

Figure S27 ^{31}P{^{1}H} NMR spectrum of **2b** (C_6D_6, 298 K, 202 MHz).

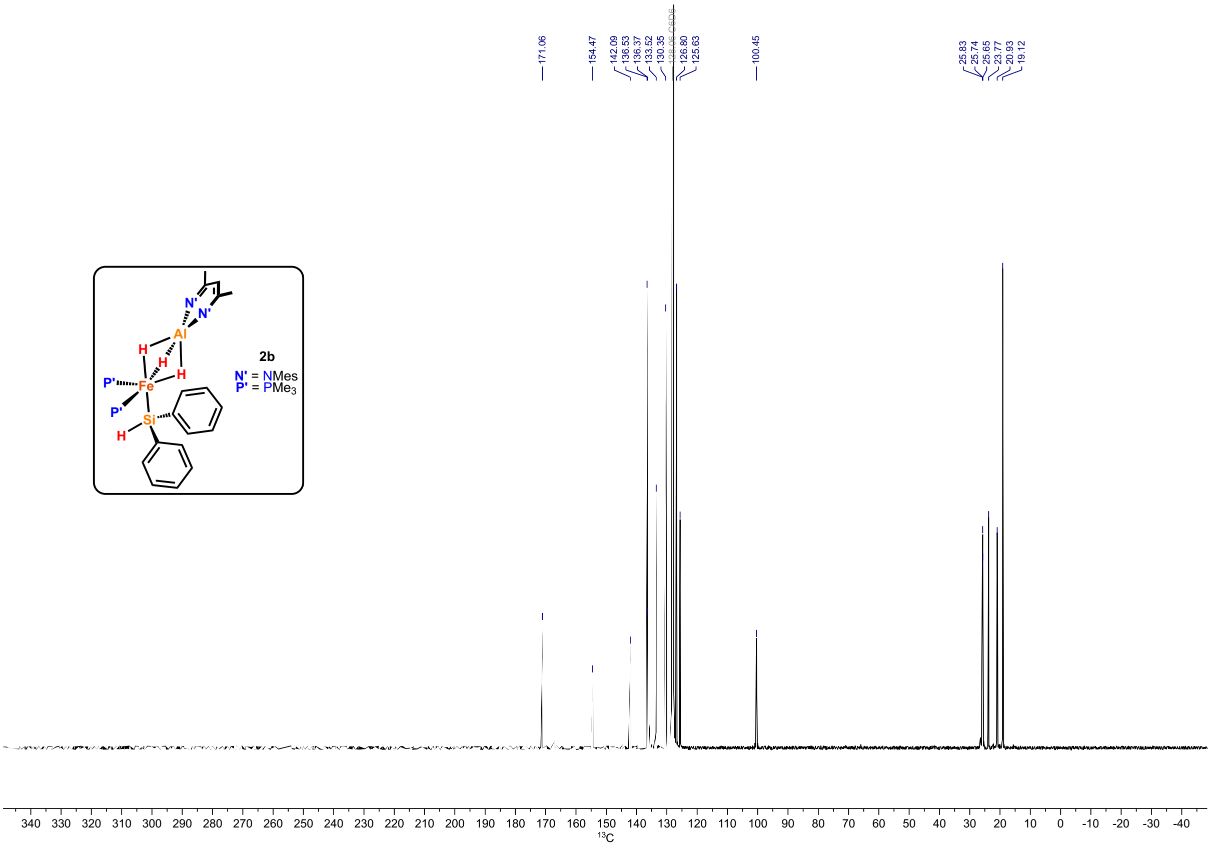

Figure S28 $^{13}C\{^1H\}$ NMR spectrum of **2b** (C_6D_6, 298 K, 126 MHz).

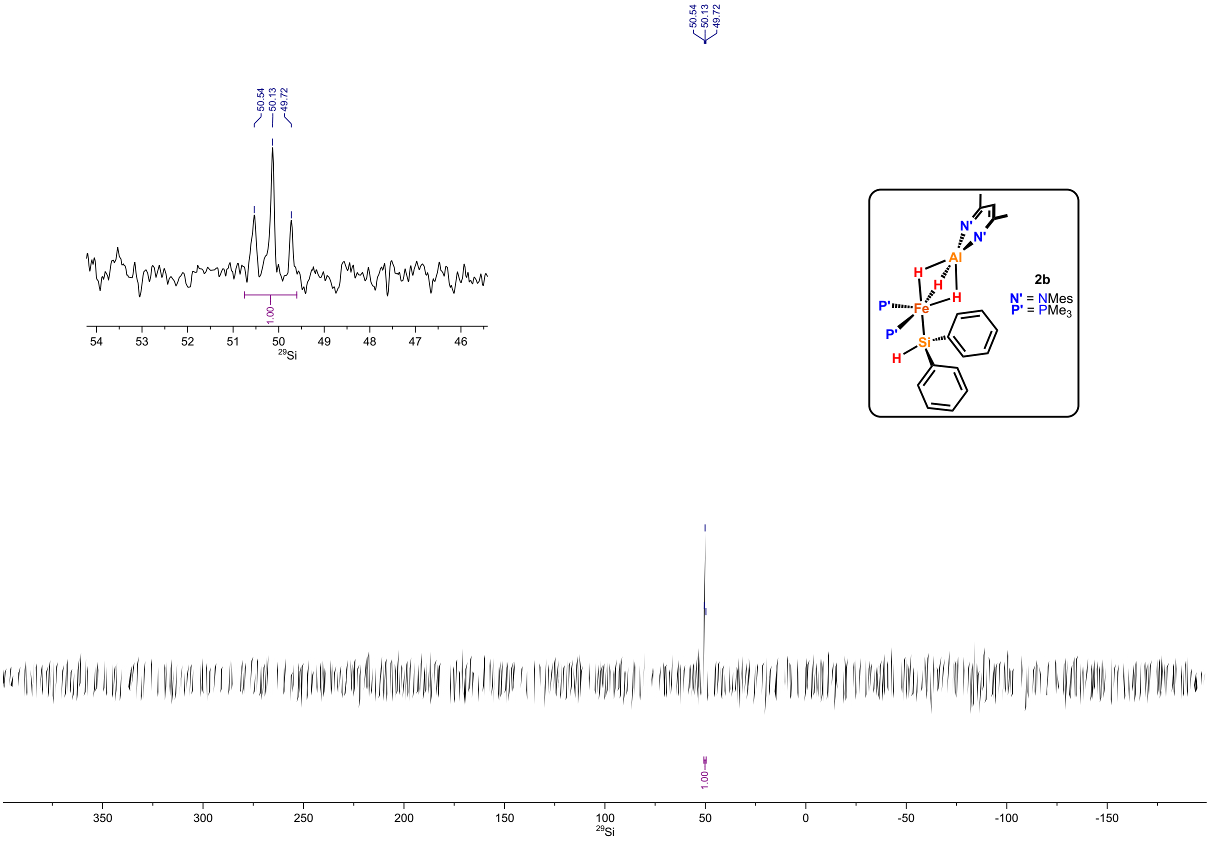

Figure S29 $^{29}Si\{^1H\}$ NMR spectrum of **2b** (C_6D_6, 298 K, 99 MHz, inverse gated).

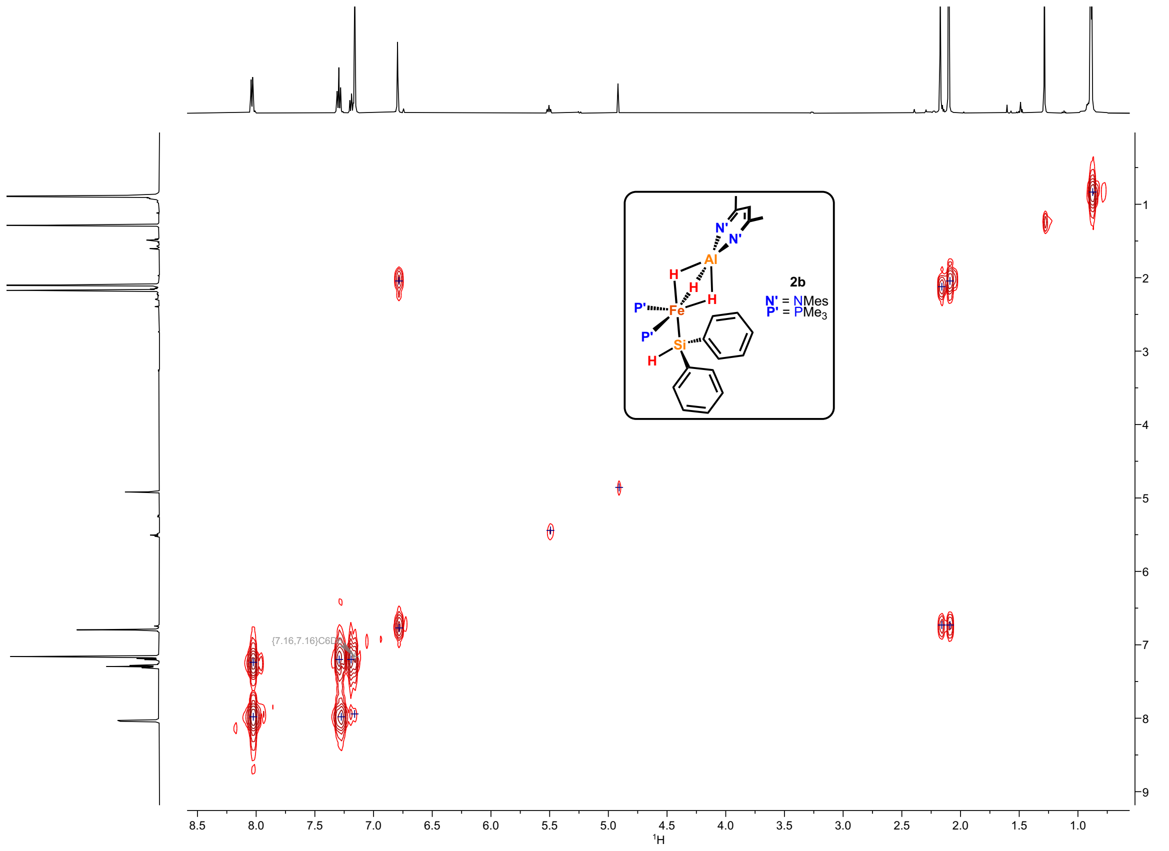

Figure S30 ^{1}H-^{1}H COSY NMR spectrum of **2b** (C_6D_6, 298 K, 500 MHz).

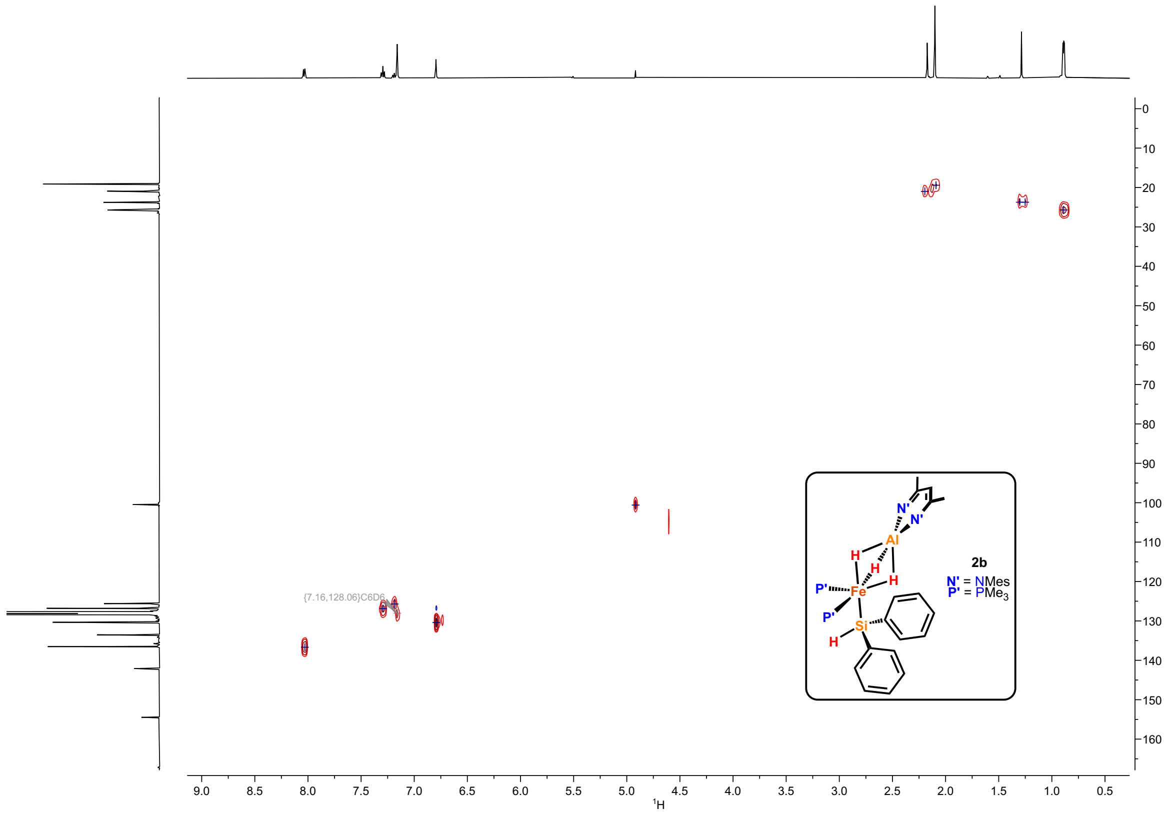

Figure S31 ^{1}H-^{13}C HSQC NMR spectrum of **2b** (C_6D_6, 298 K, 500 MHz).

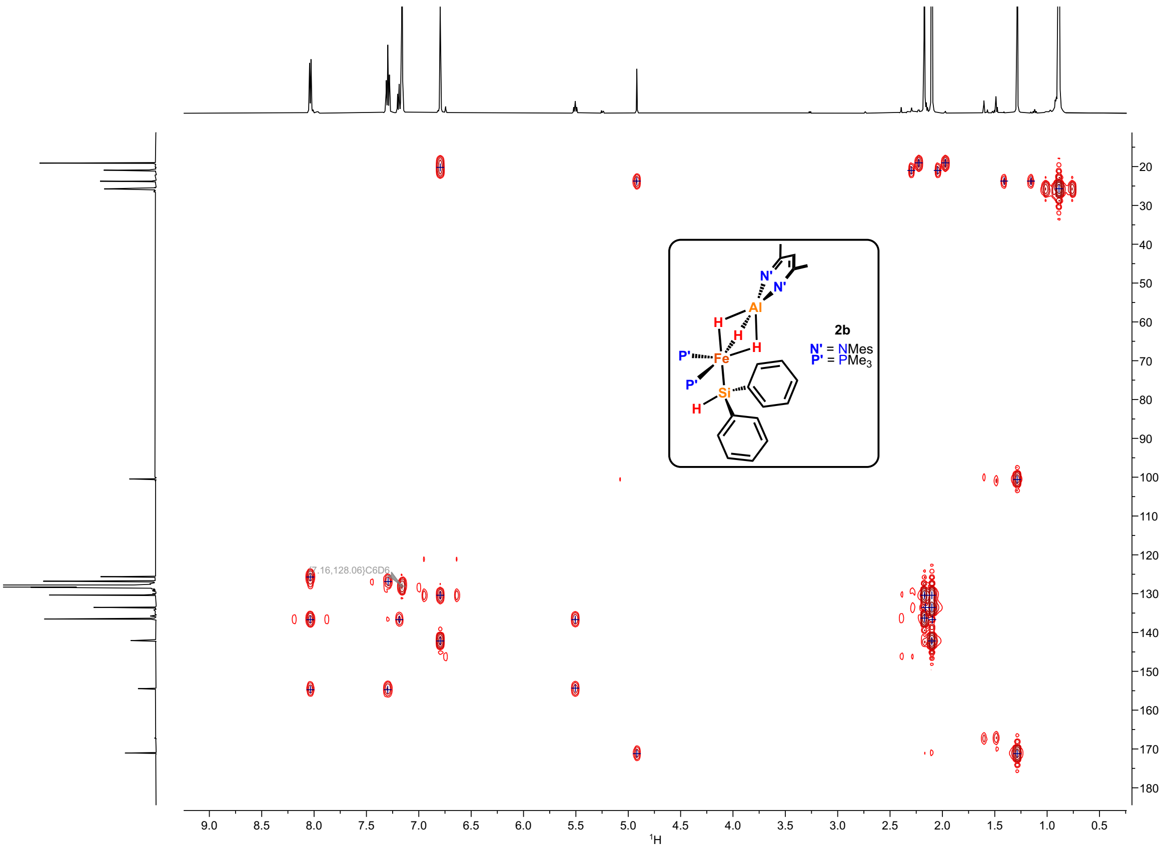

Figure S32 ^{1}H-^{13}C HMBC NMR spectrum of **2b** (C_6D_6, 298 K, 500 MHz).

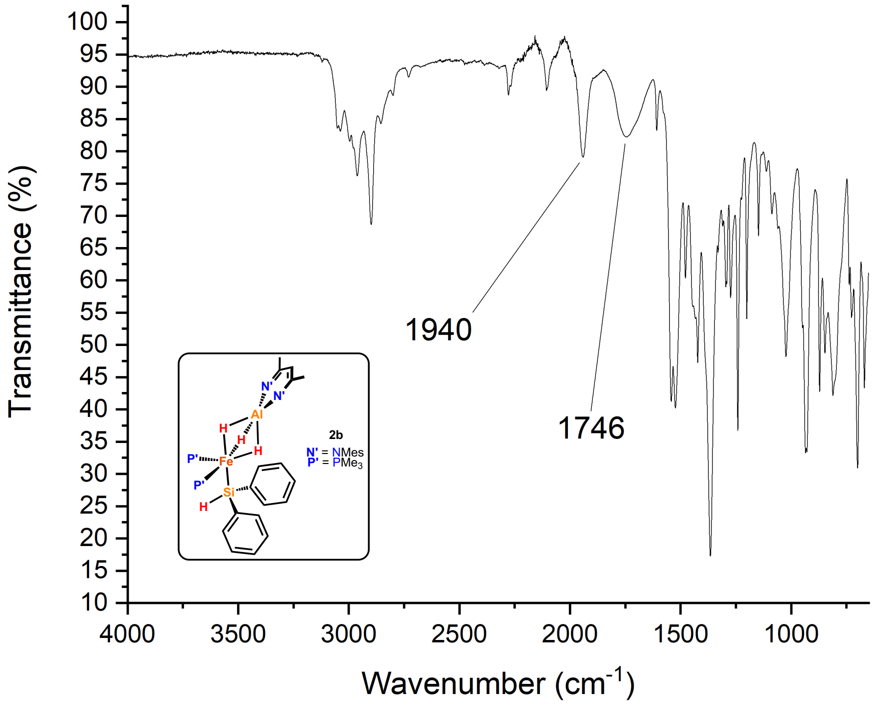

Figure S33 FT-IR spectrum of **2b** (thin film, 298 K, ATR).

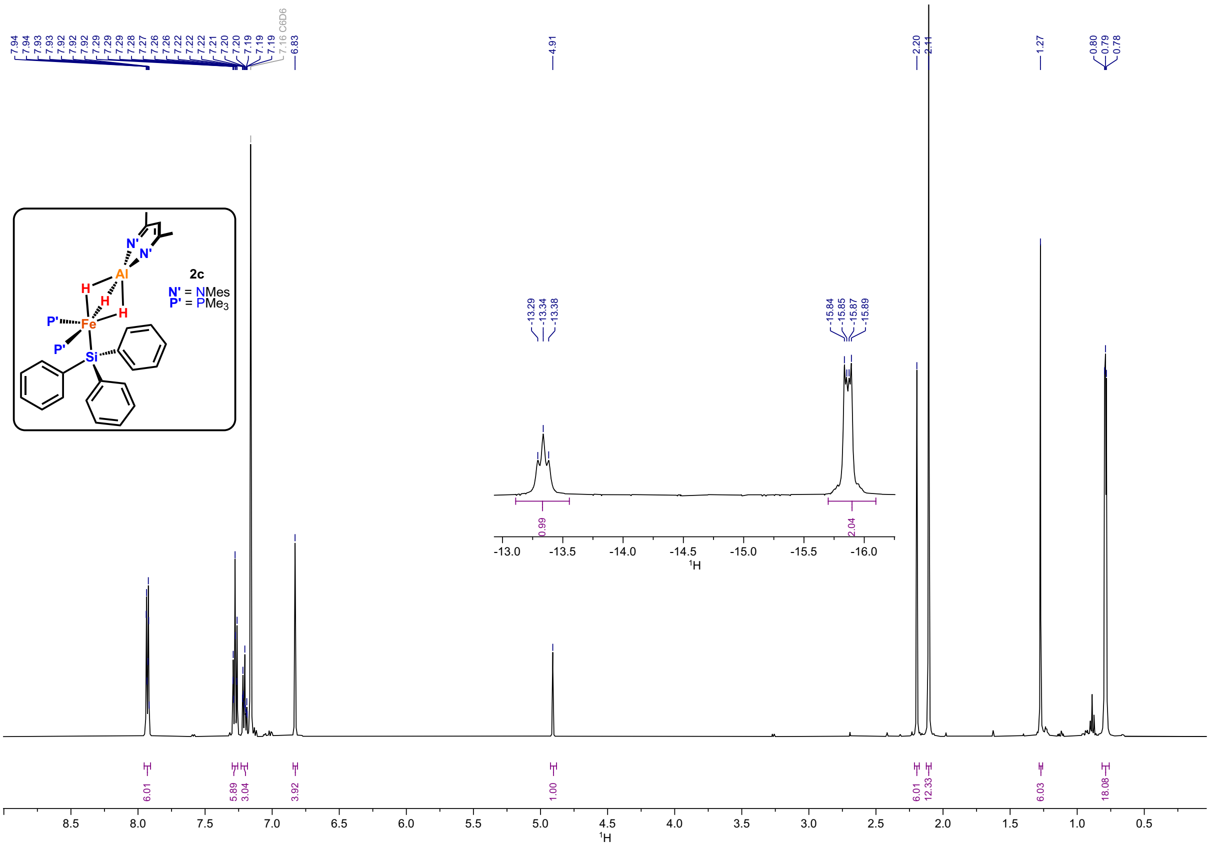

Figure S34 ^{1}H NMR spectrum of **2c** (C_6D_6, 298 K, 500 MHz).

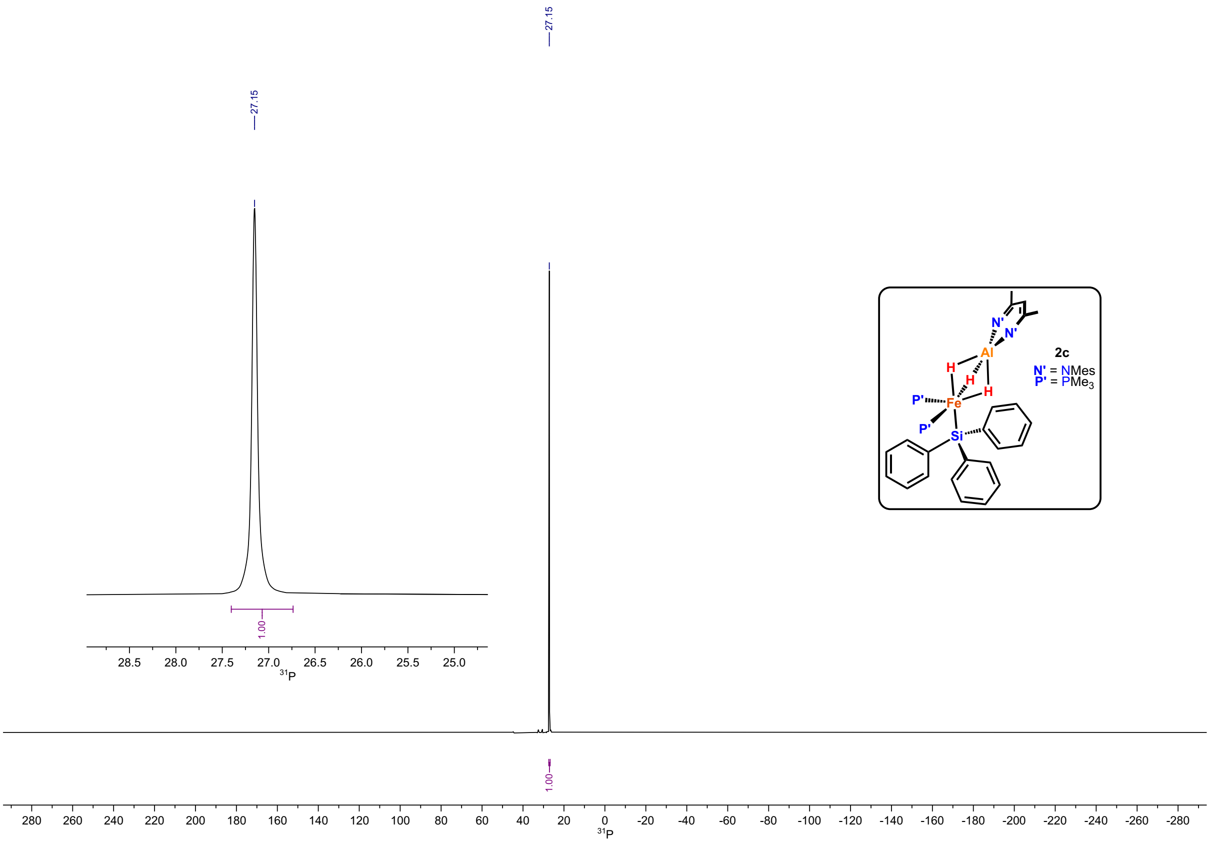

Figure S35 $^{31}P\{^{1}H\}$ NMR spectrum of **2c** (C_6D_6, 298 K, 202 MHz).

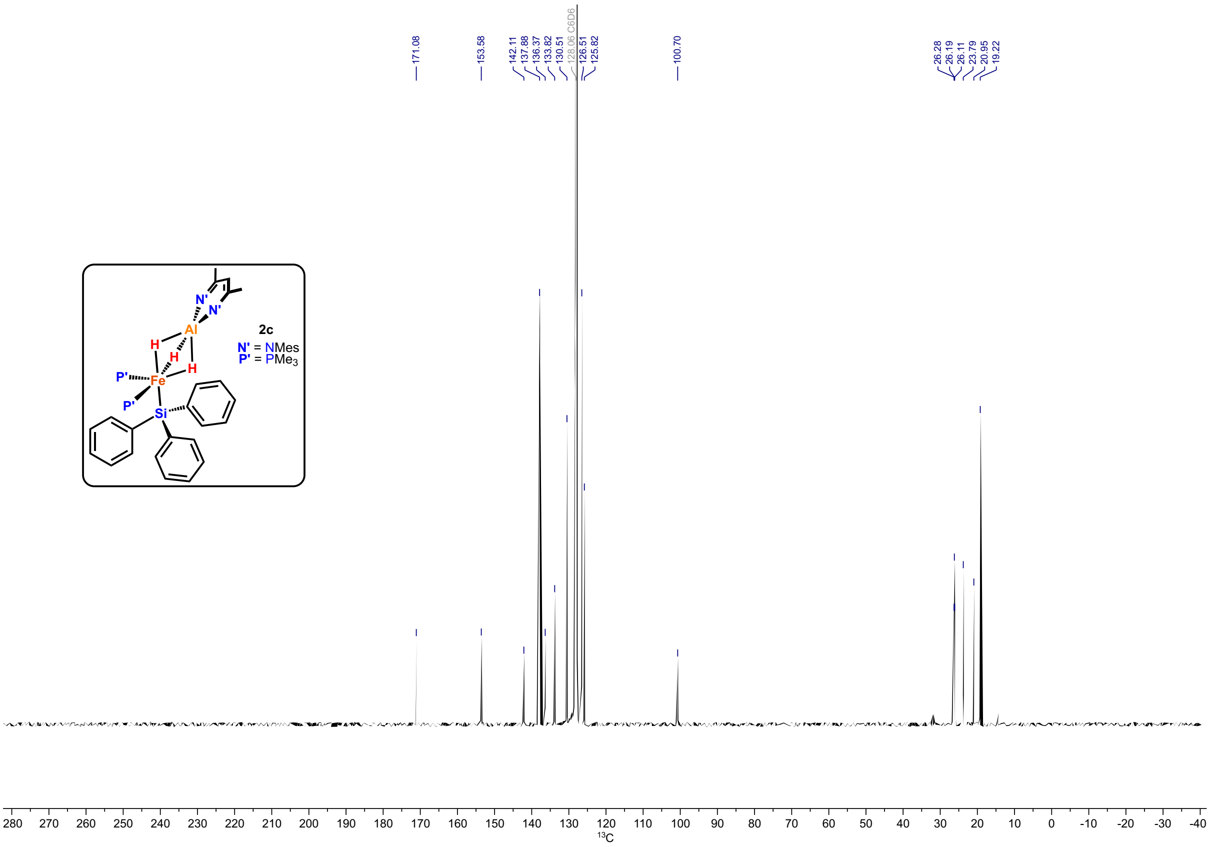

Figure S36 $^{13}C\{^{1}H\}$ NMR spectrum of **2c** (C_6D_6, 298 K, 126 MHz).

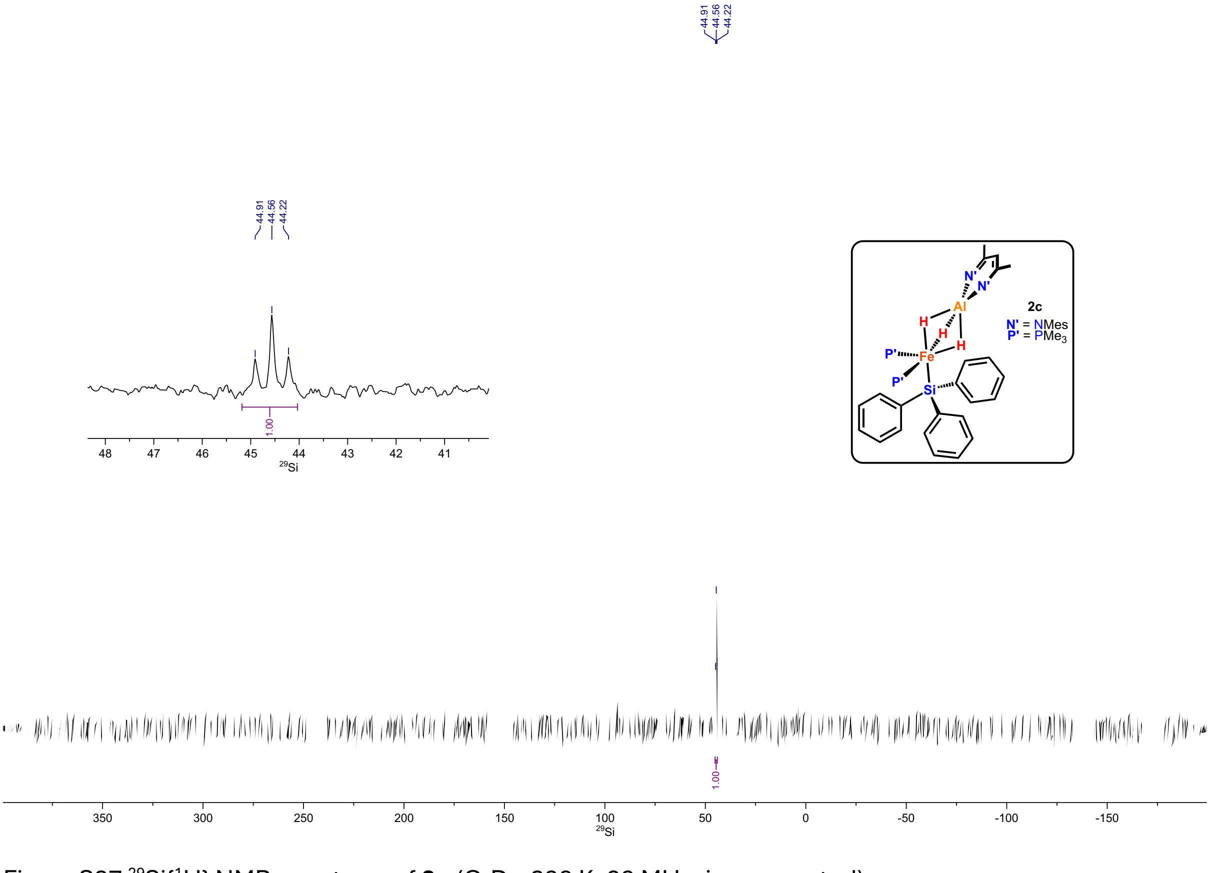

Figure S37 $^{29}Si\{^{1}H\}$ NMR spectrum of **2c** (C_6D_6, 298 K, 99 MHz, inverse gated).

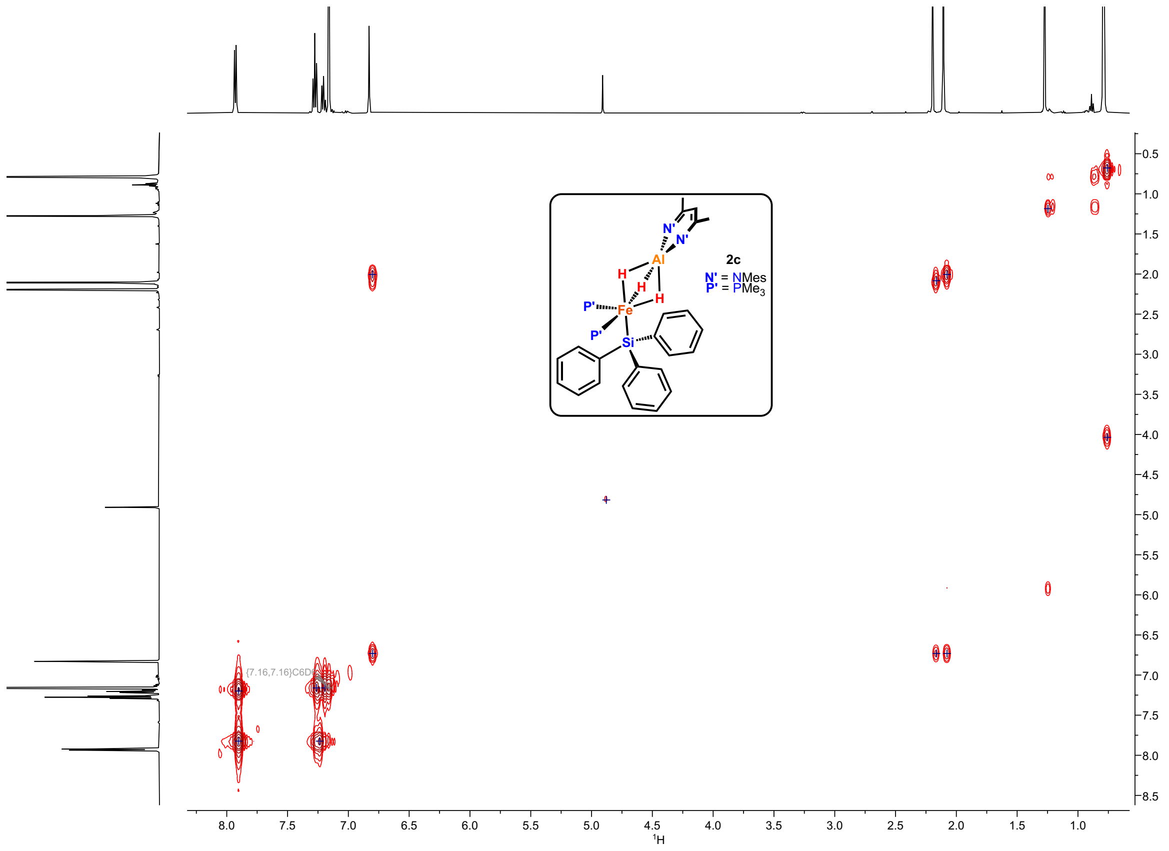

Figure S38 ^{1}H-^{1}H COSY NMR spectrum of **2c** (C_6D_6, 298 K, 500 MHz).

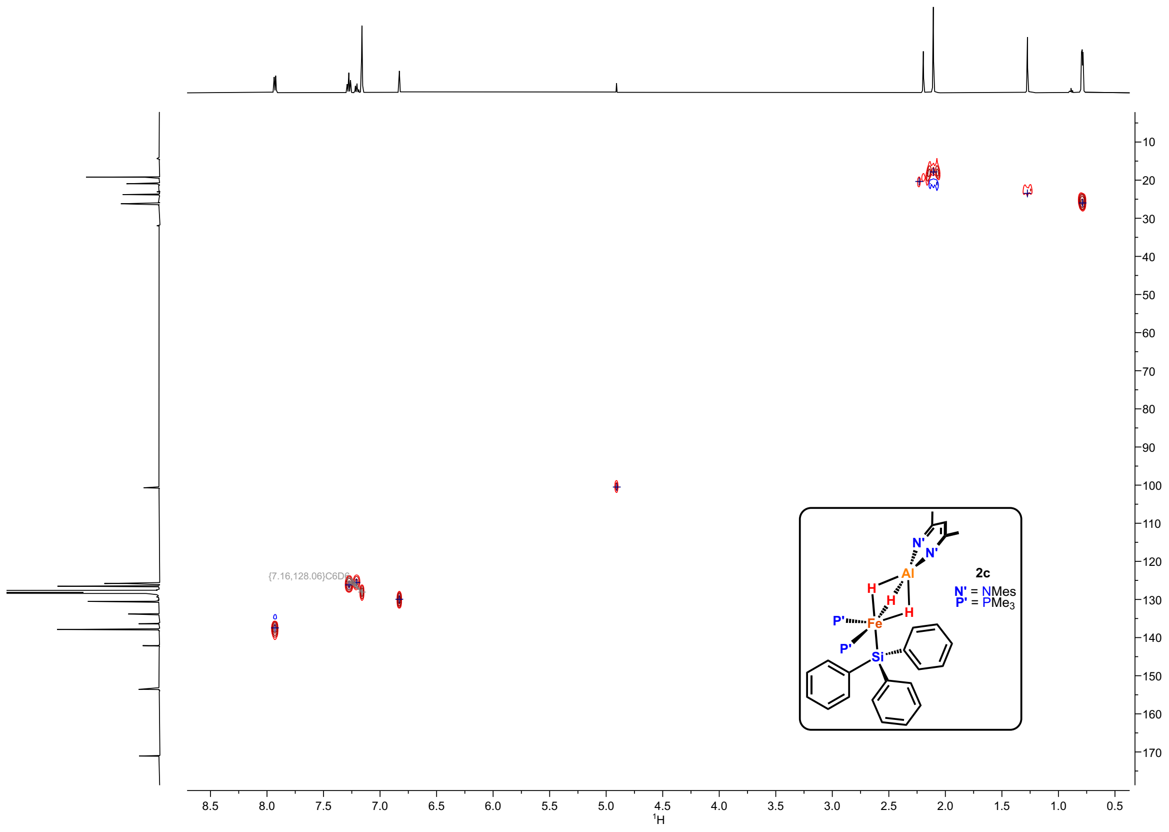

Figure S39 ^{1}H-^{13}C HSQC NMR spectrum of **2c** (C_6D_6, 298 K, 500 MHz).

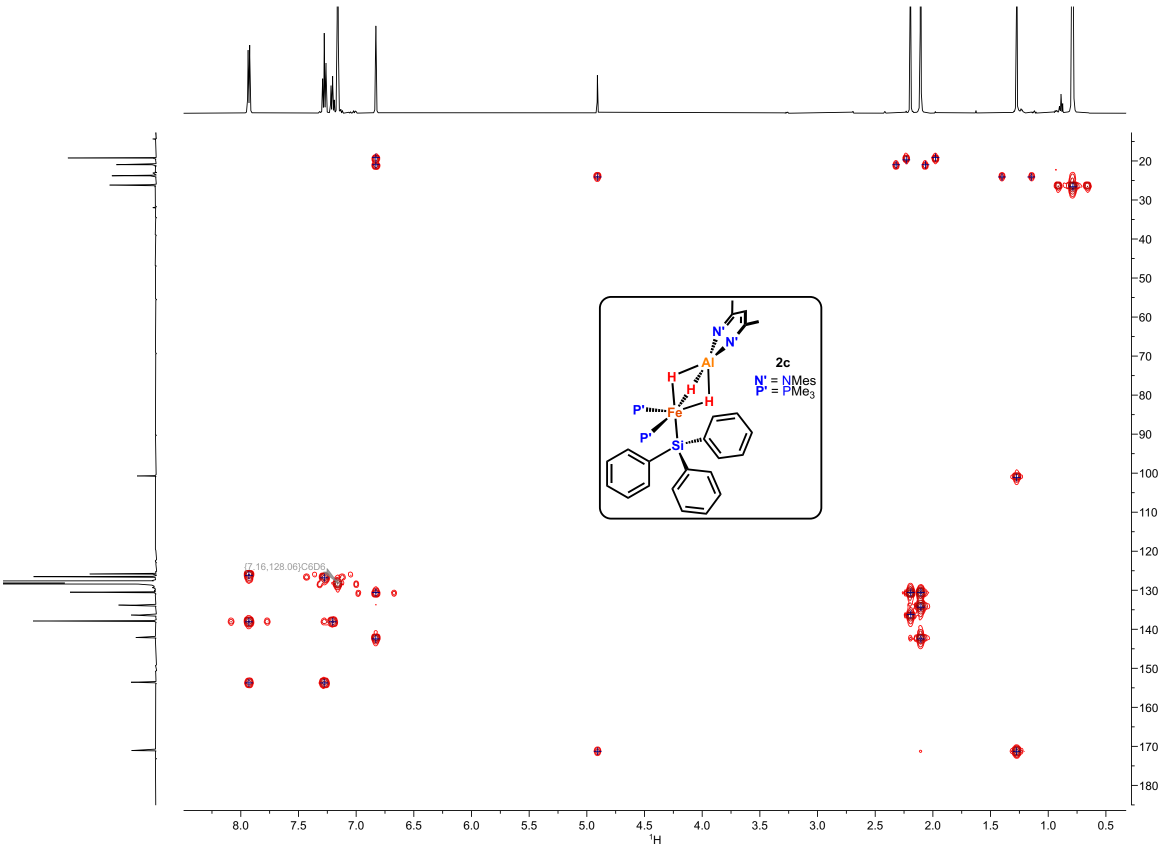

Figure S40 ^{1}H-^{13}C HMBC NMR spectrum of **2c** (C_6D_6, 298 K, 500 MHz).

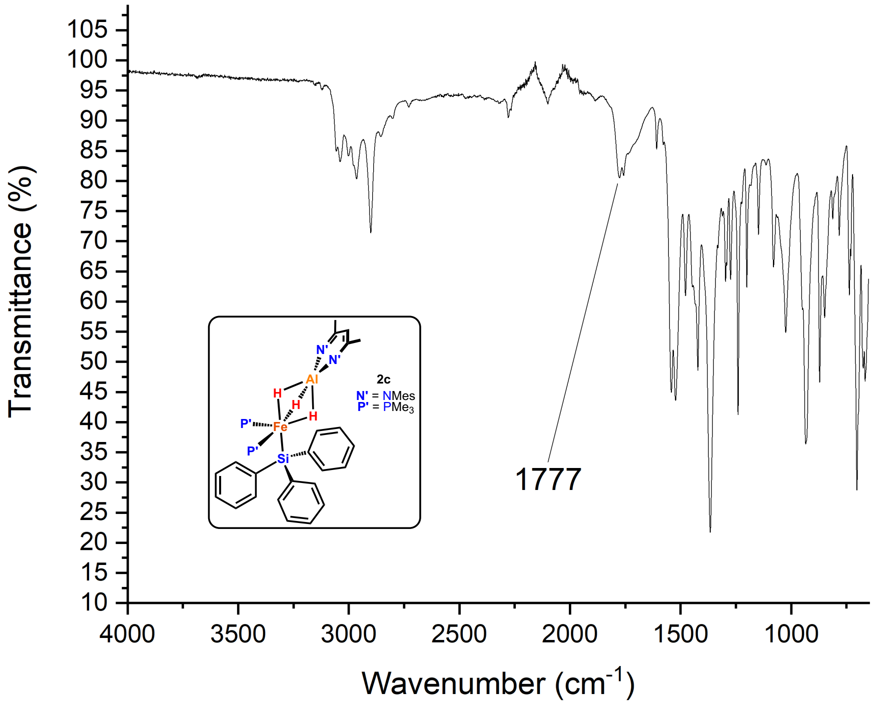

Figure S41 FT-IR spectrum of **2c** (thin film, 298 K, ATR).

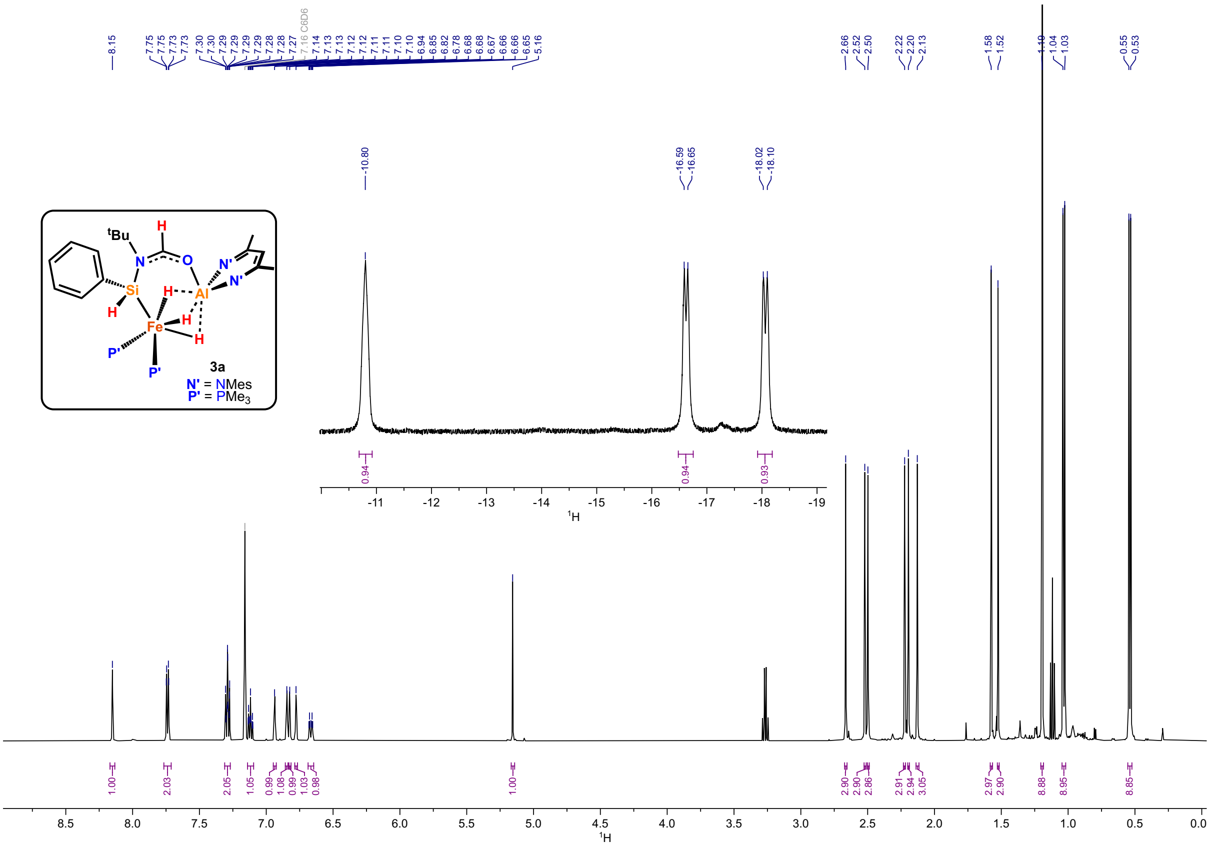

Figure S42 ^{1}H NMR spectrum of **3a** (C_6D_6, 298 K, 500 MHz).

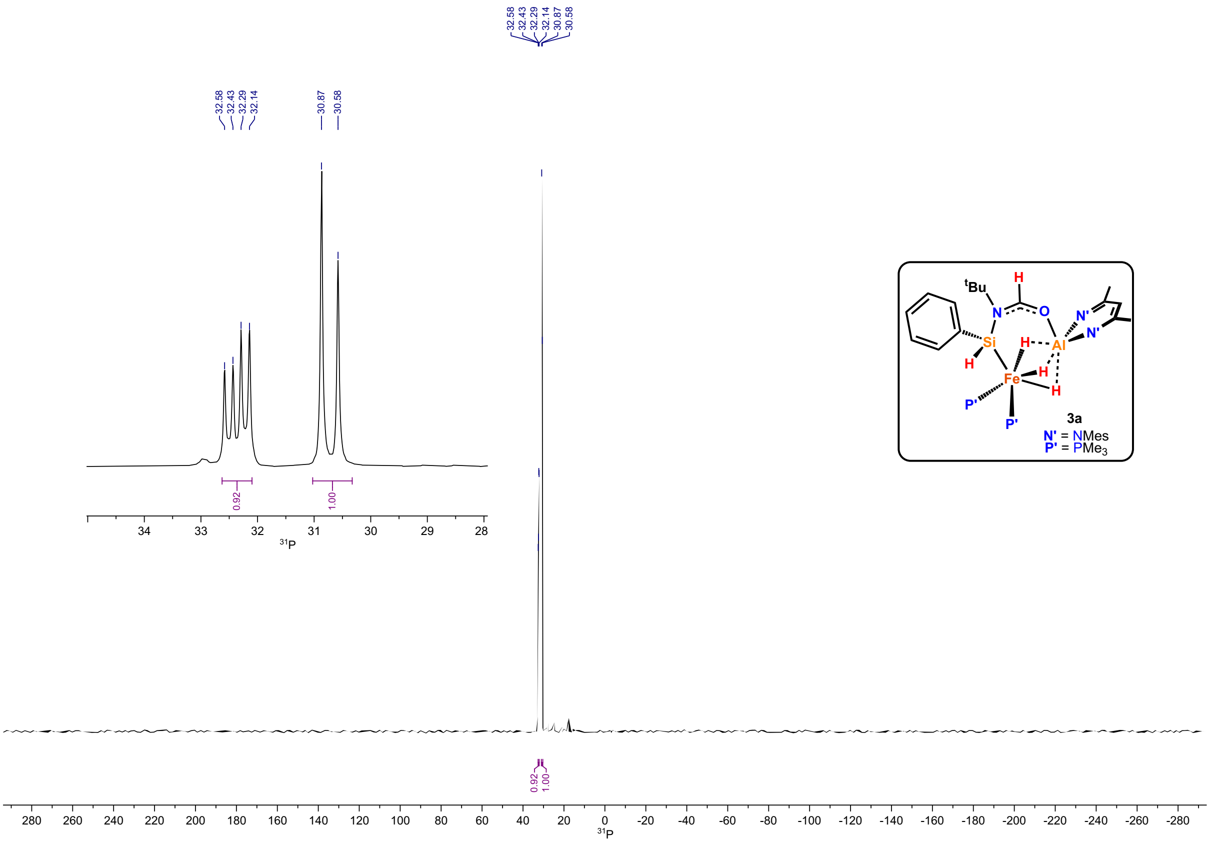

Figure S43 ^{31}P{^{1}H} NMR spectrum of **3a** (C_6D_6, 298 K, 202 MHz).

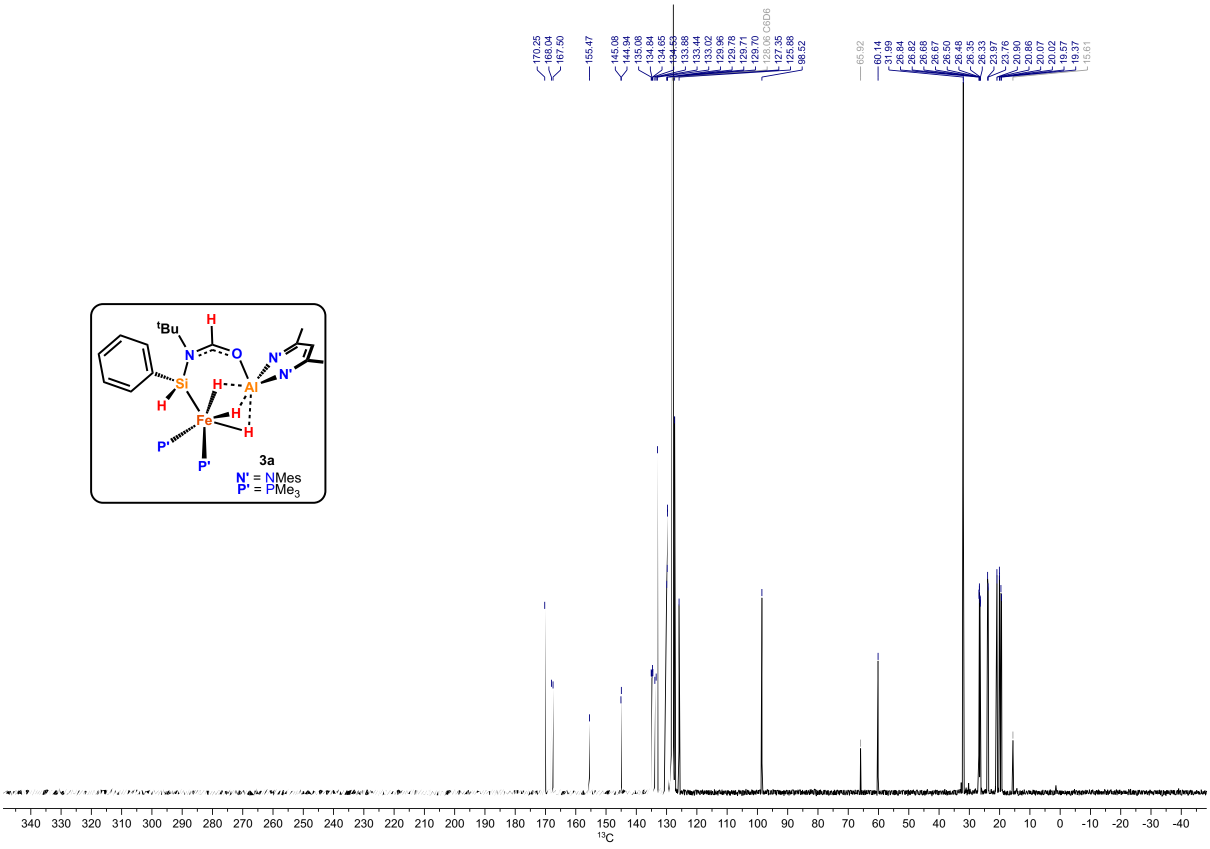

Figure S44 $^{13}C\{^{1}H\}$ NMR spectrum of **3a** (C_6D_6, 298 K, 126 MHz).

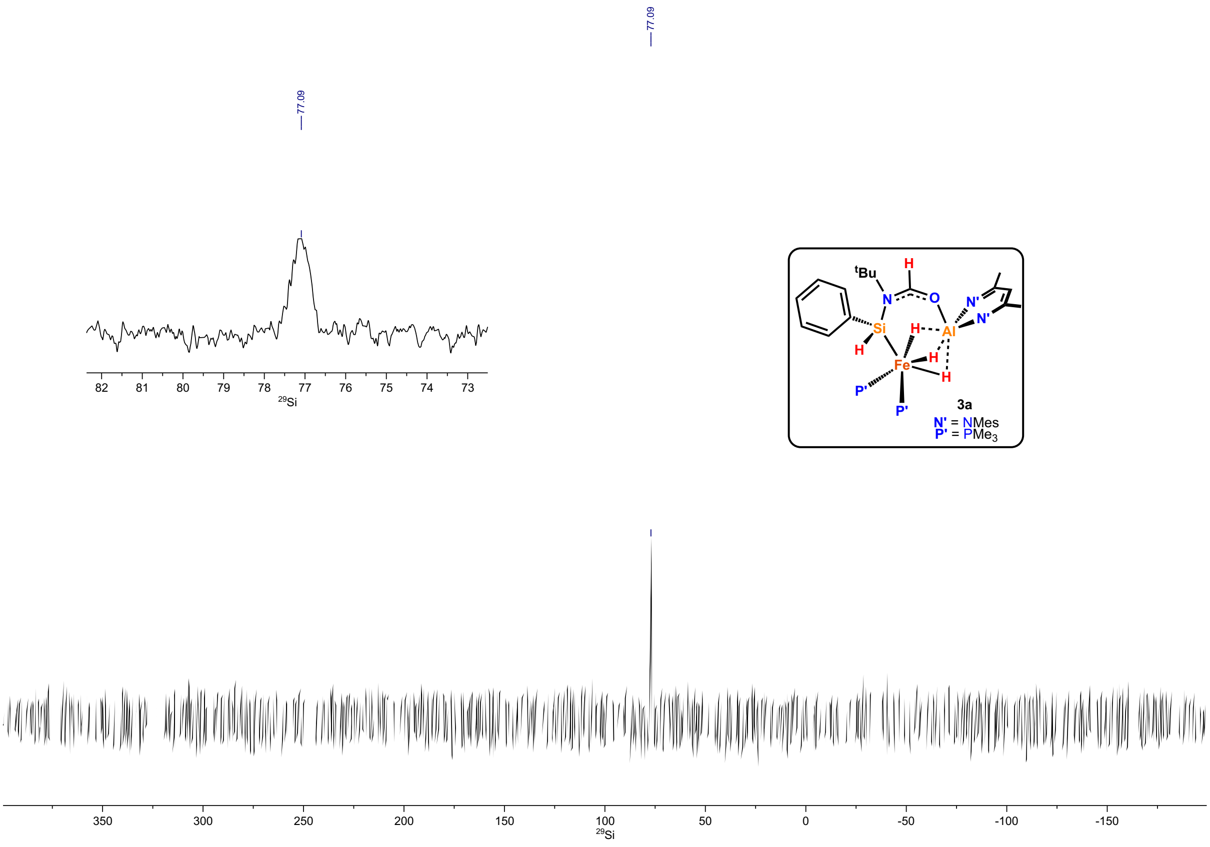

Figure S45 $^{29}Si\{^{1}H$ NMR spectrum of **3a** (C_6D_6, 298 K, 99 MHz, inverse gated).

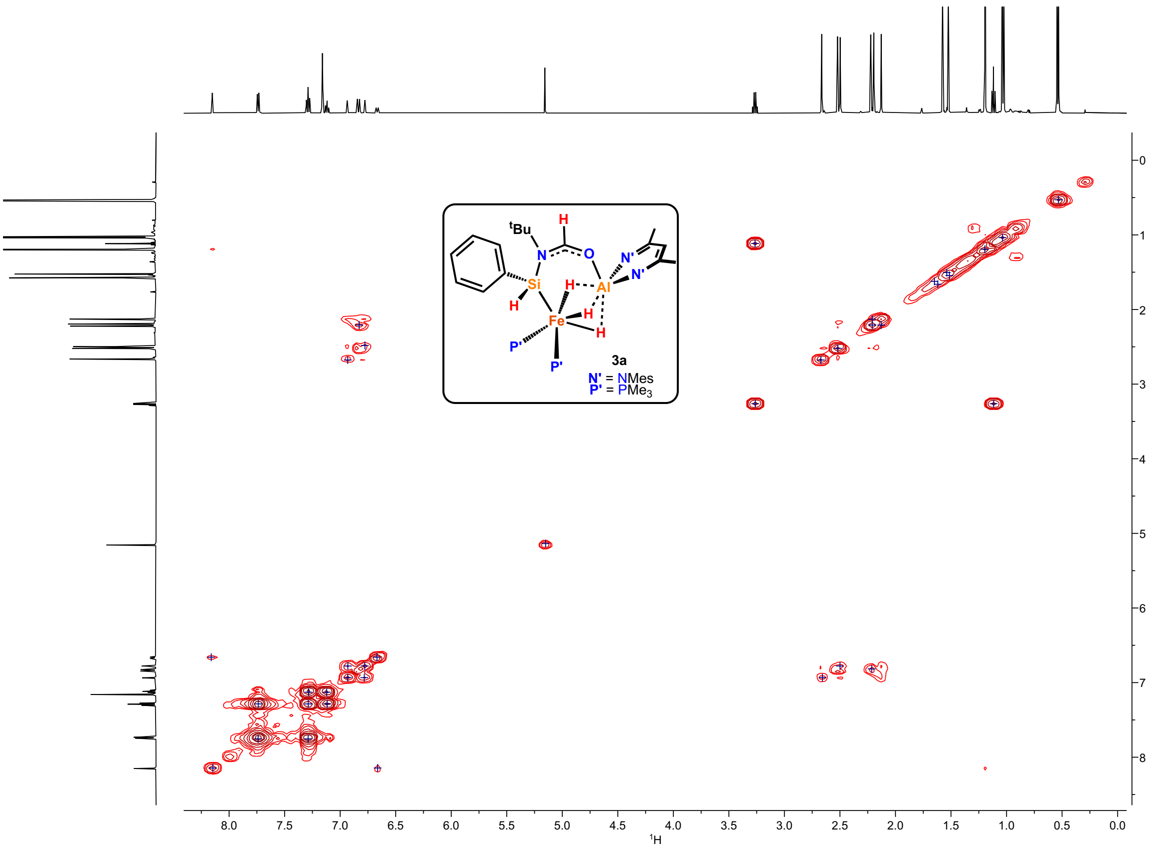

Figure S46 ^{1}H-^{1}H COSY NMR spectrum of **3a** (C_6D_6, 298 K, 500 MHz).

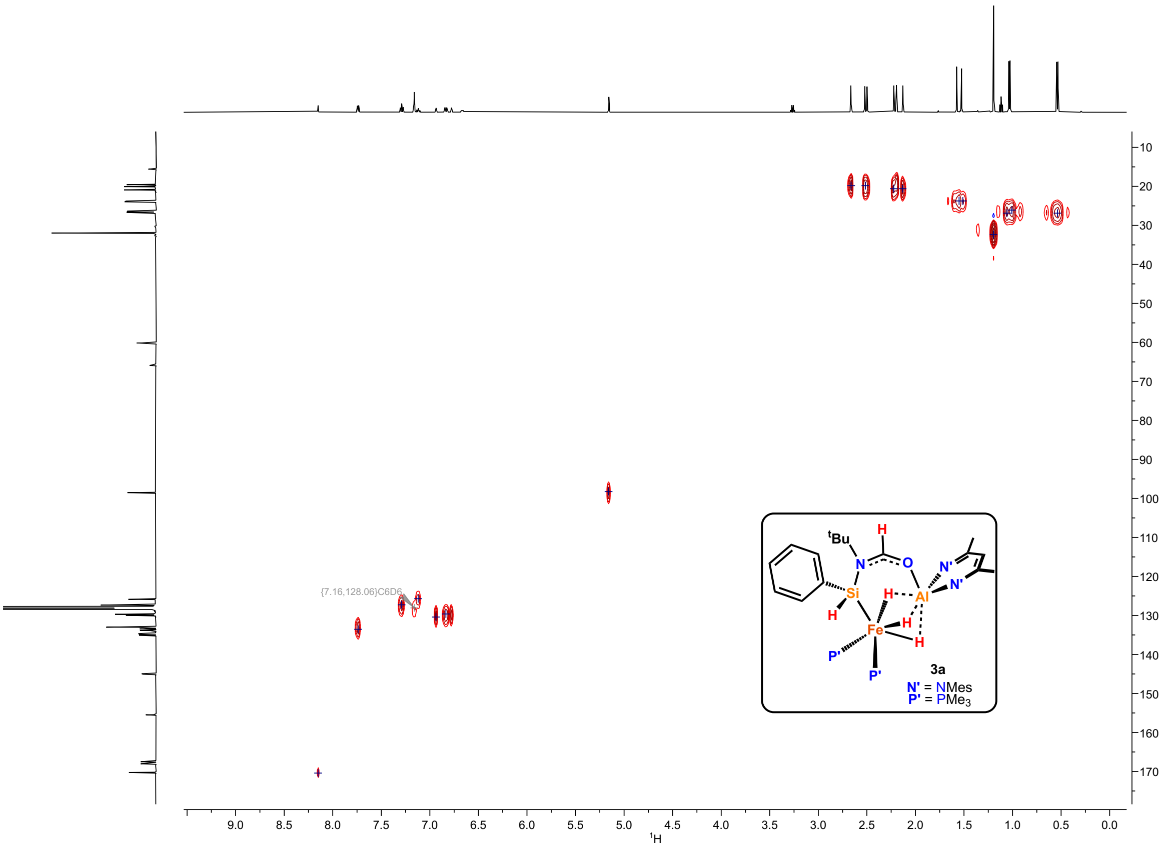

Figure S47 ^{1}H-^{13}C HSQC NMR spectrum of **3a** (C_6D_6, 298 K, 500 MHz).

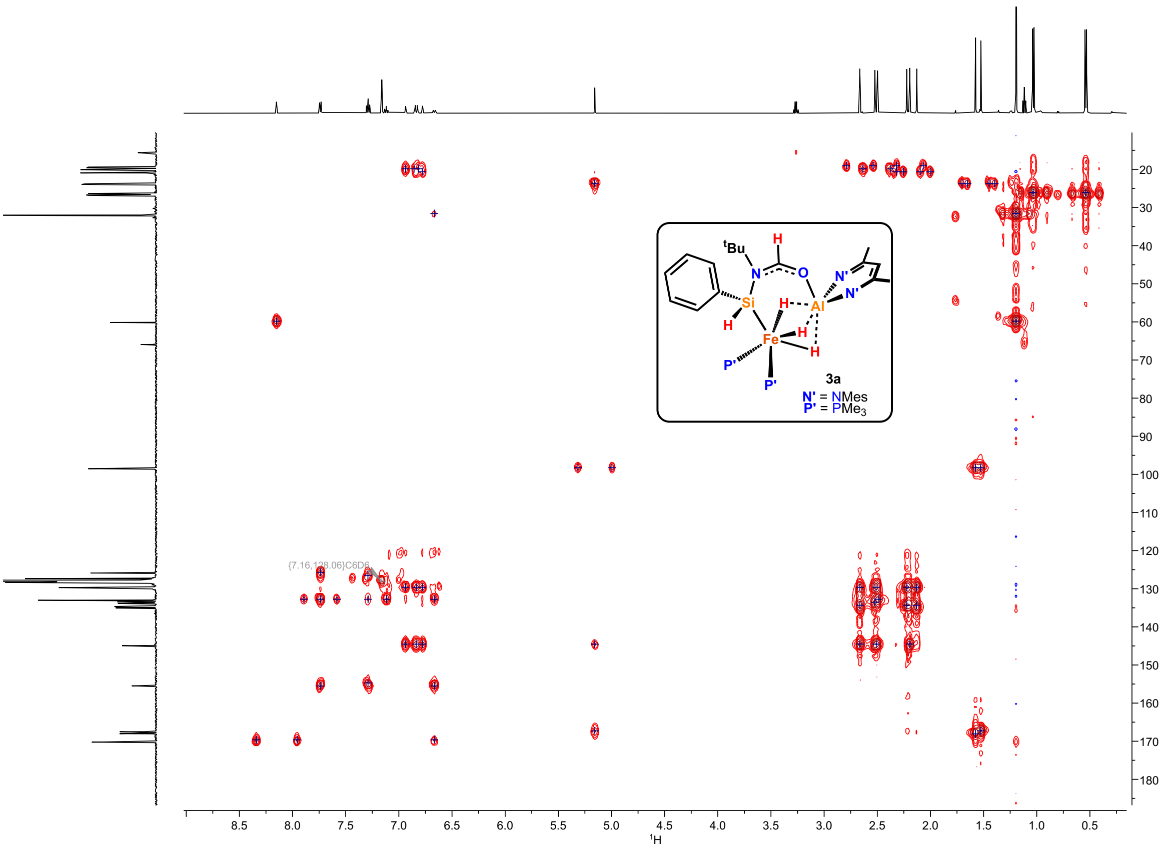

Figure S48 ^{1}H-^{13}C HMBC NMR spectrum of **3a** (C_6D_6, 298 K, 500 MHz).

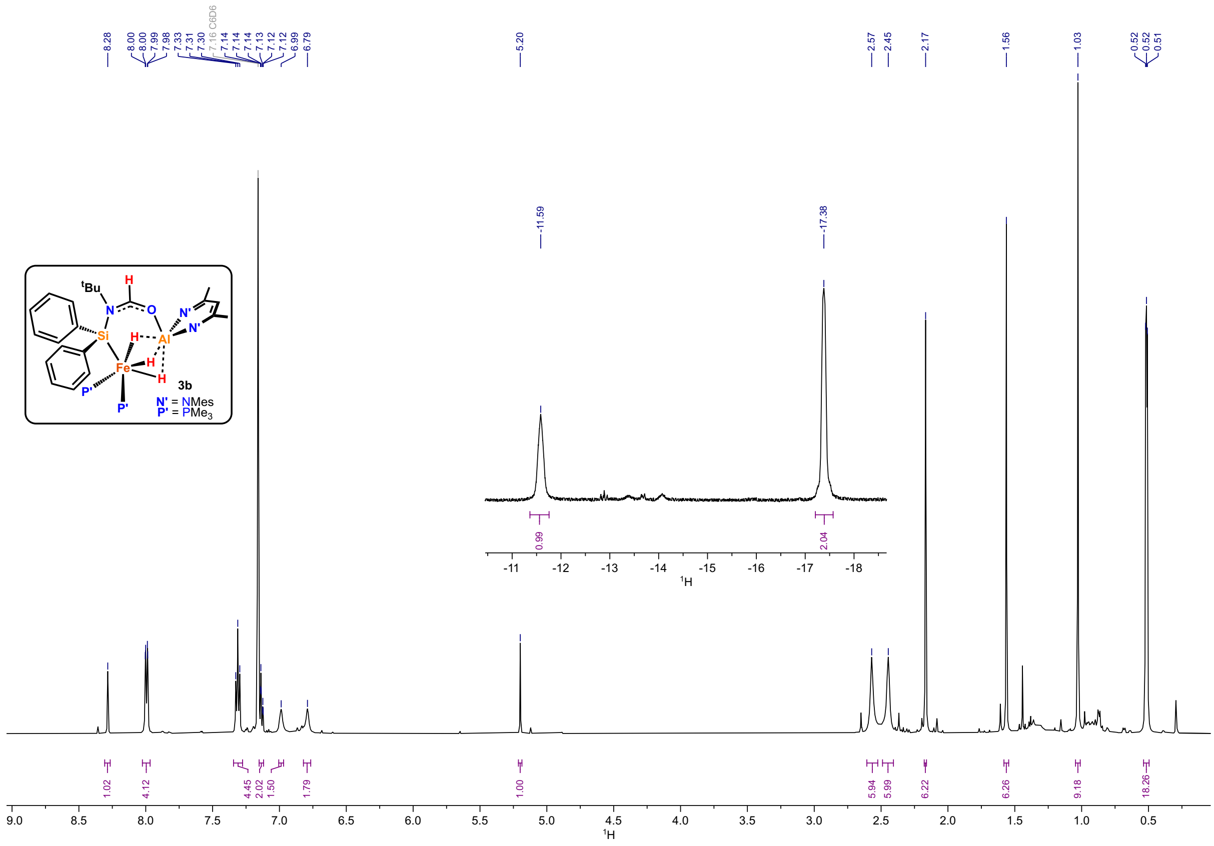

Figure S49 ^{1}H NMR spectrum of **3b** (C_6D_6, 298 K, 500 MHz).

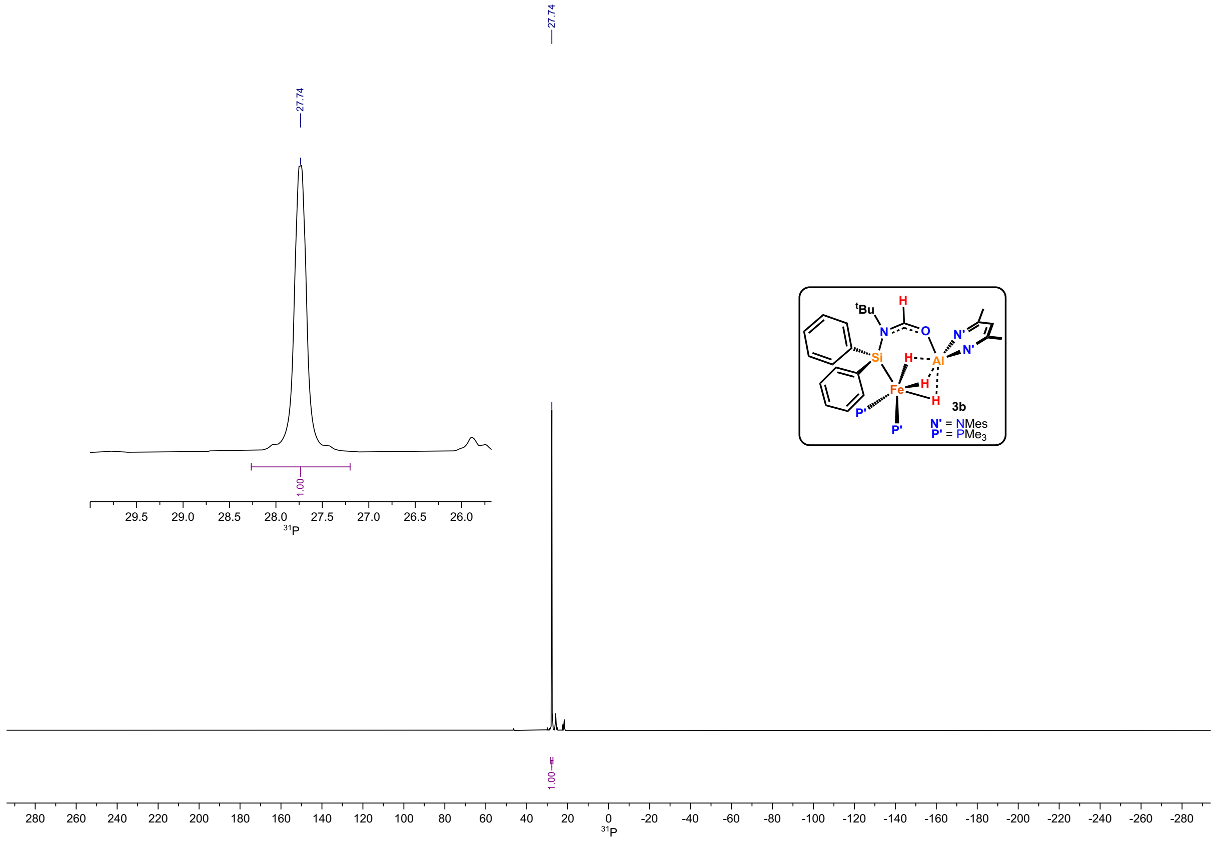

Figure S50 $^{31}P\{^{1}H\}$ NMR spectrum of **3b** (C_6D_6, 298 K, 202 MHz).

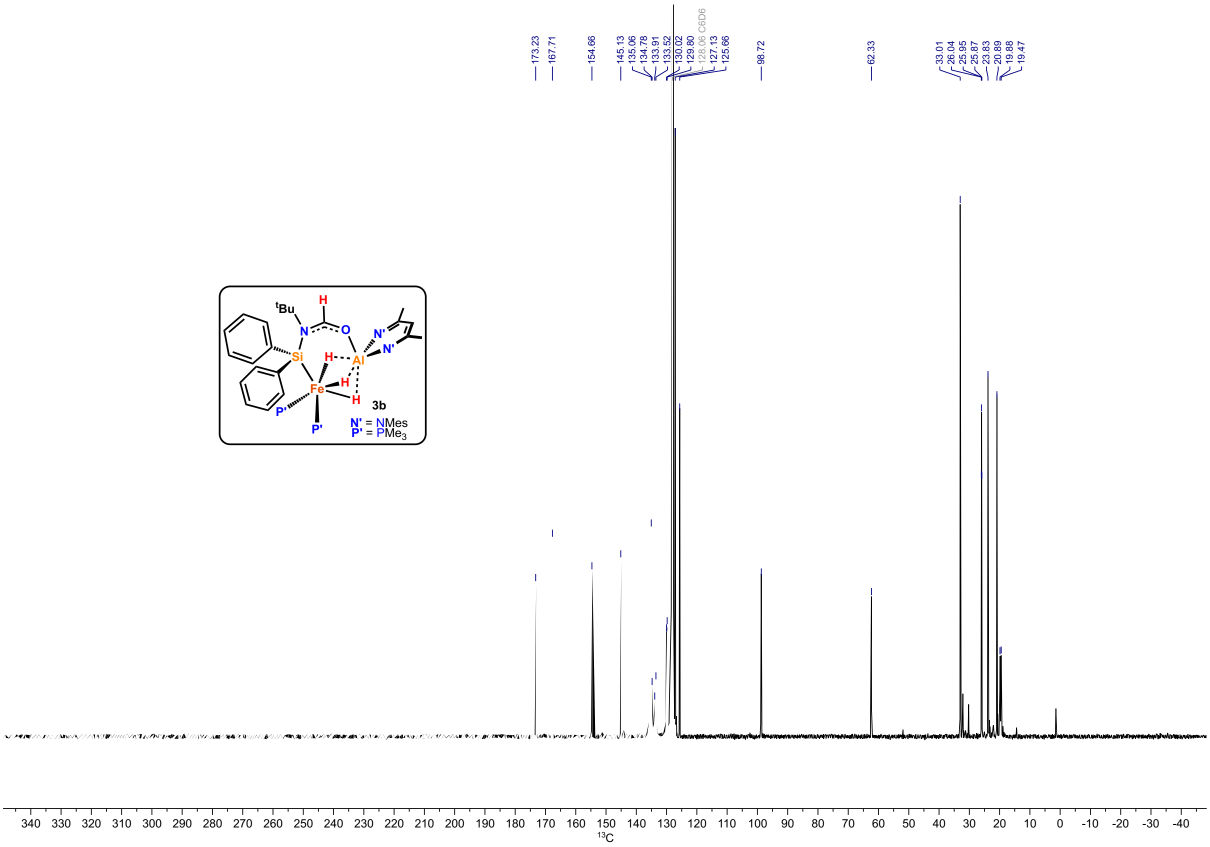

Figure S51 $^{13}C\{^{1}H\}$ NMR spectrum of **3b** (C_6D_6, 298 K, 126 MHz).

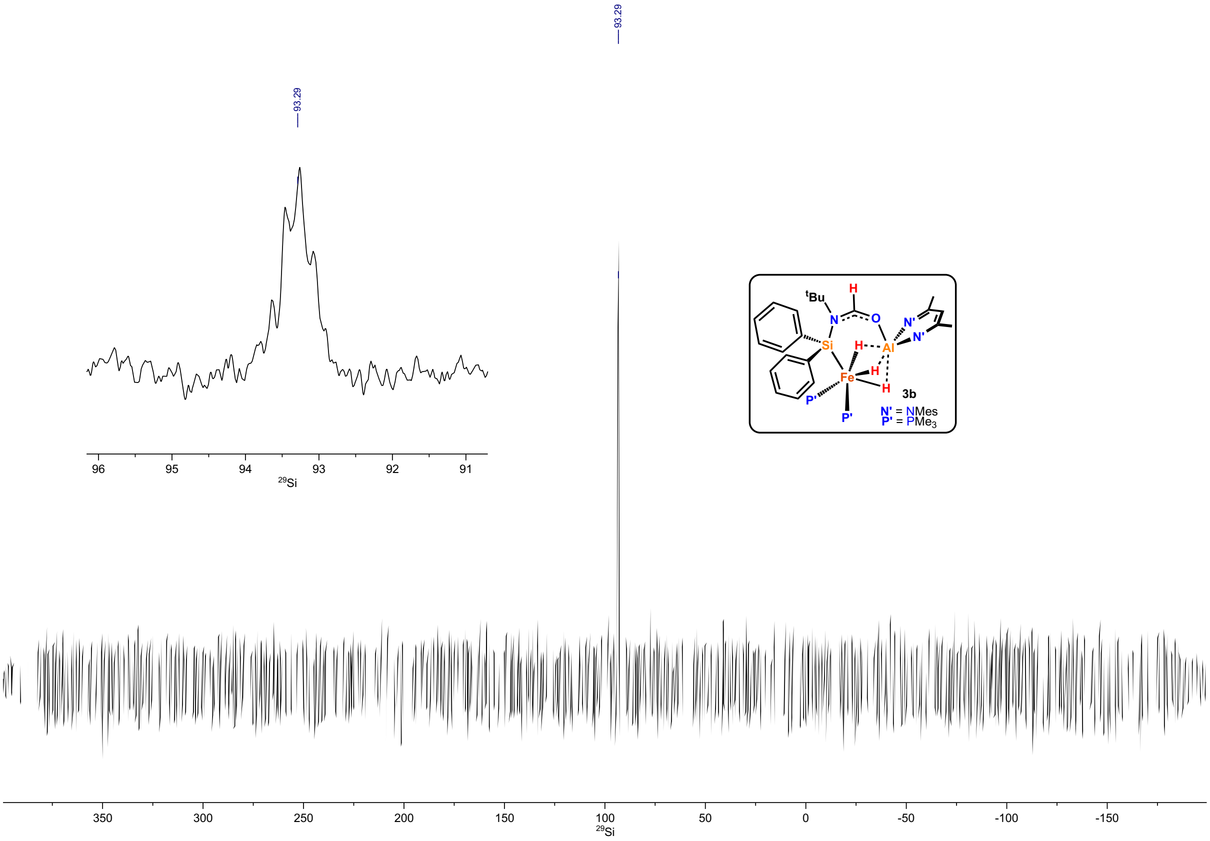

Figure S52 $^{29}Si\{^{1}H\}$ NMR spectrum of **3b** (C_6D_6, 298 K, 99 MHz, inverse gated).

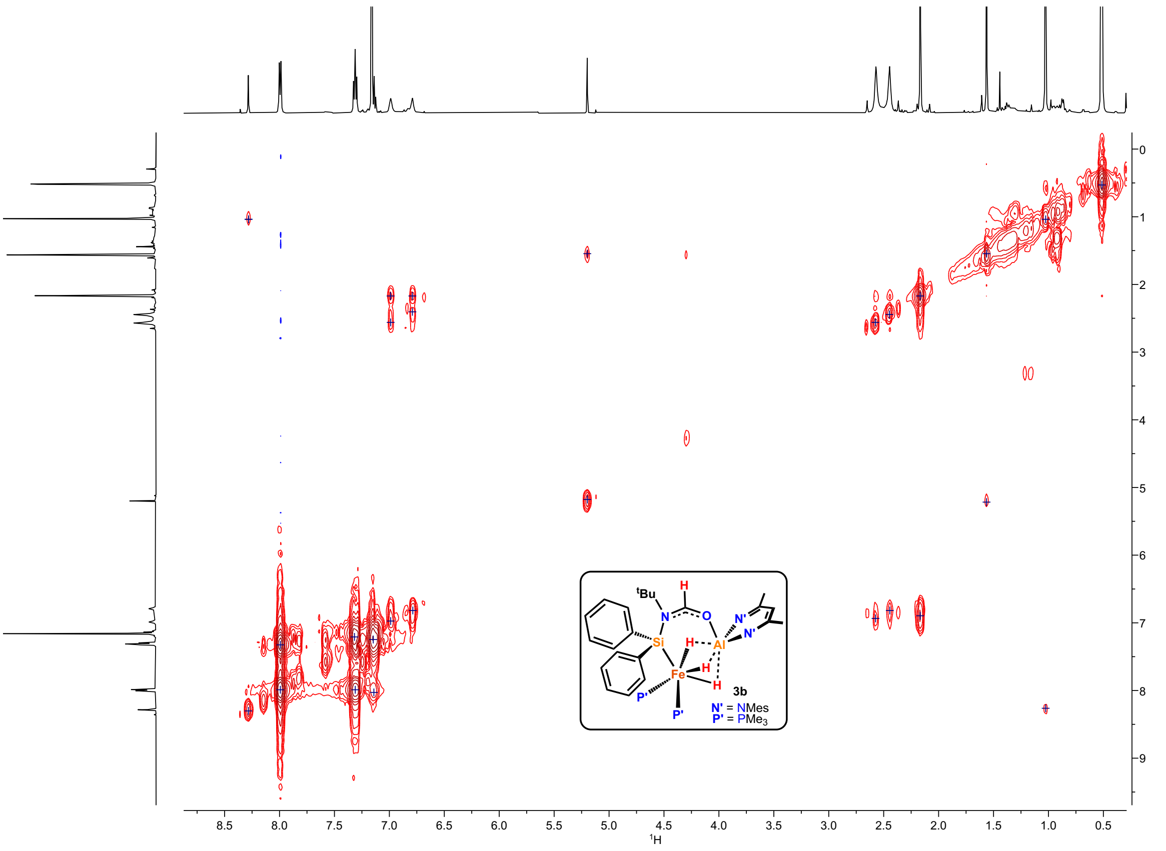

Figure S53 ^{1}H-^{1}H COSY NMR spectrum of **3b** (C_6D_6, 298 K, 500 MHz).

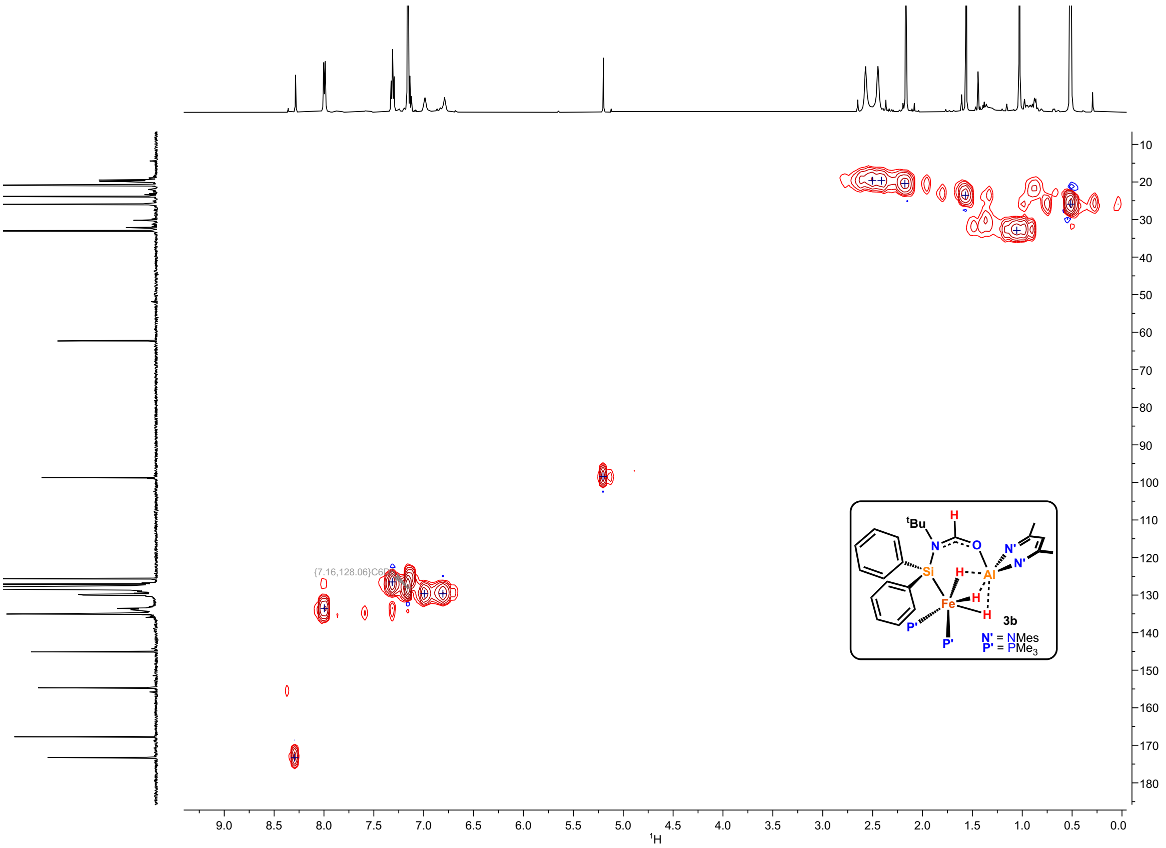

Figure S54 ^{1}H-^{13}C HSQC NMR spectrum of **3b** (C_6D_6, 298 K, 500 MHz).

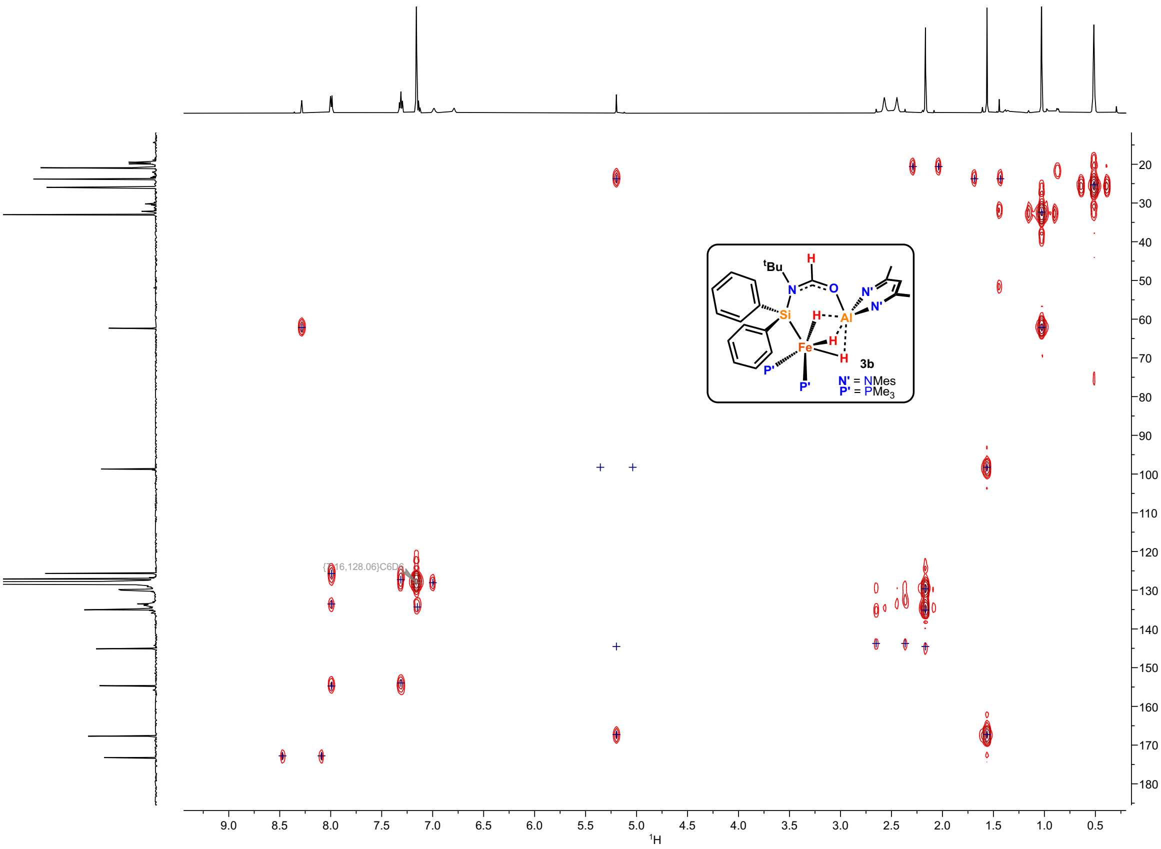

Figure S55 ^{1}H-^{13}C HSQC NMR spectrum of **3b** (C_6D_6, 298 K, 500 MHz).

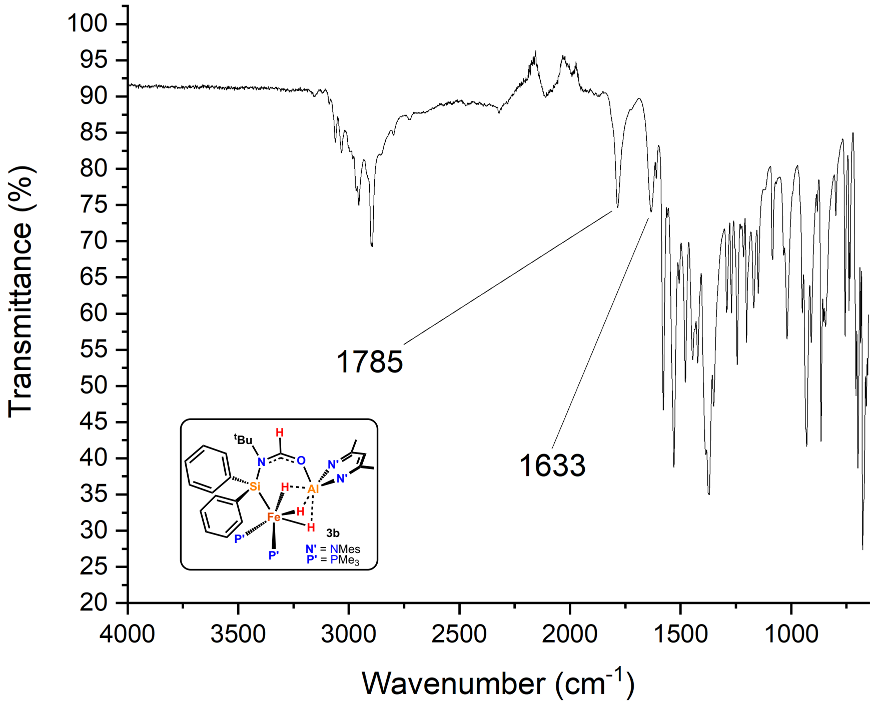

Figure S56 FT-IR spectrum of **3b** (thin film, 298 K, ATR).

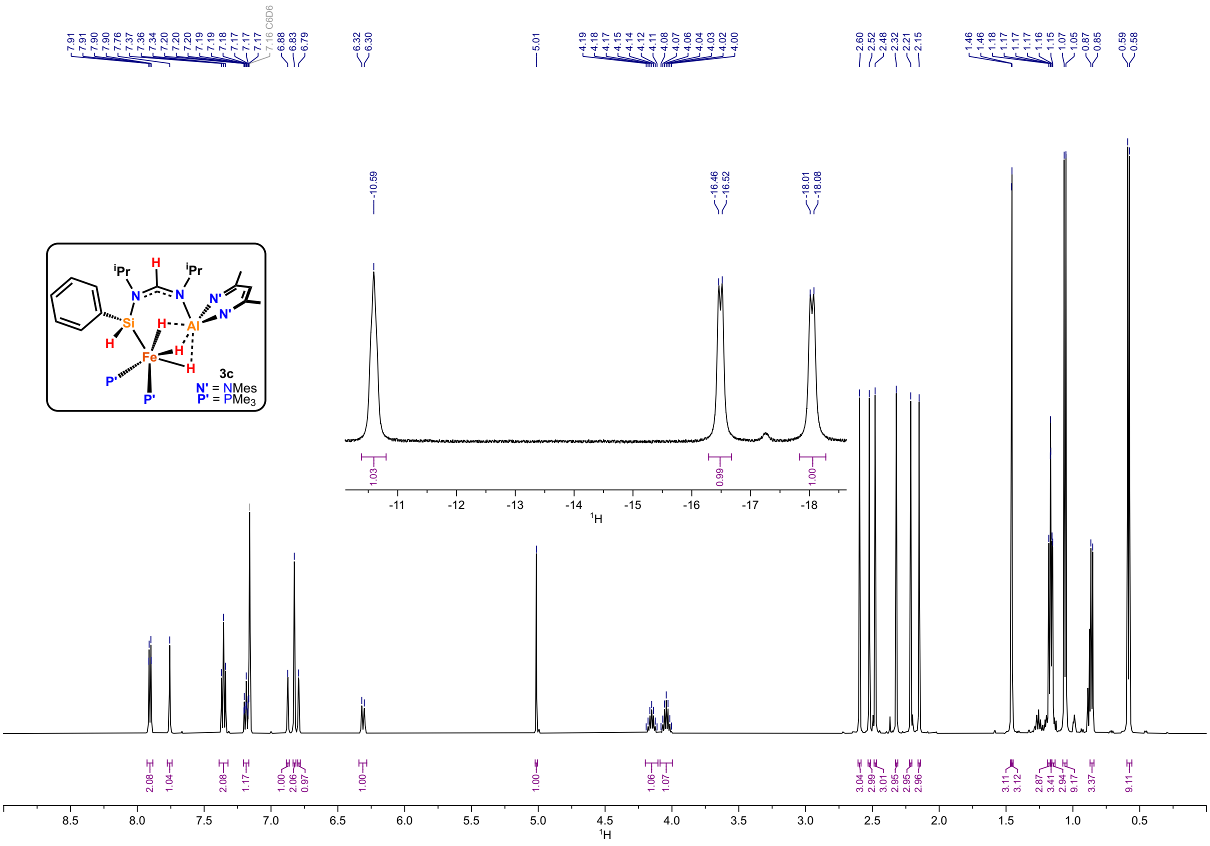

Figure S57 ^{1}H NMR spectrum of **3c** (C_6D_6, 298 K, 500 MHz).

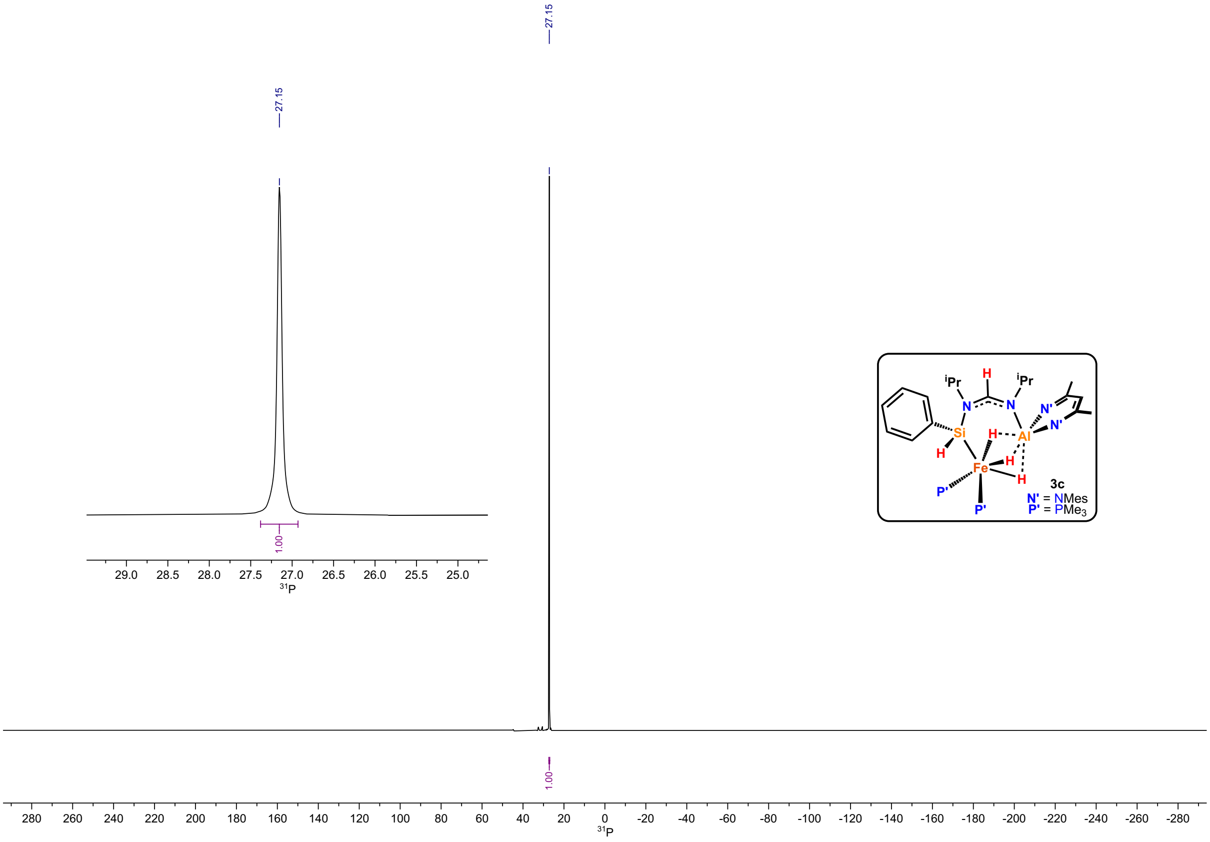

Figure S58 $^{31}P\{^{1}H\}$ NMR spectrum of **3c** (C_6D_6, 298 K, 202 MHz).

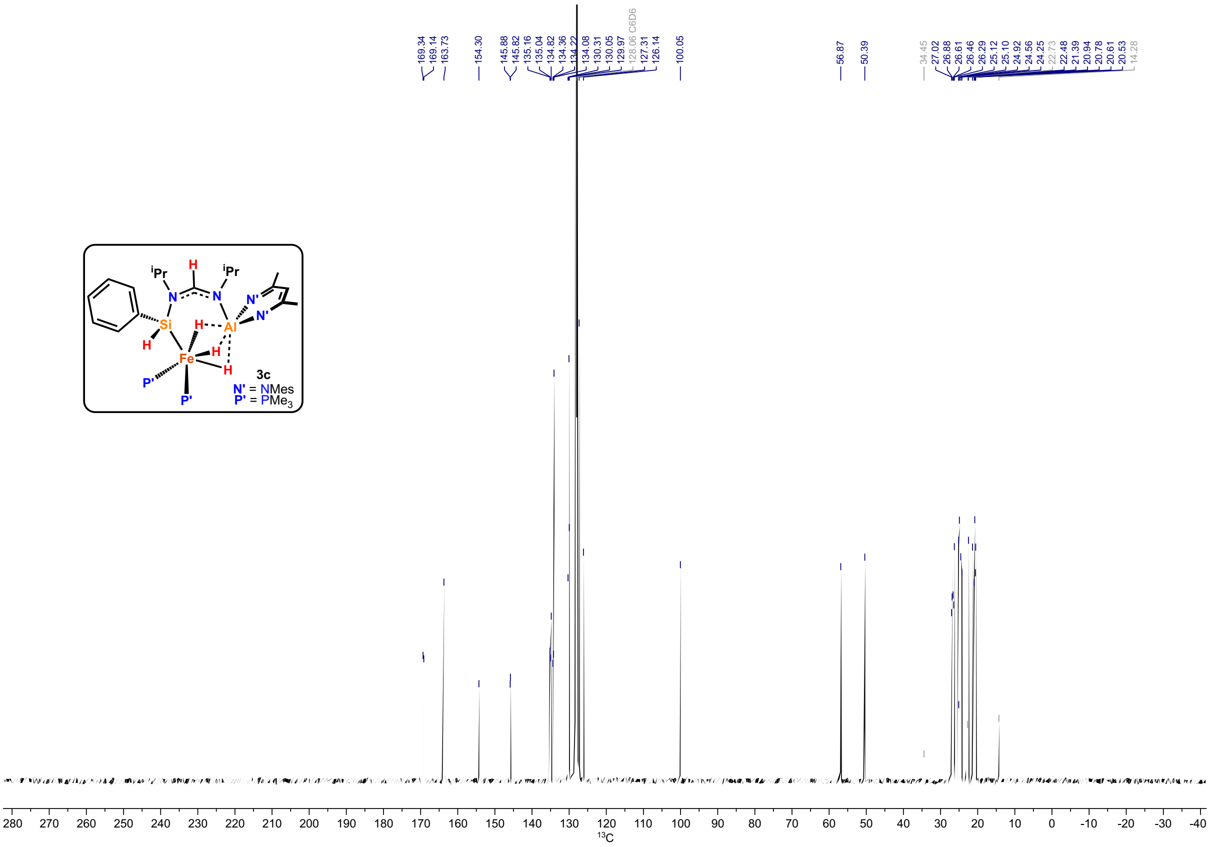

Figure S59 $^{13}C\{^{1}H\}$ NMR spectrum of **3c** (C_6D_6, 298 K, 126 MHz).

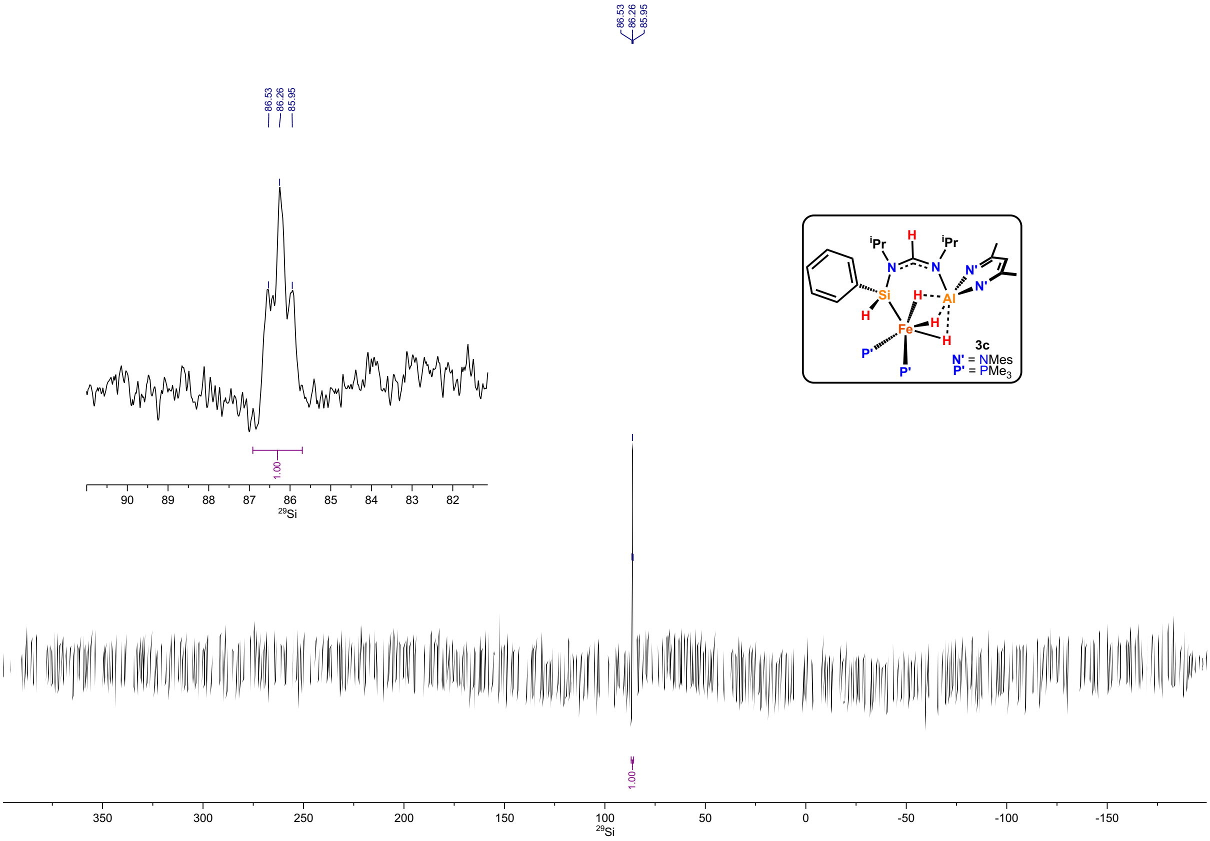

Figure S60 $^{29}Si\{^{1}H\}$ NMR spectrum of **3c** (C_6D_6, 298 K, 99 MHz, inverse gated).

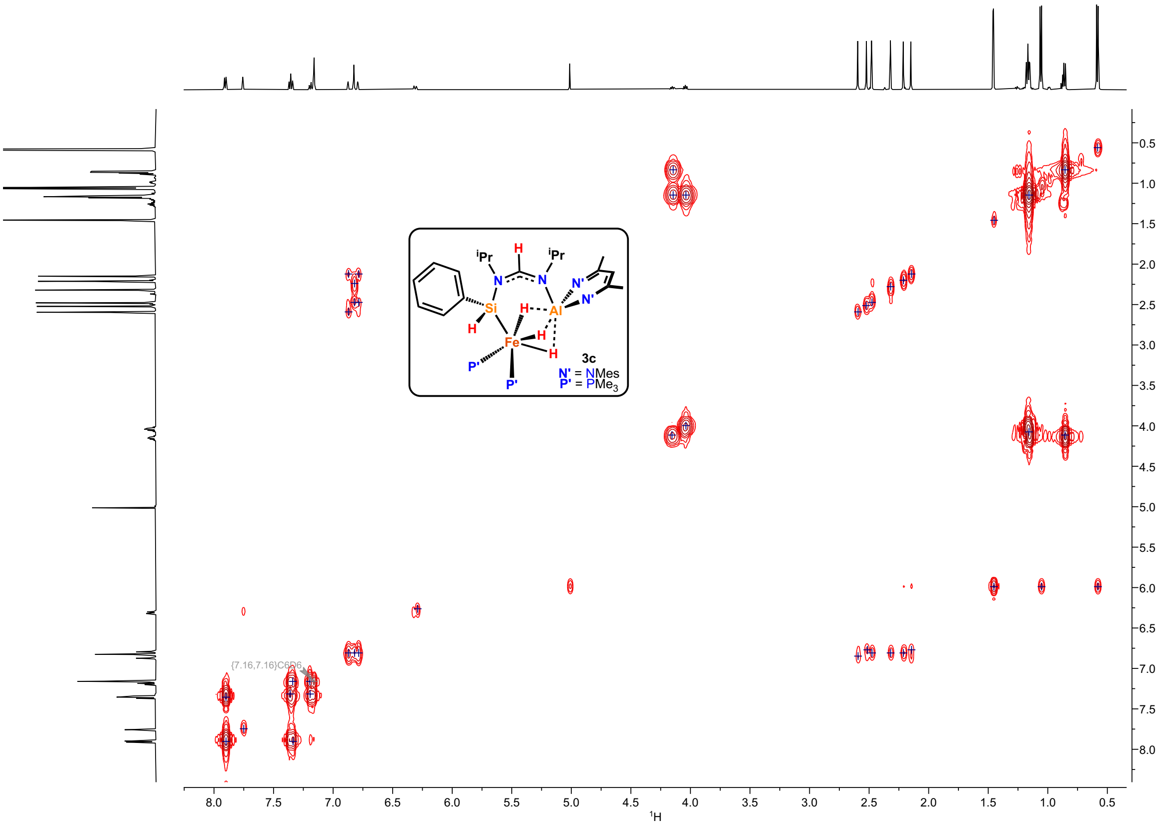

Figure S61 ^{1}H-^{1}H COSY NMR spectrum of **3c** (C_6D_6, 298 K, 500 MHz).

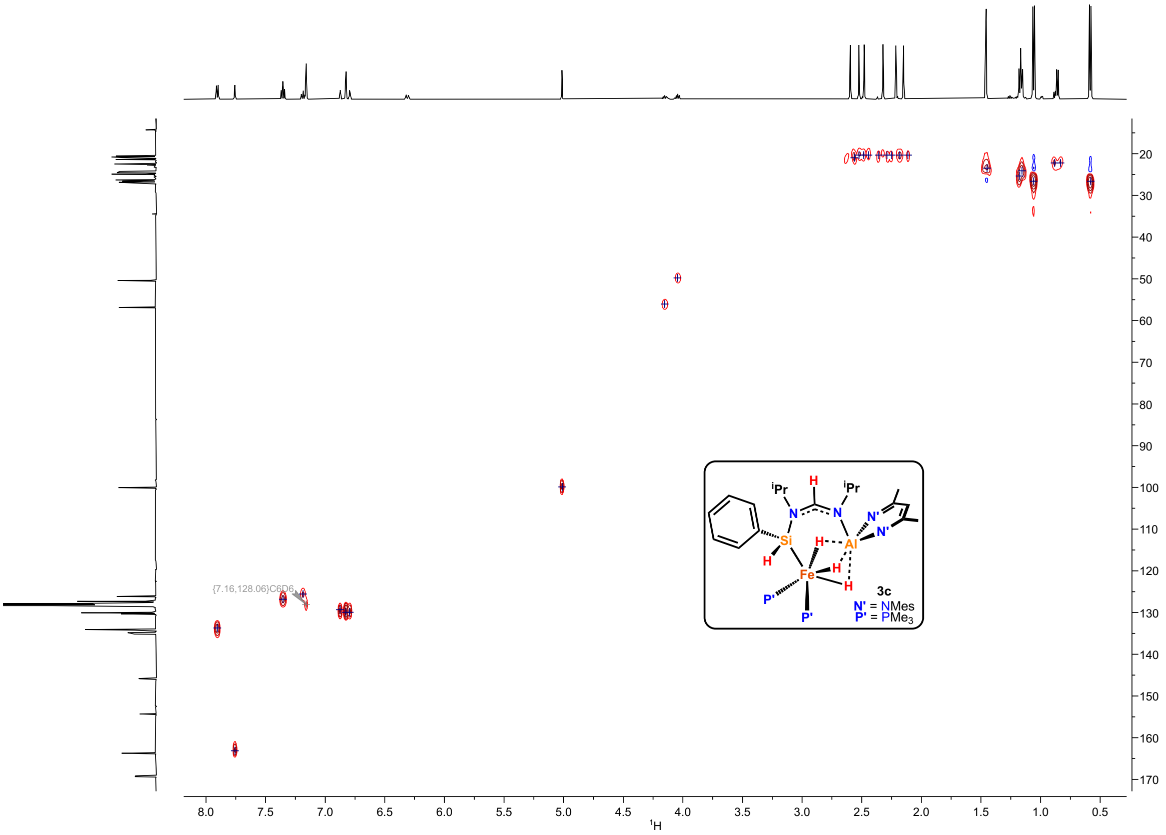

Figure S62 ^{1}H-^{1}H HSQC NMR spectrum of **3c** (C_6D_6, 298 K, 500 MHz).

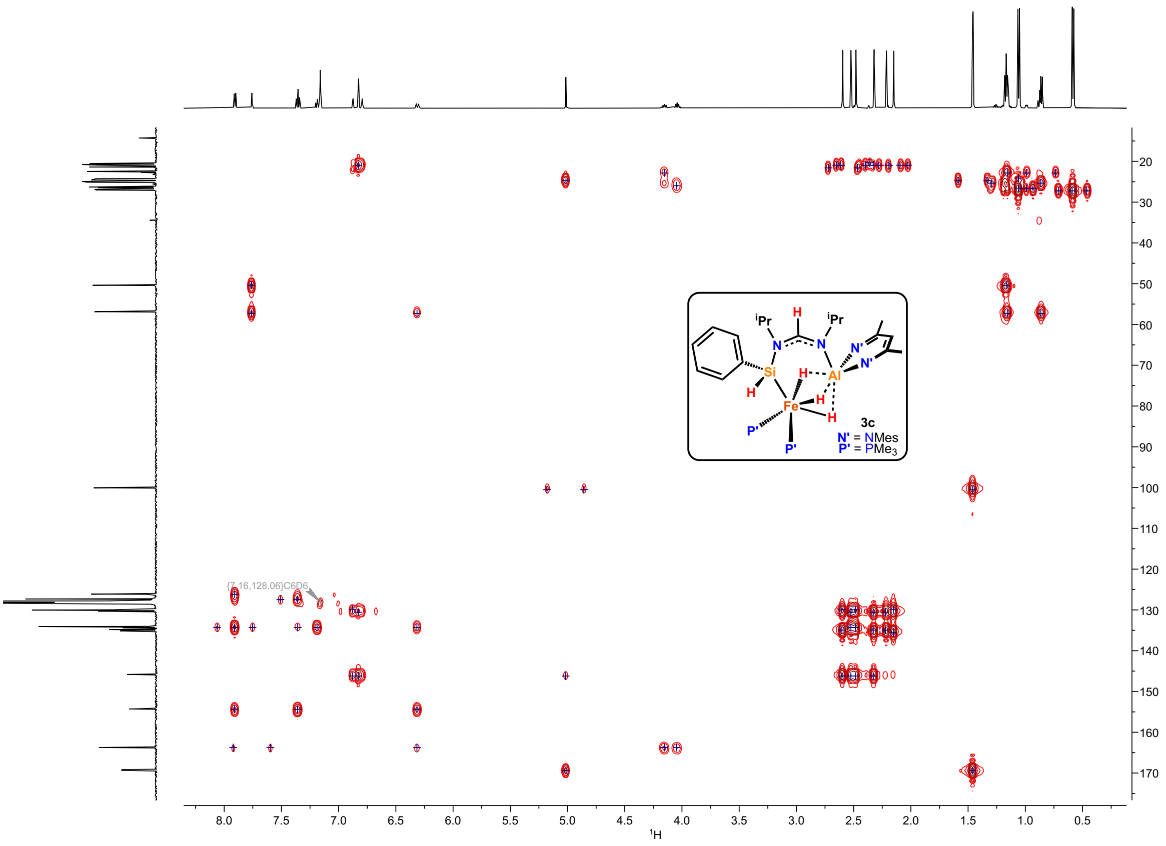

Figure S63^{1}H-^{1}H HMBC NMR spectrum of **3c** (C_6D_6, 298 K, 500 MHz).

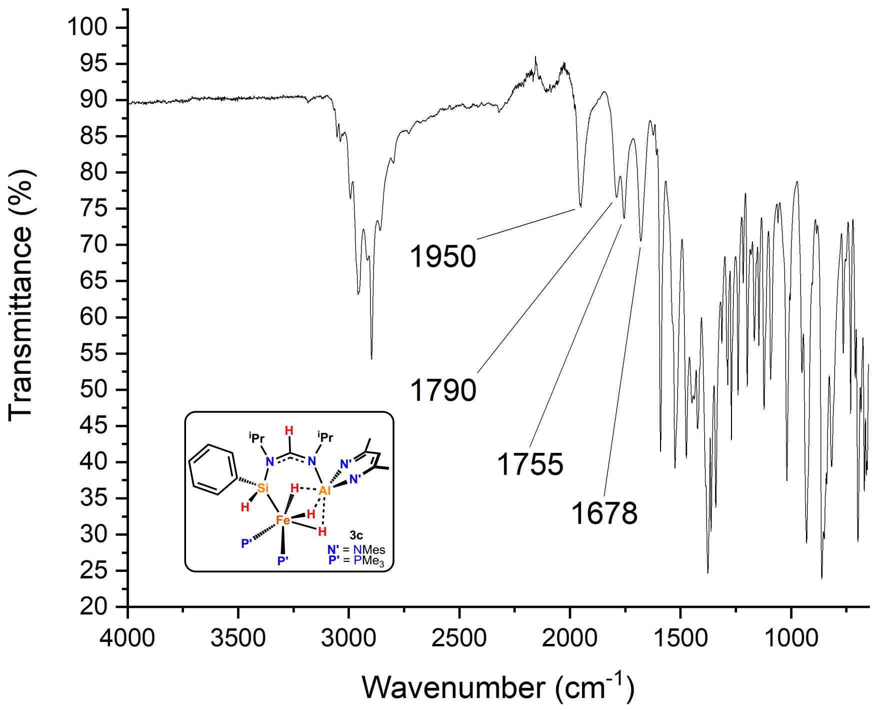

Figure S64 FT-IR spectrum of **3c** (thin film, 298 K, ATR).

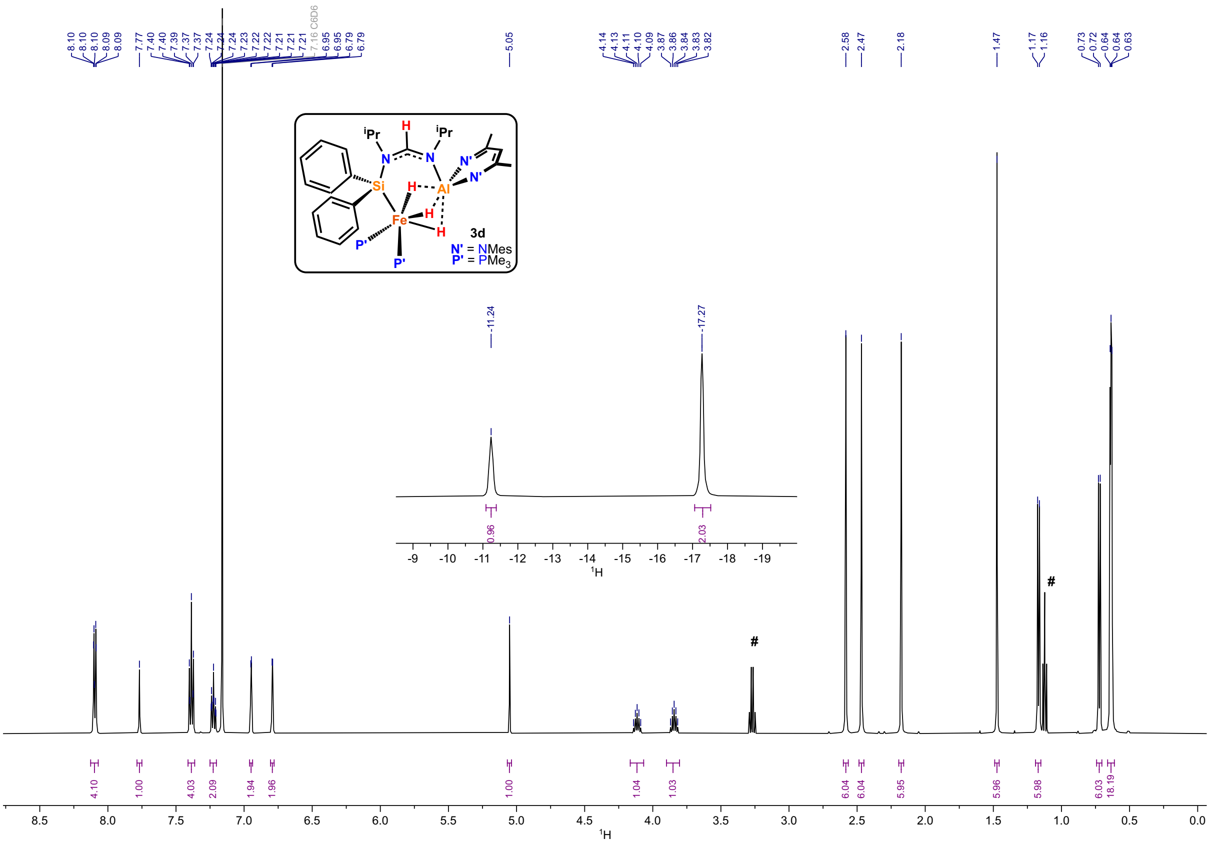

Figure S65 ^{1}H NMR spectrum of **3d** (C_6D_6, 298 K, 500 MHz). #: Et_2O

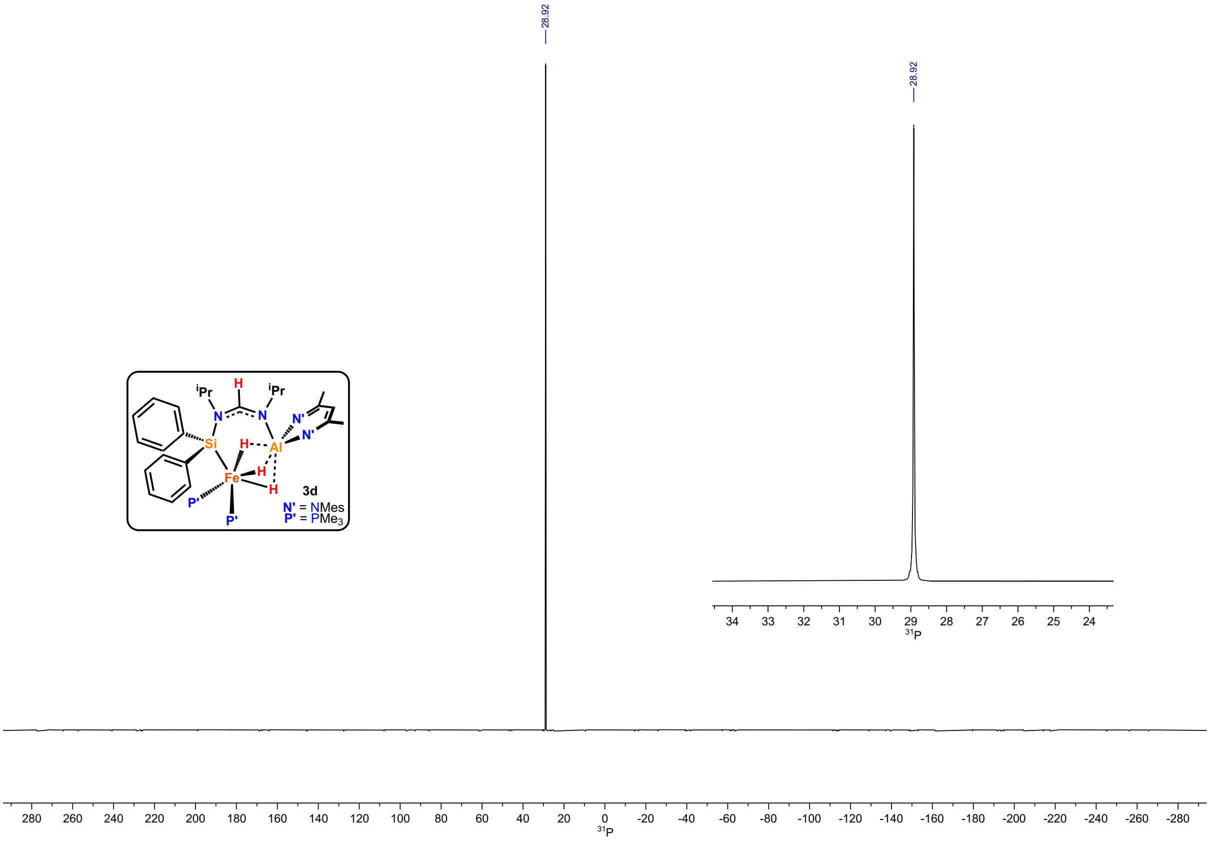

Figure S66 $^{31}P\{^{1}H\}$ NMR spectrum of **3d** (C_6D_6, 298 K, 202 MHz).

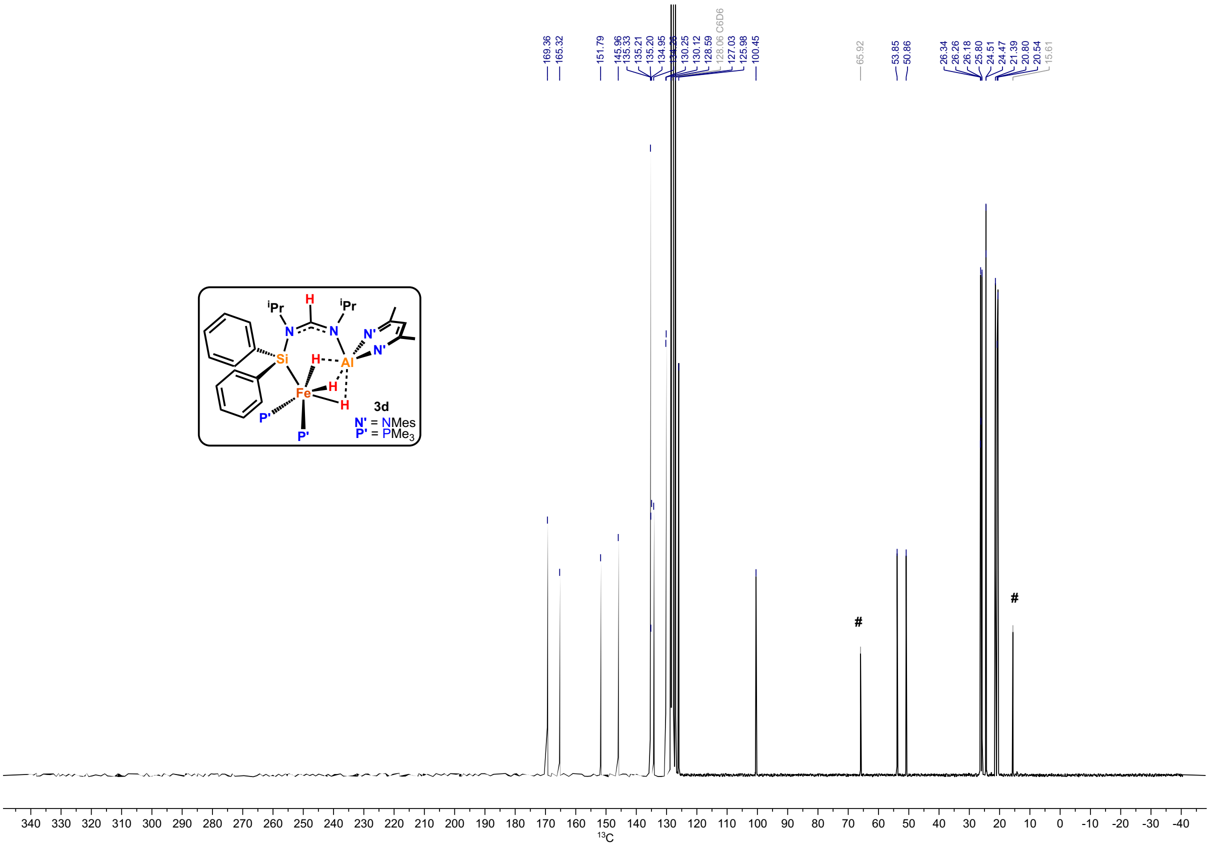

Figure S67 $^{13}C\{^{1}H\}$ NMR spectrum of **3d** (C_6D_6, 298 K, 126 MHz). #: Et_2O

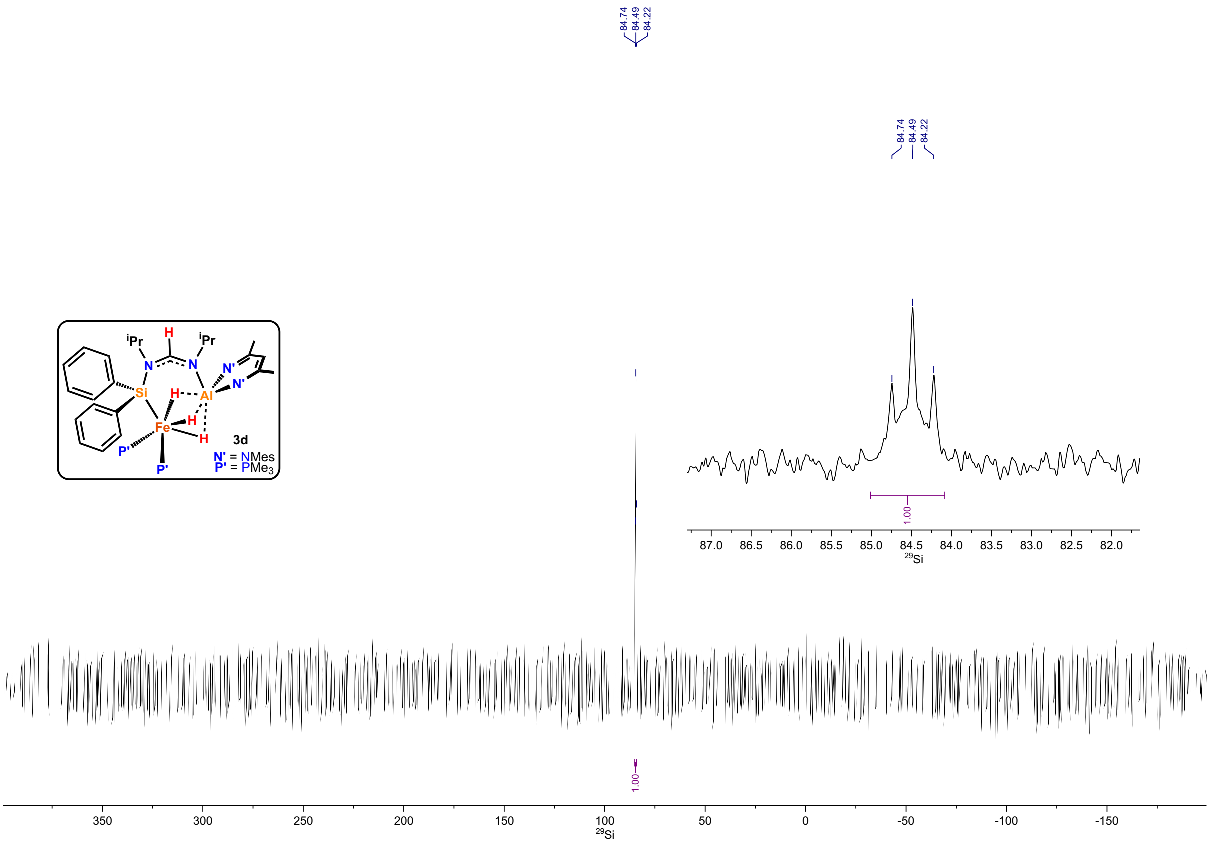

Figure S68 $^{29}Si\{^{1}H\}$ NMR spectrum of **3d** (C_6D_6, 298 K, 99 MHz, inverse gated).

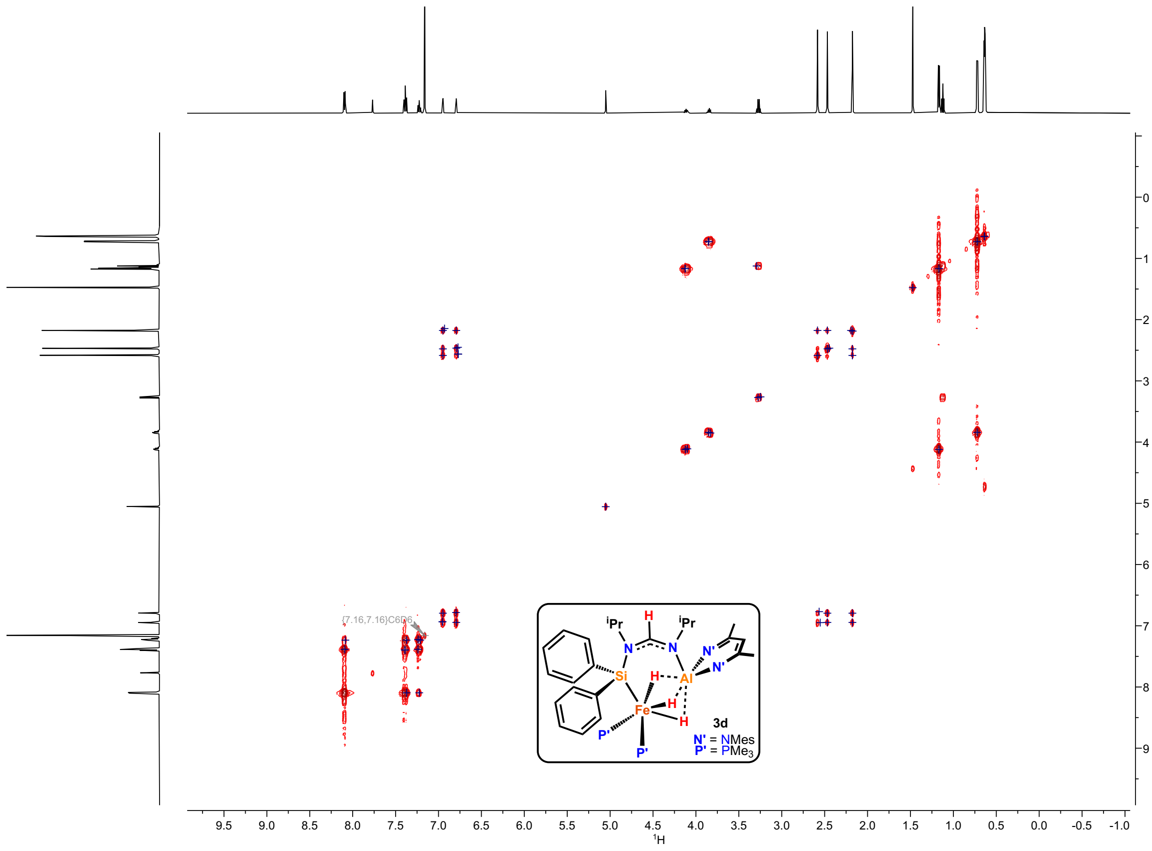

Figure S69 ^{1}H-^{1}H COSY NMR spectrum of **3d** (C_6D_6, 298 K, 500 MHz).

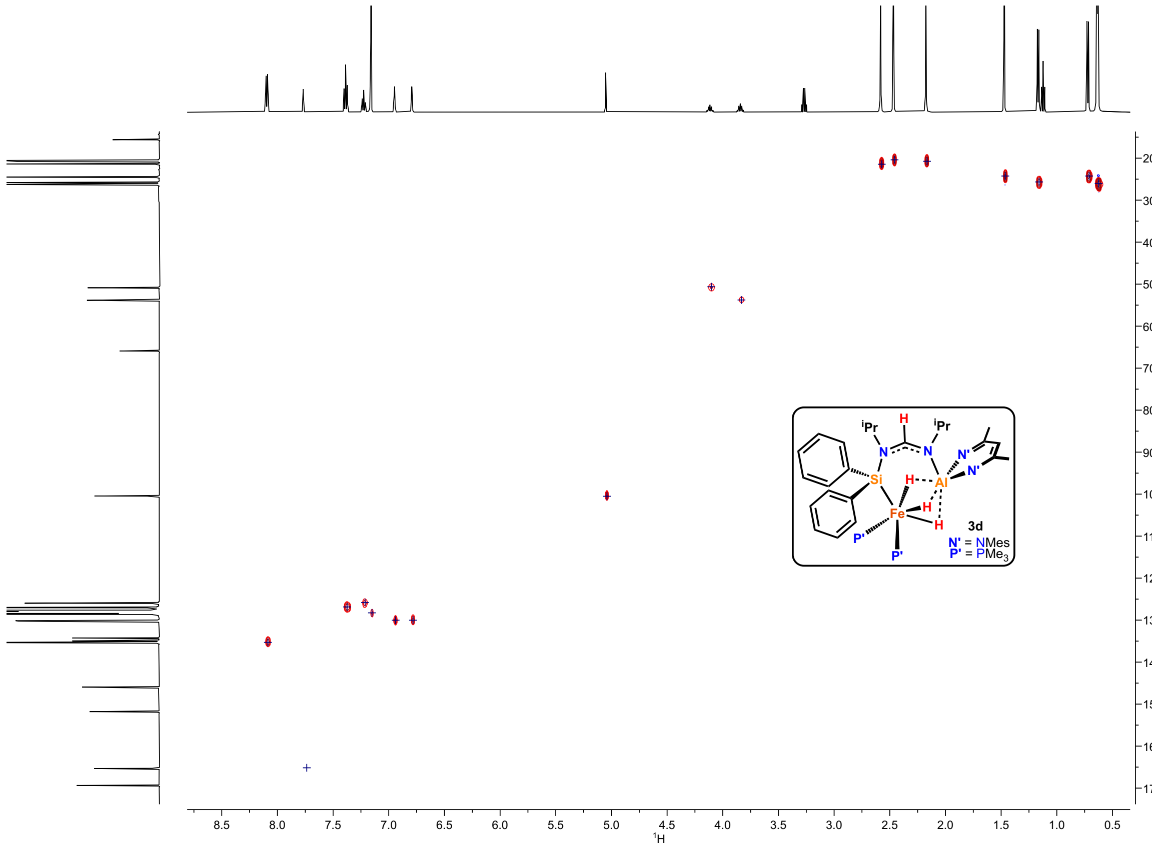

Figure S70 ^{1}H-^{13}C HSQC NMR spectrum of **3d** (C_6D_6, 298 K, 500 MHz).

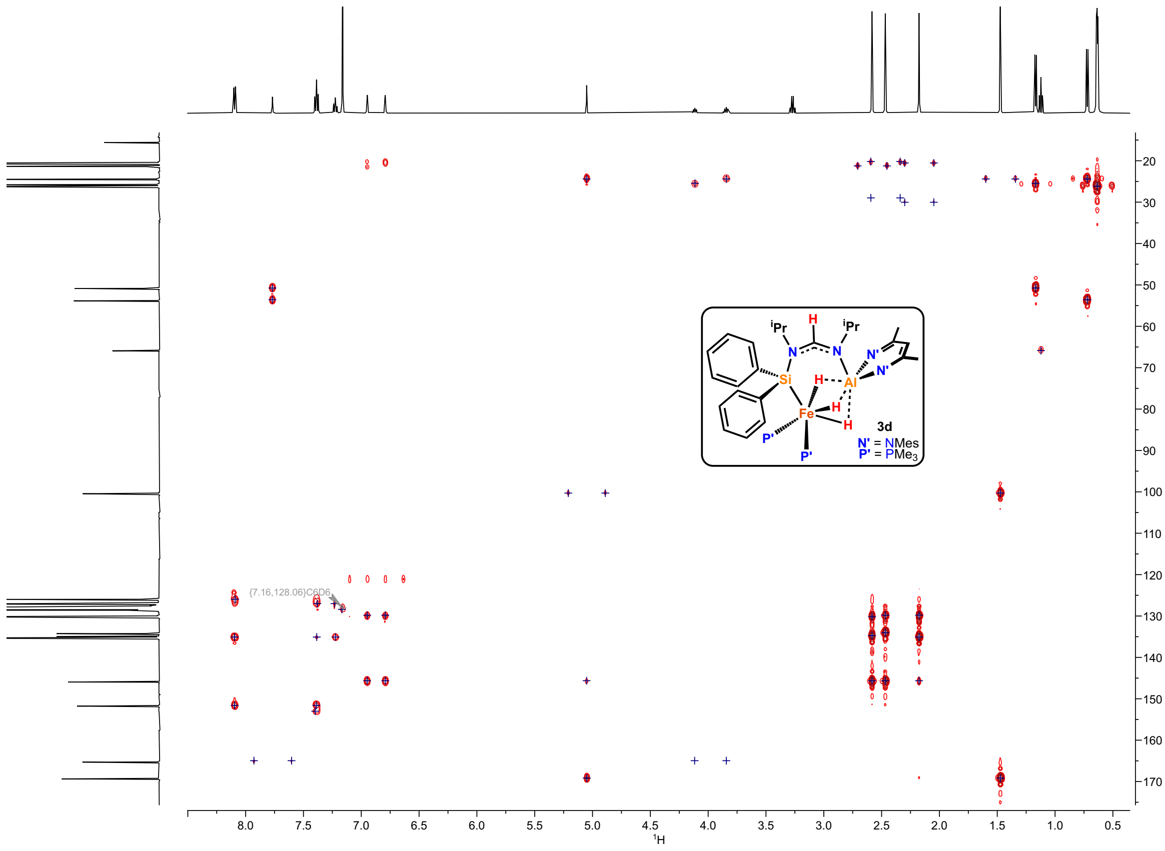

Figure S71 ^{1}H-^{13}C HMBC NMR spectrum of **3d** (C_6D_6, 298 K, 500 MHz).

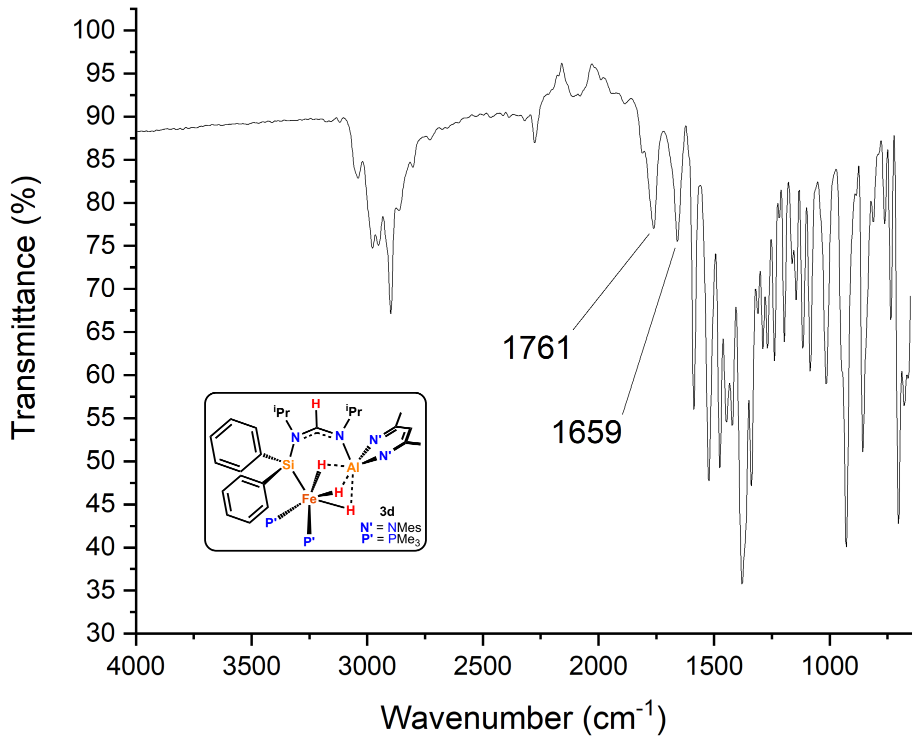

Figure S72 FT-IR spectrum of **3d** (thin film, 298 K, ATR).

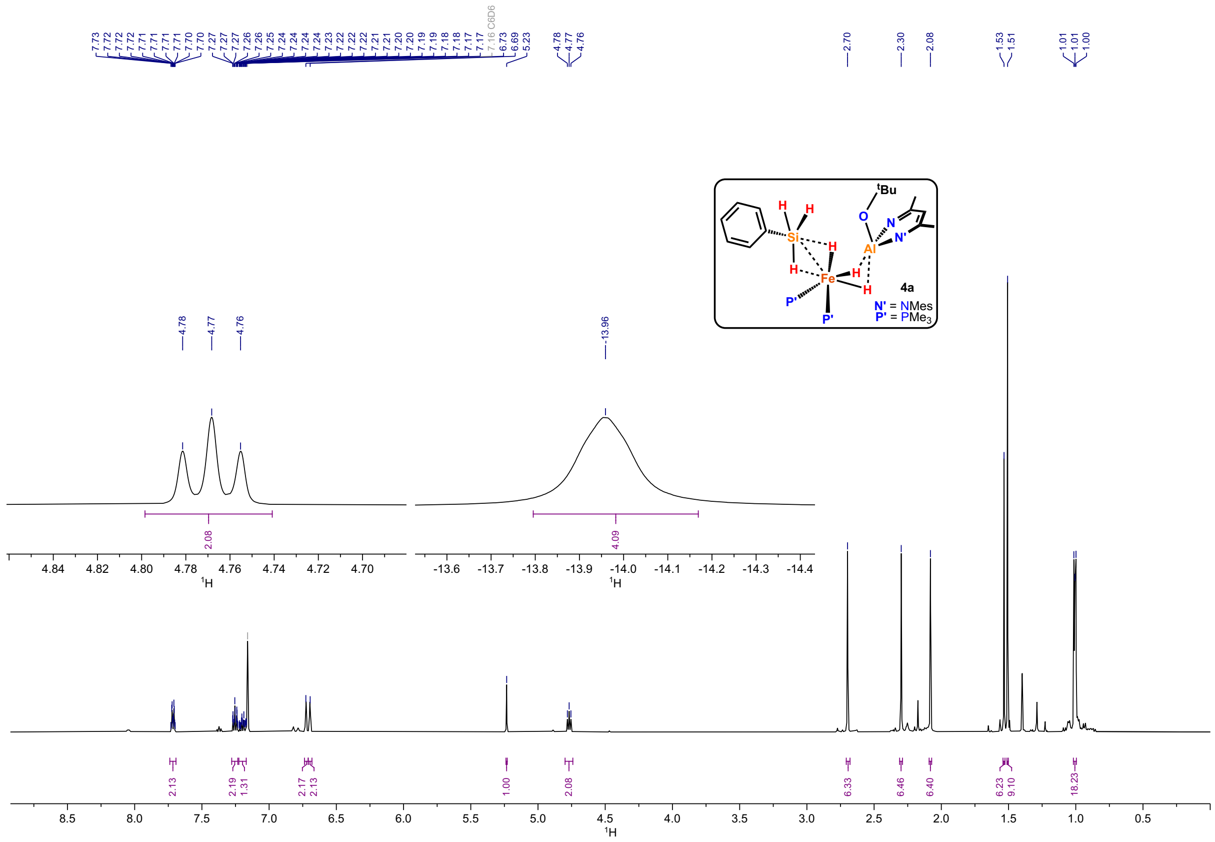

Figure S73 ^{1}H NMR spectrum of **4a** (C_6D_6, 298 K, 500 MHz).

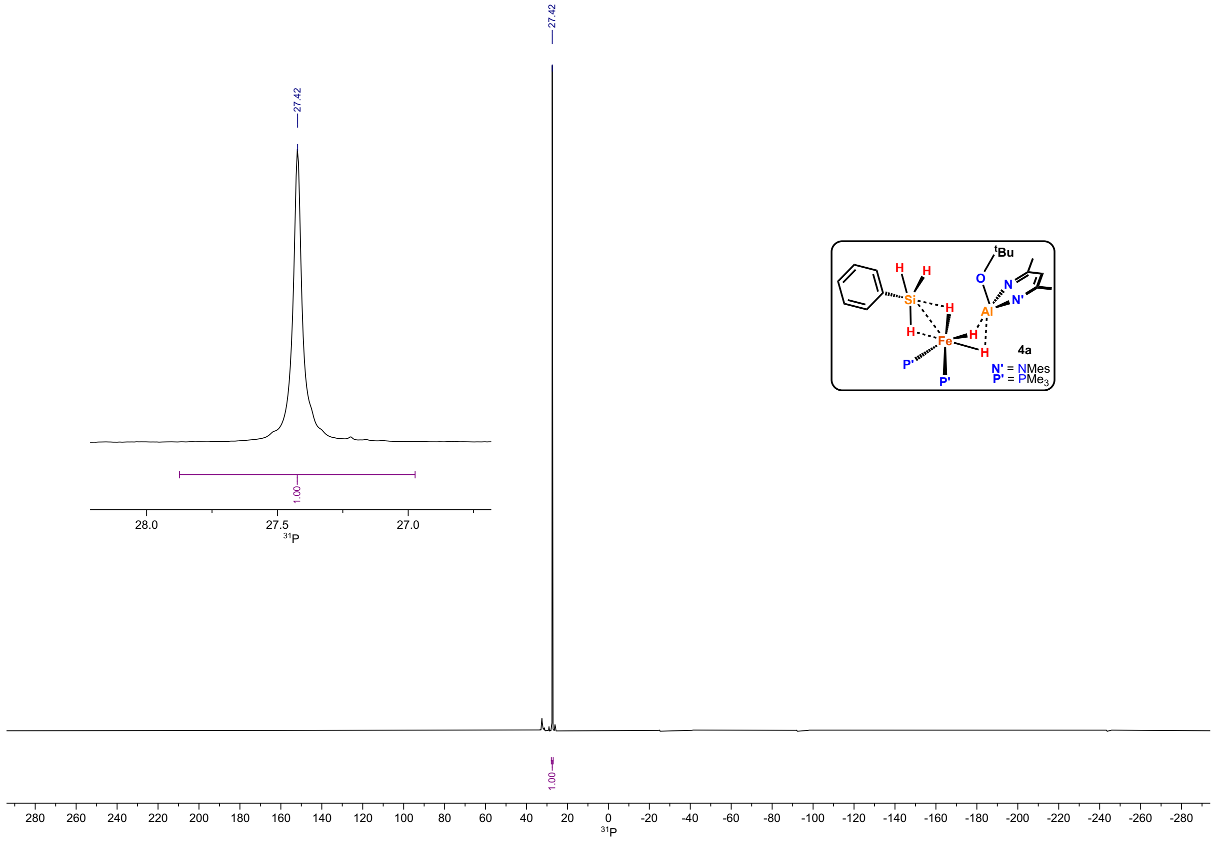

Figure S74 $^{31}P\{^{1}H\}$ NMR spectrum of **4a** (C_6D_6, 298 K, 202 MHz).

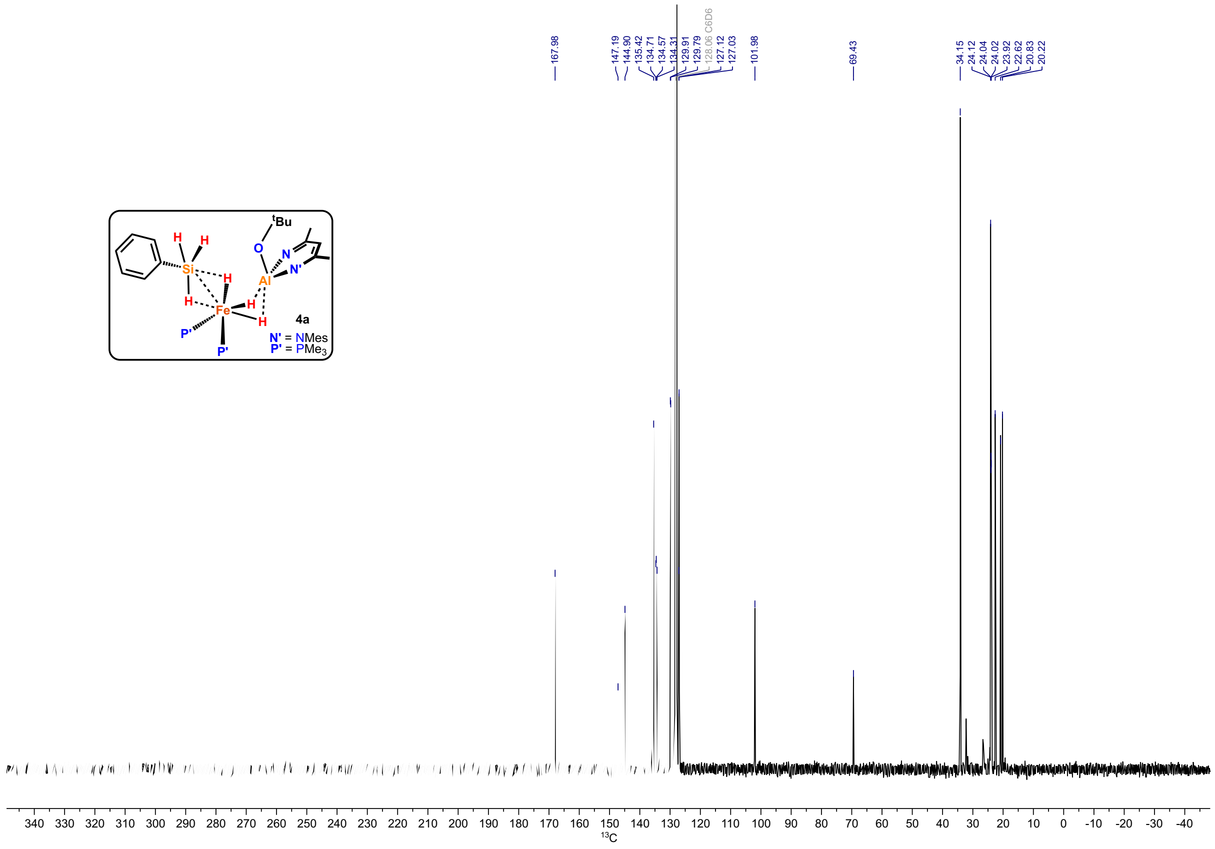

Figure S75 $^{13}C\{^{1}H\}$ NMR spectrum of **4a** (C_6D_6, 298 K, 126 MHz).

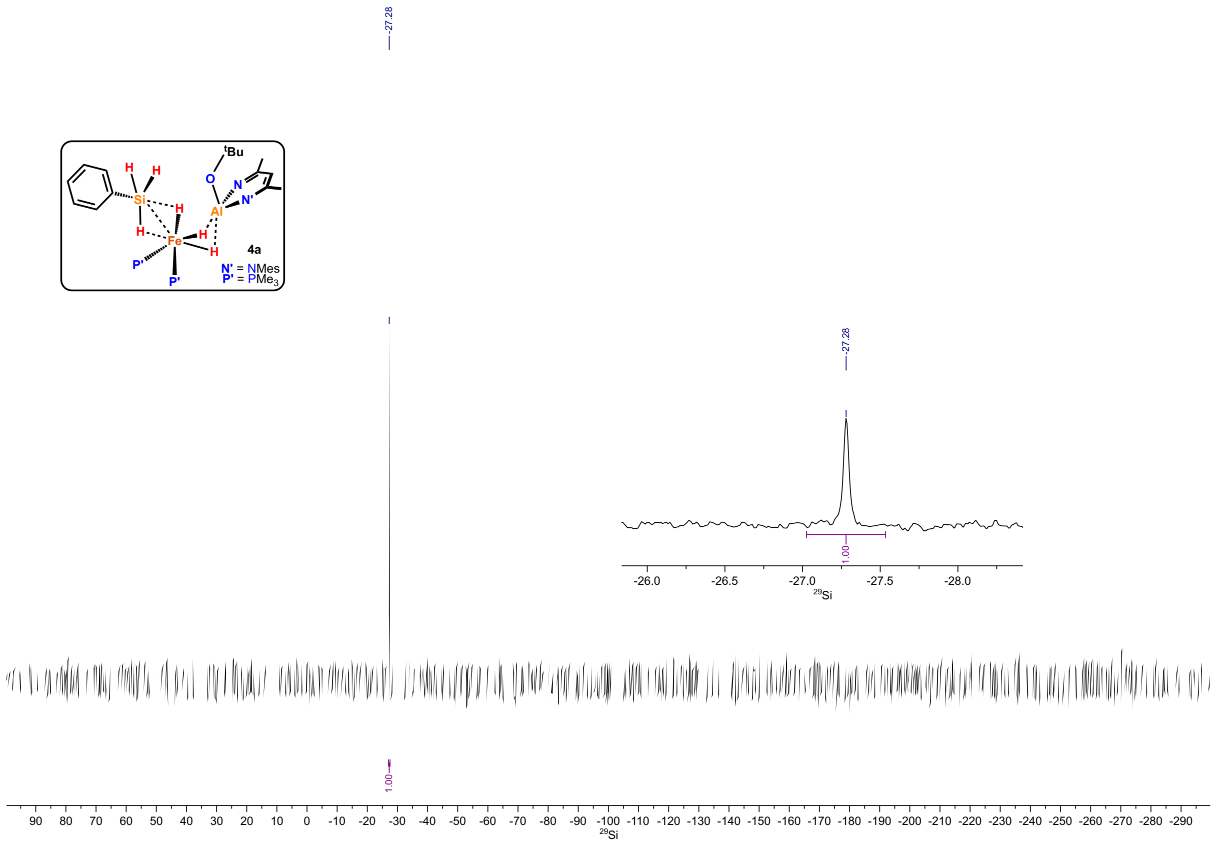

Figure S76 $^{29}Si\{^{1}H$ NMR spectrum of **4a** (C_6D_6, 298 K, 99 MHz, inverse gated).

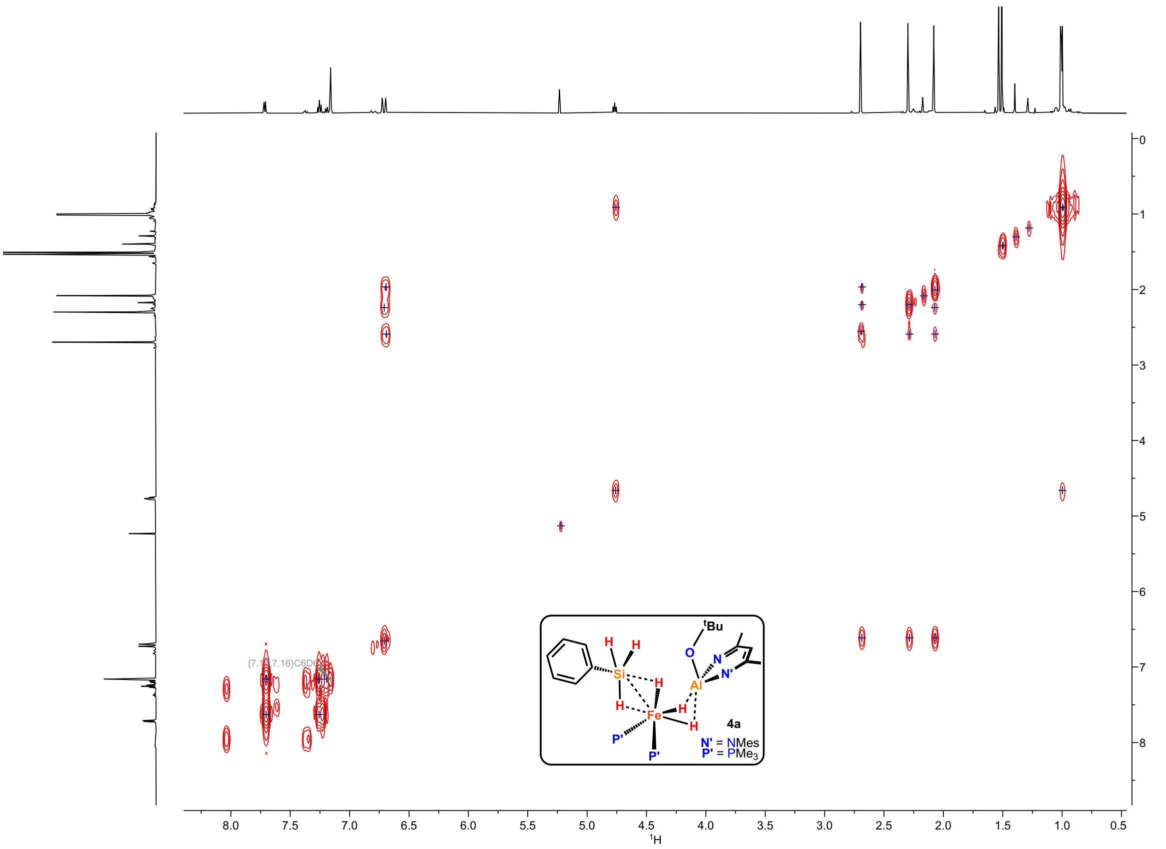

Figure S77 ^{1}H-^{1}H COSY NMR spectrum of **4a** (C_6D_6, 298 K, 500 MHz).

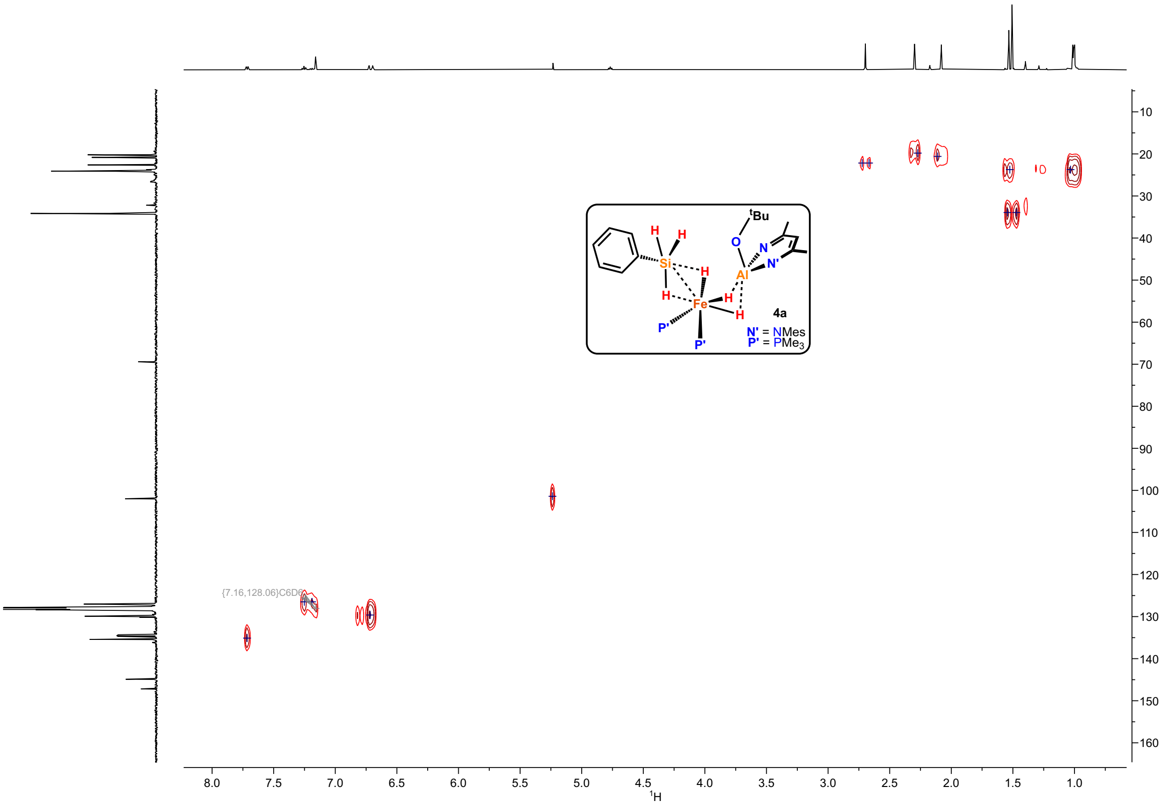

Figure S78 ^{1}H-^{13}C HSQC NMR spectrum of **4a** (C_6D_6, 298 K, 500 MHz).

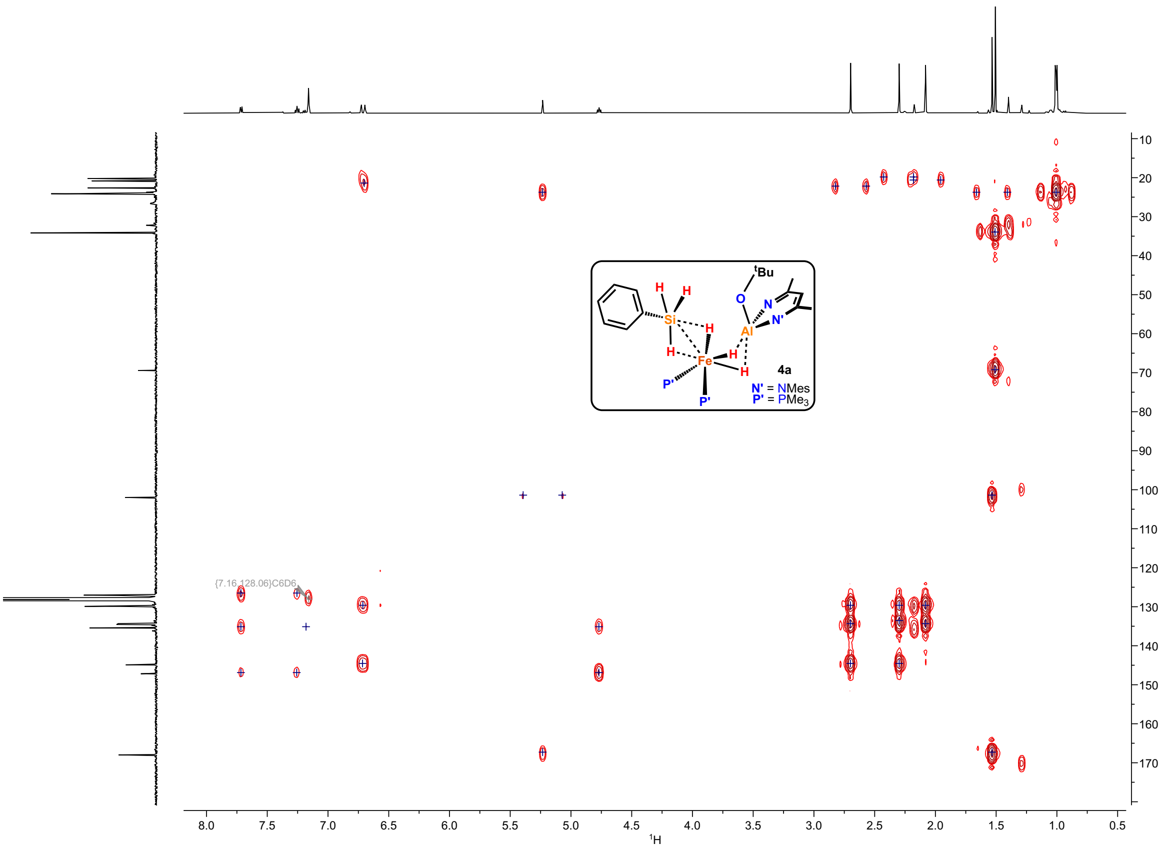

Figure S79 ^{1}H-^{13}C HMBC NMR spectrum of **4a** (C_6D_6, 298 K, 500 MHz).

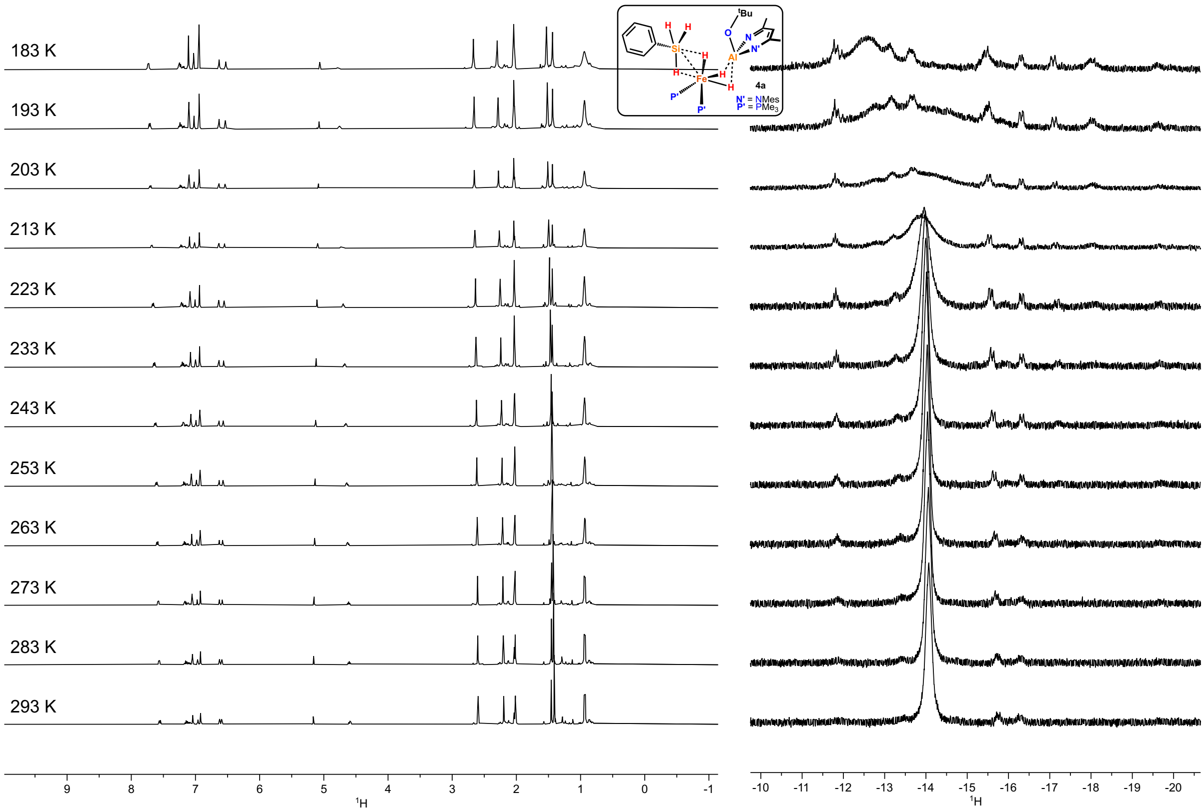

Figure S80 Stacked variable-temperature ^{1}H NMR spectra of **4a** (toluene-[D_8], 183-293 K, 400 MHz).

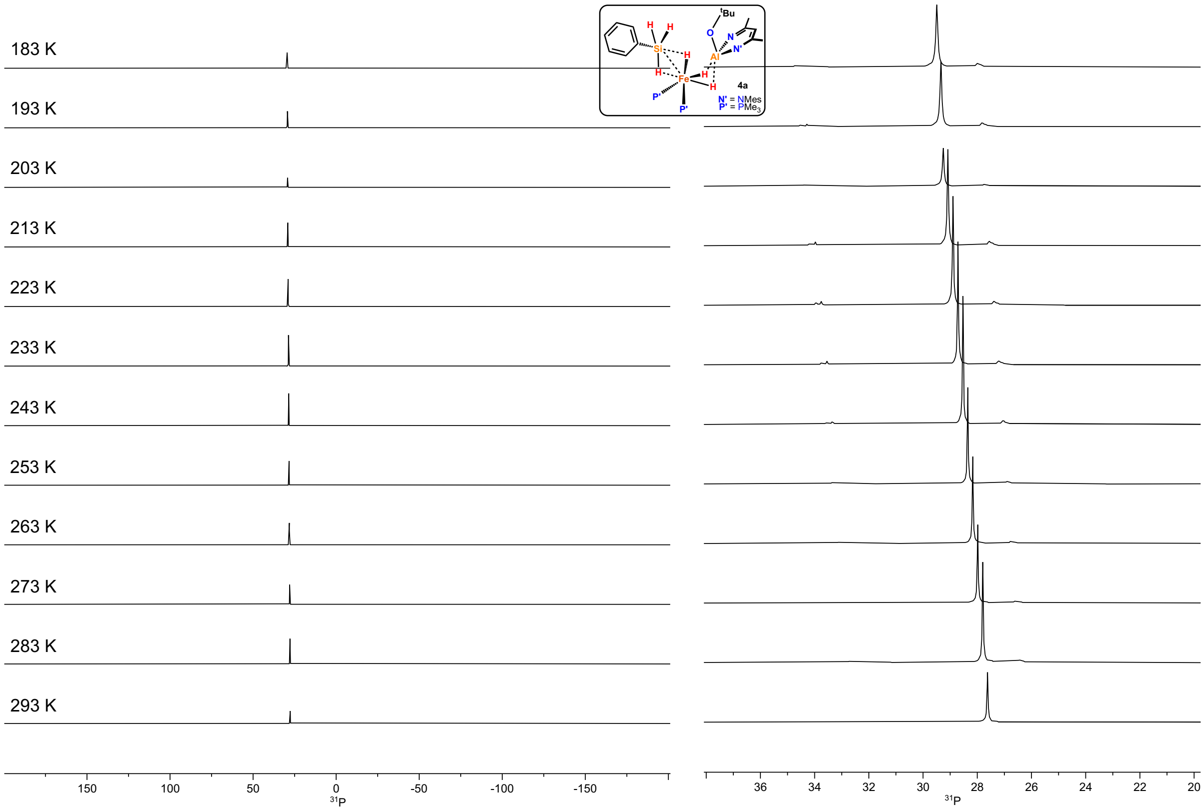

Figure S81 Stacked variable-temperature $^{31}P\{^{1}H\}$ NMR spectra of **4a** (toluene-[D_8], 183-293 K, 162 MHz).

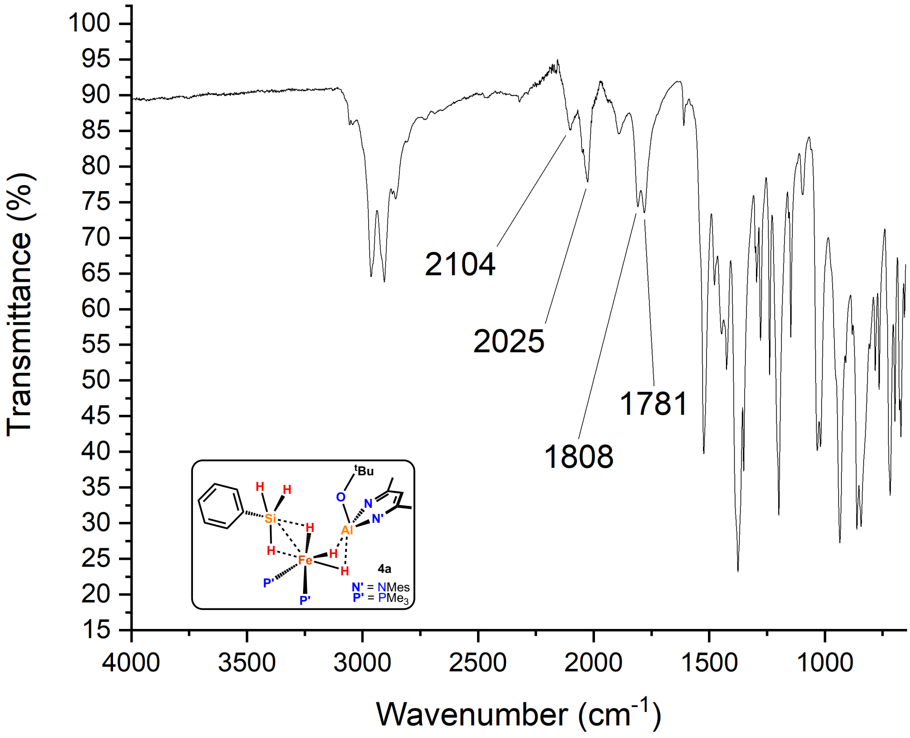

Figure S82 FT-IR spectrum of **4a** (thin film, 298 K, ATR).

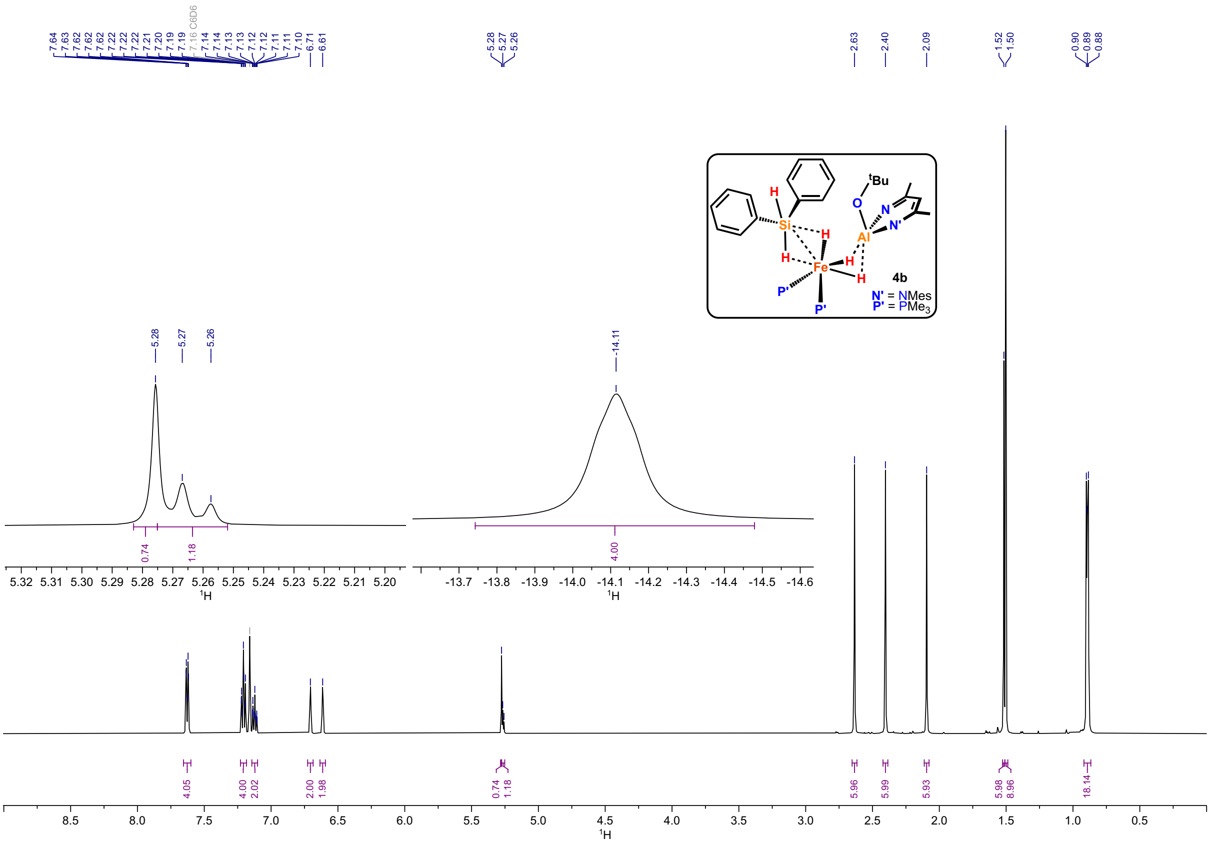

Figure S83 ^{1}H NMR spectrum of **4b** (C_6D_6, 298 K, 500 MHz).

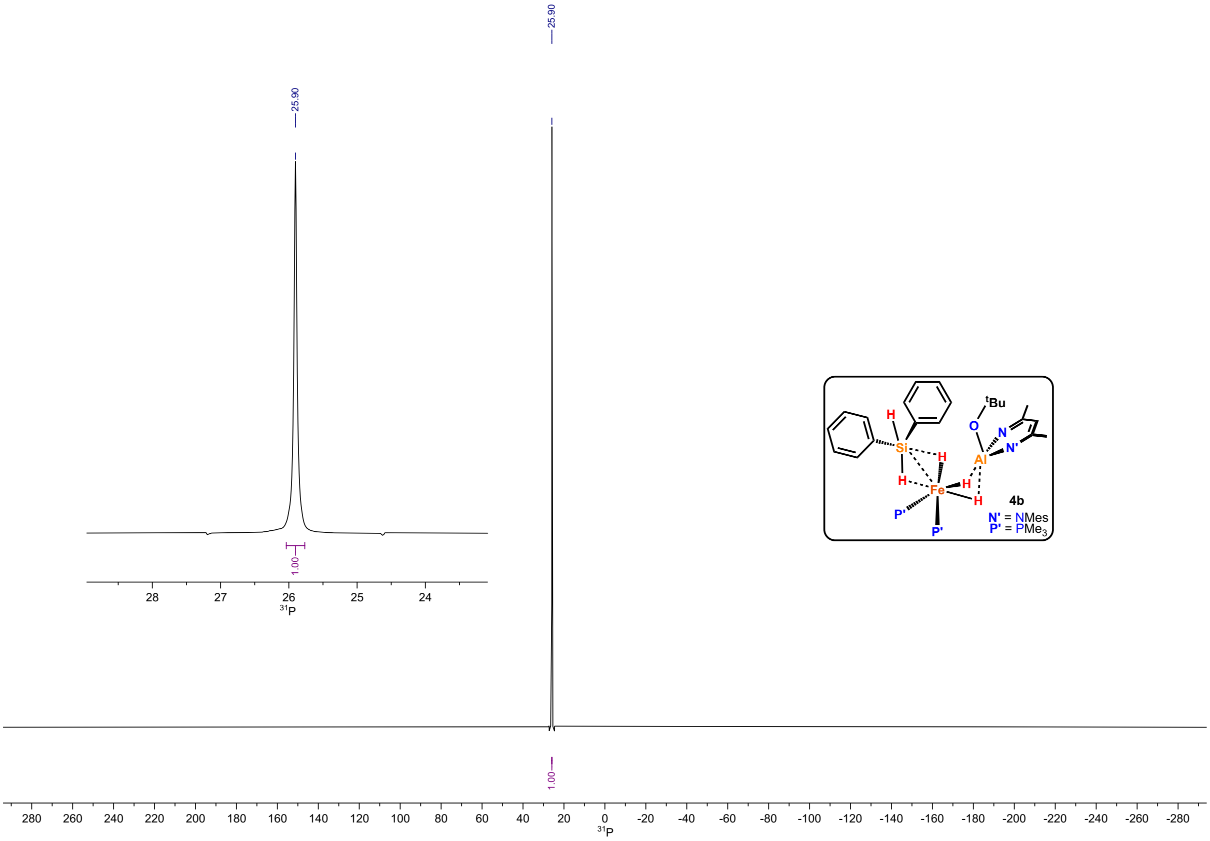

Figure S84 ^{31}P{^{1}H NMR spectrum of **4b** (C_6D_6, 298 K, 202 MHz).

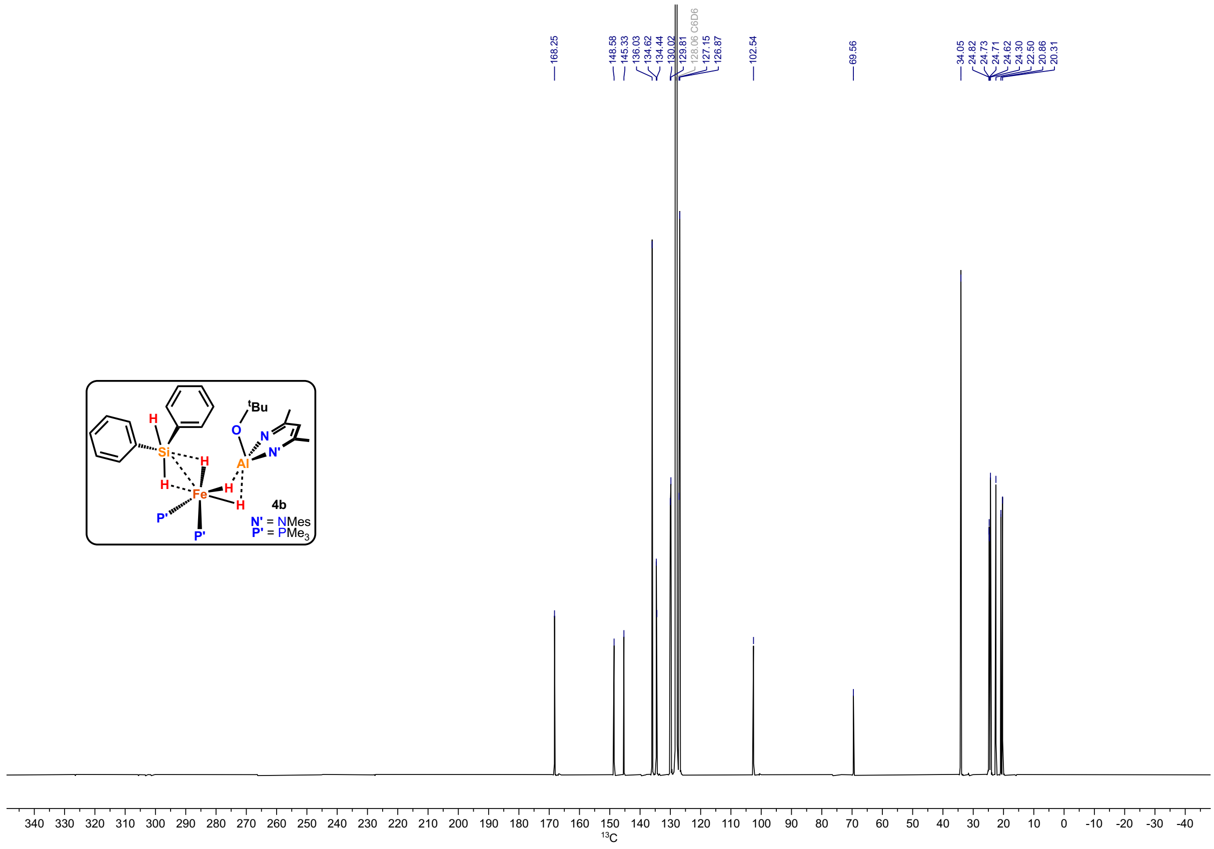

Figure S85 $^{13}C\{^{1}H\}$ NMR spectrum of **4b** (C_6D_6, 298 K, 126 MHz).

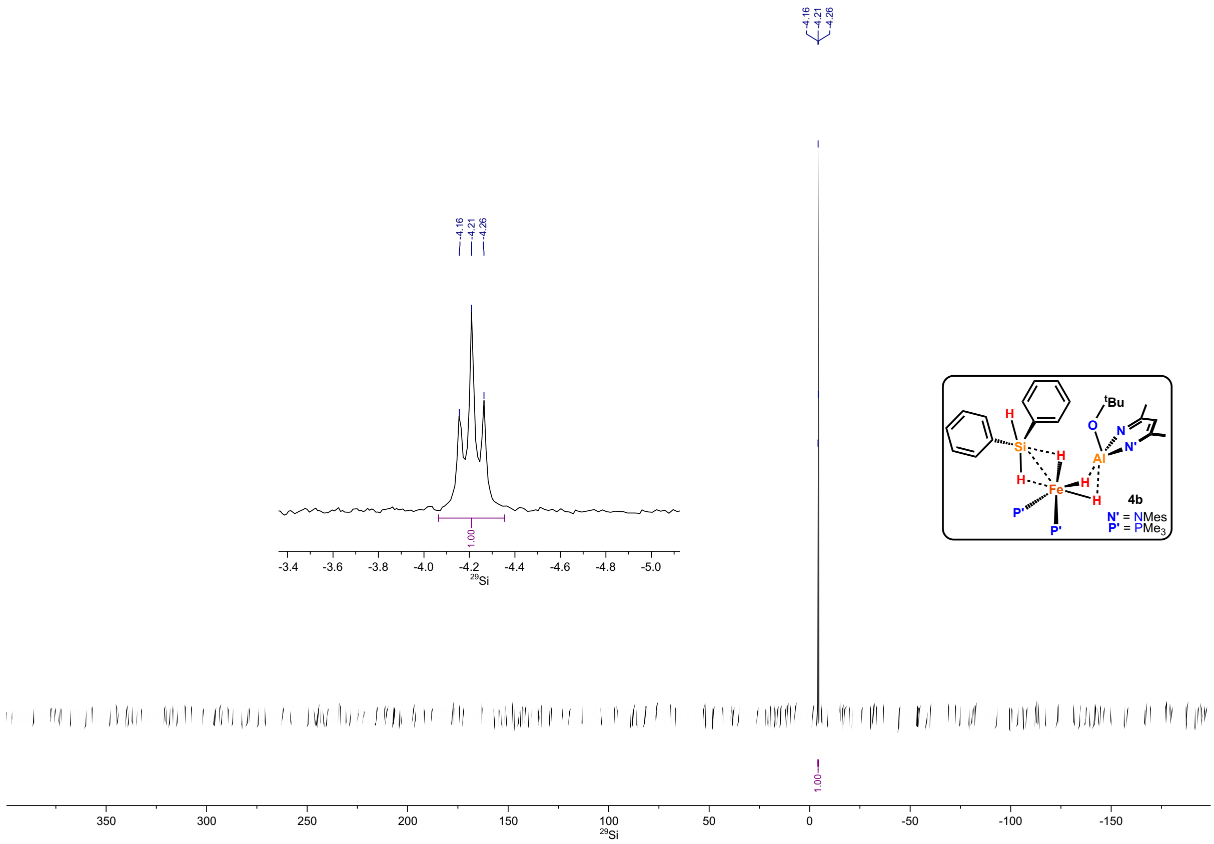

Figure S86 $^{29}Si\{^{1}H\}$ NMR spectrum of **4b** (C_6D_6, 298 K, 99 MHz, inverse gated).

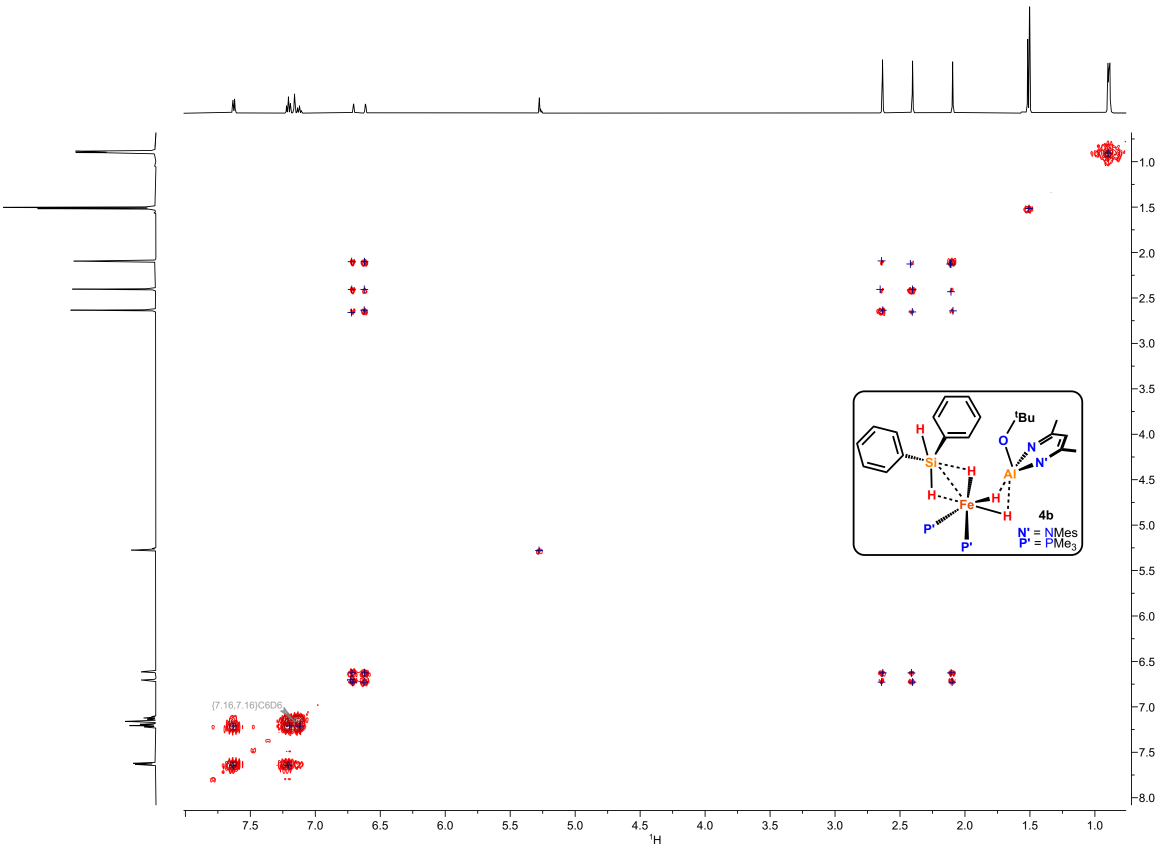

Figure S87 ^{1}H-^{1}H COSY NMR spectrum of **4b** (C_6D_6, 298 K, 500 MHz).

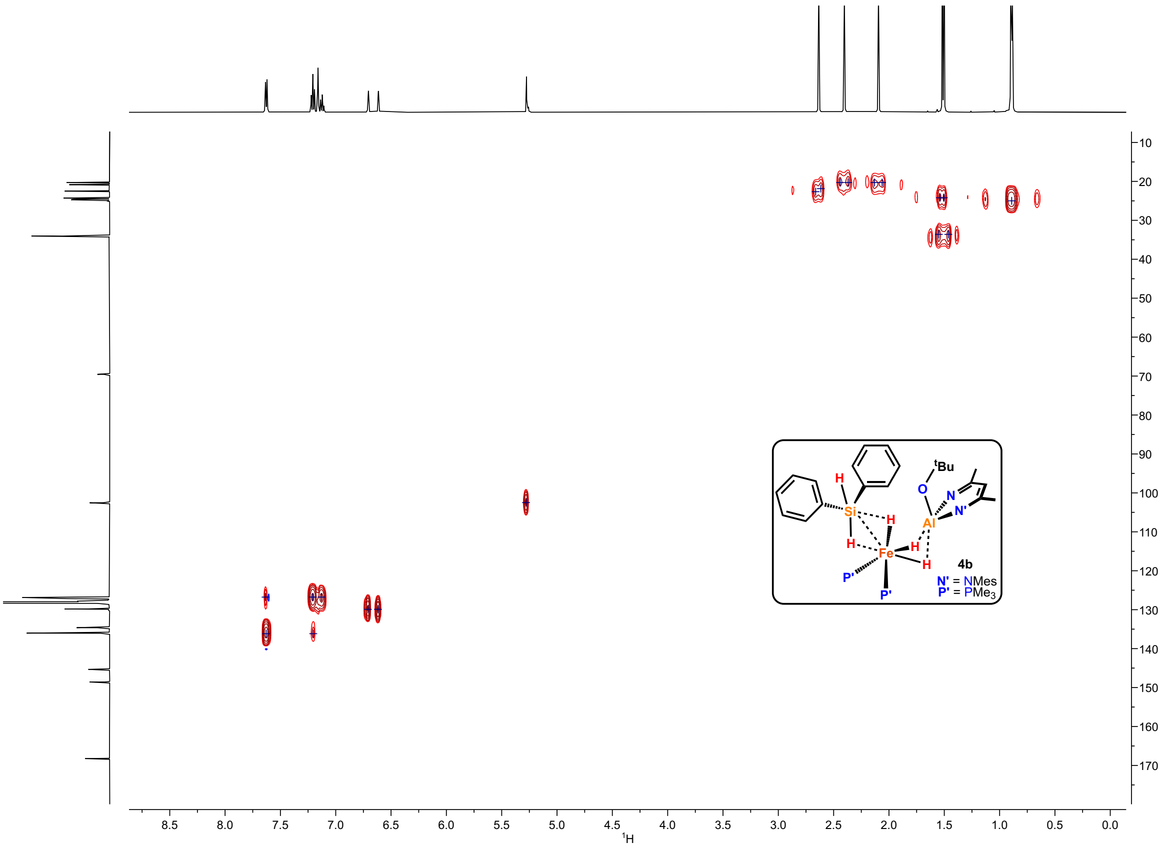

Figure S88 ^{1}H-^{13}C HSQC NMR spectrum of **4b** (C_6D_6, 298 K, 500 MHz).

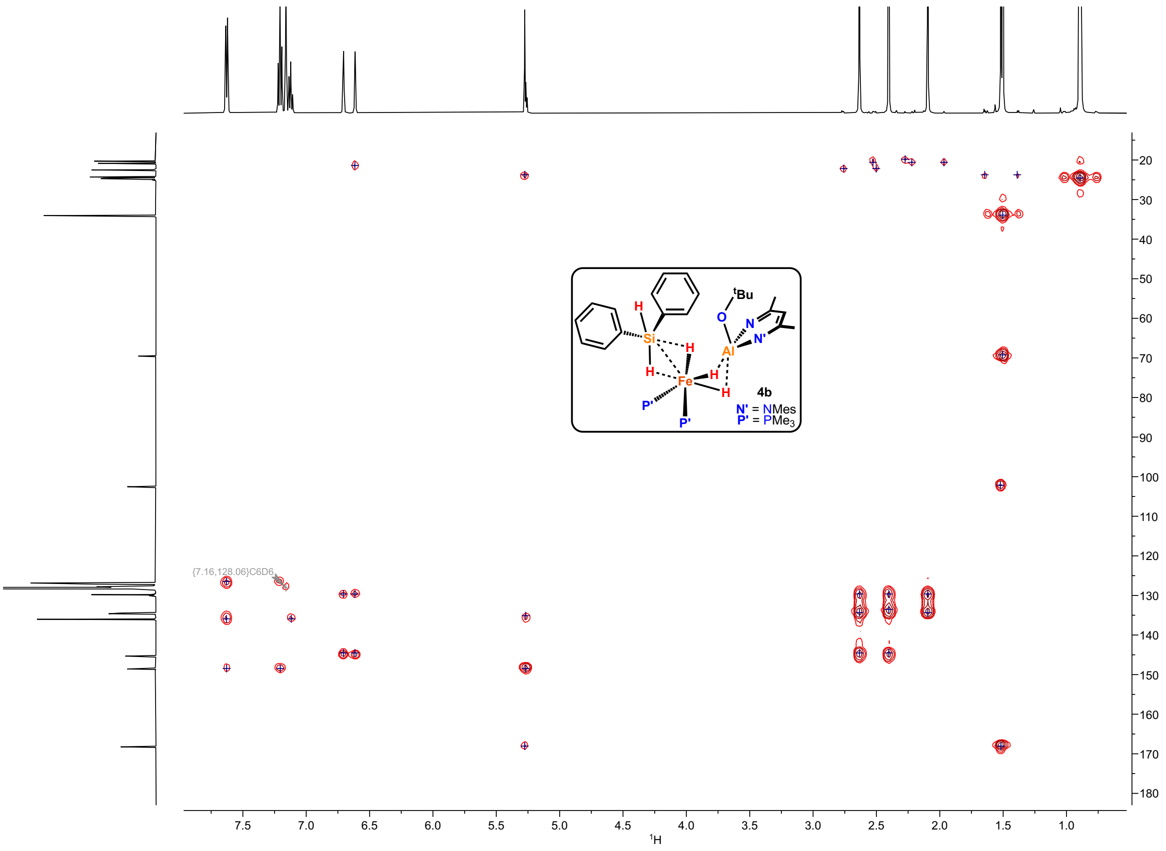

Figure S89 ^{1}H-^{1}H HMBC NMR spectrum of **4b** (C_6D_6, 298 K, 500 MHz).

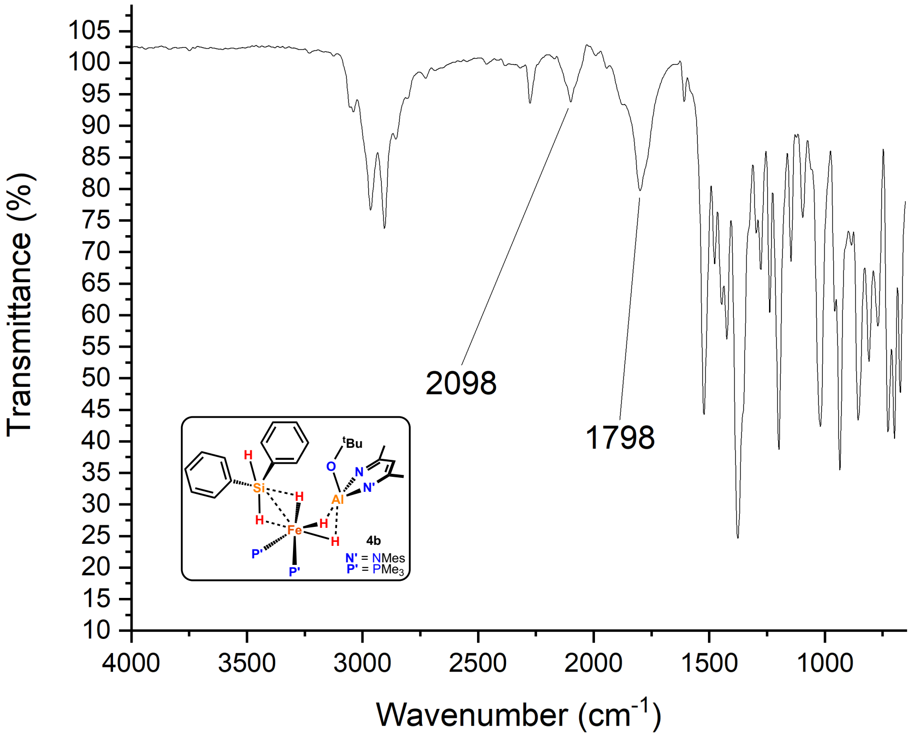

Figure S90 FT-IR spectrum of **4b** (thin film, 298 K, ATR).

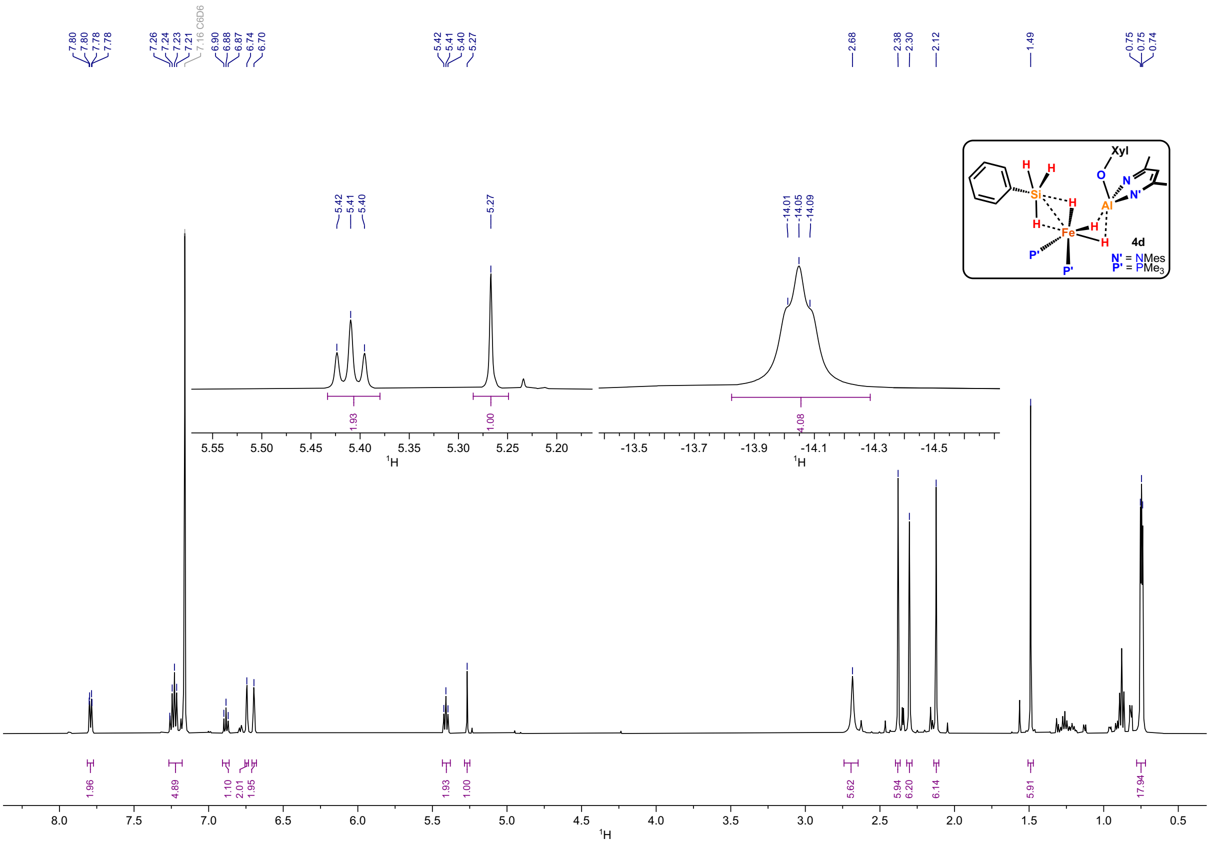

Figure S91 ^{1}H NMR spectrum of **4d** (C_6D_6, 298 K, 500 MHz).

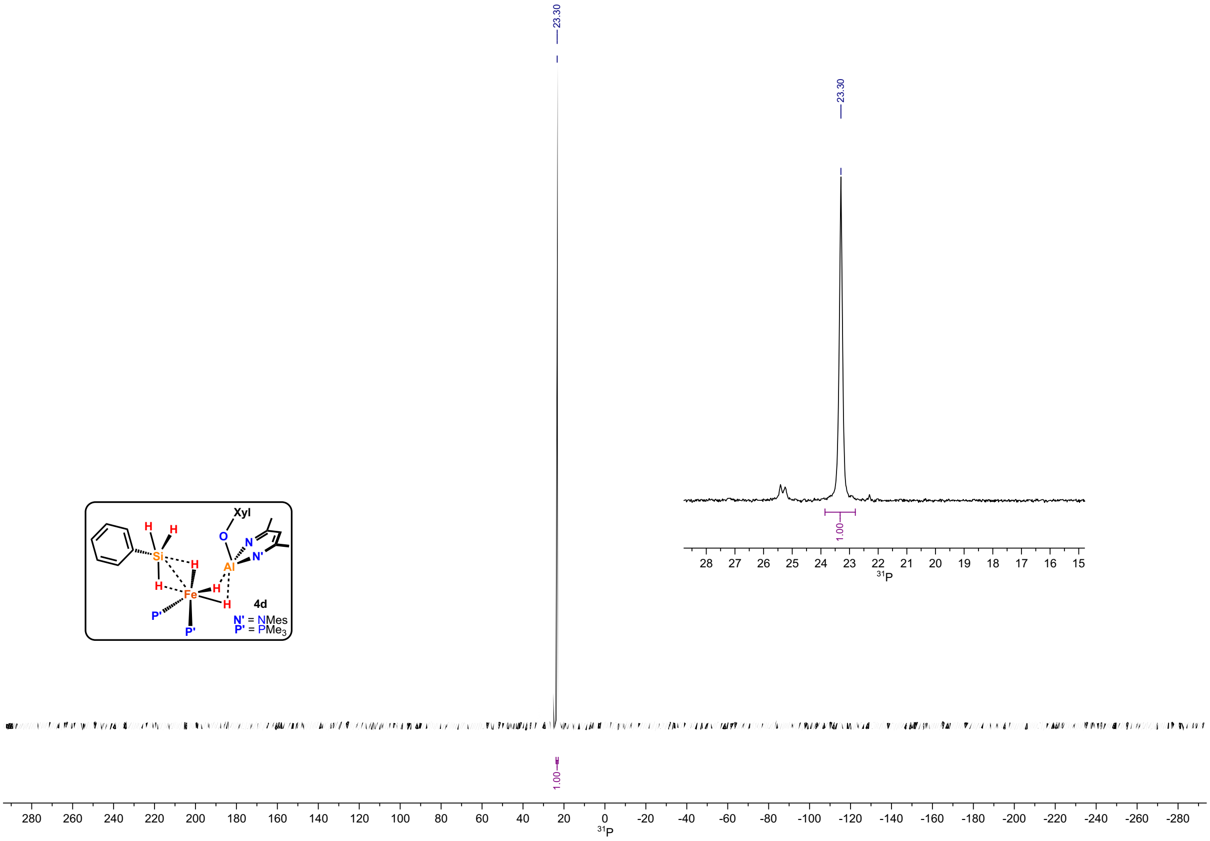

Figure S92 $^{31}P\{^{1}H\}$ NMR spectrum of **4d** (C_6D_6, 298 K, 202 MHz).

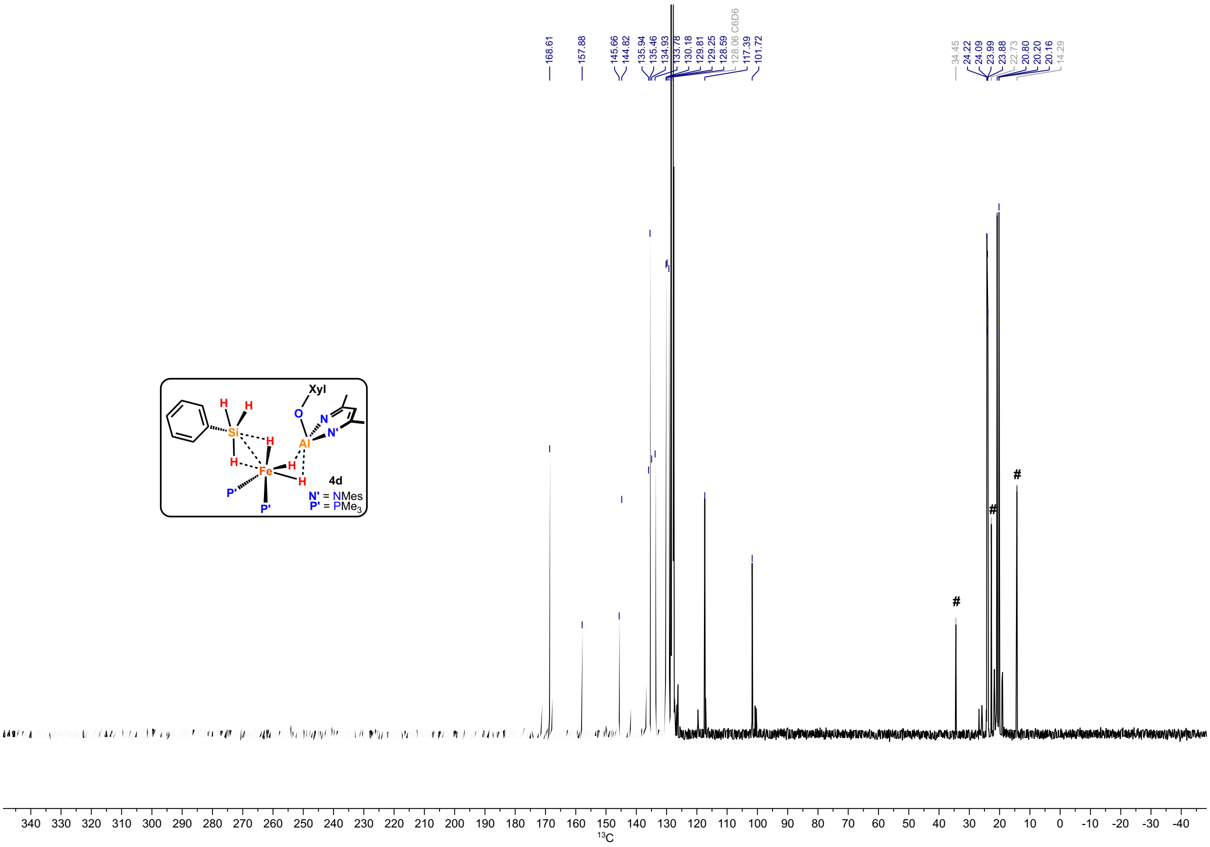

Figure S93 $^{13}C\{^{1}H\}$ NMR spectrum of **4d** (C_6D_6, 298 K, 126 MHz). #: *n*-pentane, other weak resonances: unknown impurities due to decomposition.

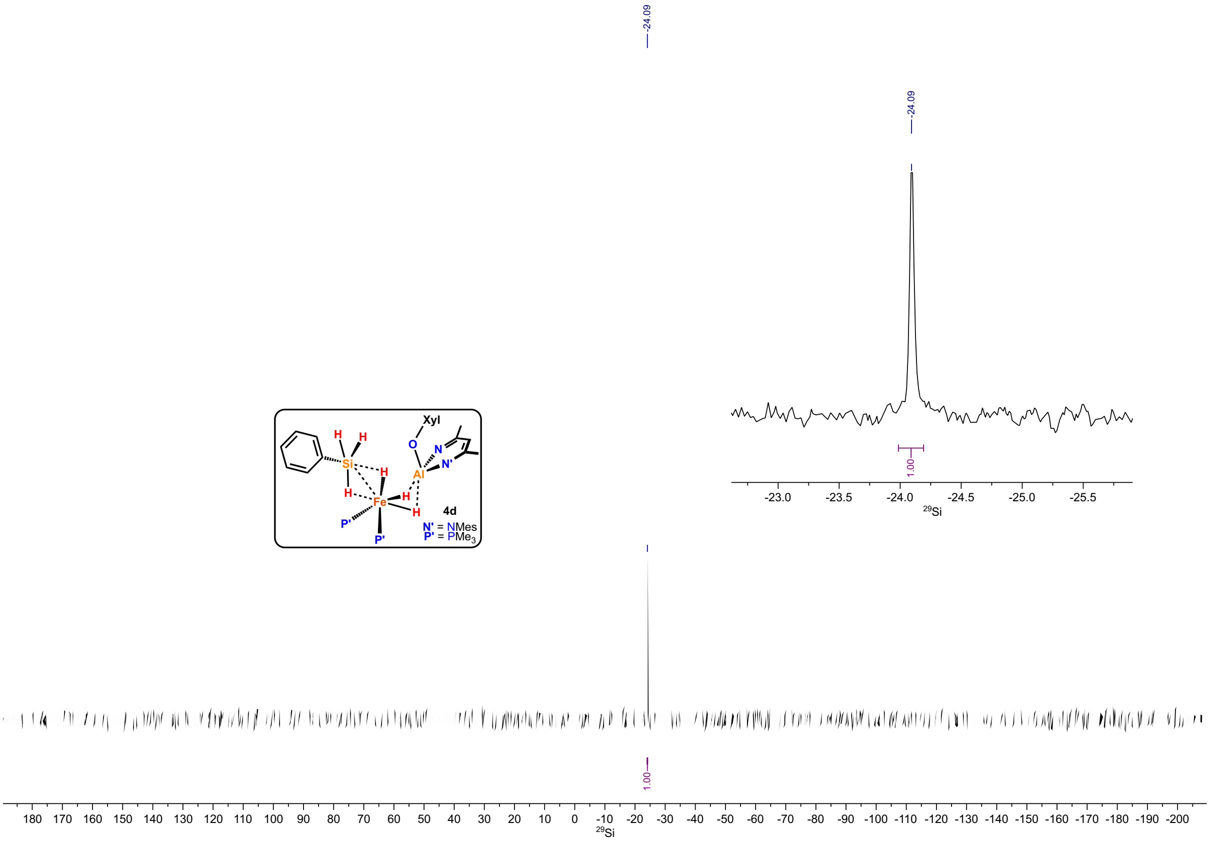

Figure S94 $^{29}Si\{^{1}H\}$ NMR spectrum of **4d** (C_6D_6, 298 K, 99 MHz, inverse gated).

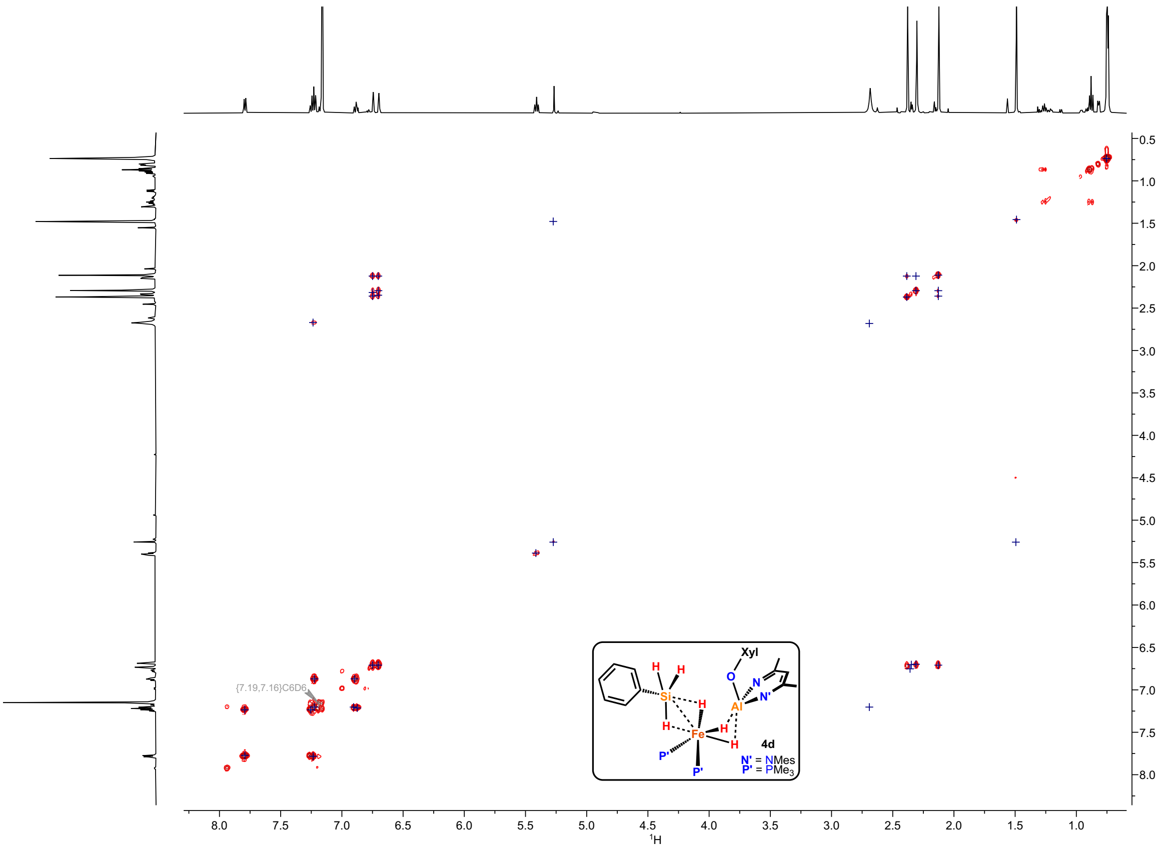

Figure S95 ^{1}H-^{1}H COSY NMR spectrum of **4d** (C_6D_6, 298 K, 500 MHz).

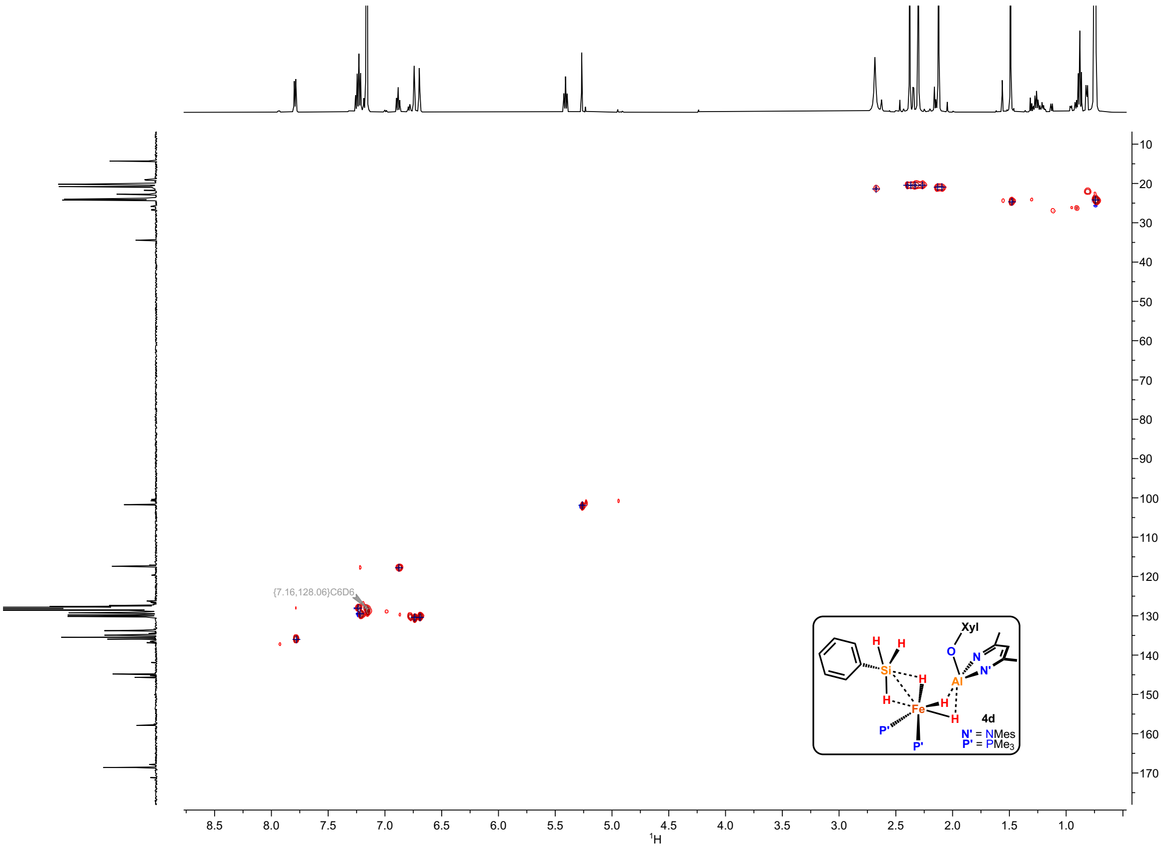

Figure S96 ^{1}H-^{13}C HSQC NMR spectrum of **4d** (C_6D_6, 298 K, 500 MHz).

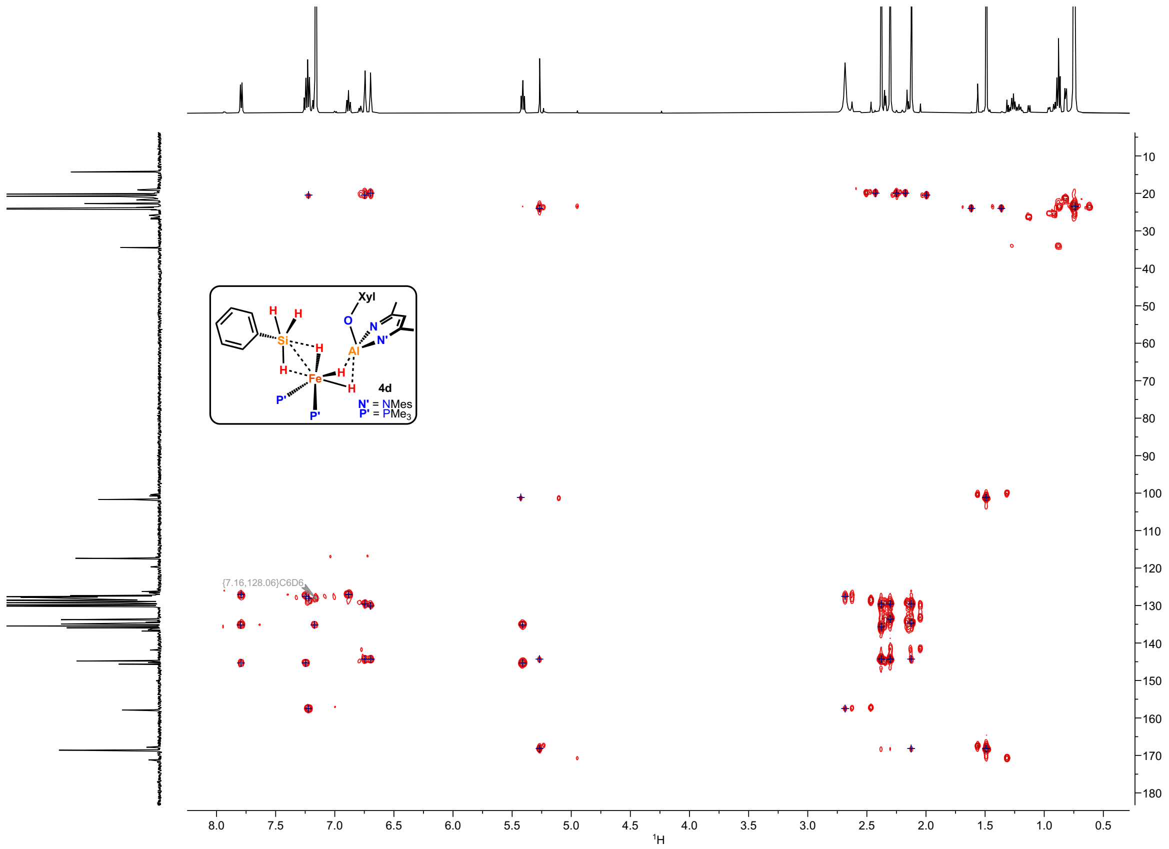

Figure S97 ^{1}H-^{13}C HMBC NMR spectrum of **4d** (C_6D_6, 298 K, 500 MHz).

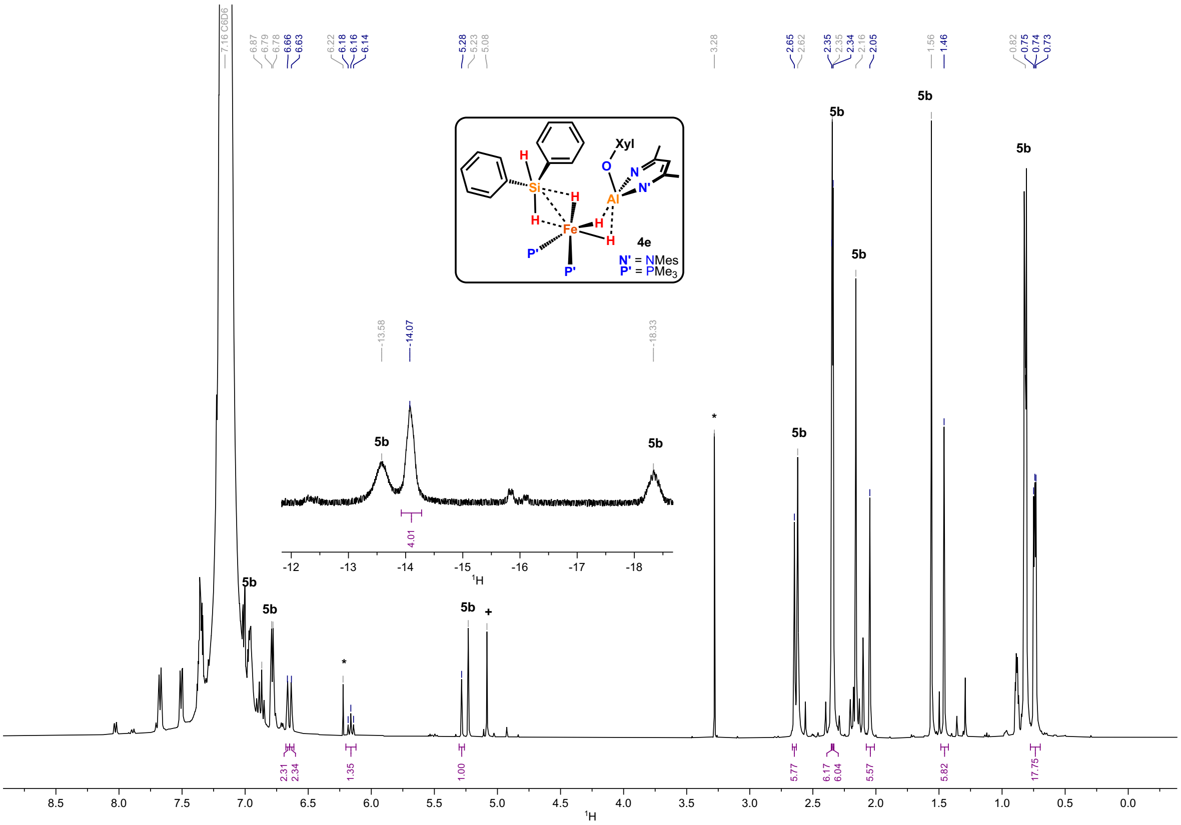

Figure S98 *In situ* ^{1}H NMR of **4e** (C_6D_6/C_6H_6, 298 K, 400 MHz). *: 1,3,5-trimethoxybenzene (internal standard in capillary), +: Ph_2SiH_2, the sample contains the corresponding dinitrogen complex **5b**, unmarked minor peaks: unidentified impurities.

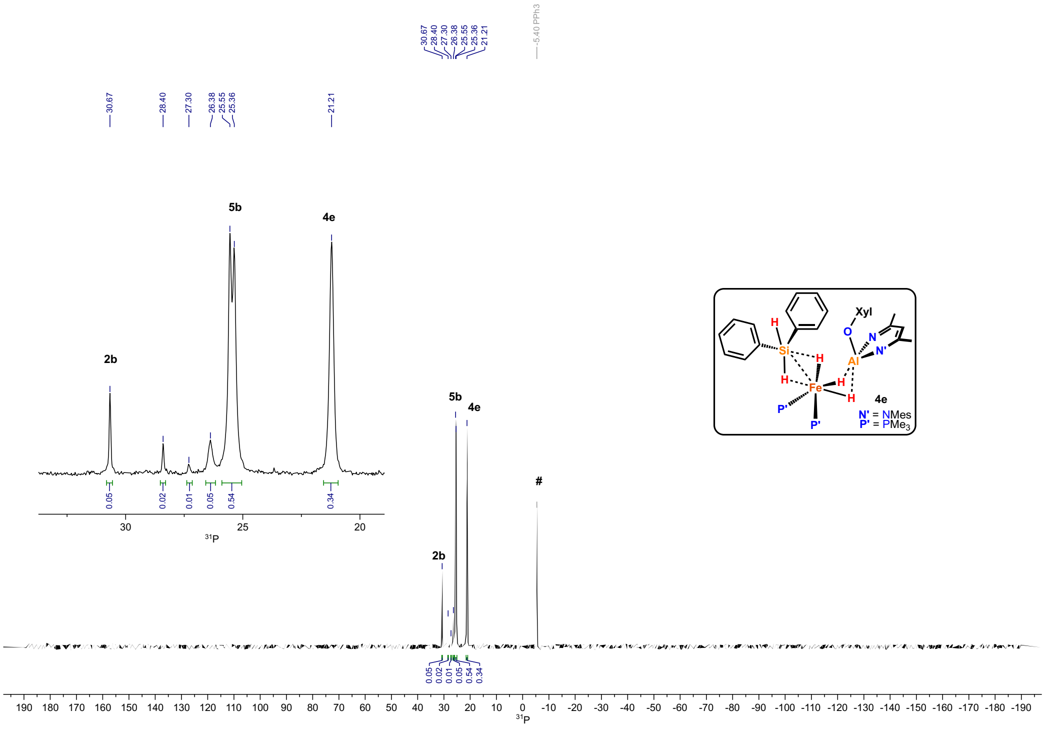

Figure S99 *In situ* ^{31}P{^{1}H} NMR of **4e** (C_6D_6/C_6H_6, 298 K, 400 MHz). #: PPh_3 (internal standard in capillary), the sample contains the corresponding silanide complex **2b** and dinitrogen complex **5b**, unmarked minor peaks: unidentified impurities. Nb. **5b** appears as a doublet in this spectrum due to insufficient decoupling from the hydrides.

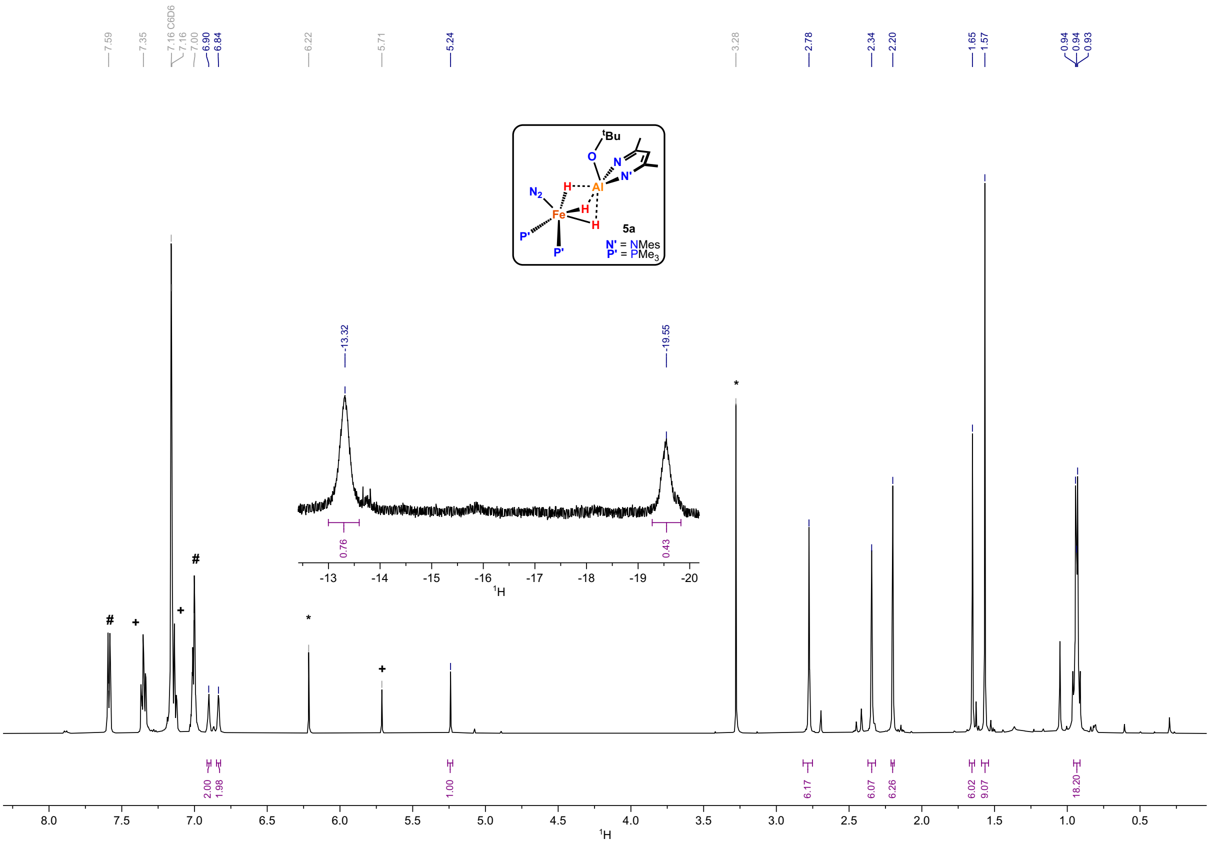

Figure S100 *In situ* ^{1}H NMR spectrum of **5a** (C_6D_6, 298 K, 500 MHz). #: PPh_3 (internal standard in capillary), *: 1,3,5-trimethoxybenzene (internal standard in capillary), +: triphenylsilane.

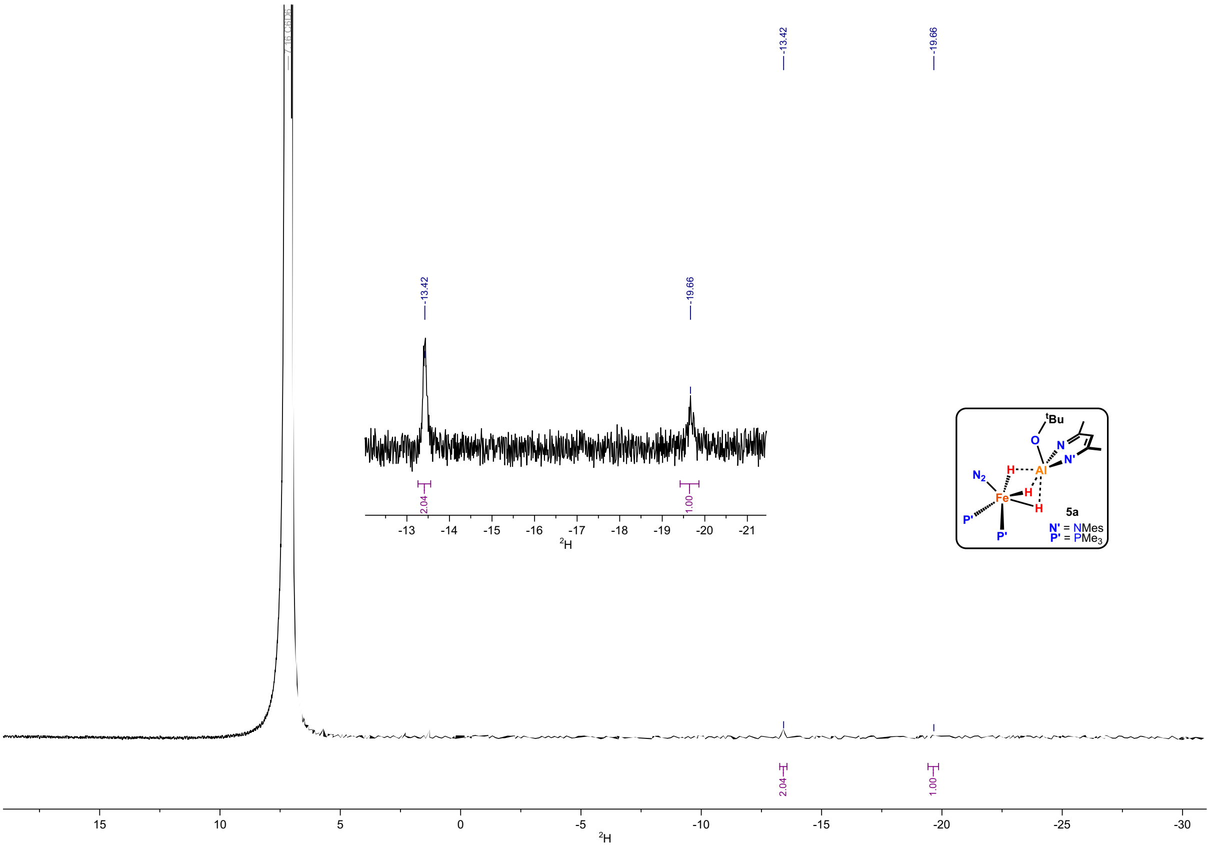

Figure S101 *In situ* ^{2}H NMR spectrum of **5a** (C_6D_6, 298 K, 500 MHz).

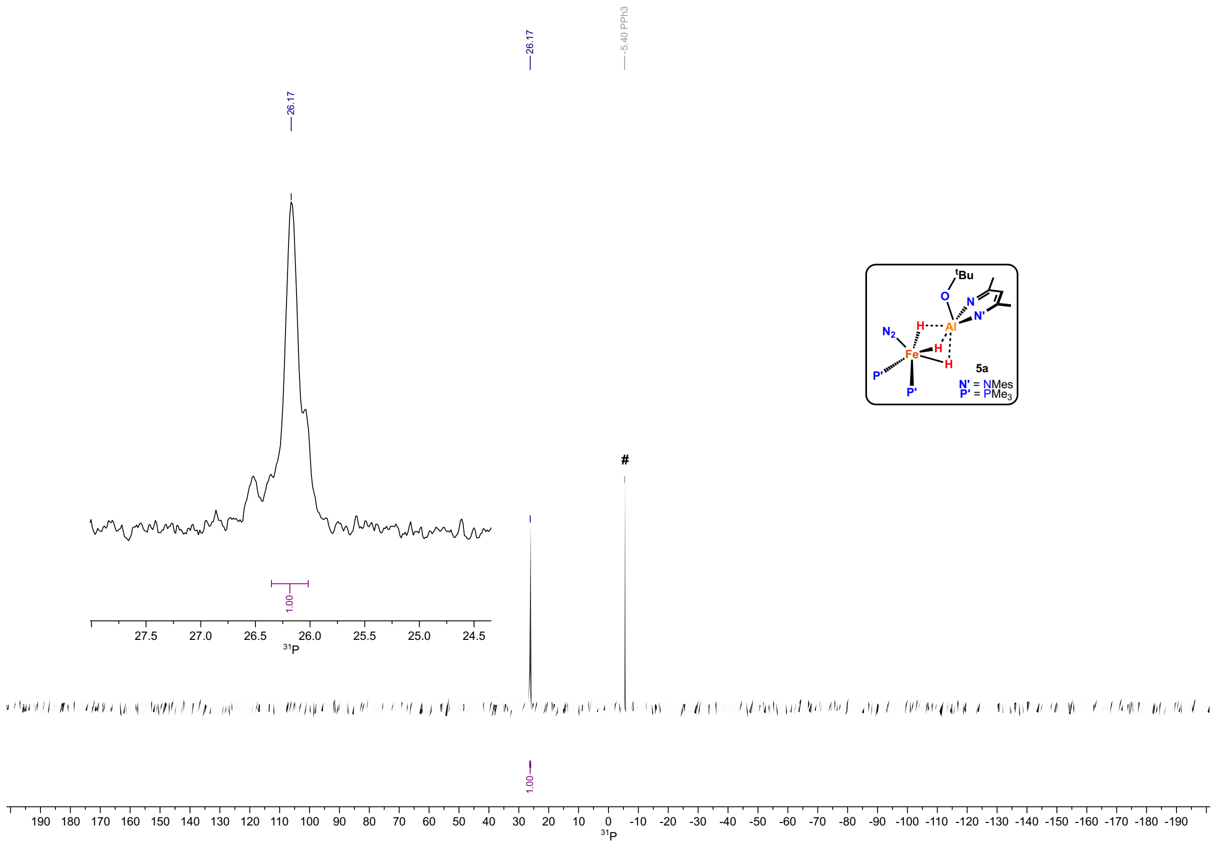

Figure S102 *In situ* $^{31}P\{^{1}H\}$ NMR spectrum of **5a** (C_6D_6, 298 K, 500 MHz). #: PPh_3 (internal standard in capillary).

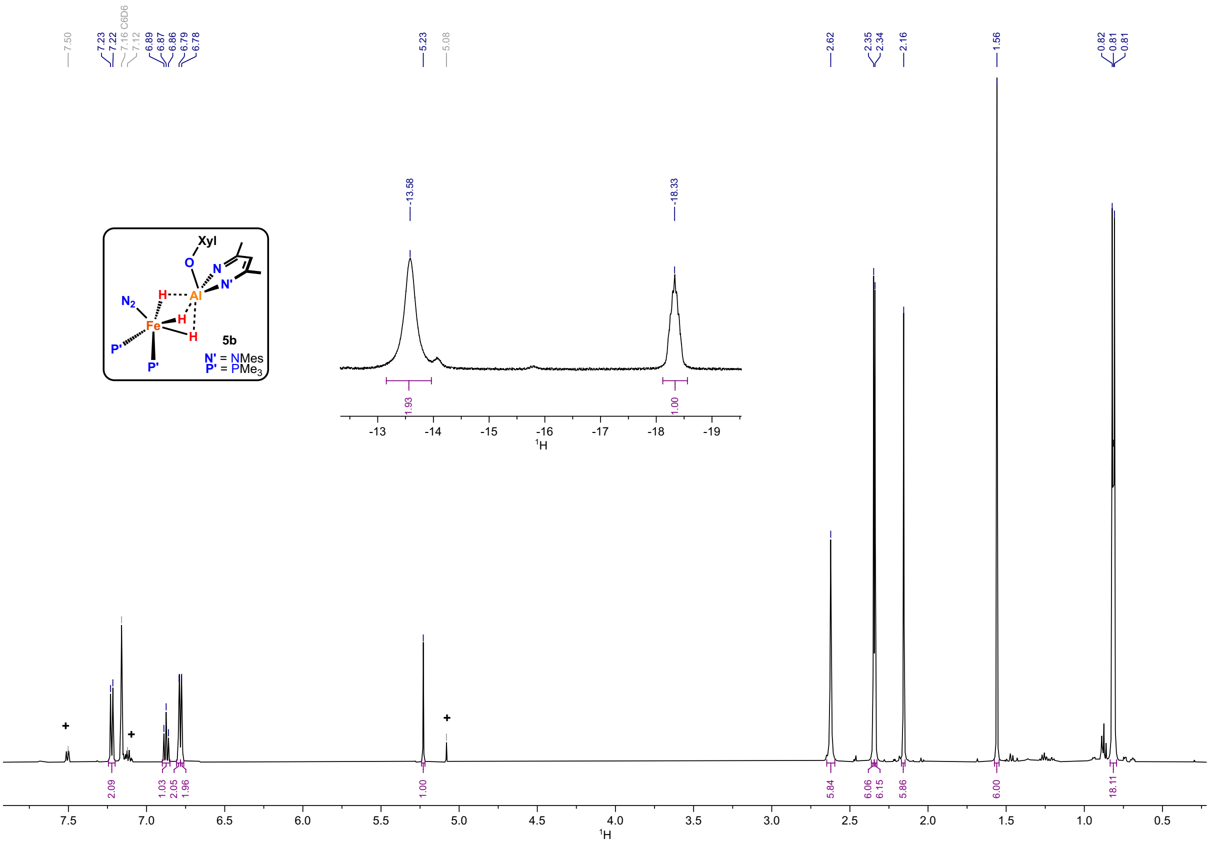

Figure S103 ^{1}H NMR spectrum of **5b** (C_6D_6, 298 K, 500 MHz). +: Ph_2SiH_2.

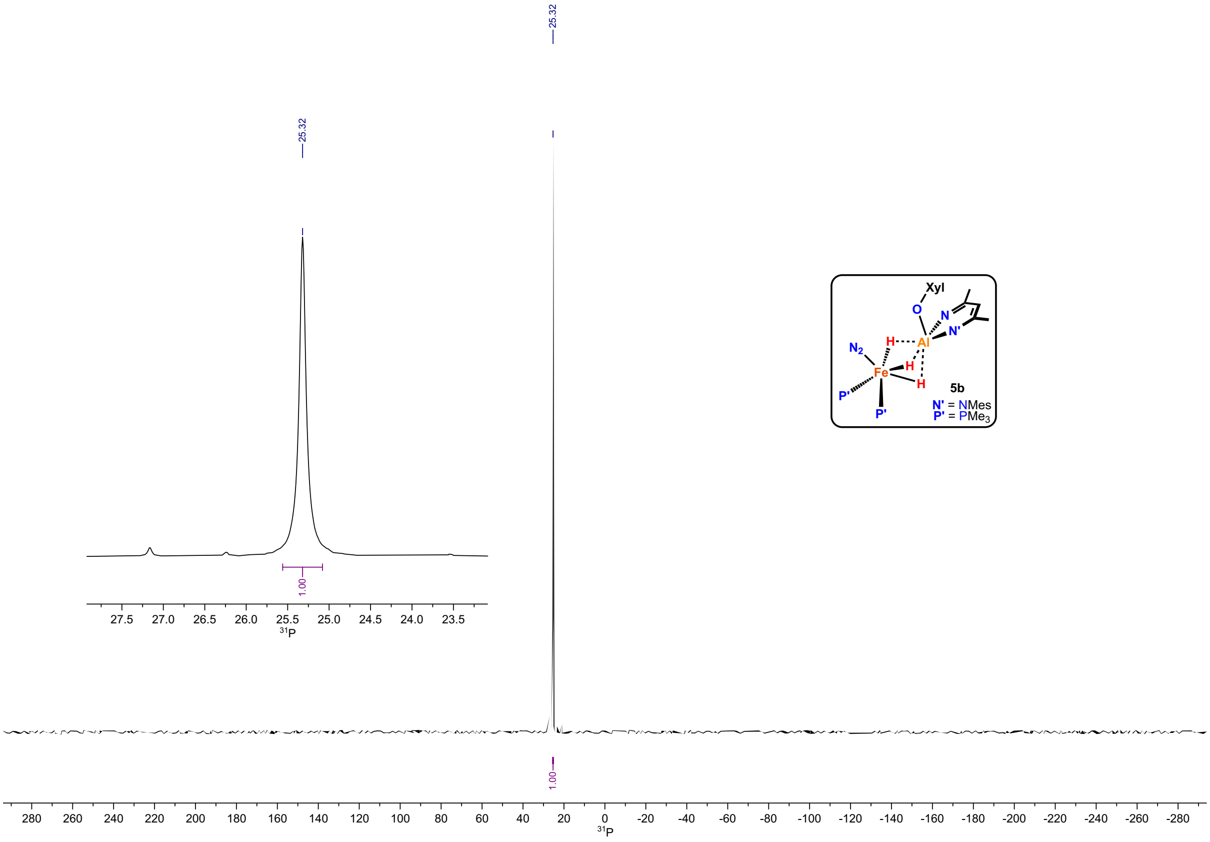

Figure S104 $^{31}P\{^{1}H\}$ NMR spectrum of **5b** (C_6D_6, 298 K, 202 MHz).

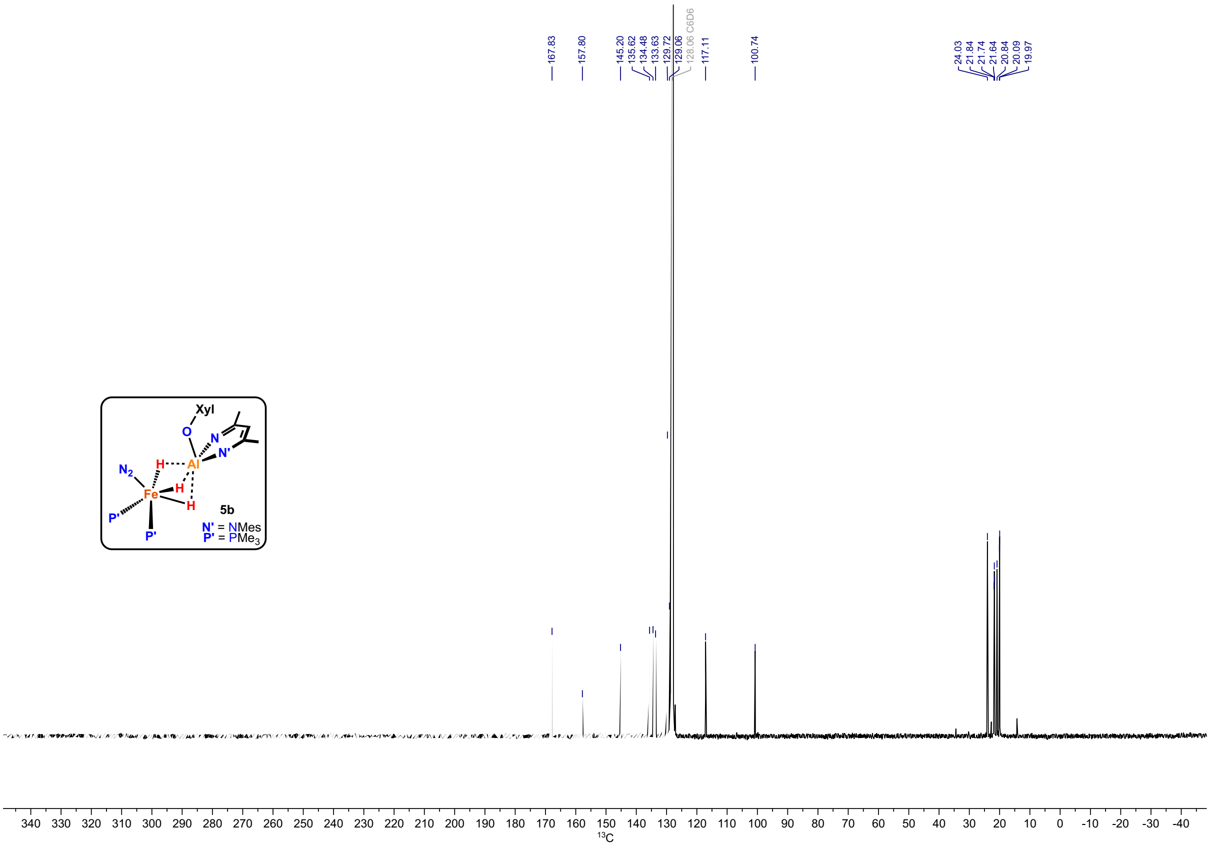

Figure S105 $^{13}C\{^{1}H$ NMR spectrum of **5b** (C_6D_6, 298 K, 126 MHz).

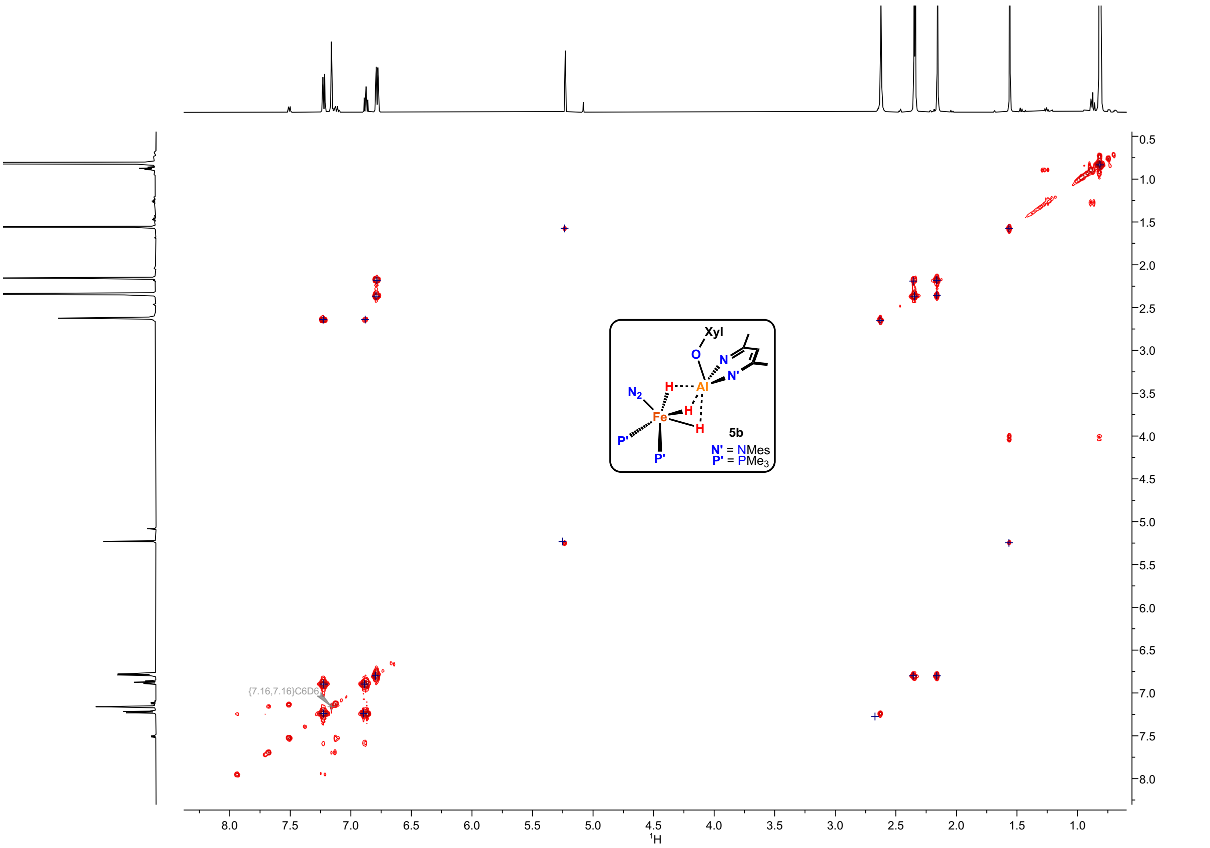

Figure S106 ^{1}H-^{1}H COSY NMR spectrum of **5b** (C_6D_6, 298 K, 500 MHz).

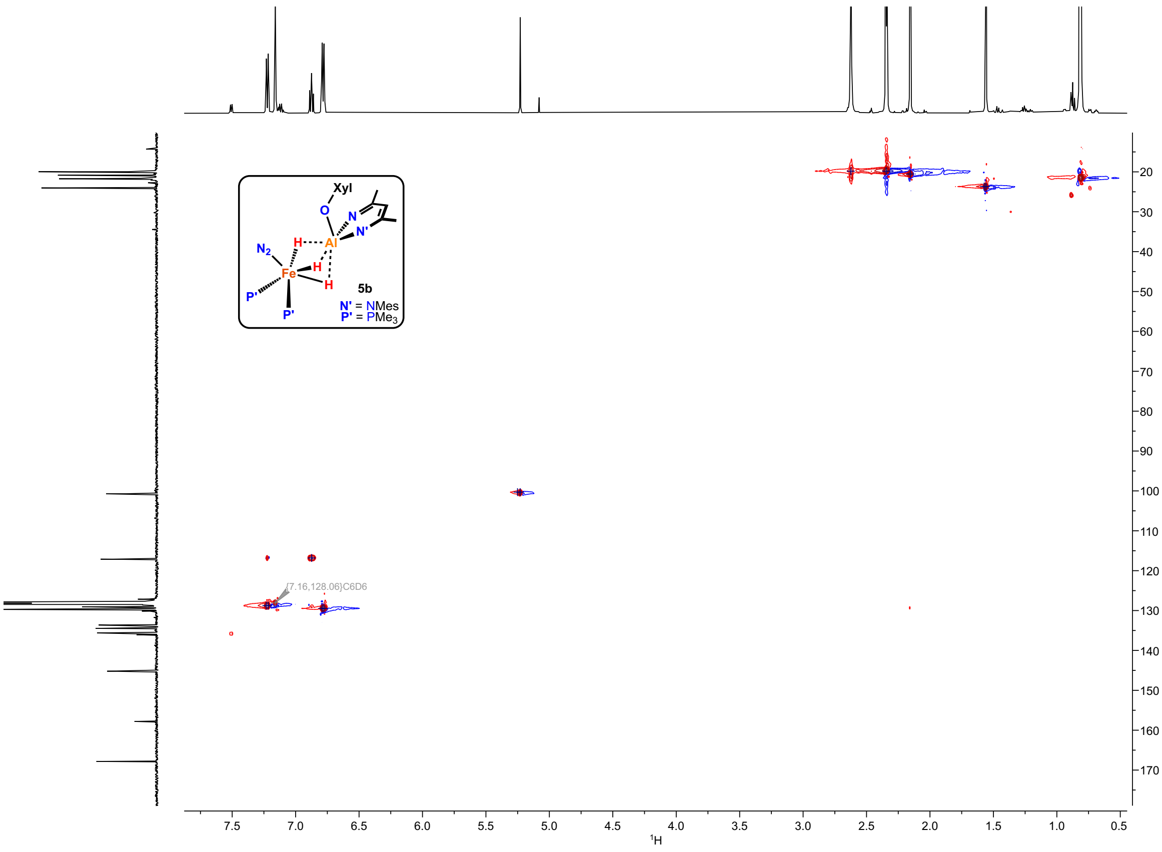

Figure S107 ^{1}H-^{13}C HSQC NMR spectrum of **5b** (C_6D_6, 298 K, 500 MHz).

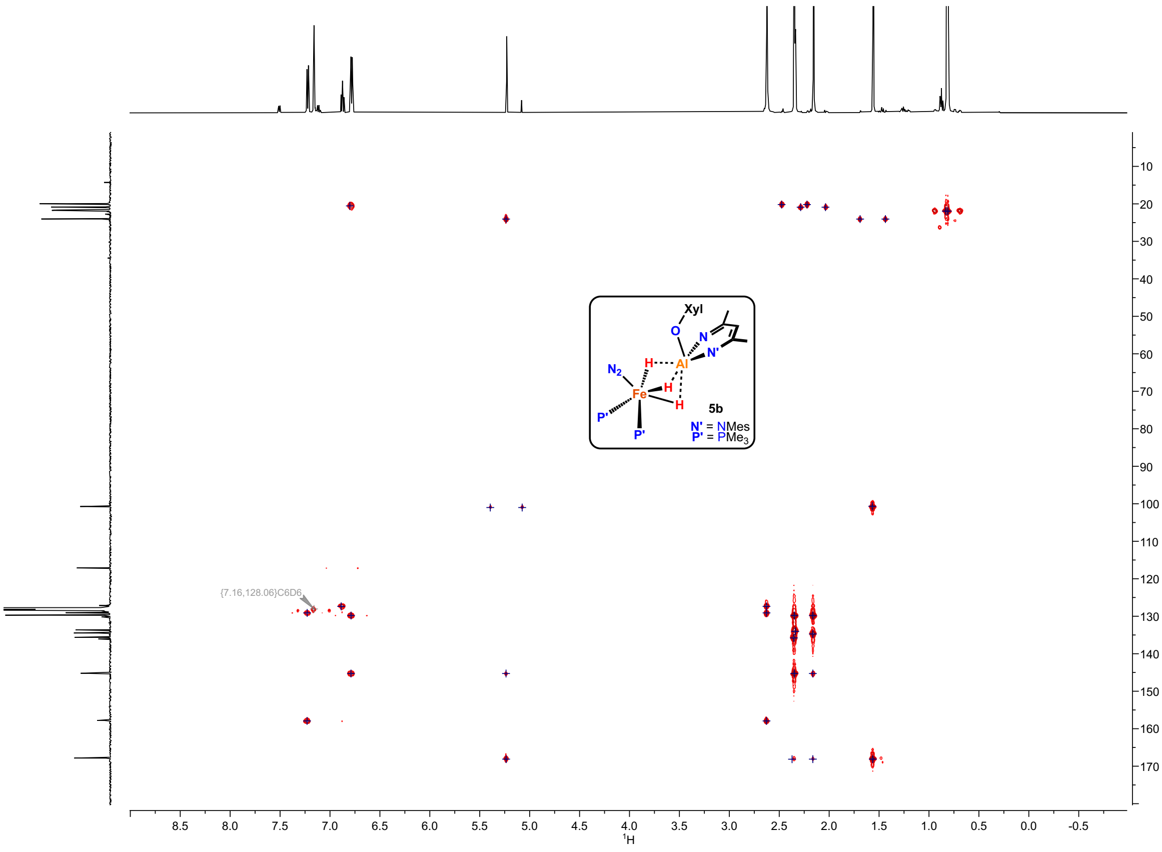

Figure S108 ^{1}H-^{13}C HMBC NMR spectrum of **5b** (C_6D_6, 298 K, 500 MHz).

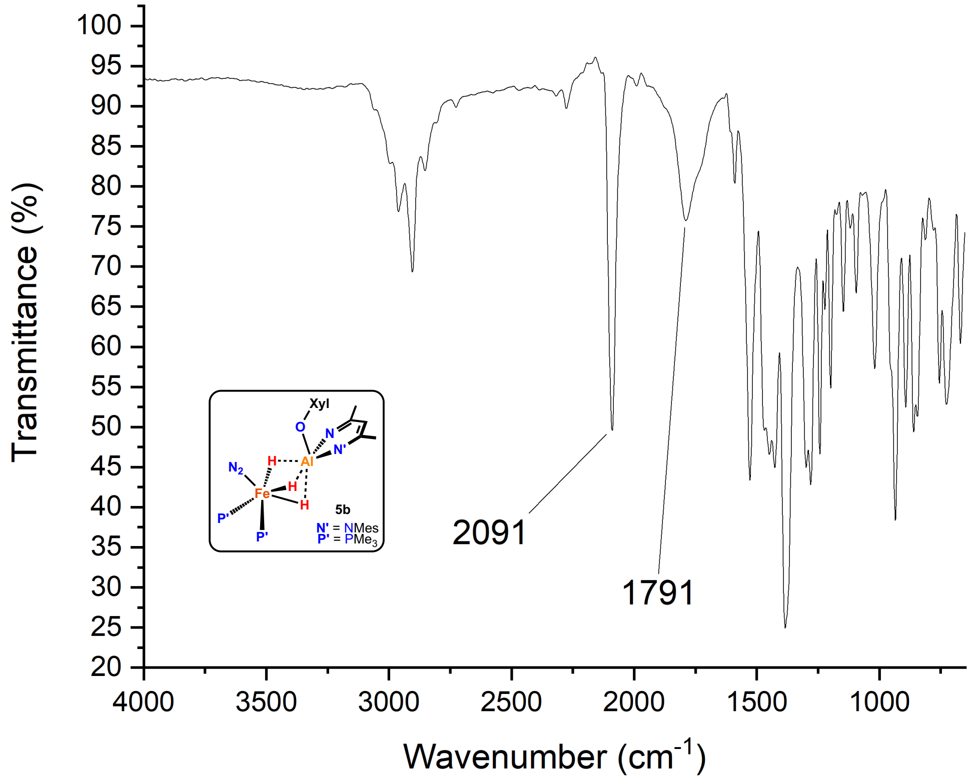

Figure S109 FT-IR spectrum of **5b** (thin film, 298 K, ATR).

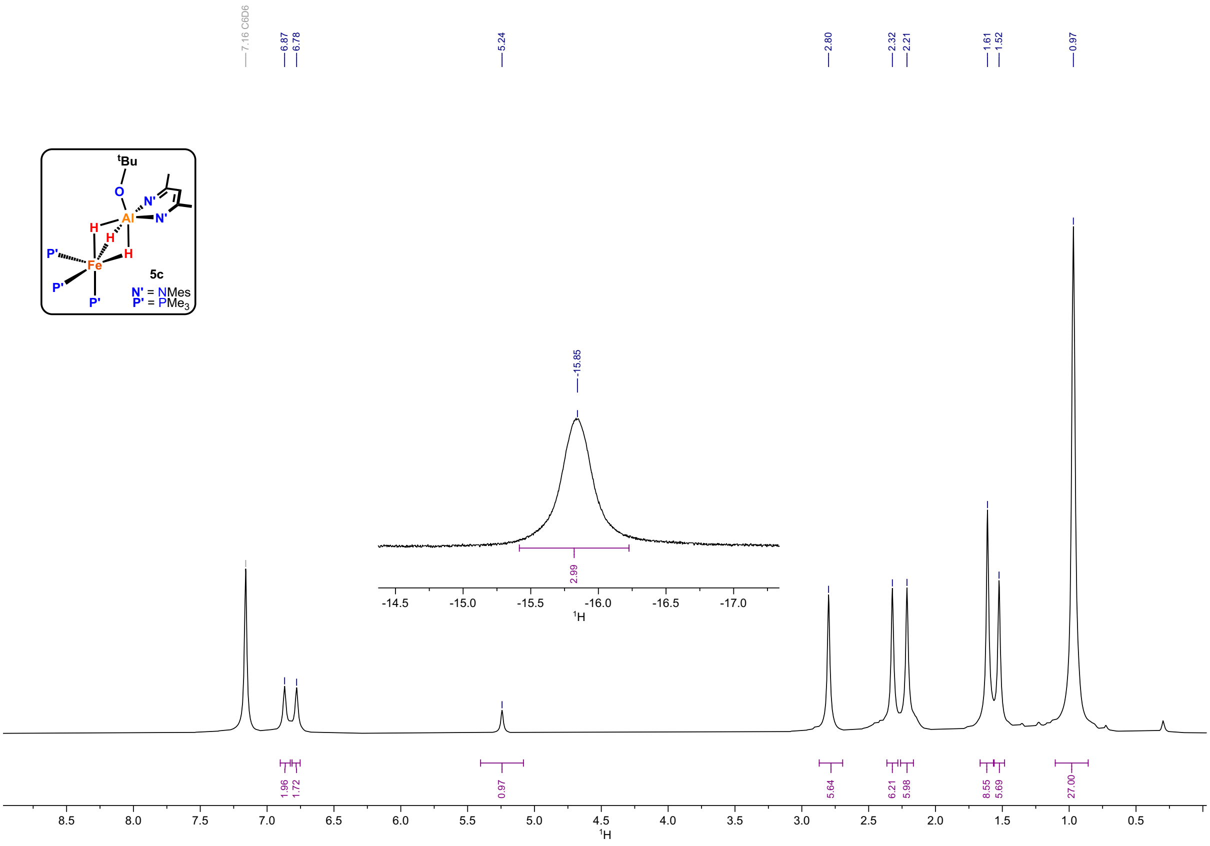

Figure S110 ^{1}H NMR spectrum of **5c** (C_6D_6, 298 K, 400 MHz).

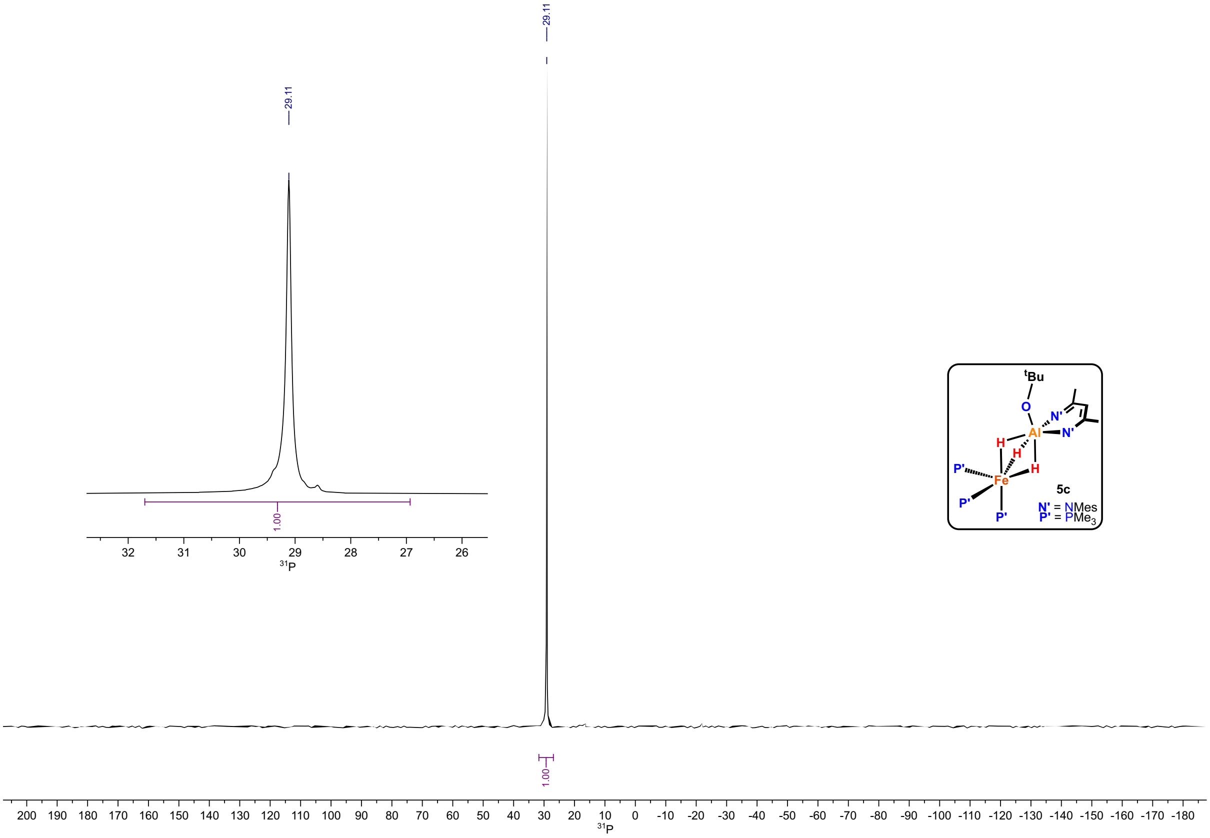

Figure S111 $^{31}P\{^{1}H\}$ NMR spectrum of **5c** (C_6D_6, 298 K, 162 MHz).

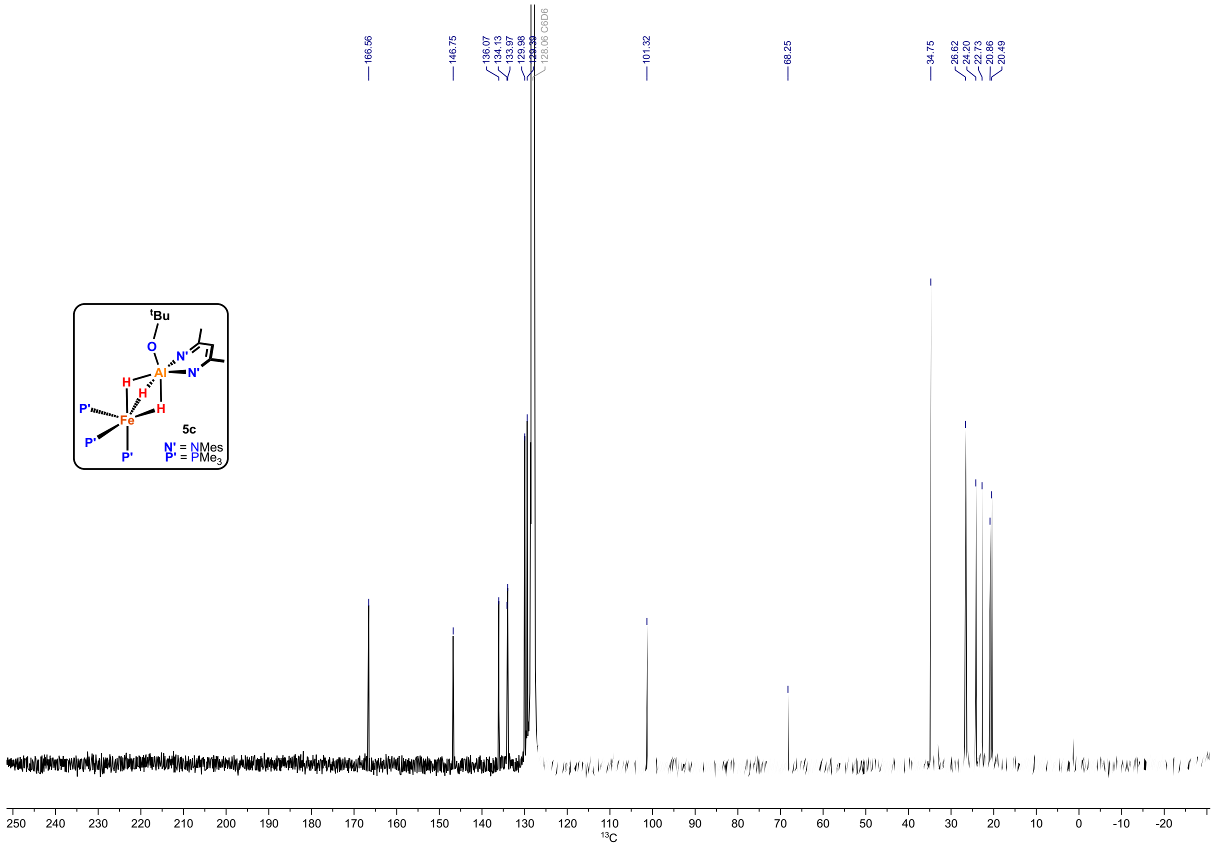

Figure S112 $^{13}C\{^{1}H\}$ NMR spectrum of **5c** (C_6D_6, 298 K, 101 MHz).

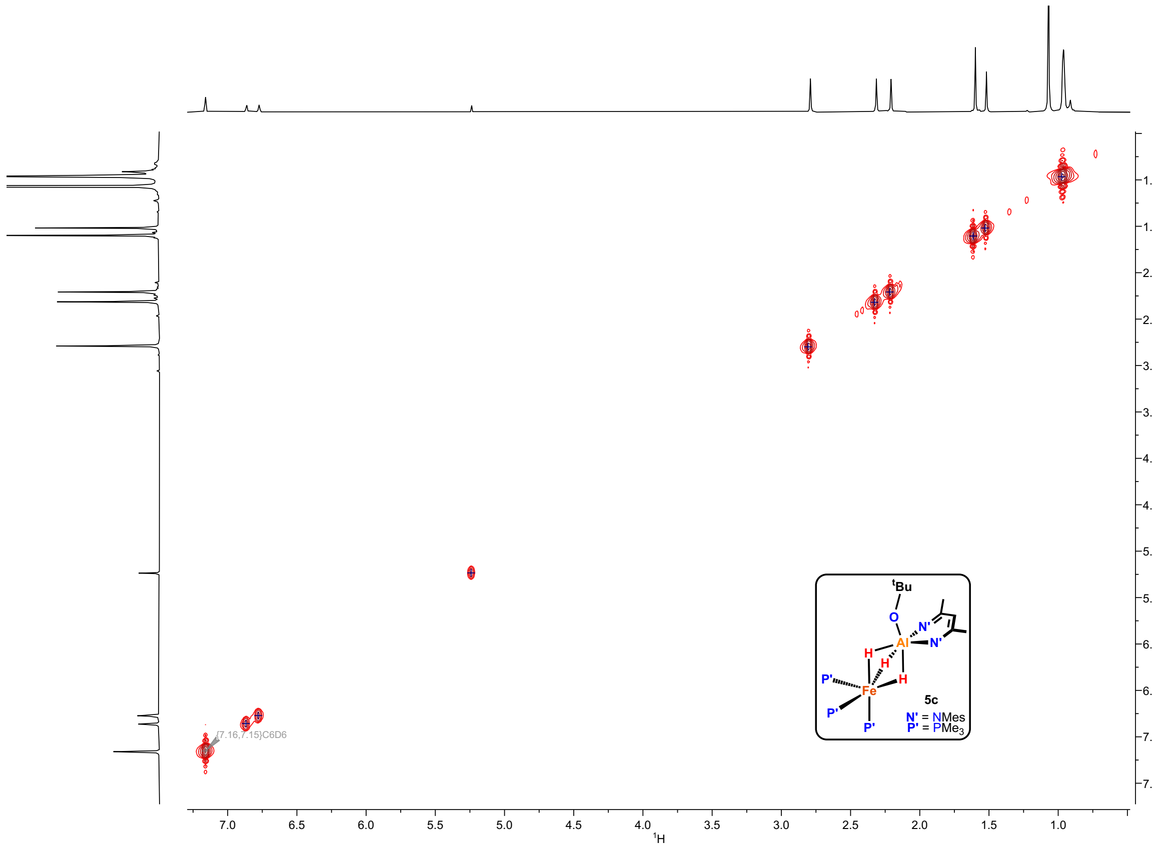

Figure S113 ^{1}H-^{1}H COSY NMR spectrum of **5c** (C_6D_6, 298 K, 400 MHz).

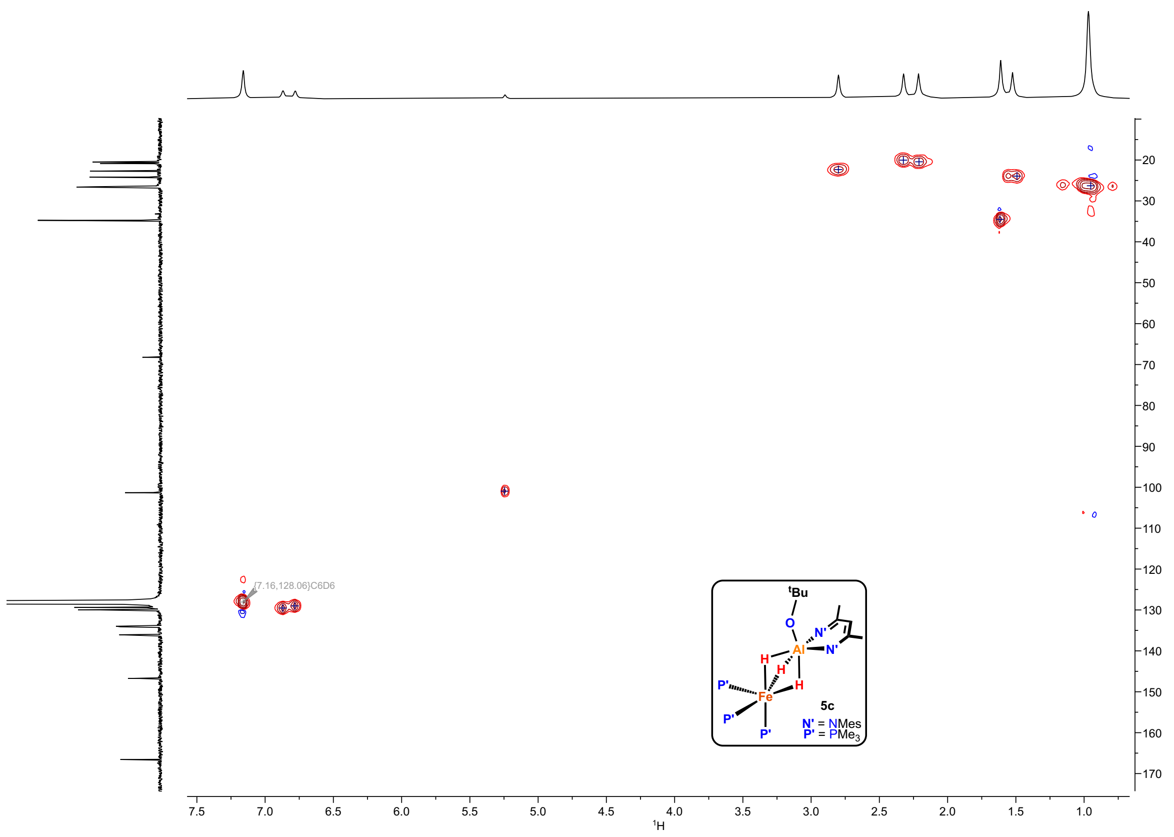

Figure S114 ^{1}H-^{13}C HSQC NMR spectrum of **5c** (C_6D_6, 298 K, 400 MHz).

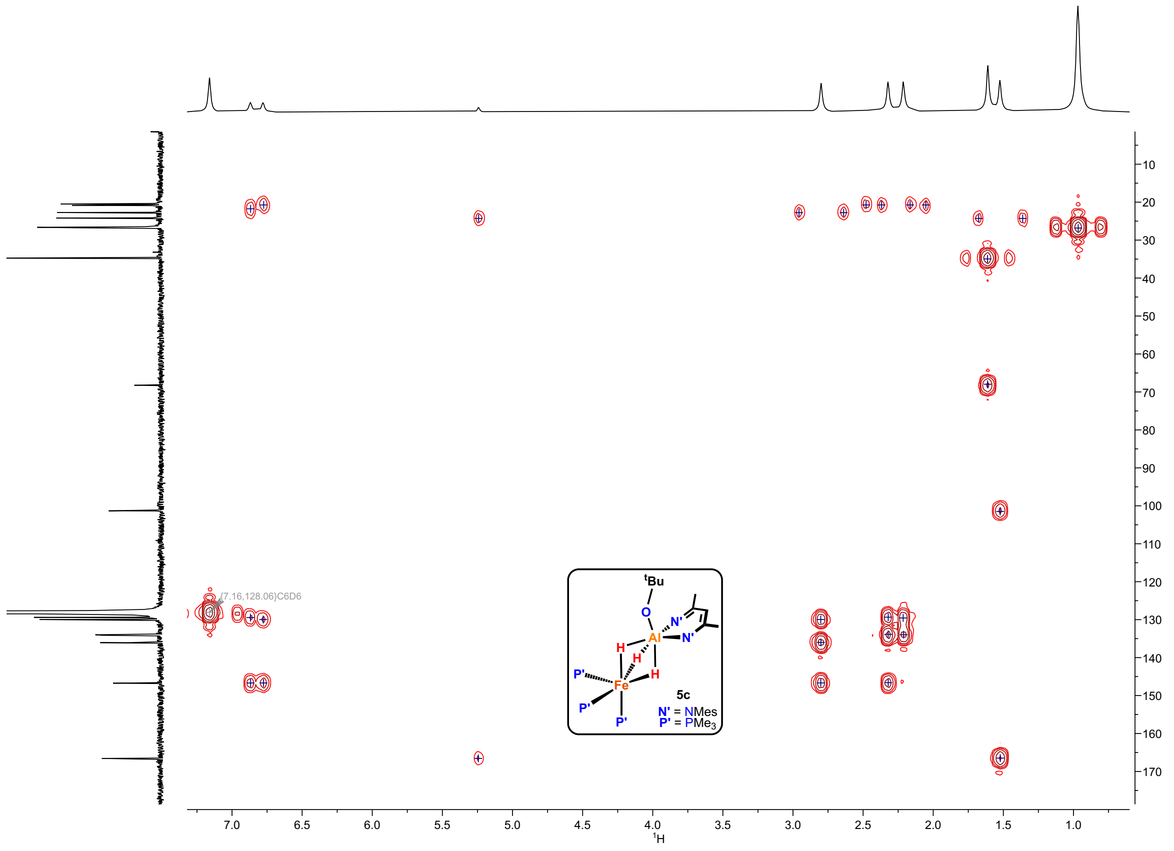

Figure S115 ^{1}H-^{13}C HMBC NMR spectrum of **5c** (C_6D_6, 298 K, 400 MHz).

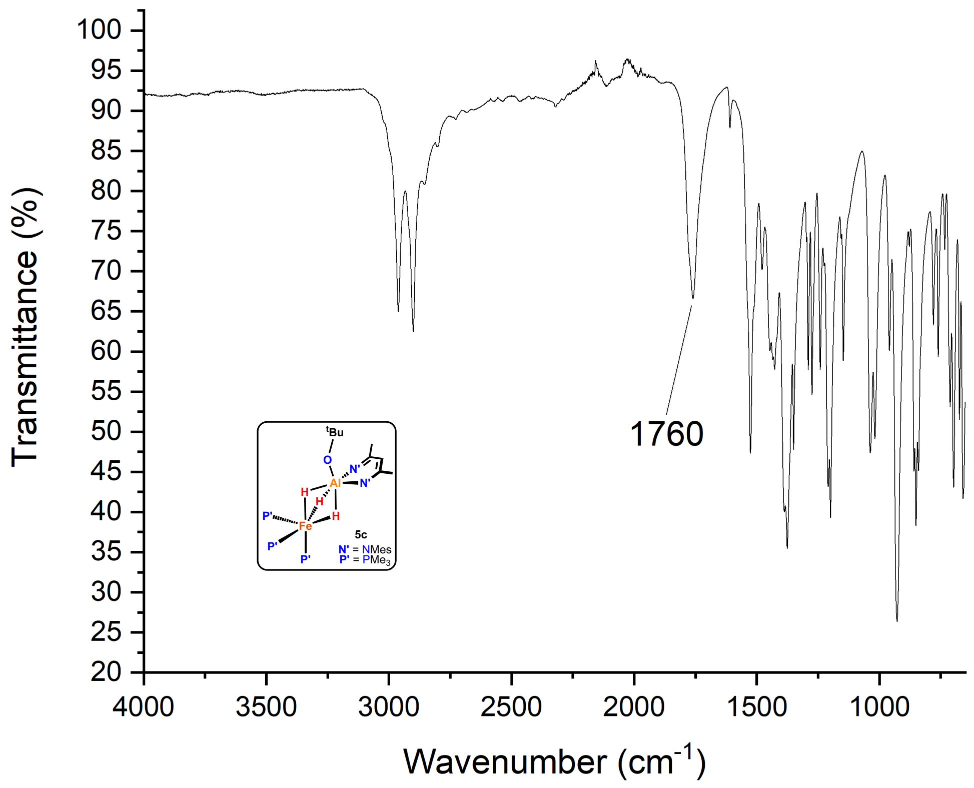

Figure S116 FT-IR spectrum of **5c** (solid, 298 K, ATR).

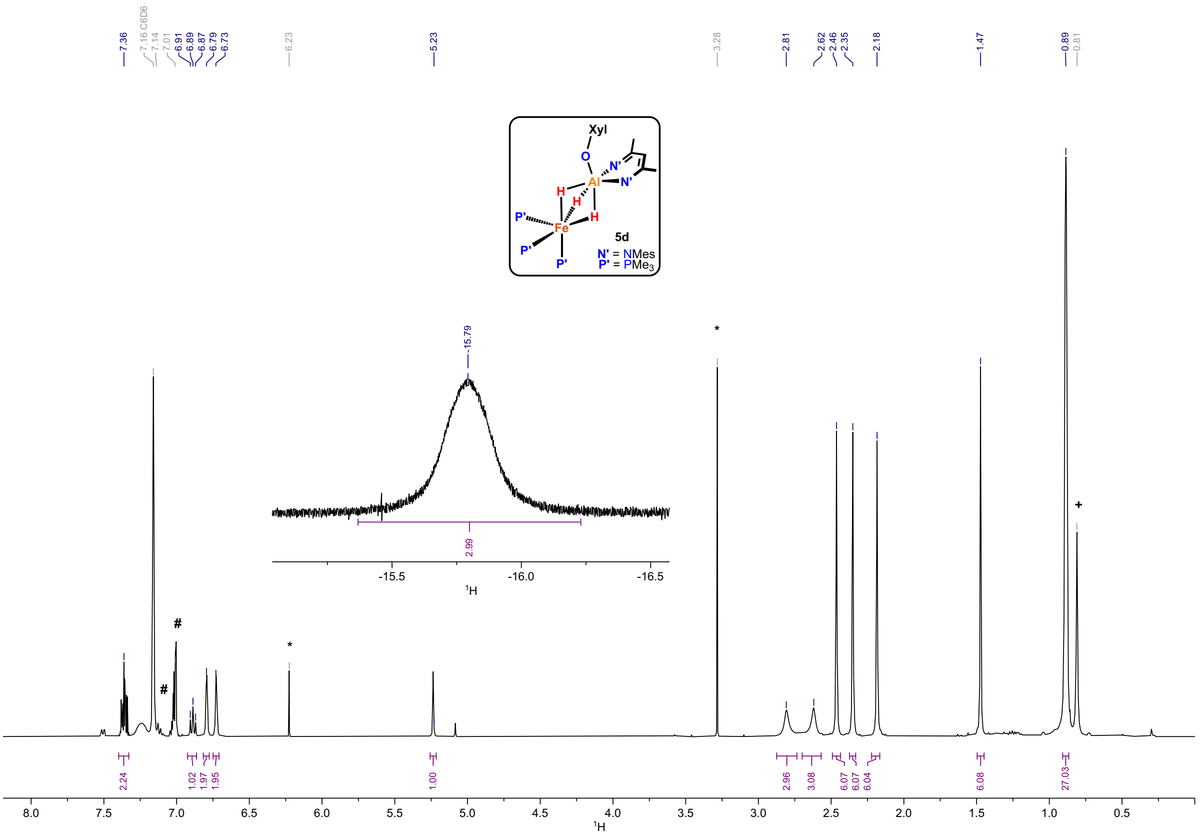

Figure S117 *In situ* ^{1}H NMR of **5d** (C_6D_6, 298 K, 500 MHz). #: PPh_3 (internal standard in capillary), *: 1,3,5-trimethoxybenzene (internal standard in capillary), +: trimethylphosphine.

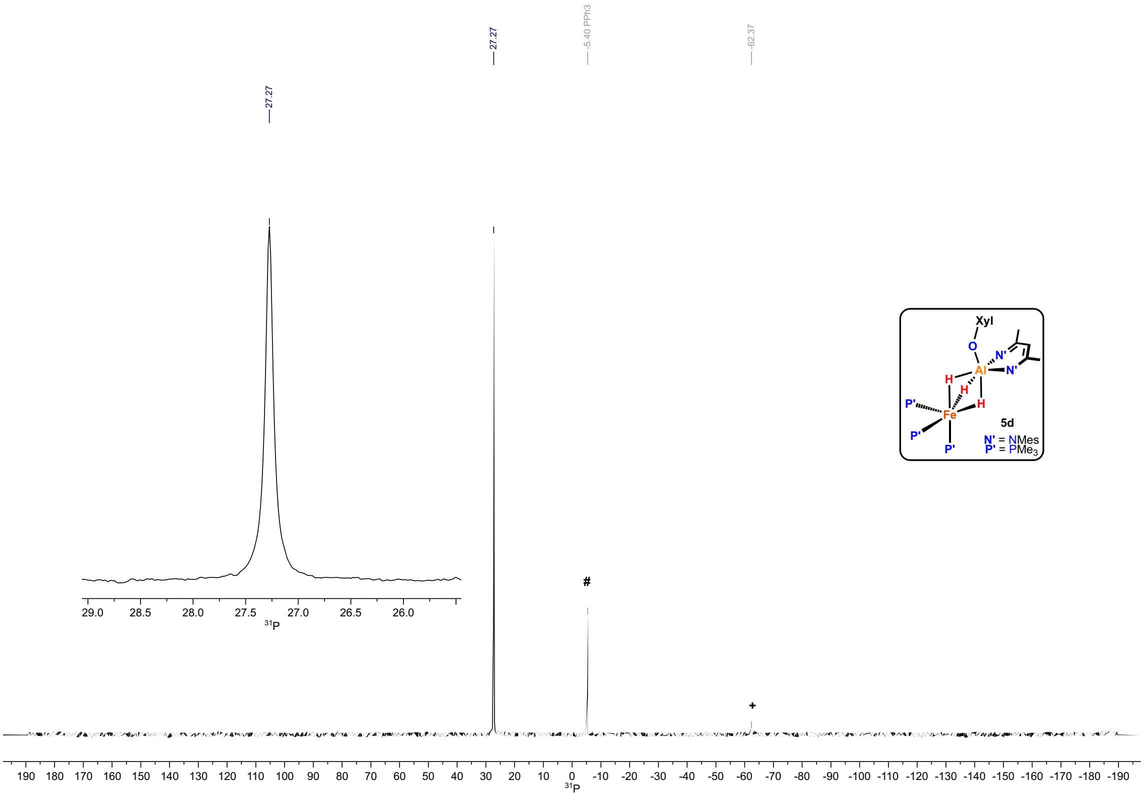

Figure S118 *In situ* $^{31}P\{^{1}H\}$ NMR of **5d** (C_6D_6, 298 K, 500 MHz). #: PPh_3 (internal standard in capillary), +: trimethylphosphine.

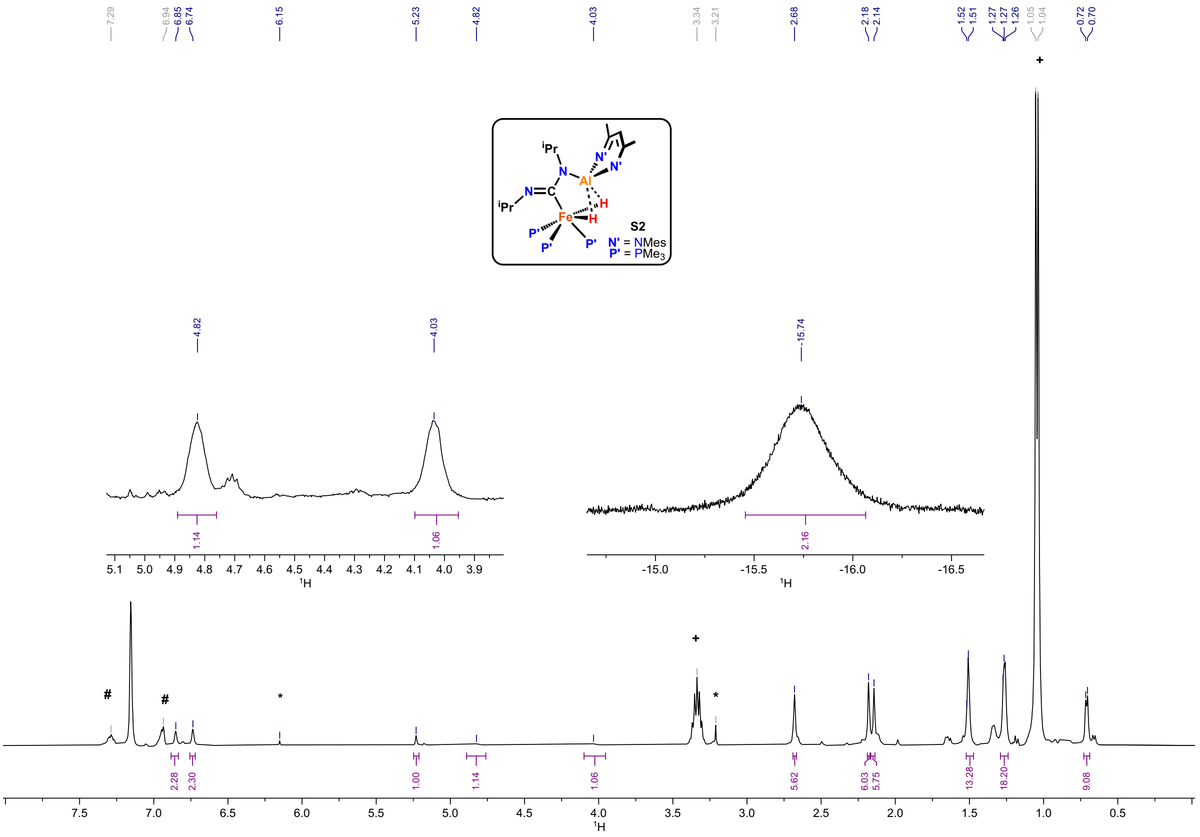

Figure S119 *In situ* ^{1}H NMR of **S2** (C_6D_6, 298 K, 400 MHz). #: PPh_3 (internal standard in capillary), * 1,3,5-trimethoxybenzene (internal standard in capillary), +: excess $^{i}PrNCN^{i}Pr$.

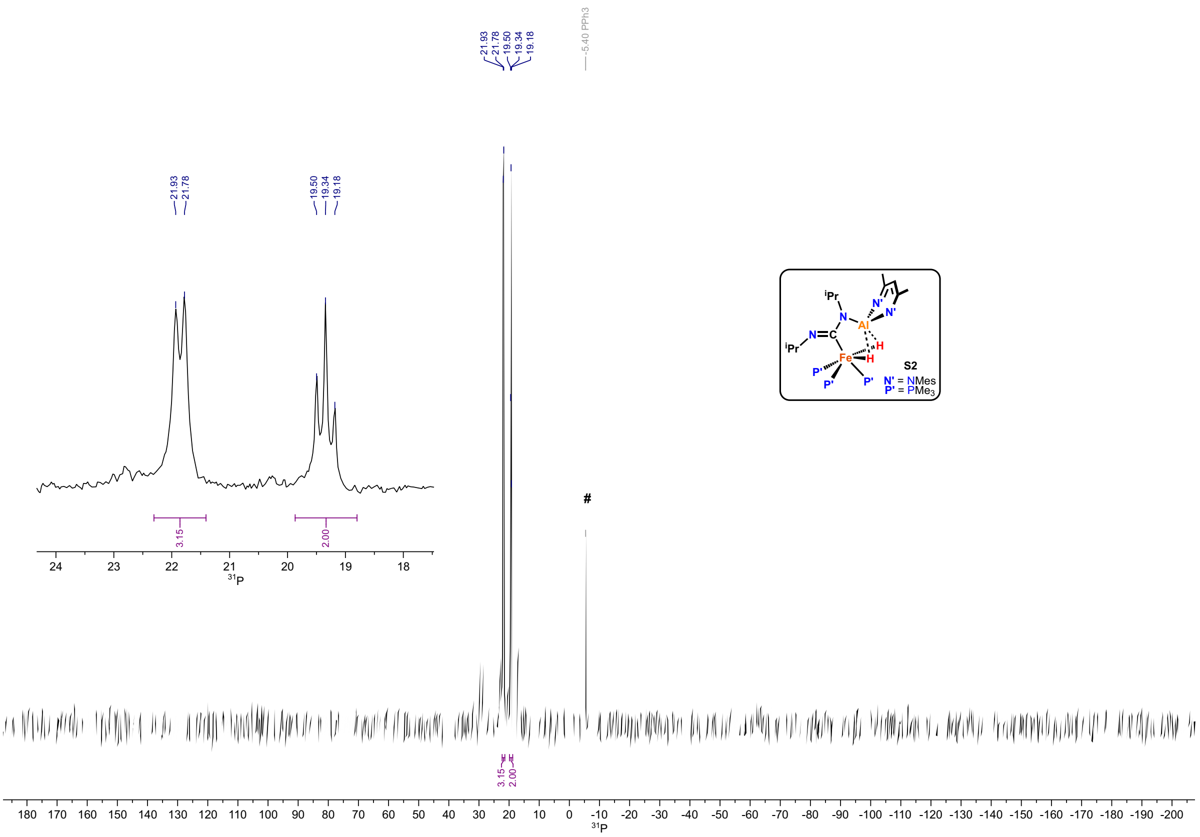

Figure S120 *In situ* $^{31}P\{^{1}H\}$ NMR of **S2** (C_6D_6, 298 K, 162 MHz). #: PPh_3 (internal standard in capillary).

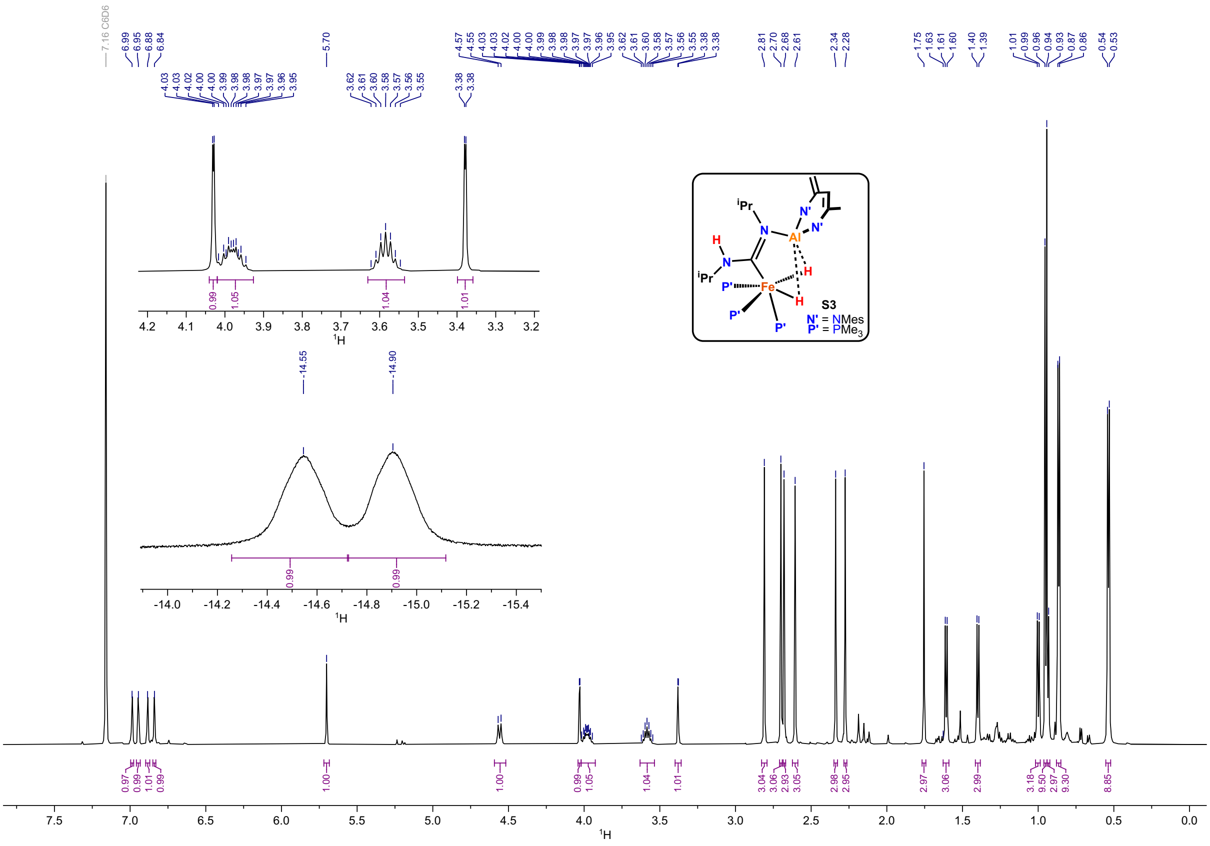

Figure S121 ^{1}H NMR spectrum of **S3** (C_6D_6, 298 K, 500 MHz).

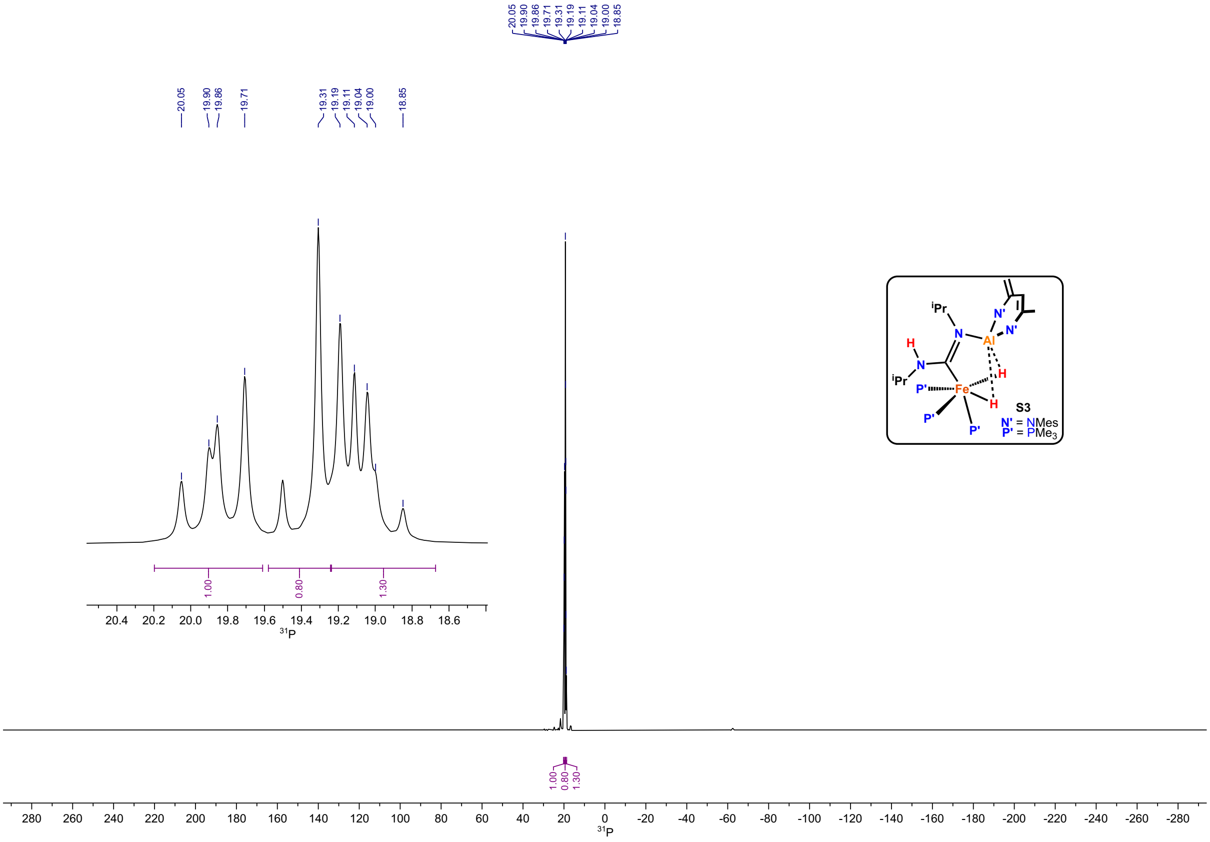

Figure S122 $^{31}P\{^{1}H\}$ NMR spectrum of **S3** (C_6D_6, 298 K, 202 MHz).

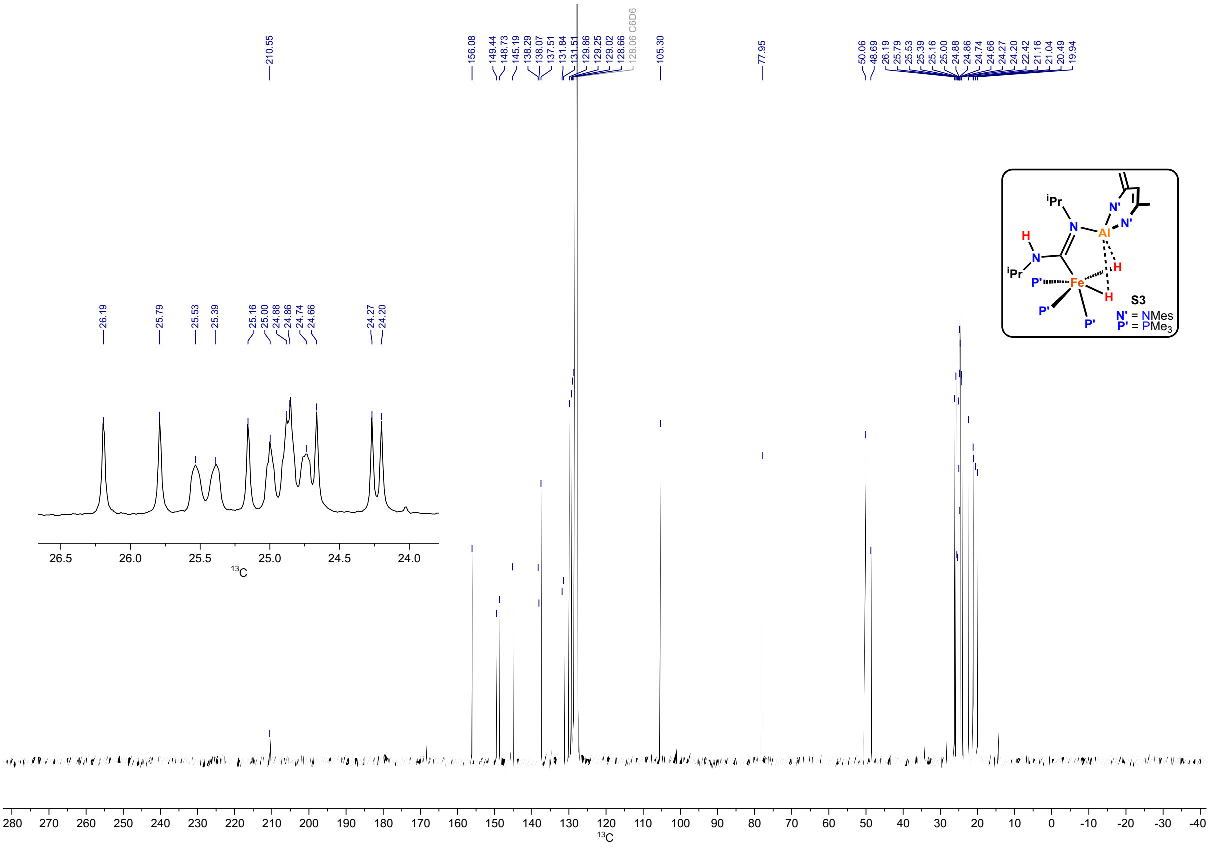

Figure S123 ^{13}C{^{1}H} NMR spectrum of **S3** (C_6D_6, 298 K, 126 MHz).

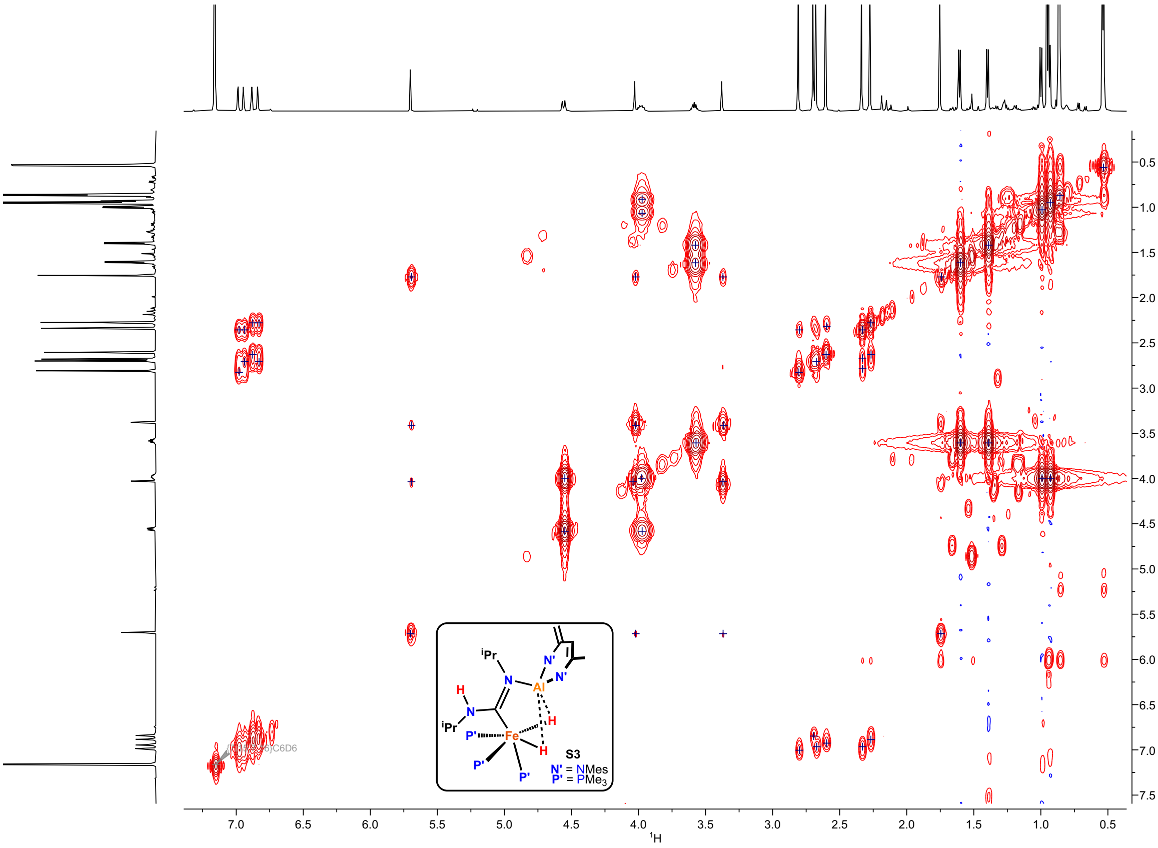

Figure S124 ^{1}H-^{1}H COSY NMR spectrum of **S3** (C_6D_6, 298 K, 500 MHz).

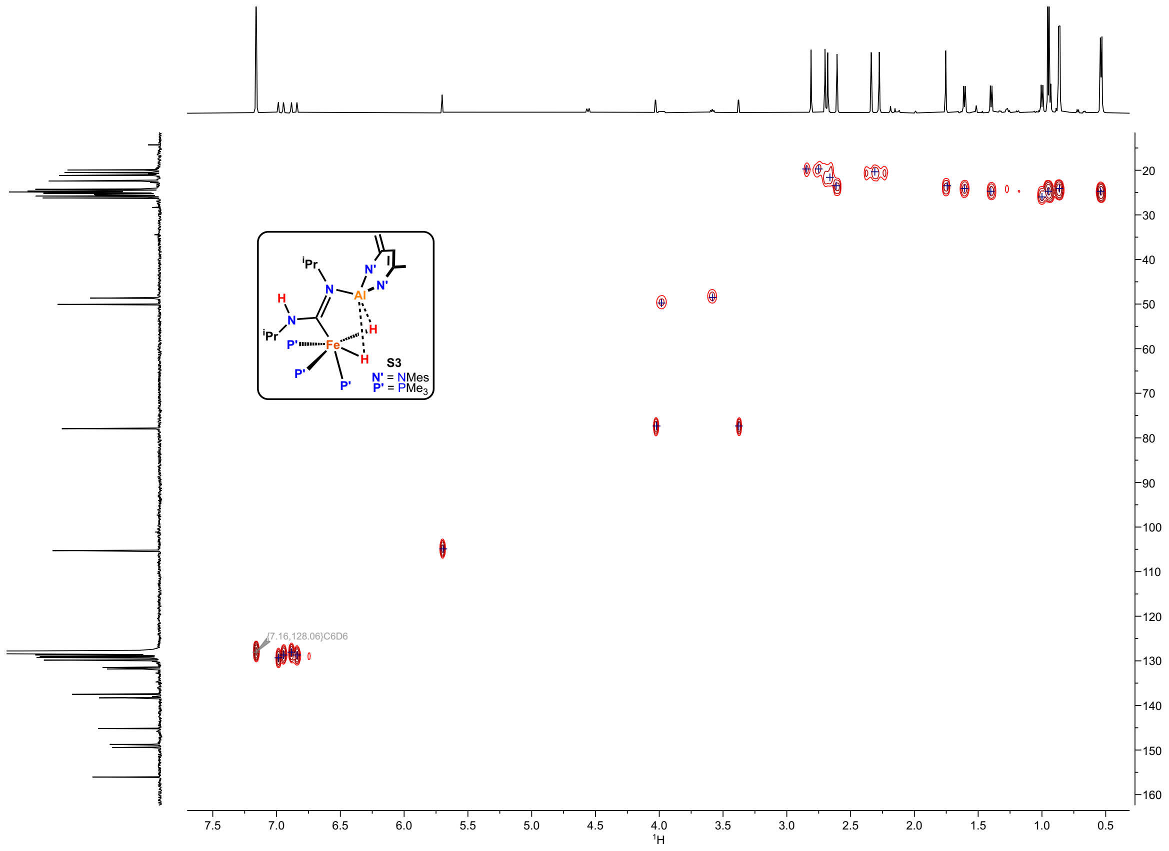

Figure S125 ^1H-^{13}C HSQC NMR spectrum of **S3** (C_6D_6, 298 K, 500 MHz).

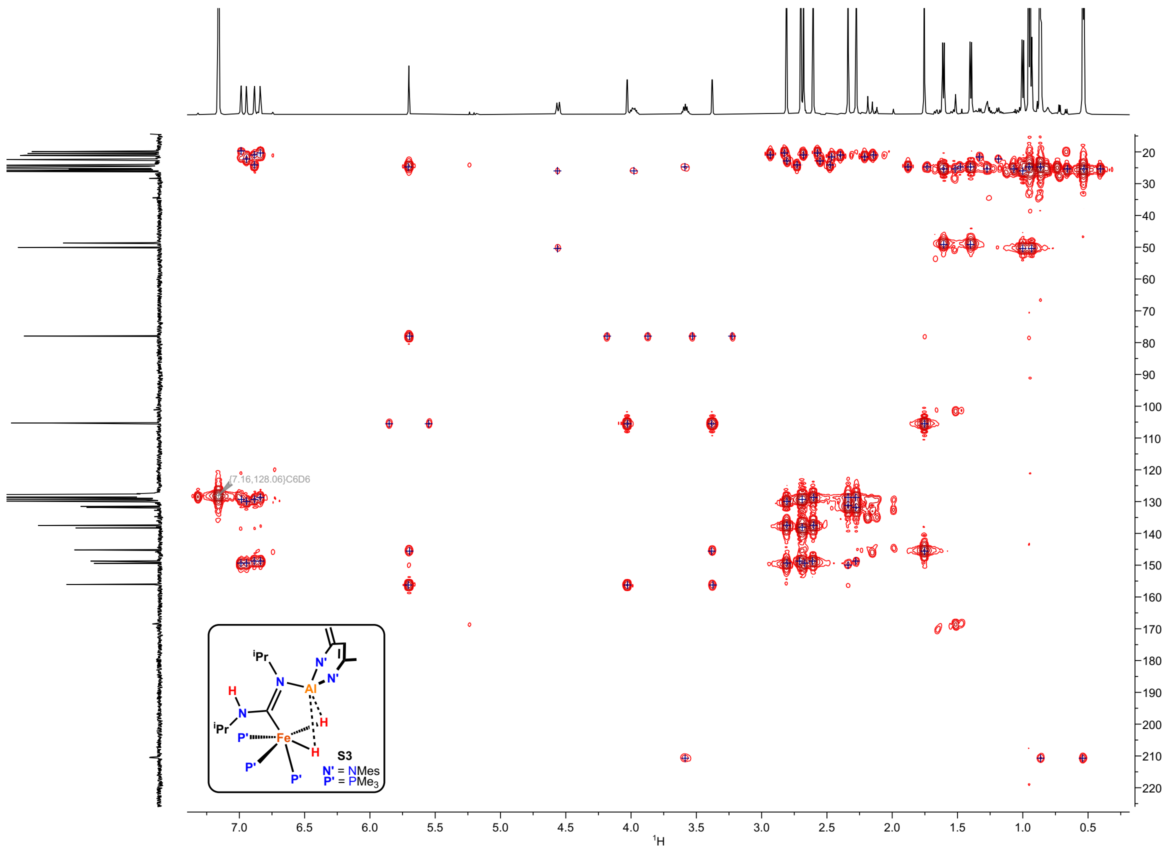

Figure S126 ^1H-^{13}C HMBC NMR spectrum of **S3** (C_6D_6, 298 K, 500 MHz).

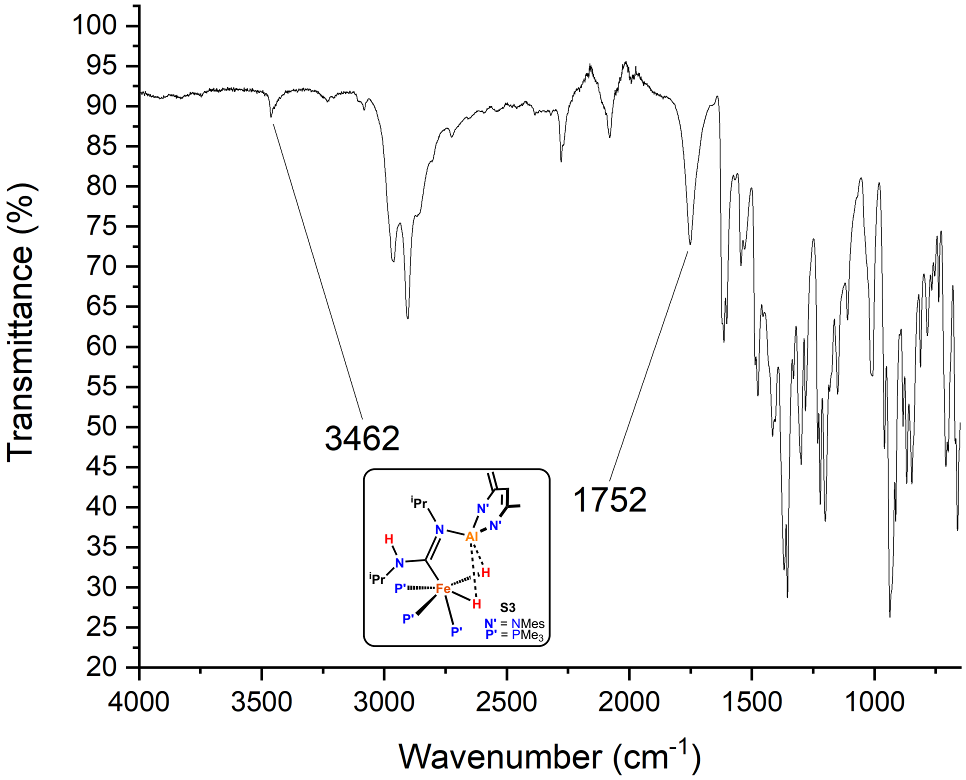

Figure S127 FT-IR spectrum of **S3** (thin film, 298 K, ATR).

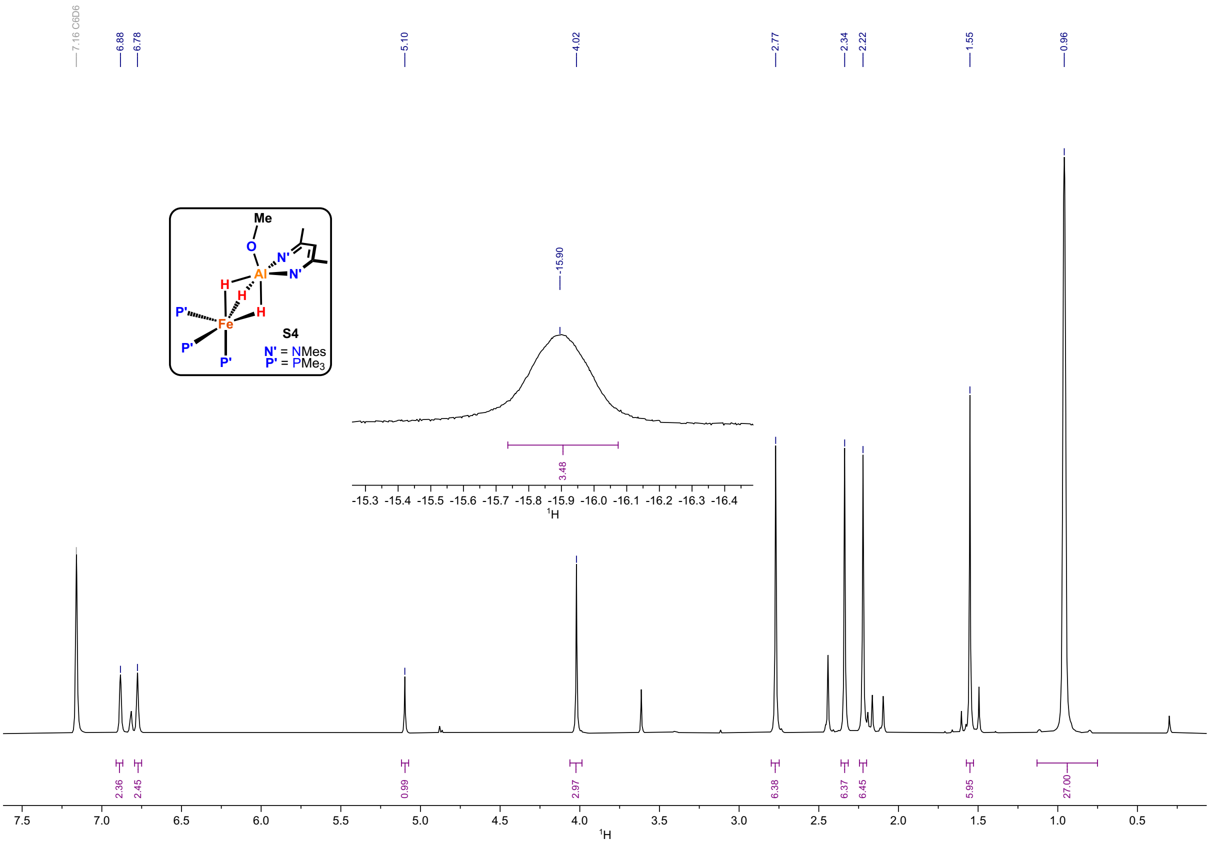

Figure S128 ^{1}H NMR of **S4** (C_6D_6, 298 K, 400 MHz).

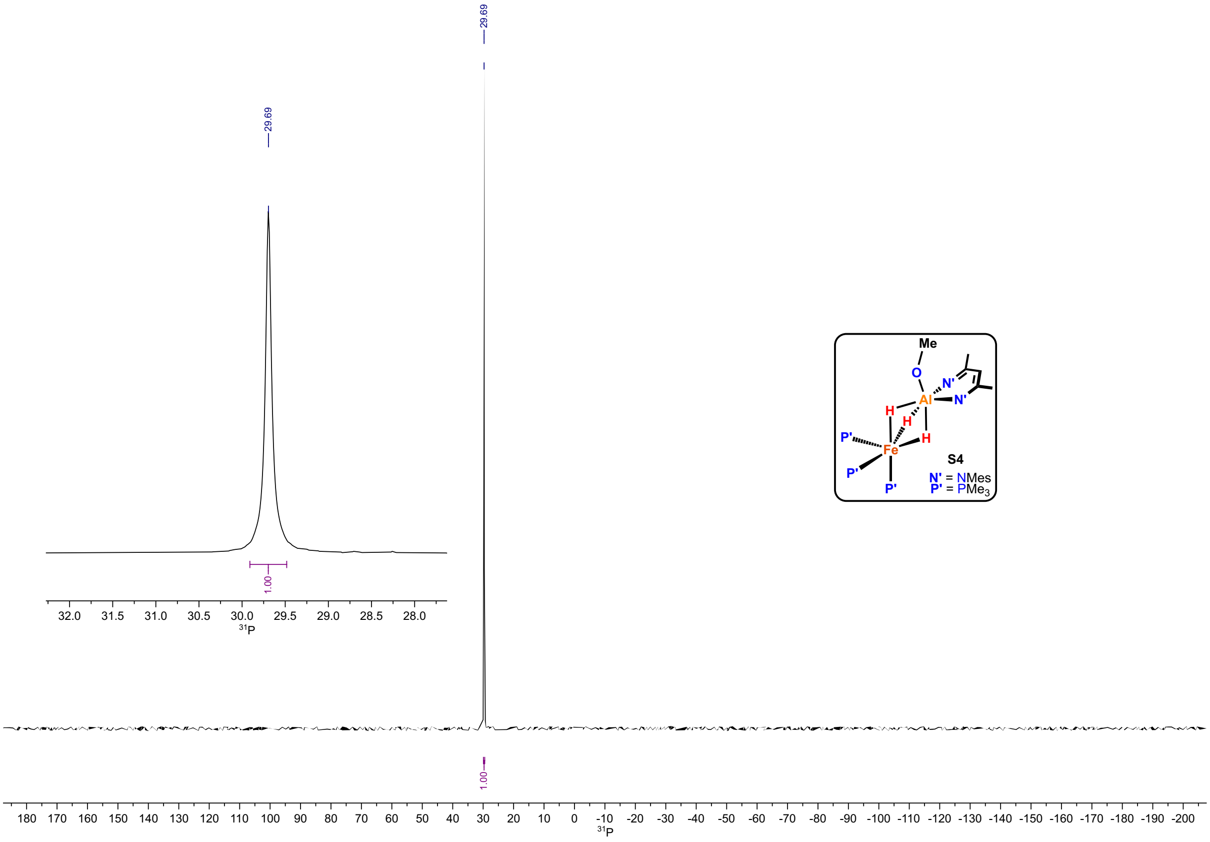

Figure S129 $^{31}P\{^{1}H\}$ NMR of **S4** (C_6D_6, 298 K, 162 MHz).

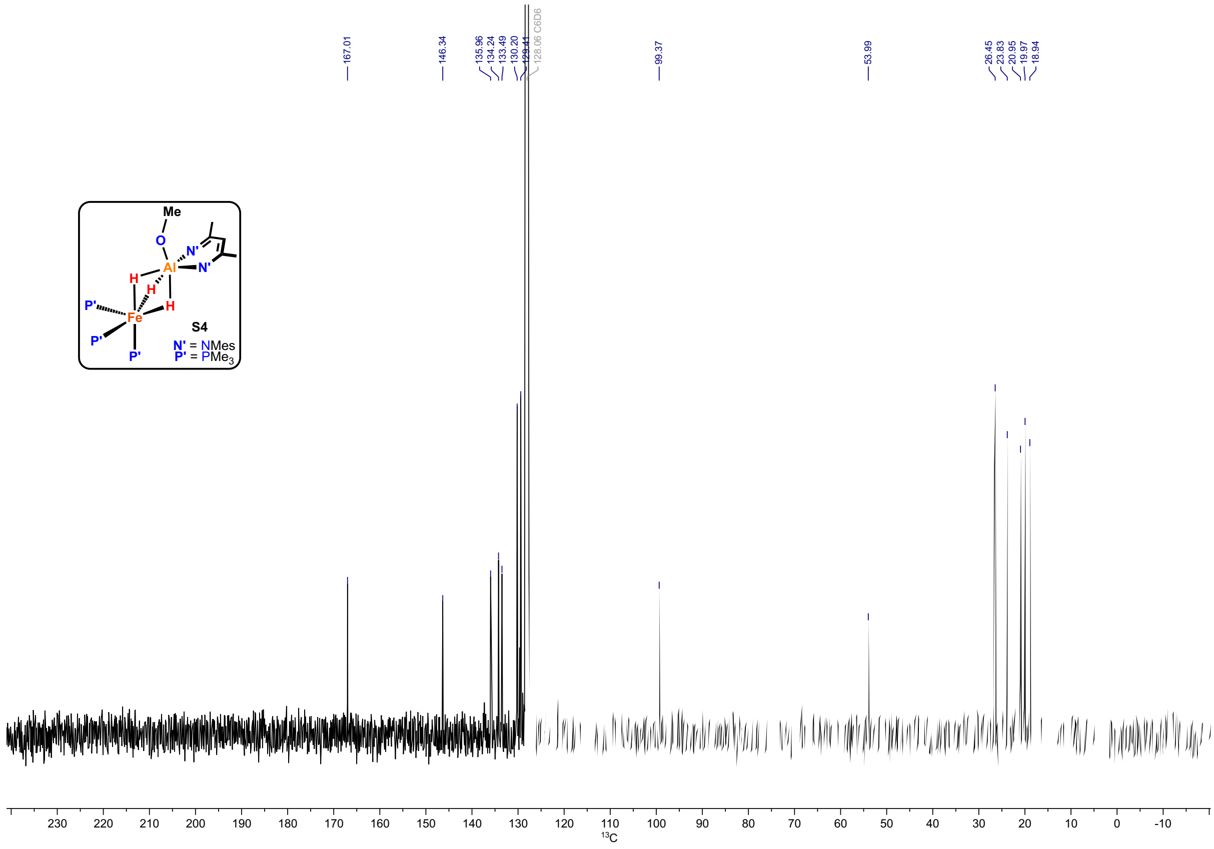

Figure S130 $^{13}C\{^1H\}$ NMR of **S4** (C_6D_6, 298 K, 101 MHz).

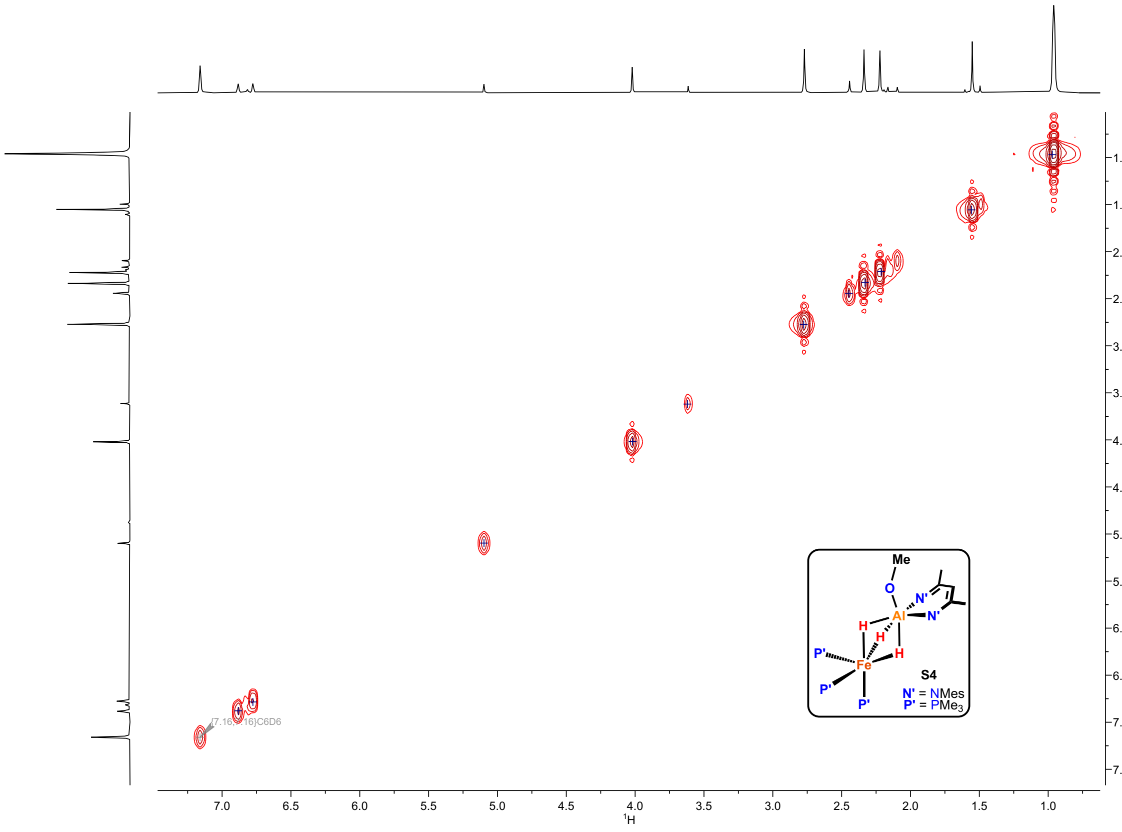

Figure S131 ^1H-^1H COSY NMR of **S4** (C_6D_6, 298 K, 400 MHz).

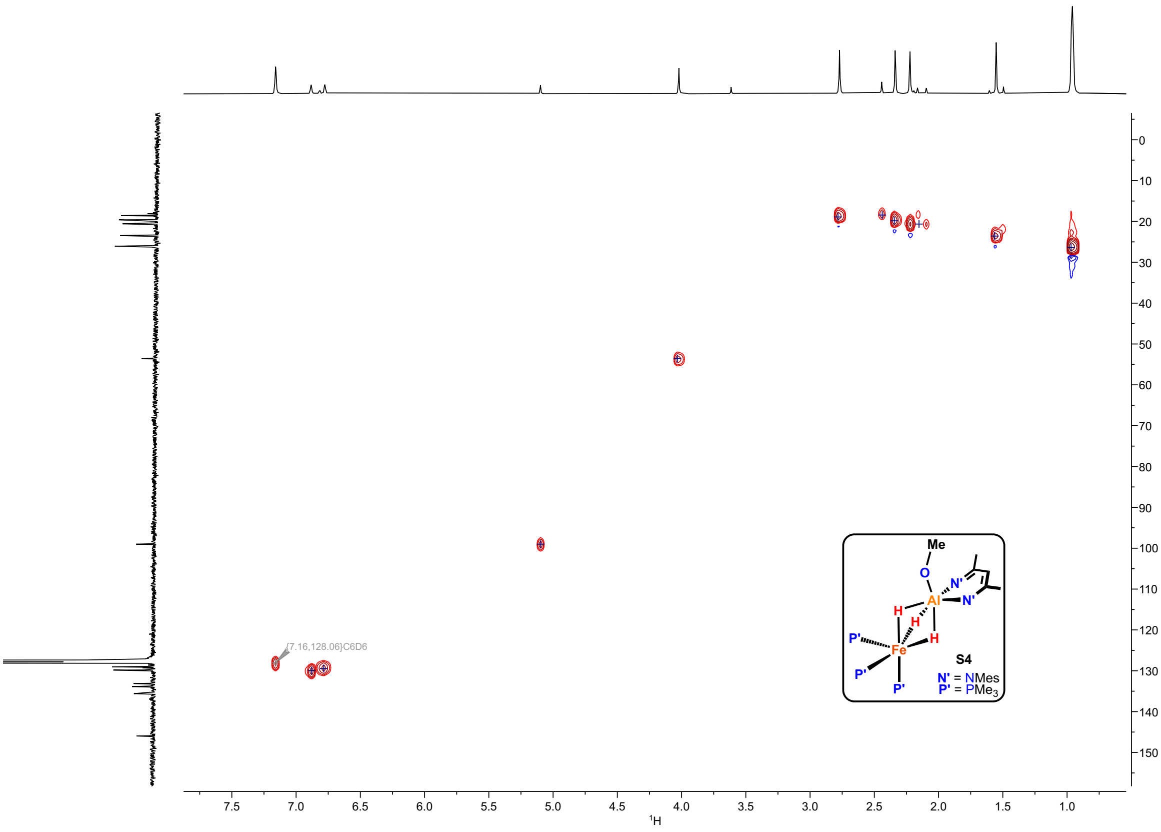

Figure S132 ^{1}H-^{13}C HSQC NMR of **S4** (C_6D_6, 298 K, 400 MHz).

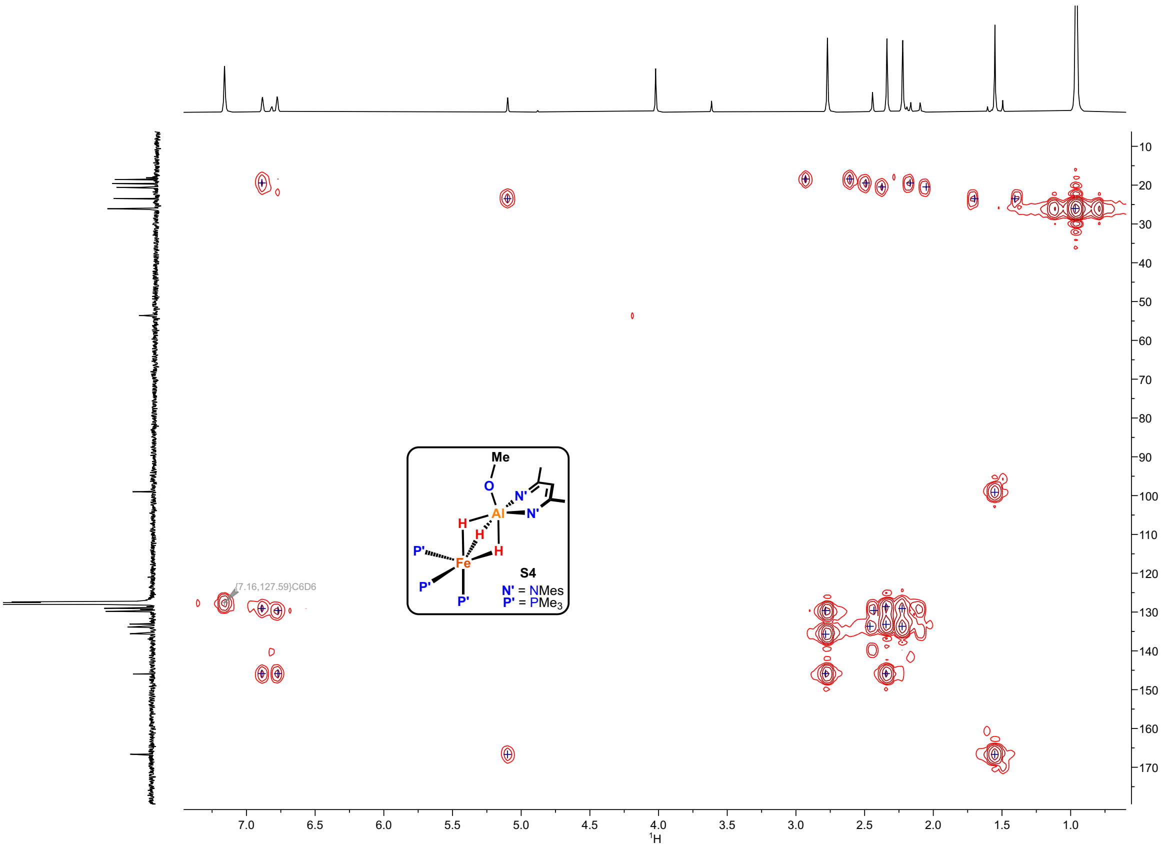

Figure S133 ^{1}H-^{13}C HMBC NMR of **S4** (C_6D_6, 298 K, 400 MHz).

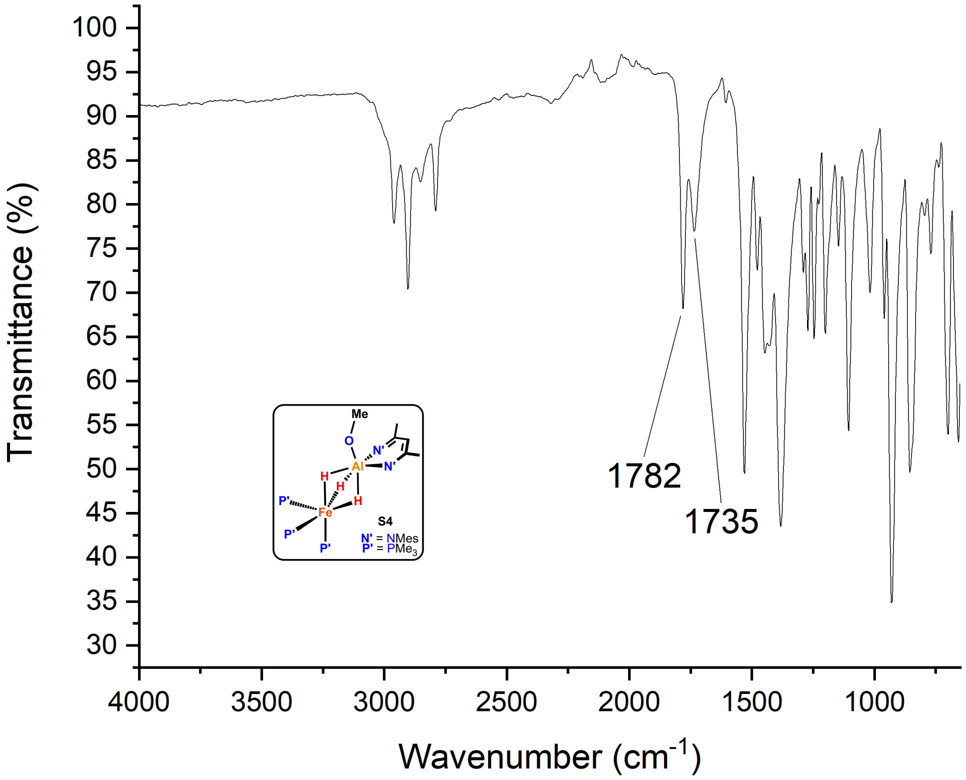

Figure S134 FT-IR spectrum of **S4** (solid, 298 K, ATR).

S5. References

1 G. R. Fulmer, A. J. M. Miller, N. H. Sherden, H. E. Gottlieb, A. Nudelman, B. M. Stoltz, J. E. Bercaw and K. I. Goldberg, *Organometallics*, 2010, **29**, 2176–2179.

2 N. Gorgas, A. J. P. White and M. R. Crimmin, *J. Am. Chem. Soc.*, 2022, **144**, 8770–8777.

3 B. Stadler, N. Gorgas, S. J. Elliott and M. R. Crimmin, *Angew. Chemie Int. Ed.*, 2024, **63**, e202408257.

4 M. Brookhart, B. Grant and A. F. Volpe, *Organometallics*, 1992, **11**, 3920–3922.

5 G. M. Sheldrick, *Acta Crystallogr. Sect. C Struct. Chem.*, 2015, **71**, 3–8.

6 O. V. Dolomanov, L. J. Bourhis, R. J. Gildea, J. A. K. Howard and H. Puschmann, *J. Appl. Crystallogr.*, 2009, **42**, 339–341.

7 G. M. Sheldrick, *Acta Crystallogr. Sect. A Found. Adv.*, 2015, **71**, 3–8.

8 F. Kleemiss, O. V. Dolomanov, M. Bodensteiner, N. Peyerimhoff, L. Midgley, L. J. Bourhis, A. Genoni, L. A. Malaspina, D. Jayatilaka, J. L. Spencer, F. White, B. Grundkötter-Stock, S. Steinhauer, D. Lentz, H. Puschmann and S. Grabowsky, *Chem. Sci.*, 2021, **12**, 1675–1692.

9 F. Neese, *WIREs Comput. Mol. Sci.*, 2012, **2**, 73–78.

10 C. Neese, F.; Wennmohs, F.; Becker, U.; Riplinger, F. Neese, F. Wennmohs, U. Becker and C. Riplinger, *J. Chem. Phys.*, 2020, **152**, 224108.

11 F. Neese, *WIREs Comput. Mol. Sci.*, 2022, **12**, e1606.

12 S. Grimme, F. Bohle, A. Hansen, P. Pracht, S. Spicher and M. Stahn, *J. Phys. Chem. A*, 2021, **125**, 4039–4054.

13 J. Sun, A. Ruzsinszky and J. Perdew, *Phys. Rev. Lett.*, 2015, **115**, 036402.

14 A. P. Bartók and J. R. Yates, *J. Chem. Phys.*, 2019, **150**, 161101.

15 J. W. Furness, A. D. Kaplan, J. Ning, J. P. Perdew and J. Sun, *J. Phys. Chem. Lett.*, 2020, **11**, 8208–8215.

16 J. W. Furness, A. D. Kaplan, J. Ning, J. P. Perdew and J. Sun, *J. Phys. Chem. Lett.*, 2020, **11**, 9248–9248.

17 J. W. Furness, A. D. Kaplan, J. Ning, J. P. Perdew and J. Sun, *J. Chem. Phys.*, 2022, **156**, 34109.

18 F. Weigend and R. Ahlrichs, *Phys. Chem. Chem. Phys.*, 2005, **7**, 3297.

19 E. Caldeweyher, S. Ehlert, A. Hansen, H. Neugebauer, S. Spicher, C. Bannwarth and S. Grimme, *J. Chem. Phys.*, 2019, **150**, 154122.

20 H. Kruse and S. Grimme, *J. Chem. Phys.*, 2012, **136**, 154101.

21 F. Neese, *J. Comput. Chem.*, 2003, **24**, 1740–1747.

22 F. Neese, F. Wennmohs, A. Hansen and U. Becker, *Chem. Phys.*, 2009, **356**, 98–109.

23 B. Helmich-Paris, B. de Souza, F. Neese and R. Izsák, *J. Chem. Phys.*, 2021, **155**, 104109.

24 F. Weigend, *Phys. Chem. Chem. Phys.*, 2006, **8**, 1057–1065.

25 C. Adamo and V. Barone, *J. Chem. Phys.*, 1998, **108**, 664–675.

26 A. V. Marenich, C. J. Cramer and D. G. Truhlar, *J. Phys. Chem. B*, 2009, **113**, 6378–6396.

27 B. de Souza, *Angew. Chemie Int. Ed.*, 2025, e202500393.

28 C. Bannwarth, S. Ehlert and S. Grimme, *J. Chem. Theory Comput.*, 2019, **15**, 1652–1671.

29 S. Ehlert, M. Stahn, S. Spicher and S. Grimme, *J. Chem. Theory Comput.*, 2021, **17**, 4250–4261.

30 S. Spicher and S. Grimme, *Angew. Chemie Int. Ed.*, 2020, **59**, 15665–15673.

31 E. D. Glendening, K. B. J, A. E. Reed, J. E. Carpenter, J. A. Bohmann, C. M. Morales, P. Karafiloglou, C. R. Landis and F. Weinhold, 2018.

32 G. Knizia, *J. Chem. Theory Comput.*, 2013, **9**, 4834–4843.

33 M. P. Mitoraj, A. Michalak and T. Ziegler, *J. Chem. Theory Comput.*, 2009, **5**, 962–975.

34 S. Grimme and A. Hansen, *Angew. Chemie Int. Ed.*, 2015, **54**, 12308–12313.

35 N. Gorgas, B. Stadler, A. J. P. White and M. R. Crimmin, *J. Am. Chem. Soc.*, 2024, **146**, 4252–4259.

36 M. Müller, A. Hansen and S. Grimme, *J. Chem. Phys.*, 2023, **158**, 14103.

37 Y. Tawada, T. Tsuneda, S. Yanagisawa, T. Yanai and K. Hirao, *J. Chem. Phys.*, 2004, **120**, 8425–8433.
